# Supplementary material for: GRA12 is a common virulence factor across Toxoplasma gondii strains and mouse subspecies
Source: Nat Commun. 2025 Apr 16;16:3570. doi: 10.1038/s41467-025-58876-2 (PMC12003902; doi:10.1038/s41467-025-58876-2)
Supplement: Supplementary file 1 — Supplementary Information [file 41467_2025_58876_MOESM1_ESM.pdf]

a

Strain	Transfection efficiency [%]	sgRNA coverage [# parasite/sgRNA]
VAND	0.07	1625
RH	0.33	17431
PRU	0.45	6822
VEG	0.40	4484

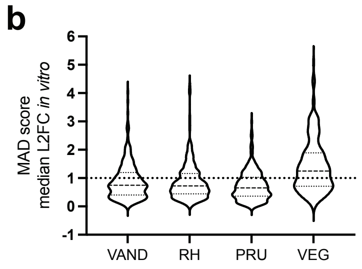

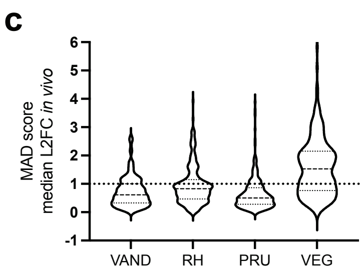

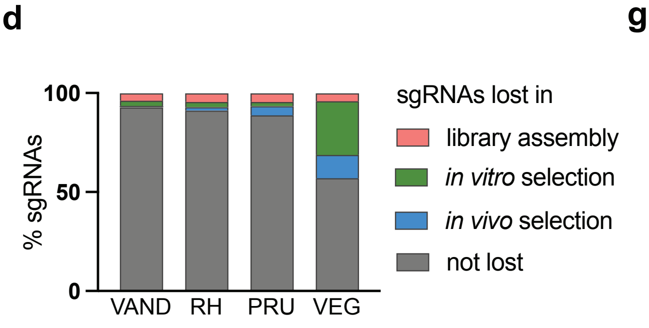

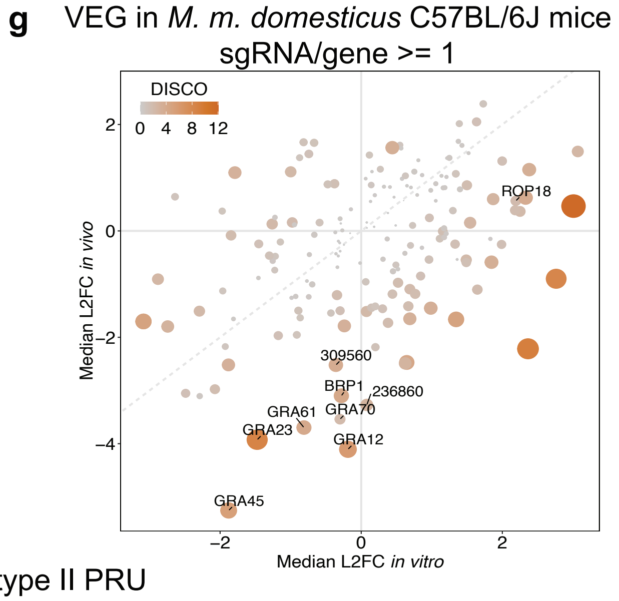

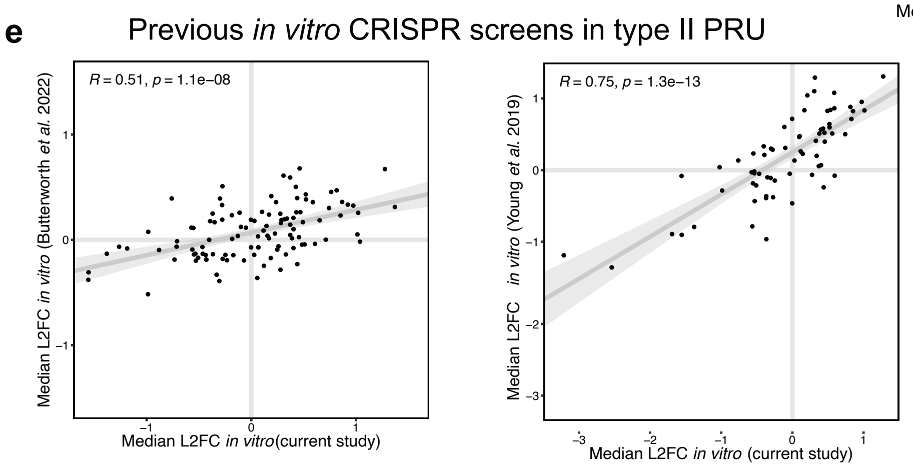

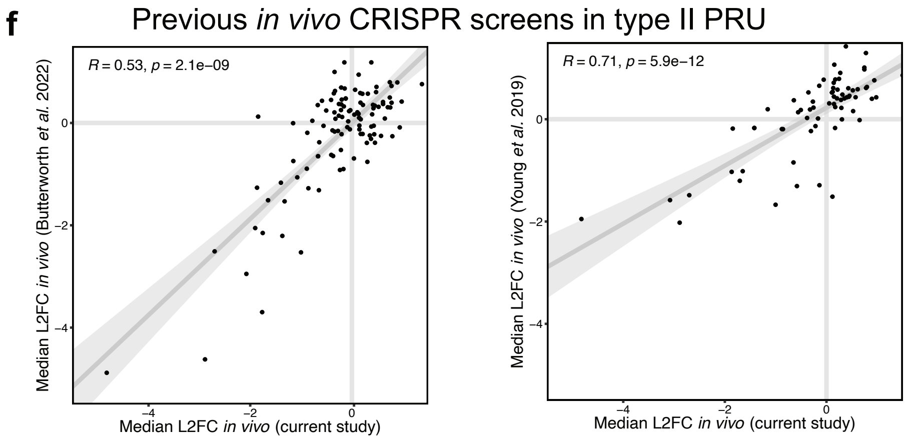

Supplementary Fig. 1. *In vivo* CRISPR screens of the secretome of four *Toxoplasma* strains. a Transfection efficiency and relative sgRNA coverage in each screen. **b** Median Absolute Deviation (MAD) of the log2 fold change (L2FC) of sgRNA read counts between the plasmid and the inoculum and **c** of the inoculum and the mouse peritoneum in each screen. **d** sgRNA loss at each screen step. **e** Correlation of the median L2FC *in vitro* and **f** *in vivo* of the current type II screen with previous screens performed in our research group [42,43]. **g** Scatter plots of the median L2FC for each gene *in vitro* and *in vivo* of the CRISPR screen of VEG in C57BL/6J mice. The colour and size of each point reflects the Discordance/Concordance (DISCO) score, and the dashed grey line indicates equal L2FC. A threshold of 1 sgRNA per gene was applied compared to 3 sgRNAs per gene in Fig. 1b.

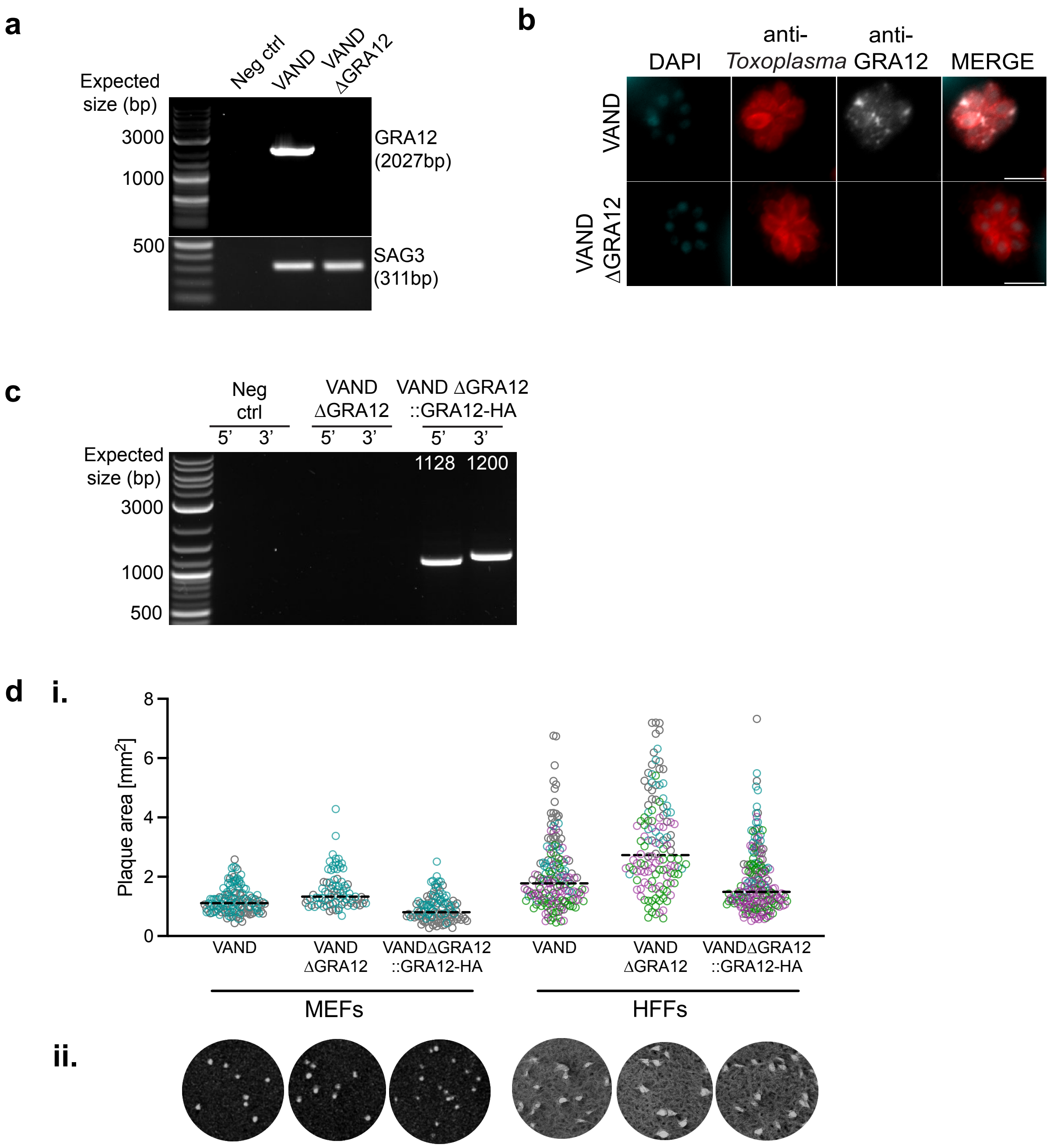

Supplementary Fig. 2. Establishment of VAND ΔGRA12 and ΔGRA12::GRA12-HA strains. **a** PCR amplification of the GRA12 locus in the parental VAND and ΔGRA12 strains. Amplification of SAG3 was performed as control. **b** Immunofluorescence verification of GRA12 in Suppl Fig 2d the VAND ΔGRA12 strain. Scale bar represents 10 µm. **c** PCR validation of the VAND ΔGRA12::GRA12-HA strain. **d** Scatter plot of the plaque size of VAND parental and derived clones in MEFs (N=2) and HFFs (N=4), bar represents the median and different colours refer to individual biological replicates (**i**), and respective images (**ii**). Source data are provided as a Source Data file.

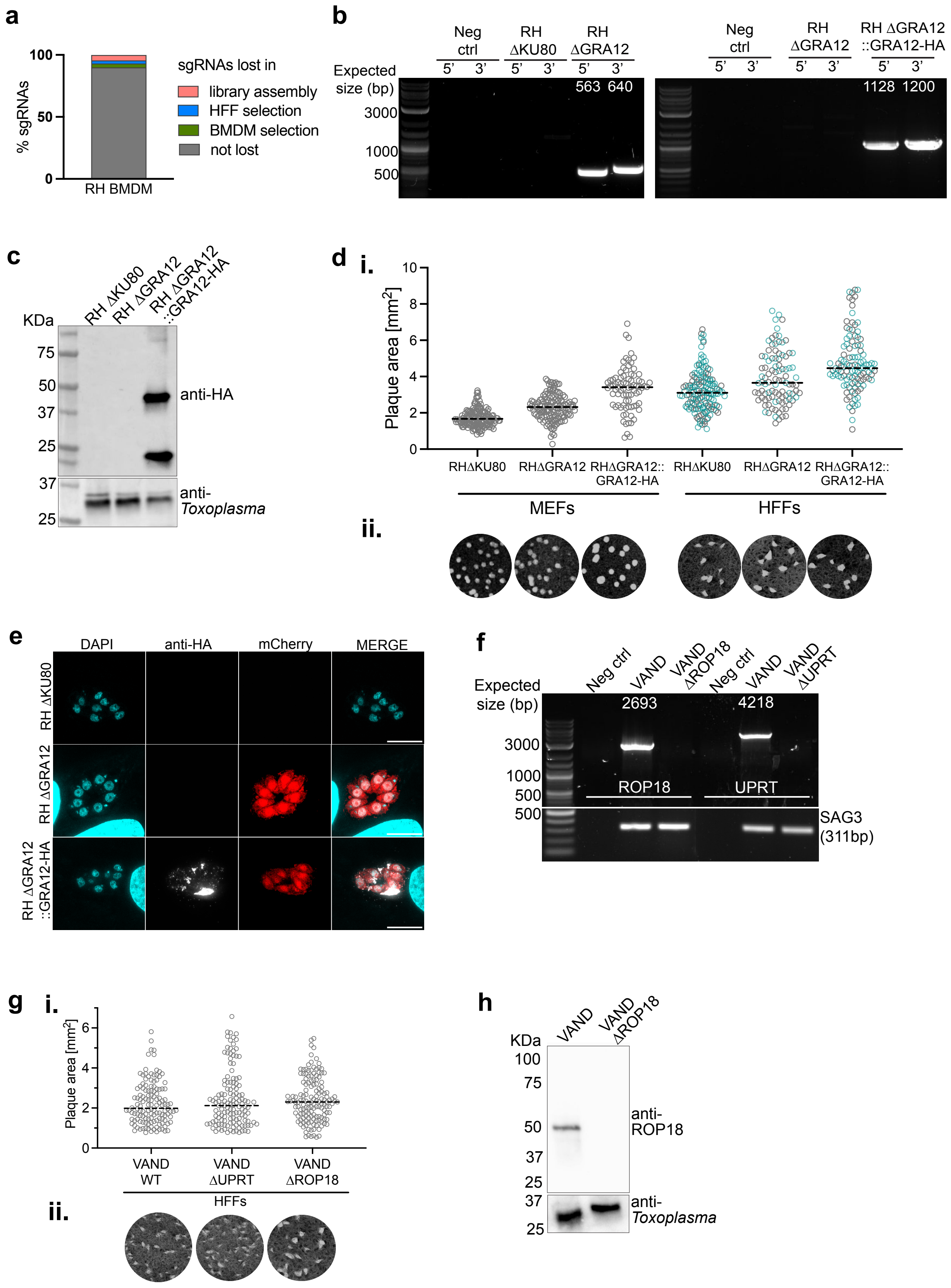
a
100
50
0
% sgRNAs
RH BMDM
sgRNAs lost in
library assembly
HFF selection
BMDM selection
not lost
b
Expected size (bp)
Neg ctrl
RH ΔKU80
RH ΔGRA12
RH ΔGRA12 ::GRA12-HA
5' 3'
563 640
1128 1200
3000
1000
500
c
KDa
RH ΔKU80
RH ΔGRA12
RH ΔGRA12 ::GRA12-HA
75
50
37
25
anti-HA
anti-Toxoplasma
d
i.
Plaque area [mm2]
RHΔKU80
RHΔGRA12
RHΔGRA12:: GRA12-HA
MEFs
HFFs
ii.
e
DAPI
anti-HA
mCherry
MERGE
RH ΔKU80
RH ΔGRA12
RH ΔGRA12 ::GRA12-HA
f
Expected size (bp)
Neg ctrl
VAND
VAND ΔROP18
VAND ΔUPRT
2693
4218
3000
1000
500
ROP18
UPRT
SAG3 (311bp)
g
i.
Plaque area [mm2]
VAND WT
VAND ΔUPRT
VAND ΔROP18
HFFs
ii.
h
KDa
VAND
VAND ΔROP18
100
75
50
37
25
anti-ROP18
anti-Toxoplasma

Supplementary Fig. 3. Creation of RH and VAND KO mutants for the validation of GRA12 as transcendent virulence factor *in vitro*. a sgRNA loss at each screen step of the type I RH CRISPR screen in BMDMs. **b** PCR validation of the RH ΔGRA12 and complemented strains. **c** Verification of the GRA12::HA expression via anti-HA detection in the RH ΔGRA12::GRA12-HA strain by western blot. **d** Plaque size of RH ΔKU80 parental and derived clones in MEFs (N=1) and HFFs (N=2), bar represents the median and different colours refer to individual biological replicates (**i**), and respective images (**ii**). **e** Immunofluorescence verification of the C-terminal HA-tagged GRA12 in the RH ΔGRA12::GRA12-HA strain. Scale bar is 10 μm. **f** PCR validation of the VAND ΔUPRT and ΔROP18. Amplification of SAG3 was performed as control. **g** Plaque size of VAND parental and derived clones ΔUPRT and ΔROP18, N=1 (**i**), and respective images (**ii**). **h** Verification of the ROP18 KO via anti-ROP18 detection in the VAND ΔROP18 strain by western blot. Source data are provided as a Source Data file.

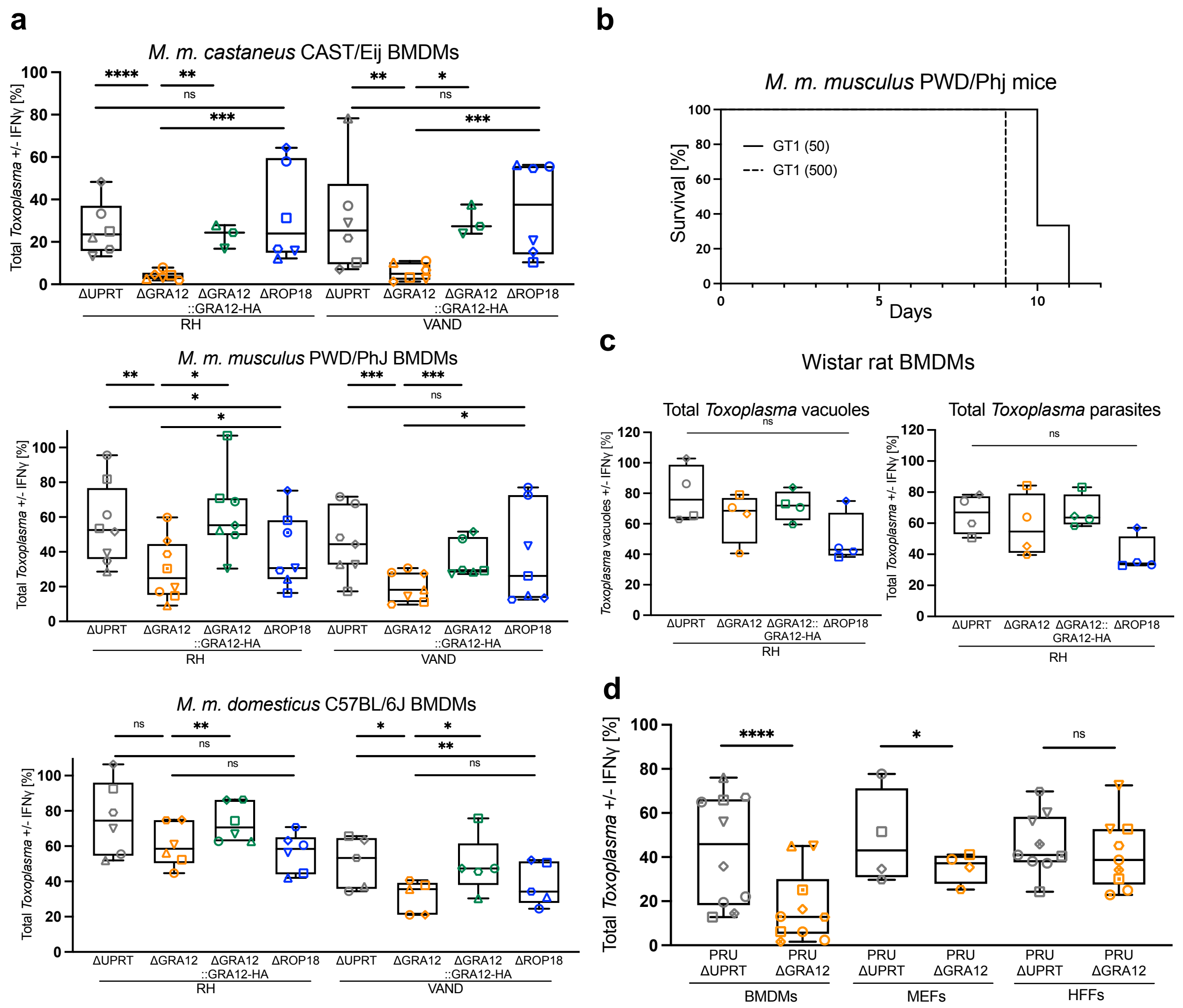

Supplementary Fig. 4. Validation of GRA12 as *Toxoplasma* transcendent virulence factor in mouse subspecies. a Quantification of high content-automated imaging of parasite numbers in IFNγ-treated BMDMs of different mouse subspecies relative to untreated controls. Cells were infected with RH ΔUPRT, ΔGRA12, ΔGRA12::GRA12-HA and ΔROP18 and *Toxoplasma* parasites were quantified at 24 h after infection. **b** Survival curve of PWD/Phj mice infected with the type I GT1 strain, dose in parenthesis. N=3 mice per group. **c** Quantification of high-content automated imaging of *Toxoplasma* infection in IFNγ-treated Wistar rat BMDMs or **d** murine embryonic fibroblasts (MEFs), human foreskin fibroblasts (HFFs) and murine BMDMs, relative to untreated controls. Cells were infected with RH ΔUPRT, ΔGRA12, ΔGRA12::GRA12-HA and ΔROP18, or PRU ΔUPRT and PRU ΔGRA12, and parasites were quantified at 24 h after infection. Symbol shapes indicate biological repeats. Significance was tested using the One-way Anova test with the Benjamini, Krieger and Yekutieli FDR correction. p * <0.05, ** <0.01, *** <0.001, **** <0.0001. Source data are provided as a Source Data file.

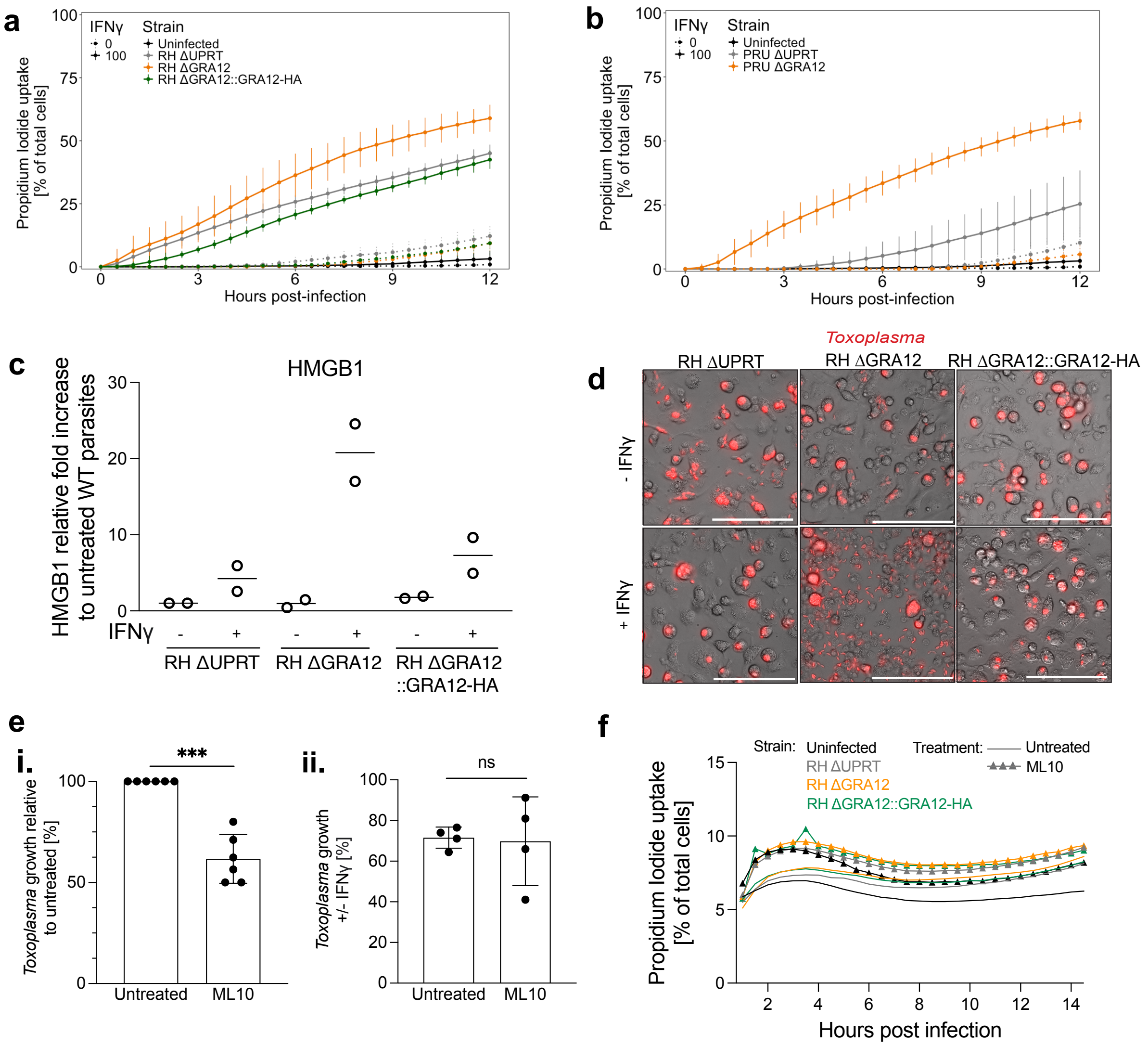

Supplementary Fig. 5. Lack of GRA12 results in IFNγ-mediated host cell death by necrosis. Time course of PWD/Phj BMDMs cell death quantified via Propidium Iodide uptake. Cells were pre-stimulated for 24 h with IFNγ (continue line) or left untreated (dashed line) and then infected with (**a**) RH ΔUPRT, ΔGRA12, ΔGRA12::GRA12-HA or (**b**) PRU ΔUPRT or ΔGRA12, or left uninfected as a control. The number of dead cells is expressed as a percentage of the total at each time point. **c** Quantification of the HMGB1 necrosis marker from the western blot analysis of the supernatant of infected BMDMs, relative to the untreated parental condition. **d** Microscopy images of IFNγ-treated or untreated C57BL/6J BMDMs, infected for 24 h with RH ΔUPRT, ΔGRA12 or ΔGRA12::GRA12-HA. Scale bar represents 100 µm. **e** Quantification by imaging of *Toxoplasma* growth in ML10-treated relative to untreated BMDMs, in naïve conditions (**i**) or in IFNγ-treated conditions relative to untreated controls (**ii**). Significance was tested using a paired t-test, p *** <0.001. **f** Time course of C57BL/6J BMDMs cell death quantified via Propidium Iodide uptake. Cells were infected with RH ΔUPRT, ΔGRA12, ΔGRA12::GRA12-HA or left uninfected, and 1 hpi treated with 1 µM ML10 (triangle symbol) or left untreated as a control (continue line). The number of dead cells is expressed as a percentage of the total at each time point. Source data are provided as a Source Data file.

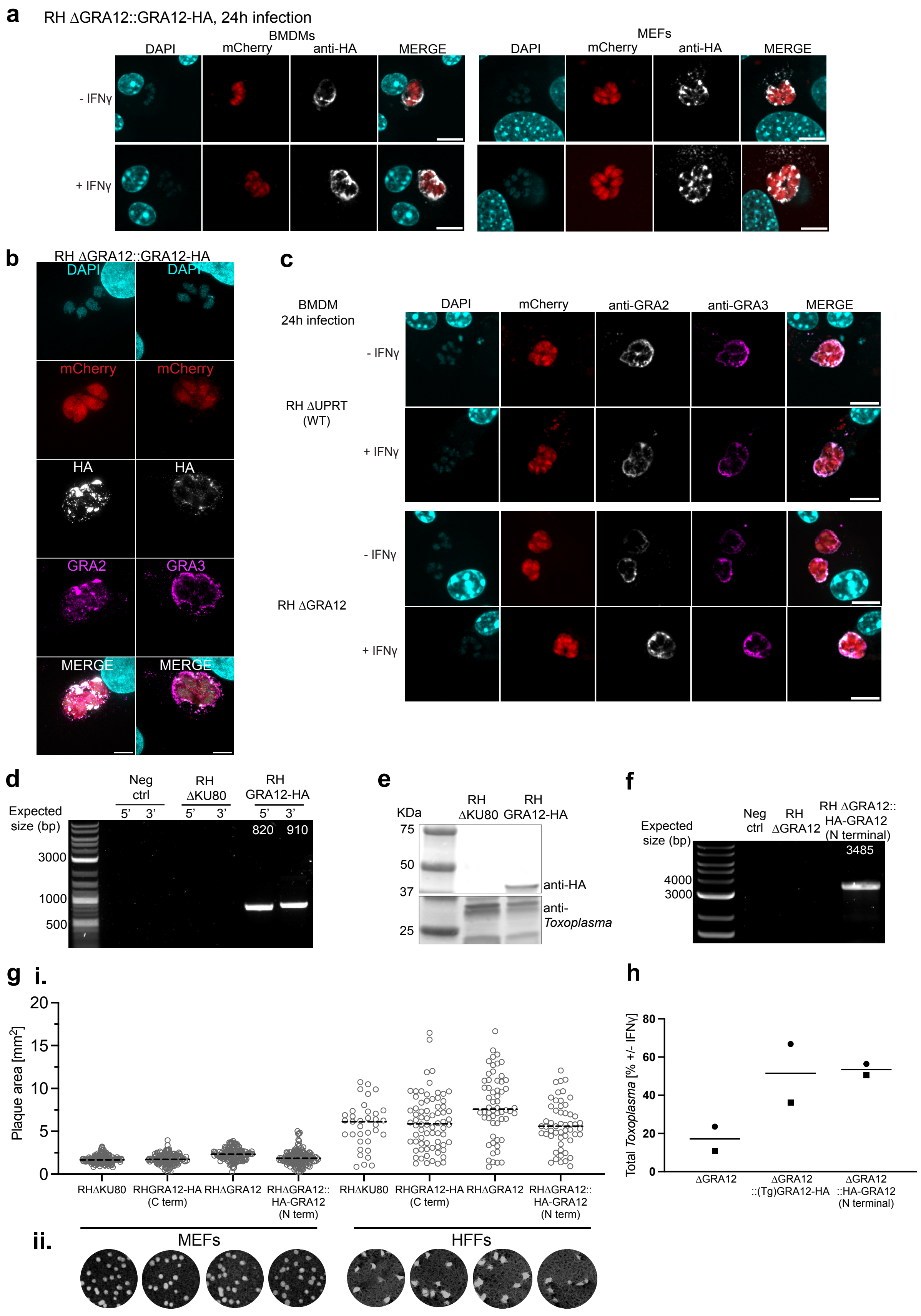
a
RH ΔGRA12::GRA12-HA, 24h infection
BMDMs
MEFs
DAPI
mCherry
anti-HA
MERGE
- IFNγ
+ IFNγ
b
RH ΔGRA12::GRA12-HA
DAPI
mCherry
HA
GRA2
GRA3
MERGE
c
BMDM
24h infection
DAPI
mCherry
anti-GRA2
anti-GRA3
MERGE
RH ΔUPRT
(WT)
RH ΔGRA12
d
Expected
size (bp)
Neg
ctrl
RH
ΔKU80
RH
GRA12-HA
5'
3'
820
910
3000
1000
500
e
KDa
RH
ΔKU80
RH
GRA12-HA
75
50
37
25
anti-HA
anti-
Toxoplasma
f
Expected
size (bp)
Neg
ctrl
RH
ΔGRA12
RH ΔGRA12::
HA-GRA12
(N terminal)
3485
4000
3000
g i.
Plaque area [mm²]
20
15
10
5
0
RHΔKU80
RHGRA12-HA
(C term)
RHΔGRA12
RHΔGRA12::
HA-GRA12
(N term)
ii.
MEFs
HFFs
h
Total Toxoplasma [% +/- IFNγ]
80
60
40
20
0
ΔGRA12
ΔGRA12
::(Tg)GRA12-HA
ΔGRA12
::HA-GRA12
(N terminal)

Supplementary Fig. 6. Localisation of GRA12 and creation of the N and C terminally HA-tagged GRA12 strains. a Immunofluorescence verification of the C-terminal HA-tagged GRA12 in BMDMs and MEFs, pretreated or not with 100 U/ml IFNγ. Scale bar is 10 µm. **b** Immunofluorescence localisation of GRA2 (left panel) and GRA3 (right panel) in relation to GRA12 in the RH ΔGRA12::GRA12-HA line infecting HFFs. Scale bar represents 5 µm. **c** Immunofluorescence verification of the localisation of the GRA2 and GRA3 in the RH ΔUPRT and RH ΔGRA12 strains in BMDMs pretreated or not with 100 U/ml IFNγ. Scale bar is 10 µm. **d** PCR validation of the RH GRA12 C-terminally HA-tagged line in the endogenous locus (RH GRA12-HA) by PCR and **e** by western blot. **f** PCR validation of the RH GRA12 N-terminally HA-tagged line (RH ΔGRA12::HA-GRA12) created by complementing GRA12 in the *Uprt* locus. **g** Plaque size of RH GRA12-HA and RH ΔGRA12::HA-GRA12 lines compared to their respective parental RH ΔKU80 and RH ΔGRA12 strains in MEFs and HFFs, N=1 (**i**) and respective images (**ii**). Data from control RH ΔKU80 and RH ΔGRA12 strains are the same as in Supplementary Fig. 3d. **h** Relative *Toxoplasma* growth in IFNγ-treated versus untreated BMDMs. BMDMs were infected with the RH ΔGRA12 strain, or the RHΔGRA12 strain complemented with *Toxoplasma* (Tg) GRA12 or the N-terminally HA-tagged line RH ΔGRA12::HA-GRA12, or the parental RH ΔGRA12 strain as control for 24h before a plate reader quantification of the mCherry signal as proxy for parasite growth. N=2. Source data are provided as a Source Data file.

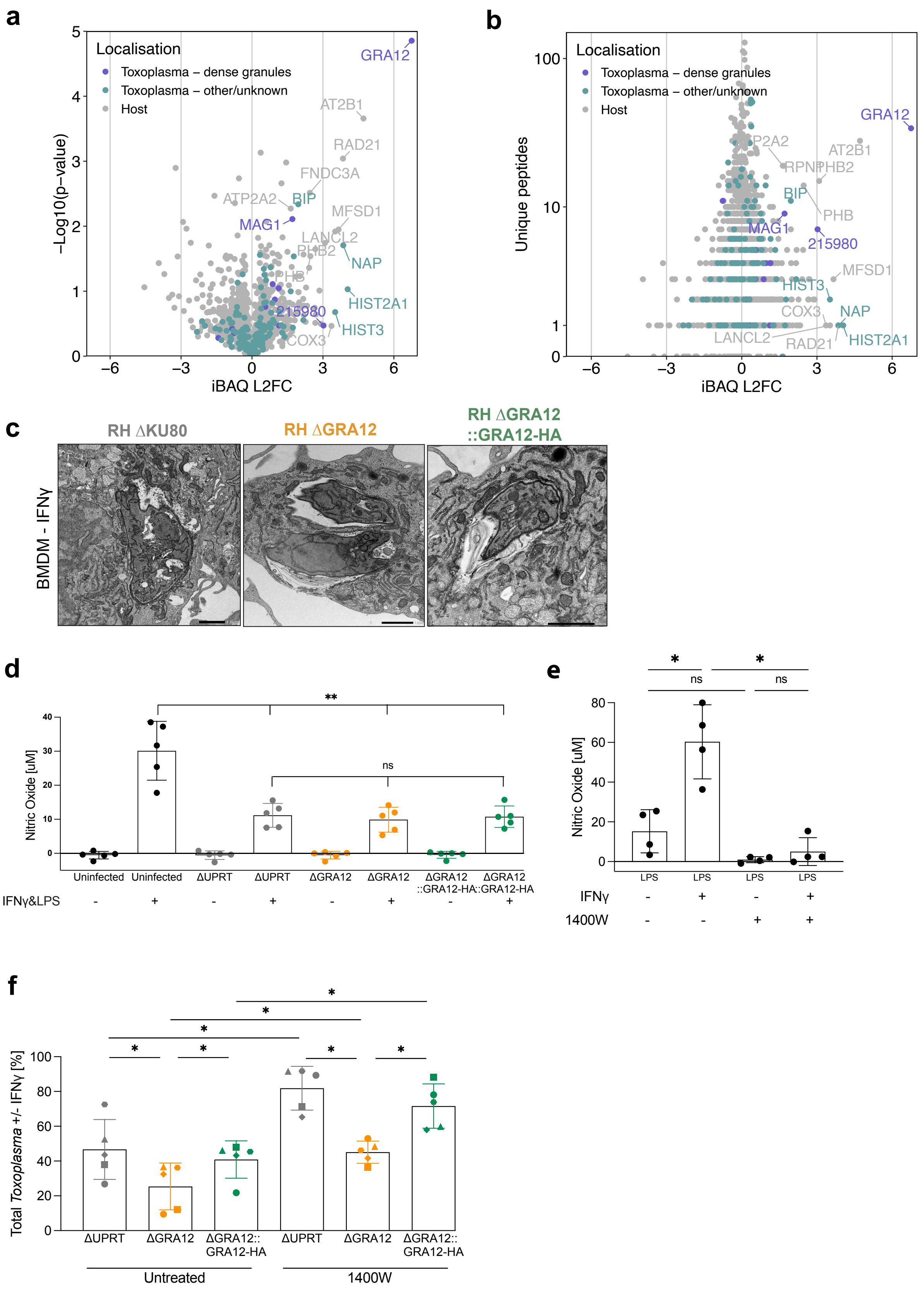
a
Localisation
Toxoplasma – dense granules
Toxoplasma – other/unknown
Host
GRA12
AT2B1
RAD21
FNDC3A
ATP2A2
BIP
MFSD1
MAG1
LANCL2
PHB2
NAP
PHB
HIST2A1
215980
HIST3
COX3
−Log10(p−value)
iBAQ L2FC
b
Localisation
Toxoplasma – dense granules
Toxoplasma – other/unknown
Host
GRA12
AT2B1
PHB2
BIP
PHB
MAG1
215980
MFSD1
HIST3
COX3
NAP
LANCL2
RAD21
HIST2A1
Unique peptides
iBAQ L2FC
c
RH ΔKU80
RH ΔGRA12
RH ΔGRA12 ::GRA12-HA
BMDM - IFNγ
d
Nitric Oxide [uM]
Uninfected
Uninfected
ΔUPRT
ΔUPRT
ΔGRA12
ΔGRA12
ΔGRA12 ::GRA12-HA
ΔGRA12 ::GRA12-HA
IFNγ&LPS
**
ns
e
Nitric Oxide [uM]
LPS
IFNγ
1400W
ns
f
Total Toxoplasma +/- IFNγ [%]
ΔUPRT
ΔGRA12
ΔGRA12:: GRA12-HA
Untreated
1400W

Supplementary Fig. 7. a Volcano plot of proteins identified by mass spectrometry in the anti-HA pull down in lysates from IFNγ-treated BMDM infected with either RH GRA12-HA or the parental RH ΔKU80 strains. The relative abundance of the proteins (iBAQ L2FC) is plotted against the p-value (-Log10(p-value), (**a**)) and against the number of peptides detected ((Unique peptides), (**b**)). **c** Example TEM images of untreated PWD/Phj BMDMs infected with RH ΔKU80, ΔGRA12, ΔGRA12::GRA12-HA for 2 h. **d** Quantification of Nitric Oxide from C57BL/6J BMDMs stimulated for 24 h with 100 U/ml IFNγ and 0.2 µg/ml LPS or left untreated as control, and infected for 24 h with RH ΔUPRT, ΔGRA12, ΔGRA12::GRA12-HA or left uninfected. Significance was tested using the One-way Anova test with the Benjamini, Krieger and Yekutieli FDR correction, **e** Quantification of Nitric Oxide from a culture of uninfected C57BL/6J BMDMs pretreated for 24 h with 100 U/ml IFNγ and with the iNOS inhibitor 1400W, or left untreated as control, and stimulated for 24 h with 0.2 µg/ml LPS. Significance was tested using the One-way Anova test with the Benjamini, Krieger and Yekutieli FDR correction, **f** Quantification by imaging of *Toxoplasma* restriction in IFNγ-treated or untreated C57BL/6 BMDMs, pretreated of not with 1400W, infected with RH ΔUPRT, ΔGRA12, ΔGRA12::GRA12-HA and parasites were quantified at 24 h after infection. Symbol shapes indicate biological repeats. p *<0.05, ** < 0.01. Source data are provided as a Source Data file.

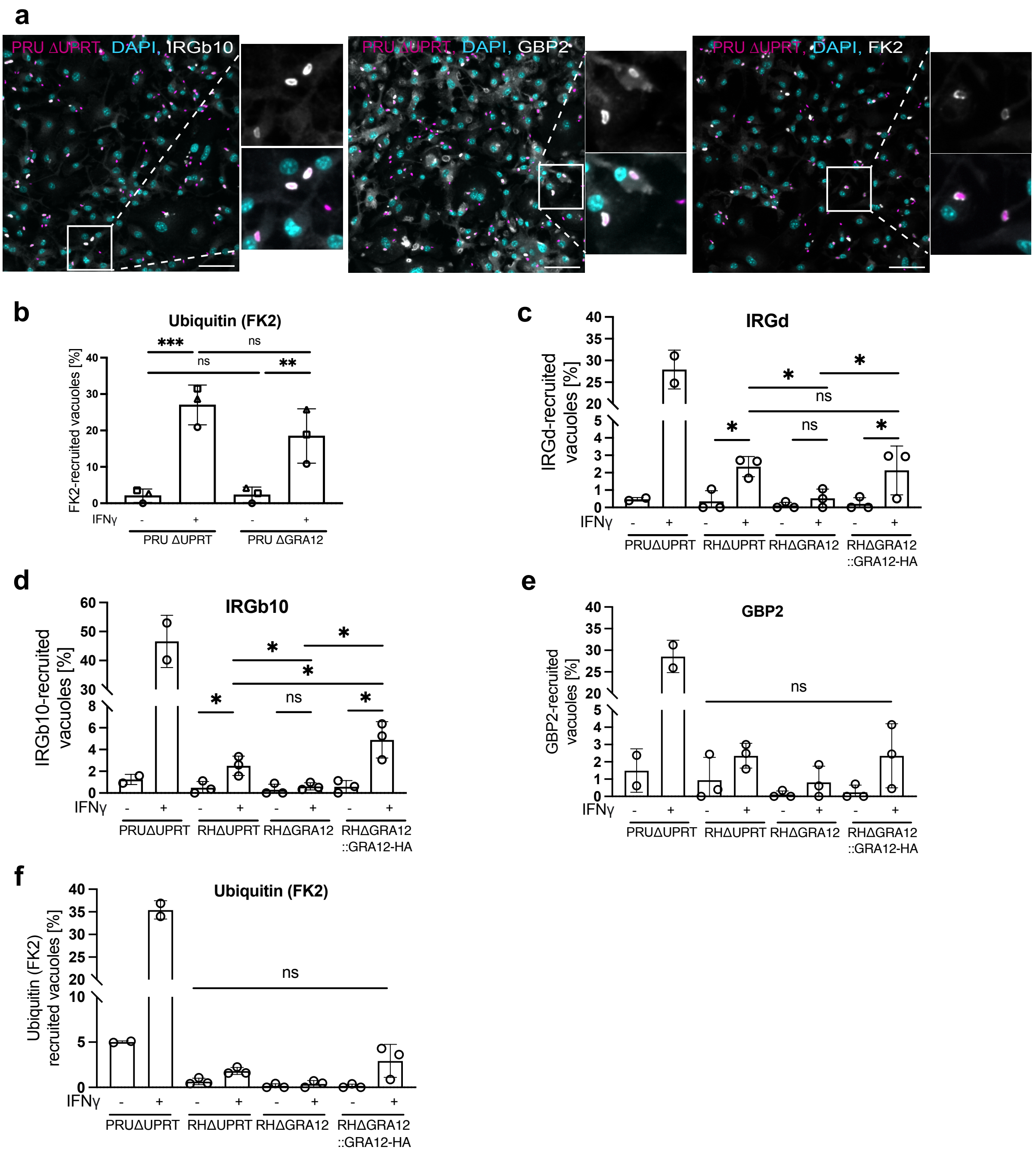

Supplementary Fig. 8. ΔGRA12 vacuoles are less recognised by host immune factors. a Representative images of anti-IRGb10, anti-GBP2 and anti-FK2 stained infections with PRU ΔUPRT. Scale bar represents 50 μm. **b** Quantification of the recruitment to the PVM of Ubiquitin (FK2) after 90 minutes infection of IFNγ-treated or untreated C57BL/6J BMDMs with PRU ΔUPRT or ΔGRA12 parasites, N=3. **c** Quantification of the recruitment to the PVM of IRGd (**c**), IRGb10 (**d**), GBP2 (**e**) and FK2 (**f**) after 90 minutes infection of IFNγ-treated or untreated C57BL/6J BMDMs with RH ΔUPRT, RH ΔGRA12 or RH ΔGRA12::GRA12-HA parasites, N=3. PRU ΔUPRT was used as positive control, N=2. Significance was tested using a One-way Anova test with the Benjamini, Krieger and Yekutieli FDR correction. p * <0.05, p ** <0.01, *** <0.001, **** <0.0001. Source data are provided as a Source Data file.

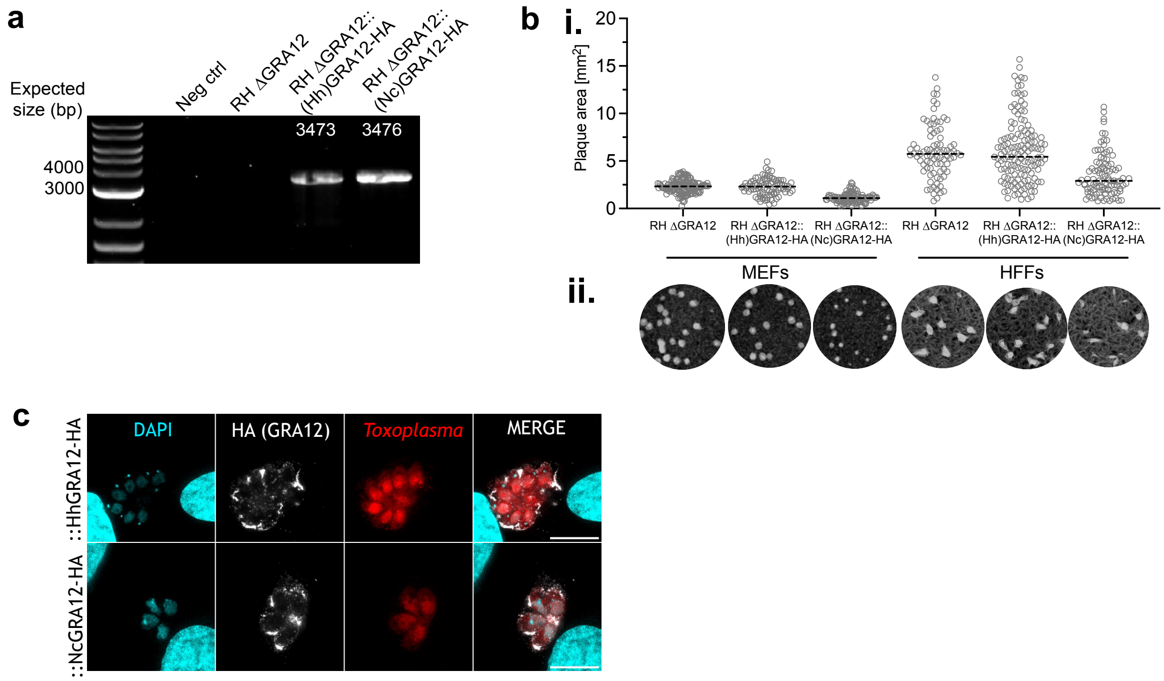

Supplementary Fig. 9. Creation of the *H. hammondi* and *N. caninum* GRA12 paralogs-complemented strains. a PCR validation of the RH ΔGRA12 strain complemented with the *H. hammondi* (::(Hh)GRA12-HA) or the *N. caninum* (::(Nc)GRA12-HA) GRA12 homologues. **b** Plaque size of the complemented ::(Hh)GRA12-HA and ::(Nc)GRA12-HA strains, compared to the parental RH ΔGRA12 strain in MEFs and HFFs, N=1 (**i**) and respective images (**ii**). Data from the control RH ΔGRA12 strain is the same as in Supplementary Fig. 3d. **c** Immunofluorescence localisation of GRA12 via anti-HA in the RH ΔGRA12::(Hh)GRA12-HA strain (upper panel) and RH ΔGRA12::(Nc)GRA12-HA strain (lower panel). Scale bar represents 10 µm. Source data are provided as a Source Data file.

TGVAND_312480 putative uracil phosphoribosyltransferase FUR1
KN044602:1,695,635-1,700,389

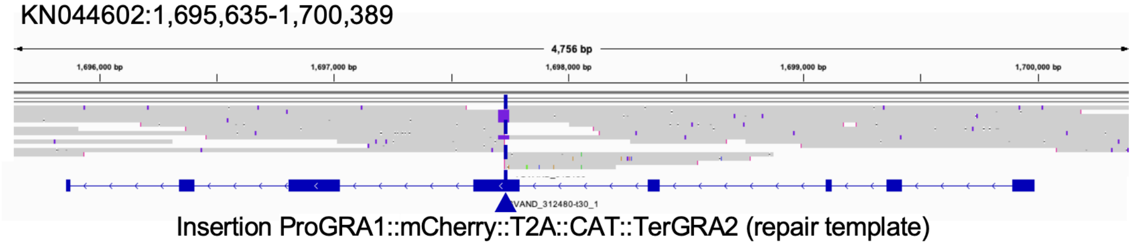

Insertion ProGRA1::mCherry::T2A::CAT::TerGRA2 (repair template)

TGVAND_288650 dense granule protein GRA12
KN044604:1,177,561-1,179,354

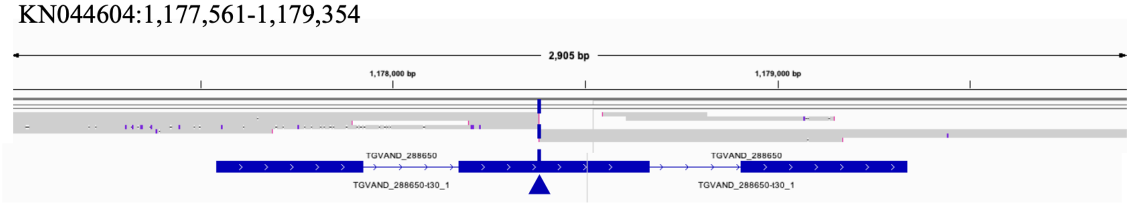

Insertion ProGRA1::mCherry::T2A::CAT::TerGRA2 (repair template)

TGVAND_205250 rhoptry protein ROP18
KN042496:360,725-362,389

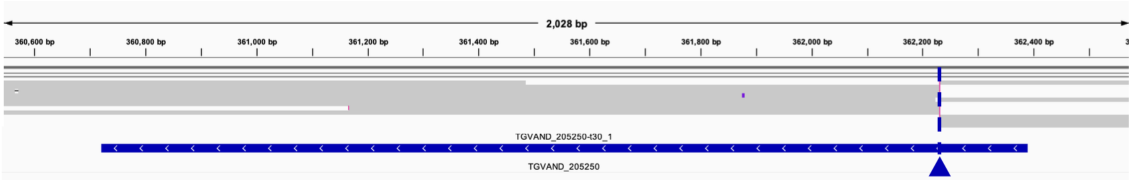

Insertion ProGRA1::mCherry::T2A::CAT::TerGRA2 (repair template)

Supplementary Fig. 10. Validation by Nanopore sequencing of the VAND mutant strains. Alignment of reads from Nanopore sequencing to the reference genome assembly ToxoDB-65_TgondiVAND. The alignment shows disruption of the endogenous UPRT (TGVAND_312480), GRA12 (TGVAND_205250) and ROP18 (TGVAND_205250) loci, for the creation of the VAND ΔUPRT, ΔGRA12 and ΔROP18 strains respectively. The arrowhead indicates the integration of the repair template ProGRA1::mCherry::T2A::CAT::TerGRA2 in the coding sequence.
