# Supplementary material for: GRA12 is a common virulence factor across Toxoplasma gondii strains and mouse subspecies
Source: Nat Commun. 2025 Apr 16;16:3570. doi: 10.1038/s41467-025-58876-2 (PMC12003902; doi:10.1038/s41467-025-58876-2)
Supplement: Supplementary file 2 — Description Of Additional Supplementary File [file 41467_2025_58876_MOESM2_ESM.pdf]

Description of Additional supplementary file

Supplementary Movie 1. Live cell brightfield time-lapse of C57BL/6J BMDMs infected with RHΔGRA12 parasites and treated with IFNγ. Time post infection reported as hh:mm in the bottom right corne

Supplementary Data 1. List of primers. A) List of primers and gDNA block used in the study

Supplementary Data 2. List of protospacers. A) List of protospacers included in the libraries, with their sequence and their targeted gene identity. B) Alignment of protospacers with 0 mismatches with the reference parasite genomes used in this study. C) Summary table of number of protospacers with 0 mismatches. D) Alignment of protospacers with 1 mismatch with the reference parasite genomes used in this study. E) Summary table of number of protospacers with 1 mismatch. F) Alignment of protospacers with 0-2 mismatches with the reference parasite genomes used in this study. G) Summary table of number of protospacers with 0-2 mismatches. H) 21 Alignment of protospacers with 0-3 mismatches with the reference parasite genomes used in this study. I) Summary table of number of protospacers with 0-3 mismatches. J) List of target genes with 3 or more mismatches with the relative protospacers.

Supplementary Data 3. VAND CRISPR knockout screen results. A) Raw protospacer sequencing read counts. B) Normalised protospacer sequencing read counts. C) Protospacer L2FCs in vivo and in vitro. D) Gene L2FCs in vivo, in vitro and their difference, p-values, L2FCs median absolute deviation (MAD) and DISCO scores.

Supplementary Data 4. RH CRISPR knockout screen results. A) Raw protospacer sequencing read counts. B) Normalised protospacer sequencing read counts. C) Protospacer L2FCs in vivo and in vitro. D) Gene L2FCs in vivo, in vitro and their difference, p-values, L2FCs median absolute deviation (MAD) and DISCO scores.

Supplementary Data 5. PRU CRISPR knockout screen results. A) Raw protospacer sequencing read counts. B) Normalised protospacer sequencing read counts. C) Protospacer L2FCs in vivo and in vitro. D) Gene L2FCs in vivo, in vitro and their difference, p-values, L2FCs median absolute deviation (MAD) and DISCO scores.

Supplementary Data 6. VEG CRISPR knockout screen results. A) Raw protospacer sequencing read counts. B) Normalised protospacer sequencing read counts. C) Protospacer L2FCs in vivo and in vitro. D) Gene L2FCs in vivo, in vitro and their difference, p-values, L2FCs median absolute deviation (MAD) and DISCO scores.

Supplementary Data 7. Gene L2FCs and their difference in all screens. A) Gene L2FCs in vivo, in vitro and their difference, p-values, L2FCs median absolute deviation (MAD) and DISCO scores. Genes are ranked for each screen based on the L2FC difference in vivo vs in vitro. B) Top ranking genes shared between the VAND, RH and PRU screens.

Supplementary Data 8. RH CRISPR knockout screen results in BMDMs. A) Raw protospacer sequencing read counts. B) Normalised protospacer sequencing read counts. C) Protospacer L2FCs in IFNγ-treated versus untreated BMDMs. D) Gene 57 L2FCs in IFNγ-treated BMDMs, in untreated BMDMs and their difference, p-values, 58 L2FCs median absolute deviation (MAD) and DISCO scores.

Supplementary Data 9. GRA12 co-immunoprecipitation mass spectrometry data. A) Peptide abundance of proteins pulled down with anti-HA agarose beads in PWD/PhJ BMDMs infected for 24h with RH GRA12-HA or RH ΔKU80 as control.
