# Supplementary material for: GRA12 is a common virulence factor across Toxoplasma gondii strains and mouse subspecies
Source: Nat Commun. 2025 Apr 16;16:3570. doi: 10.1038/s41467-025-58876-2 (PMC12003902; doi:10.1038/s41467-025-58876-2)
Supplement: Supplementary file 13 — Reporting Summary [file 41467_2025_58876_MOESM13_ESM.pdf]

nature portfolio

Corresponding author(s): Moritz Treeck

Last updated by author(s): Mar 18, 2025

# Reporting Summary

Nature Portfolio wishes to improve the reproducibility of the work that we publish. This form provides structure for consistency and transparency in reporting. For further information on Nature Portfolio policies, see our Editorial Policies and the Editorial Policy Checklist.

## Statistics

For all statistical analyses, confirm that the following items are present in the figure legend, table legend, main text, or Methods section.

| n/a | Confirmed | |
|---|---|---|
| ☐ | ☒ | The exact sample size (*n*) for each experimental group/condition, given as a discrete number and unit of measurement |
| ☐ | ☒ | A statement on whether measurements were taken from distinct samples or whether the same sample was measured repeatedly |
| ☐ | ☒ | The statistical test(s) used AND whether they are one- or two-sided<br>*Only common tests should be described solely by name; describe more complex techniques in the Methods section.* |
| ☐ | ☒ | A description of all covariates tested |
| ☐ | ☒ | A description of any assumptions or corrections, such as tests of normality and adjustment for multiple comparisons |
| ☐ | ☒ | A full description of the statistical parameters including central tendency (e.g. means) or other basic estimates (e.g. regression coefficient) AND variation (e.g. standard deviation) or associated estimates of uncertainty (e.g. confidence intervals) |
| ☐ | ☒ | For null hypothesis testing, the test statistic (e.g. *F*, *t*, *r*) with confidence intervals, effect sizes, degrees of freedom and *P* value noted<br>*Give P values as exact values whenever suitable.* |
| ☒ | ☐ | For Bayesian analysis, information on the choice of priors and Markov chain Monte Carlo settings |
| ☒ | ☐ | For hierarchical and complex designs, identification of the appropriate level for tests and full reporting of outcomes |
| ☒ | ☐ | Estimates of effect sizes (e.g. Cohen's *d*, Pearson's *r*), indicating how they were calculated |

*Our web collection on statistics for biologists contains articles on many of the points above.*

## Software and code

Policy information about availability of computer code

| | |
|---|---|
| Data collection | Microsoft Excel 16.94, GraphPad Prism v10. |
| Data analysis | R 4.2.1, RStudio 2022.07.02, GraphPad Prism v10. Code used for analysis is deposited in Github as described in the manuscript. |

For manuscripts utilizing custom algorithms or software that are central to the research but not yet described in published literature, software must be made available to editors and reviewers. We strongly encourage code deposition in a community repository (e.g. GitHub). See the Nature Portfolio guidelines for submitting code & software for further information.

## Data

Policy information about availability of data

All manuscripts must include a data availability statement. This statement should provide the following information, where applicable:

- Accession codes, unique identifiers, or web links for publicly available datasets
- A description of any restrictions on data availability
- For clinical datasets or third party data, please ensure that the statement adheres to our policy

All data are available and provided with the manuscript. The code has been deposited in the following repository doi:10.5281/zenodo.15040348

## Research involving human participants, their data, or biological material

Policy information about studies with human participants or human data. See also policy information about sex, gender (identity/presentation), and sexual orientation and race, ethnicity and racism.

| | |
|---|---|
| Reporting on sex and gender | *Use the terms sex (biological attribute) and gender (shaped by social and cultural circumstances) carefully in order to avoid confusing both terms. Indicate if findings apply to only one sex or gender; describe whether sex and gender were considered in study design; whether sex and/or gender was determined based on self-reporting or assigned and methods used. Provide in the source data disaggregated sex and gender data, where this information has been collected, and if consent has been obtained for sharing of individual-level data; provide overall numbers in this Reporting Summary. Please state if this information has not been collected. Report sex- and gender-based analyses where performed, justify reasons for lack of sex- and gender-based analysis.* |
| Reporting on race, ethnicity, or other socially relevant groupings | *Please specify the socially constructed or socially relevant categorization variable(s) used in your manuscript and explain why they were used. Please note that such variables should not be used as proxies for other socially constructed/relevant variables (for example, race or ethnicity should not be used as a proxy for socioeconomic status). Provide clear definitions of the relevant terms used, how they were provided (by the participants/respondents, the researchers, or third parties), and the method(s) used to classify people into the different categories (e.g. self-report, census or administrative data, social media data, etc.) Please provide details about how you controlled for confounding variables in your analyses.* |
| Population characteristics | *Describe the covariate-relevant population characteristics of the human research participants (e.g. age, genotypic information, past and current diagnosis and treatment categories). If you filled out the behavioural & social sciences study design questions and have nothing to add here, write "See above."* |
| Recruitment | *Describe how participants were recruited. Outline any potential self-selection bias or other biases that may be present and how these are likely to impact results.* |
| Ethics oversight | *Identify the organization(s) that approved the study protocol.* |

Note that full information on the approval of the study protocol must also be provided in the manuscript.

# Field-specific reporting

Please select the one below that is the best fit for your research. If you are not sure, read the appropriate sections before making your selection.

☒ Life sciences ☐ Behavioural & social sciences ☐ Ecological, evolutionary & environmental sciences

For a reference copy of the document with all sections, see nature.com/documents/nr-reporting-summary-flat.pdf

# Life sciences study design

All studies must disclose on these points even when the disclosure is negative.

| | |
|---|---|
| Sample size | Sample size for the CRISPR screens in vivo was established in previous work from the group (Young et al 2019). |
| Data exclusions | No data were excluded. |
| Replication | all experiments where performed in a biological replicate of 3, instead otherwise mentioned. |
| Randomization | Animals were randomly selected within a group with similar age and identical genetic background. Microscopy images, both from transmission electron microscopy and immunofluorescence, were acquired from random positions of each slide. Randomization in other experiments was not applied. |
| Blinding | Blinding was performed for TEM image analysis (3 different lab members scored the TEM images independently, now knowing what conditions each image originated from). IFA on subcellular distribution of GRA12 in different strains and cell types were blinded. Images where taken by one person, and analysed by another person without knowing which conditions they originated from. |

# Reporting for specific materials, systems and methods

We require information from authors about some types of materials, experimental systems and methods used in many studies. Here, indicate whether each material, system or method listed is relevant to your study. If you are not sure if a list item applies to your research, read the appropriate section before selecting a response.

## Materials & experimental systems

| n/a | Involved in the study | |
|---|---|---|
| ☐ | ☒ | Antibodies |
| ☐ | ☒ | Eukaryotic cell lines |
| ☒ | ☐ | Palaeontology and archaeology |
| ☐ | ☒ | Animals and other organisms |
| ☒ | ☐ | Clinical data |
| ☒ | ☐ | Dual use research of concern |
| ☒ | ☐ | Plants |

## Methods

| n/a | Involved in the study | |
|---|---|---|
| ☒ | ☐ | ChIP-seq |
| ☒ | ☐ | Flow cytometry |
| ☒ | ☐ | MRI-based neuroimaging |

# Antibodies

Antibodies used

List of commercially available antibodies: rabbit anti-Toxoplasma (Abcam ab138698); rat anti-HA Horseradish Peroxidase conjugated (Roche 12013819001); rabbit anti-caspase 8 (Cell Signaling Technology 4927); rabbit anti-cleaved caspase 8 (Cell Signaling Technology 8592); rabbit anti-MLKL(phospho Ser345, Abcam ab196436); mouse anti-β actin (Sigma A2228); rabbit anti-HMGB1 (Abcam ab18256); goat anti-rabbit HRP (Insight Biotechnology 474-1506); goat anti-mouse HRP (Insight Biotechnologies, 474–1806); mouse anti-α tubulin (Abcam ab7291); rabbit anti-GBP2 (Proteintech 11854-1-AP); mouse anti-Ubiquitin (FK2, Merck ST1200); rabbit anti-p62 (Genetex GTX111393); mouse anti-IRGm1 (Sigma SAB1305514) ; mouse anti-IRGm3 (Santa Cruz sc-136317); anti-rat AlexaFluor 647 (Thermo Fisher A21247); anti-mouse AlexaFluor 647 (Thermo Fisher A21235); anti-rabbit AlexaFluor 647 (Thermo Fisher A21244); anti-rabbit AlexaFluor 488 (Thermo Fisher A11008); anti-mouse AlexaFluor 488 (Thermo Fisher A11029); anti-rat AlexaFluor 488 (Thermo Fisher A11006); anti-rabbit AlexaFluor 405 (Thermo Fisher A-31556).
List of homemade gifted antibodies: mouse anti-GRA2 and rabbit anti-GRA3 (from Jean François Dubremetz); rat anti-GRA12 (from Maryse Lebrun); rabbit anti-IRGd6 (081/3) and anti-IRGb10 (940/6, both from Jonathan Howard); rabbit anti-ROP18 (from David Sibley)

Validation

Gifted primary antibodies have been validated in previous work from the producing laboratories. Commercially available antibodies have not been further validated.

# Eukaryotic cell lines

Policy information about cell lines and Sex and Gender in Research

Cell line source(s)

Primary cell lines (HFFs and MEFs) were obtained from ATCC.

Authentication

None of the cell lines used were authenticated.

Mycoplasma contamination

All cell culture material (cell lines and Toxoplasma strains) were regularly tested for Mycoplasma contamination at the in house Cell Services facility at the Francis Crick Institute.

Commonly misidentified lines
(See ICLAC register)

*Name any commonly misidentified cell lines used in the study and provide a rationale for their use.*

# Animals and other research organisms

Policy information about studies involving animals; ARRIVE guidelines recommended for reporting animal research, and Sex and Gender in Research

Laboratory animals

All animals used were 6-16w old and of the following strains: Mus musculus domesticus C57BL/6J strain, M. m. castaneus CAST/Eij strain, M. m. musculus PWD/Phj strain and Wistar rats. All animals were house in specific pathogen-free conditions, at controlled humidity (~50%) and temperature (23C) conditions.

Wild animals

The study did not involve wild animals.

Reporting on sex

Animals of both sexes were used.

Field-collected samples

The study did not involve samples collected from the field.

Ethics oversight

All mouse work was approved by the UK Home Office (project licence P1A20E3F9) and the Francis Crick Institute Ethical Review Panel and carried out in accordance with the UK Animals (Scientific Procedure) Act 1986 and European Union directive 2010/63/EU.

Note that full information on the approval of the study protocol must also be provided in the manuscript.

## Plants

| | |
|---|---|
| Seed stocks | *Report on the source of all seed stocks or other plant material used. If applicable, state the seed stock centre and catalogue number. If plant specimens were collected from the field, describe the collection location, date and sampling procedures.* |
| Novel plant genotypes | *Describe the methods by which all novel plant genotypes were produced. This includes those generated by transgenic approaches, gene editing, chemical/radiation-based mutagenesis and hybridization. For transgenic lines, describe the transformation method, the number of independent lines analyzed and the generation upon which experiments were performed. For gene-edited lines, describe the editor used, the endogenous sequence targeted for editing, the targeting guide RNA sequence (if applicable) and how the editor was applied.* |
| Authentication | *Describe any authentication procedures for each seed stock used or novel genotype generated. Describe any experiments used to assess the effect of a mutation and, where applicable, how potential secondary effects (e.g. second site T-DNA insertions, mosiacism, off-target gene editing) were examined.* |
