# Supplementary figures and images for: GRA12 is a common virulence factor across Toxoplasma gondii strains and mouse subspecies (part 2 of 3)
Source: Nat Commun. 2025 Apr 16;16:3570. doi: 10.1038/s41467-025-58876-2 (PMC12003902; doi:10.1038/s41467-025-58876-2)

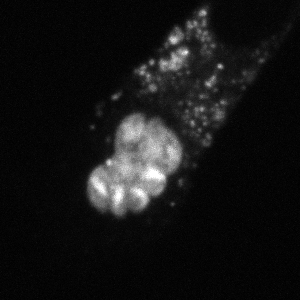

Supplement: Supplementary file 15 — Source data [file 41467_2025_58876_MOESM15_ESM.zip › Source suppl/Supplementary Figure 6_Source Data/Suppl Fig6c/BMDM_plus_UPRT_1_toxo_crop.tif]

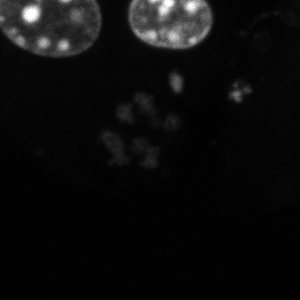

Supplement: Supplementary file 15 — Source data [file 41467_2025_58876_MOESM15_ESM.zip › Source suppl/Supplementary Figure 6_Source Data/Suppl Fig6c/BMDM_minus_UPRT_1_DAPI_crop.tif]

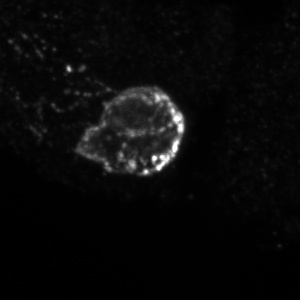

Supplement: Supplementary file 15 — Source data [file 41467_2025_58876_MOESM15_ESM.zip › Source suppl/Supplementary Figure 6_Source Data/Suppl Fig6c/BMDM_minus_UPRT_1_GRA3_crop.tif]

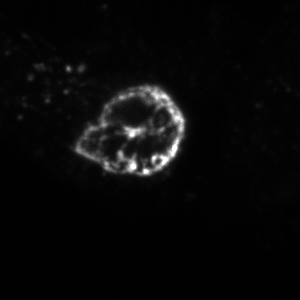

Supplement: Supplementary file 15 — Source data [file 41467_2025_58876_MOESM15_ESM.zip › Source suppl/Supplementary Figure 6_Source Data/Suppl Fig6c/BMDM_minus_UPRT_1_GRA2_crop.tif]

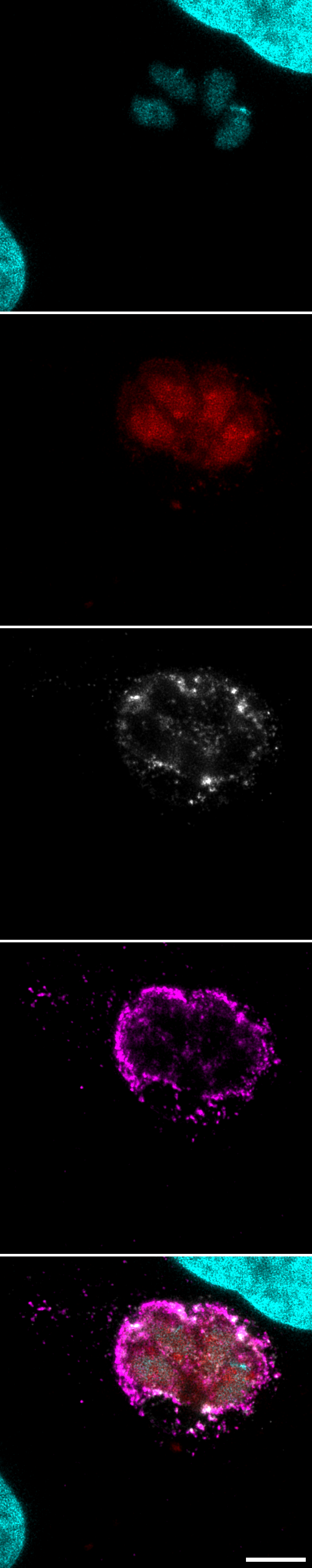

Supplement: Supplementary file 15 — Source data [file 41467_2025_58876_MOESM15_ESM.zip › Source suppl/Supplementary Figure 6_Source Data/Suppl Fig6b/COMPL_Nterm_GRA3_Montage.tif]

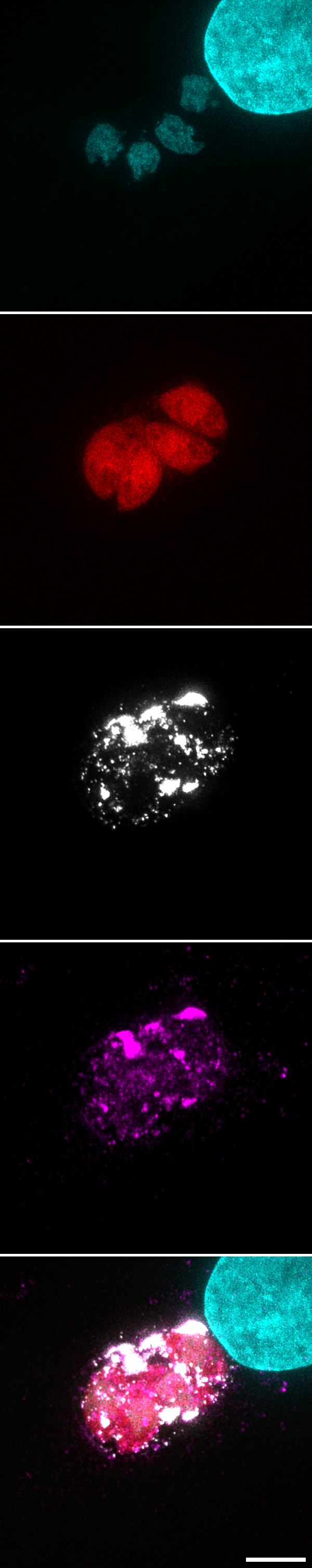

Supplement: Supplementary file 15 — Source data [file 41467_2025_58876_MOESM15_ESM.zip › Source suppl/Supplementary Figure 6_Source Data/Suppl Fig6b/COMPL_GRA2_Montage.tif]

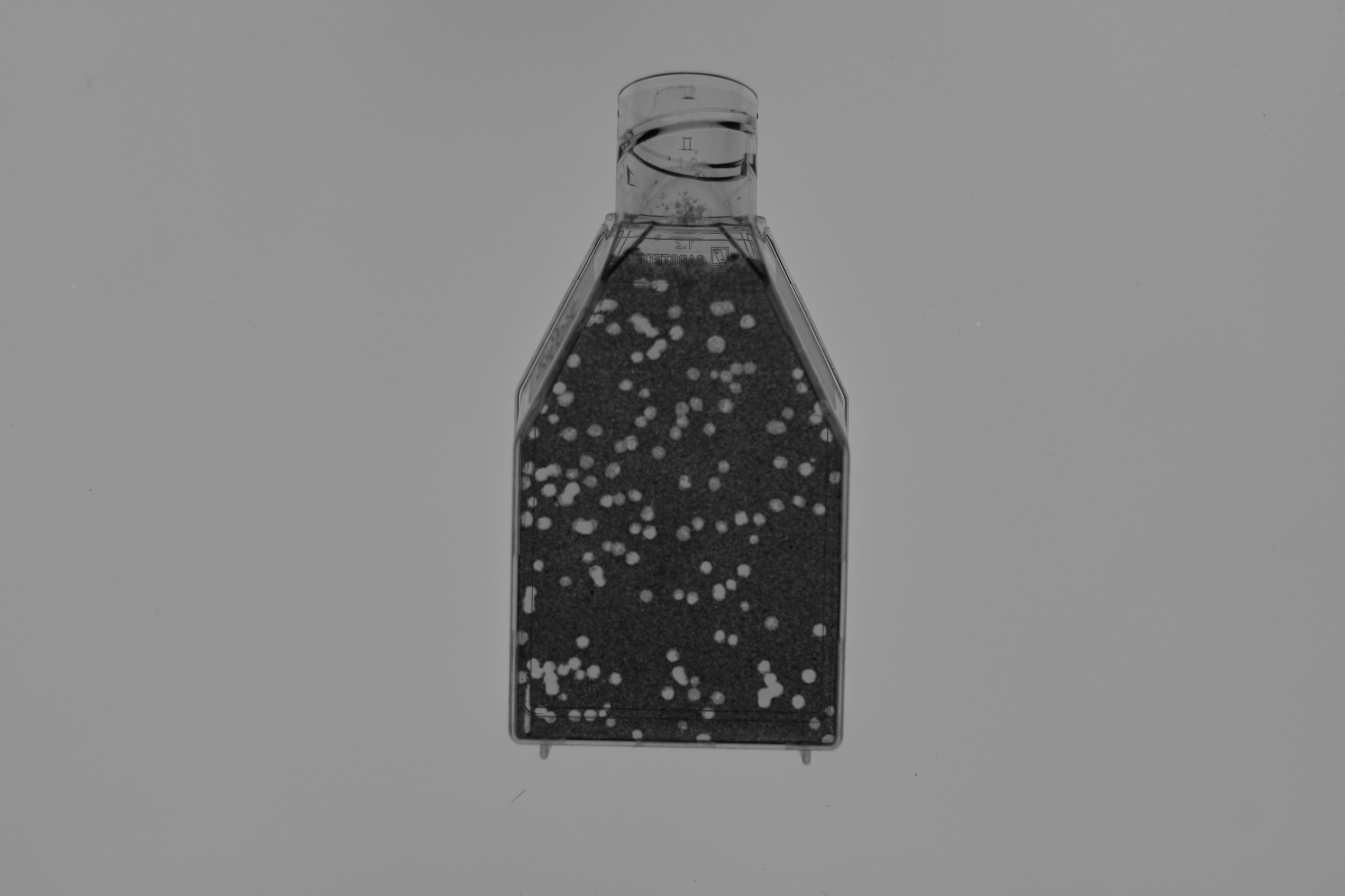

Supplement: Supplementary file 15 — Source data [file 41467_2025_58876_MOESM15_ESM.zip › Source suppl/Supplementary Figure 6_Source Data/Suppl Fig6g/igcuser 2024-12-16 16h57m55s(Silver Stain).raw16.tif]

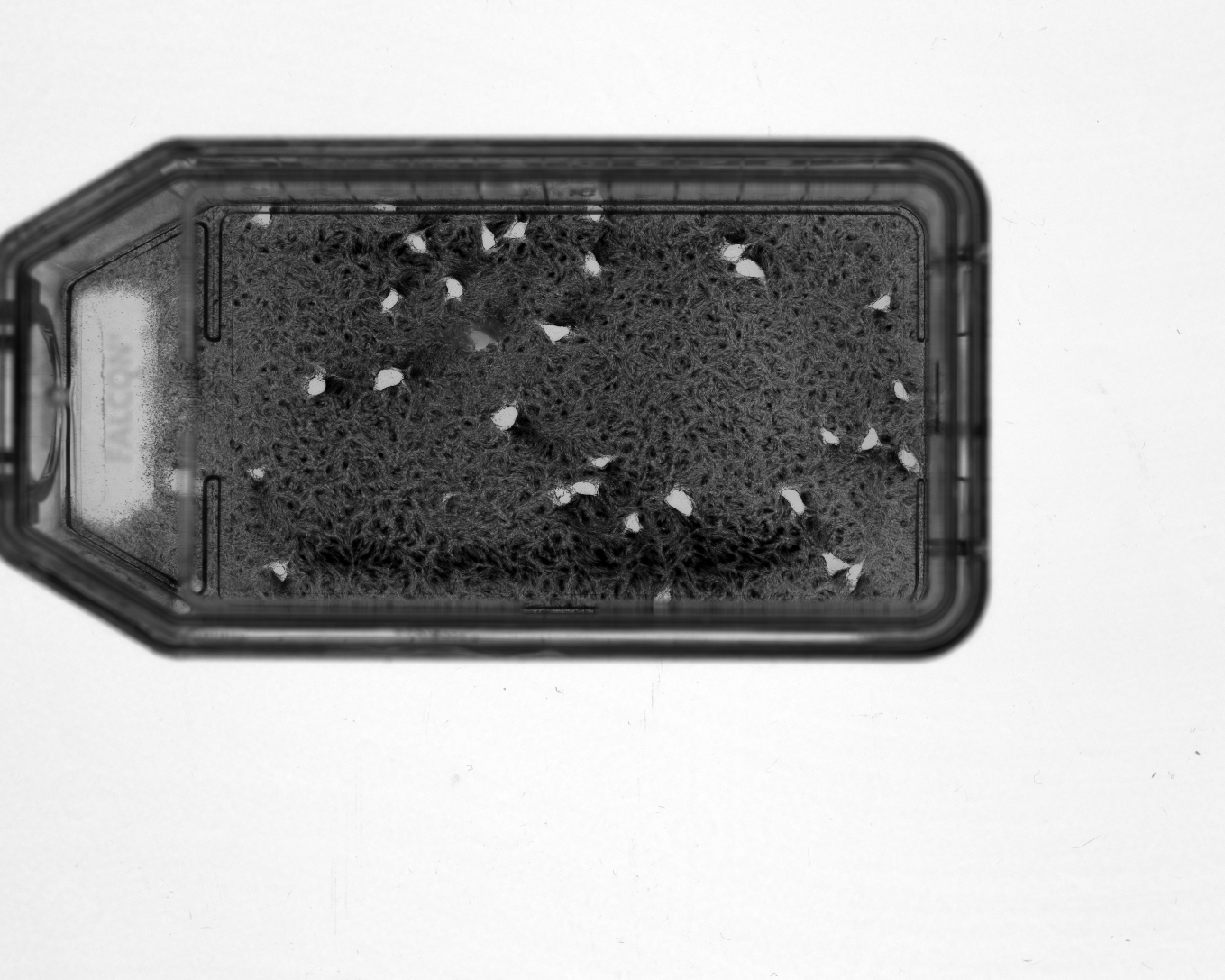

Supplement: Supplementary file 15 — Source data [file 41467_2025_58876_MOESM15_ESM.zip › Source suppl/Supplementary Figure 6_Source Data/Suppl Fig6g/new_rh_ku_100(Silver Stain).jpg]

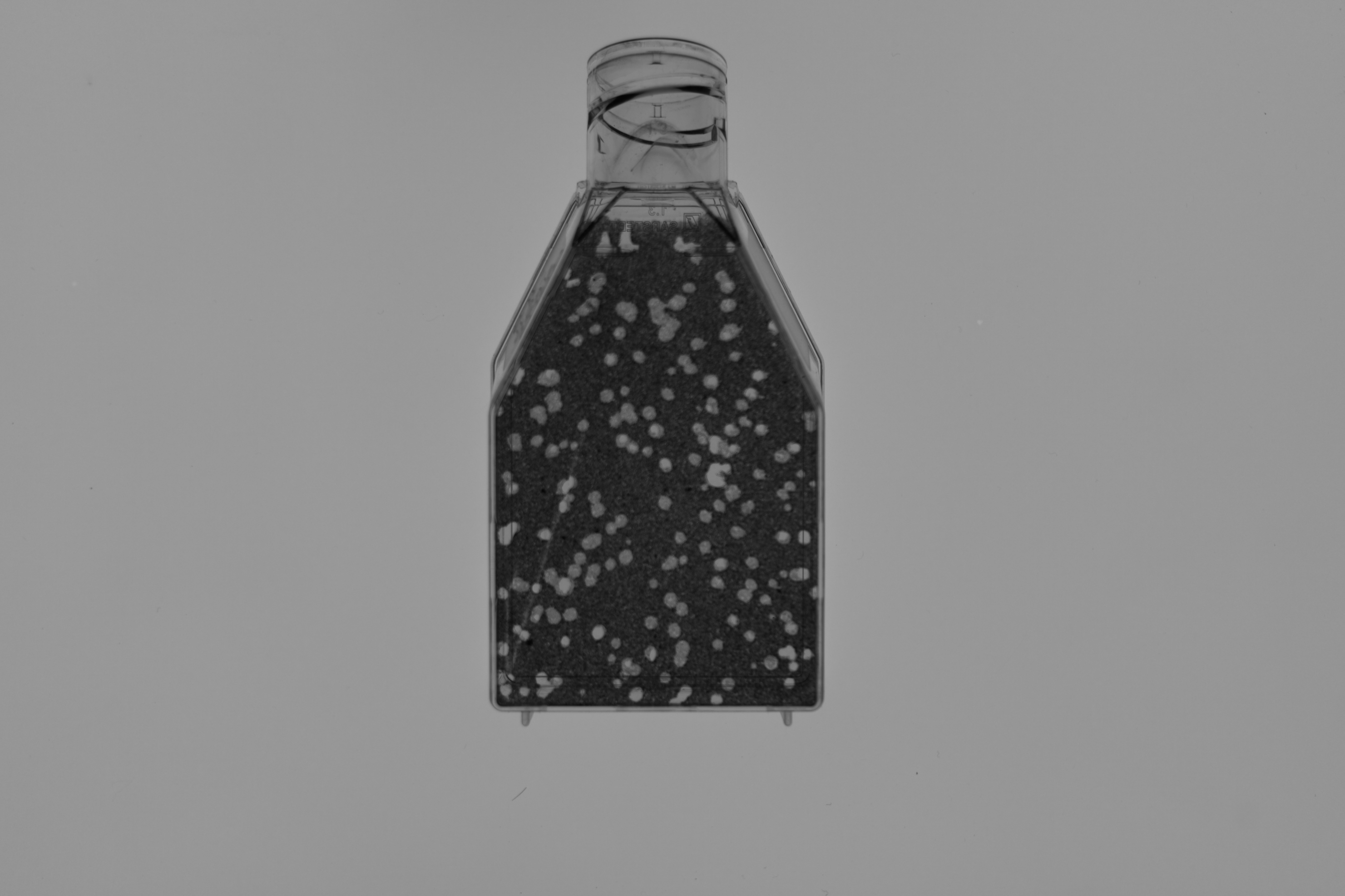

Supplement: Supplementary file 15 — Source data [file 41467_2025_58876_MOESM15_ESM.zip › Source suppl/Supplementary Figure 6_Source Data/Suppl Fig6g/igcuser 2024-12-16 16h59m16s(Silver Stain).raw16.tif]

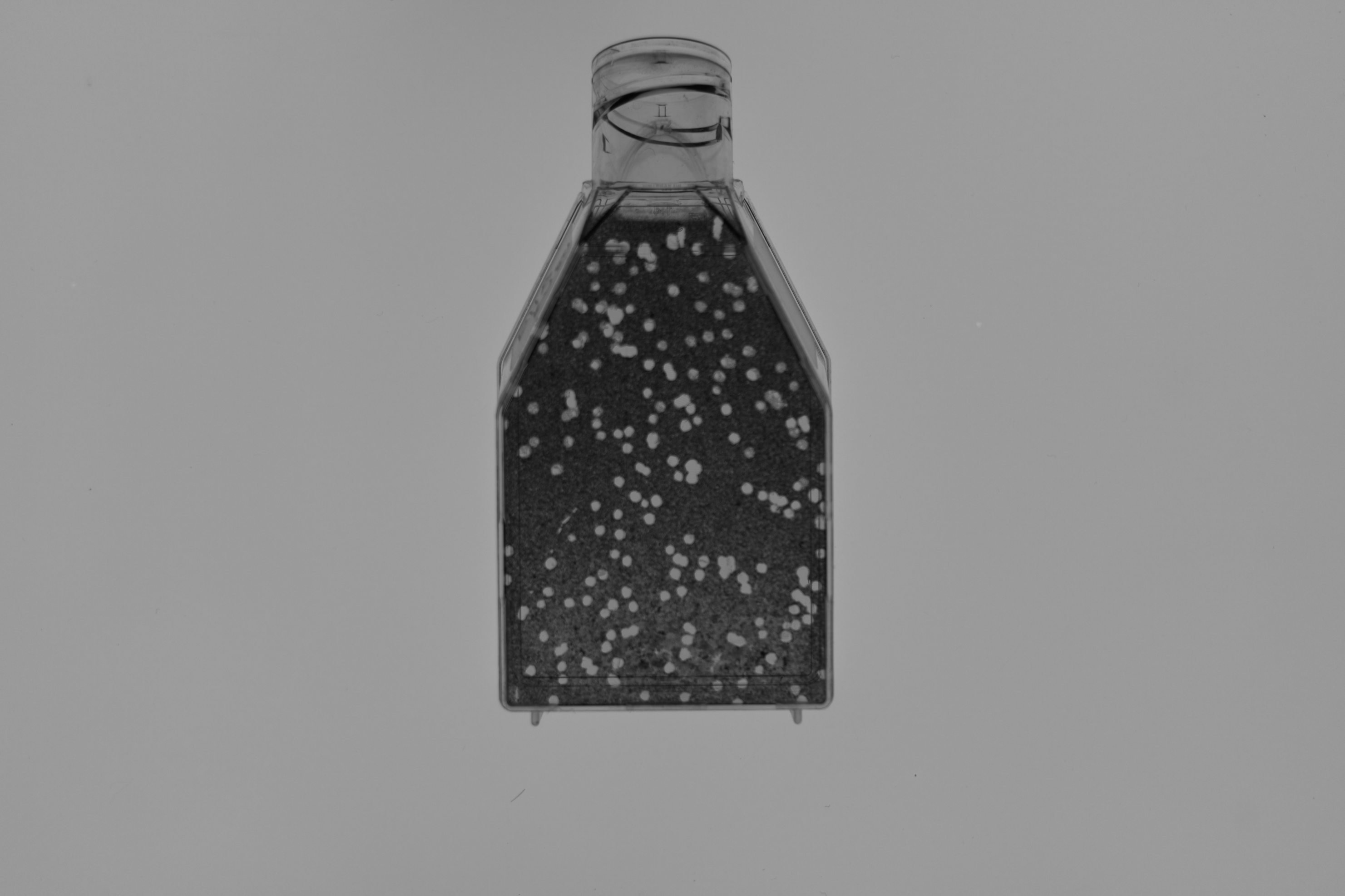

Supplement: Supplementary file 15 — Source data [file 41467_2025_58876_MOESM15_ESM.zip › Source suppl/Supplementary Figure 6_Source Data/Suppl Fig6g/igcuser 2024-12-16 16h54m42s(Silver Stain).raw16.tif]

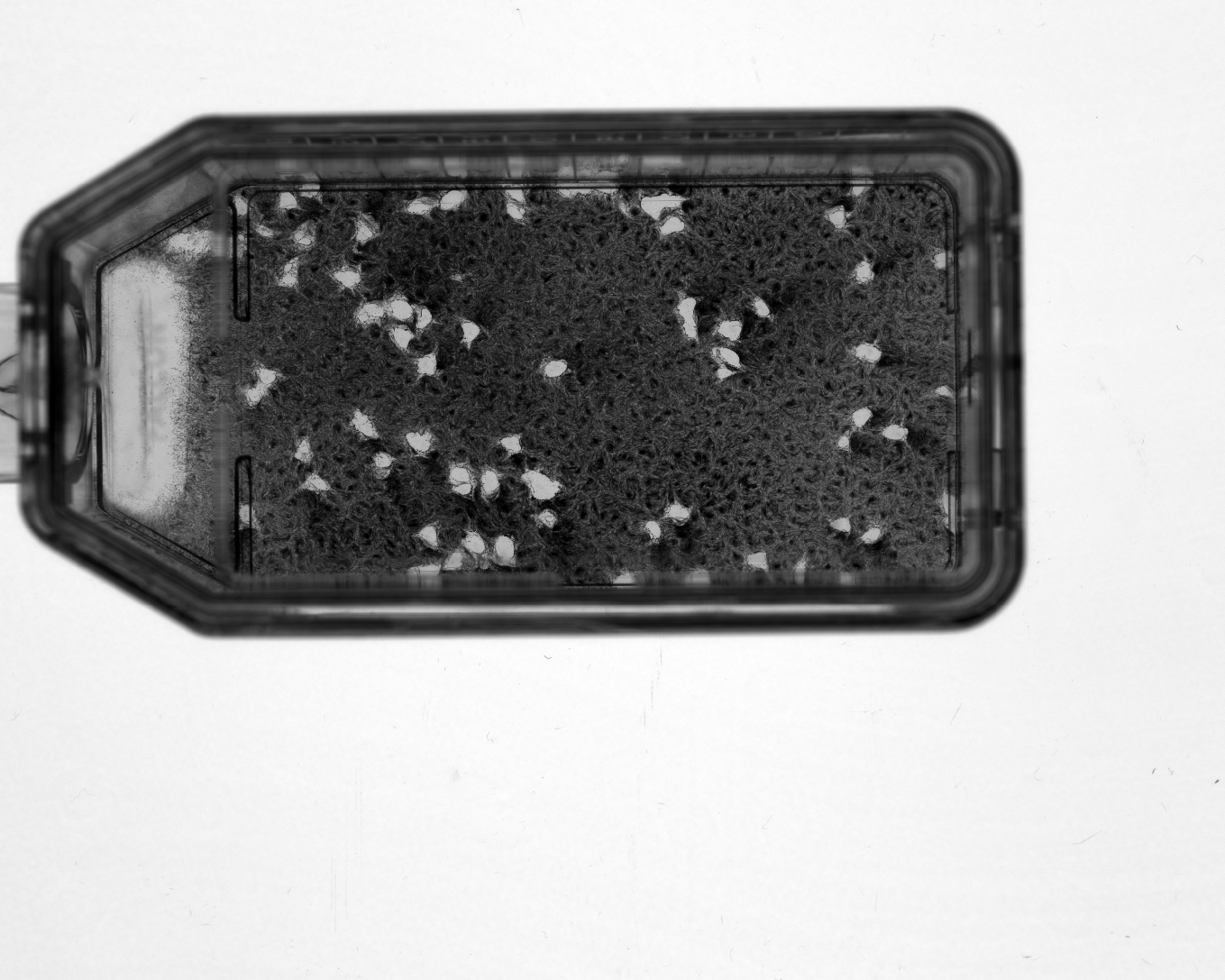

Supplement: Supplementary file 15 — Source data [file 41467_2025_58876_MOESM15_ESM.zip › Source suppl/Supplementary Figure 6_Source Data/Suppl Fig6g/new_gra12-ha(Silver Stain).jpg]

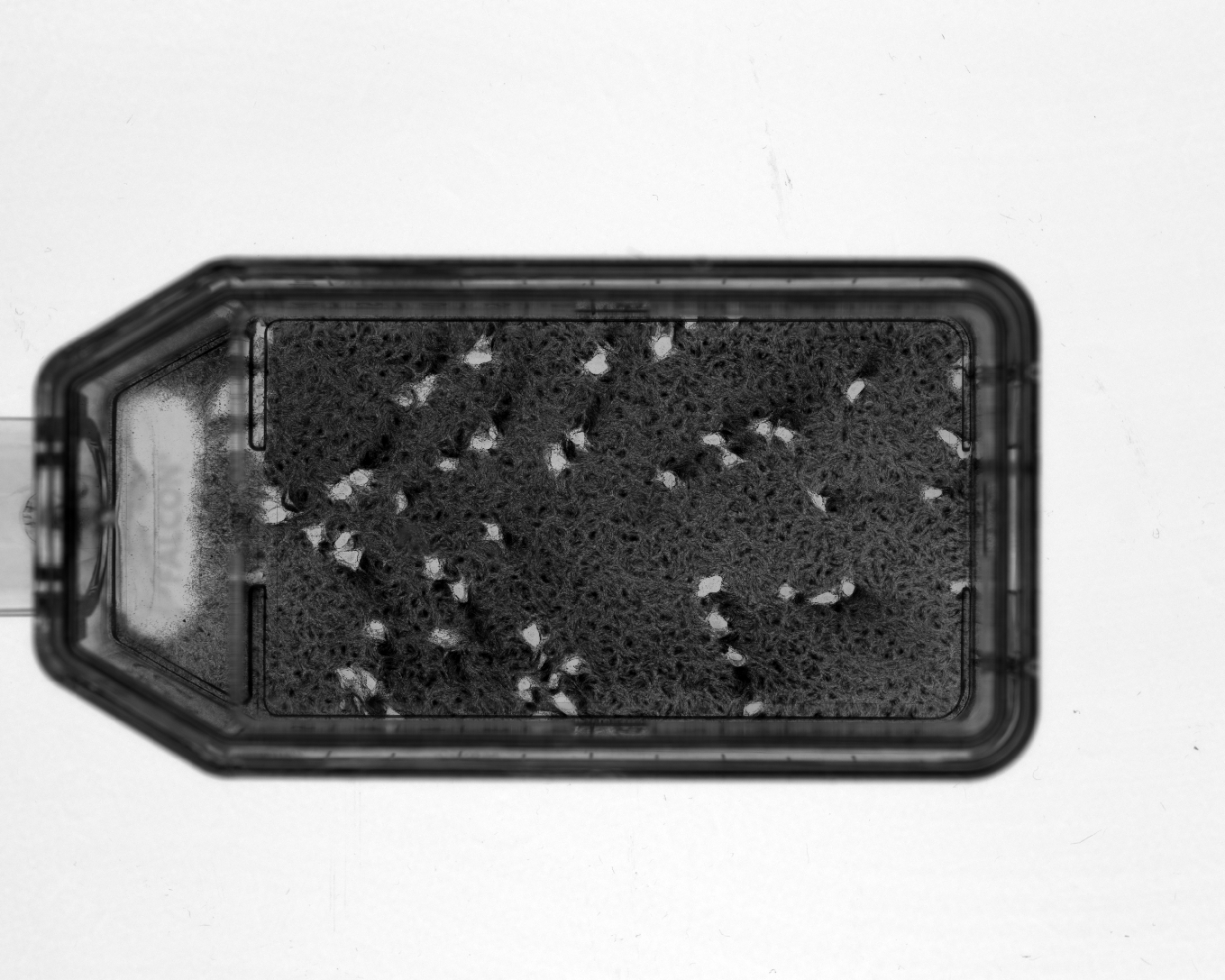

Supplement: Supplementary file 15 — Source data [file 41467_2025_58876_MOESM15_ESM.zip › Source suppl/Supplementary Figure 6_Source Data/Suppl Fig6g/new_nterm_100(Silver Stain).jpg]

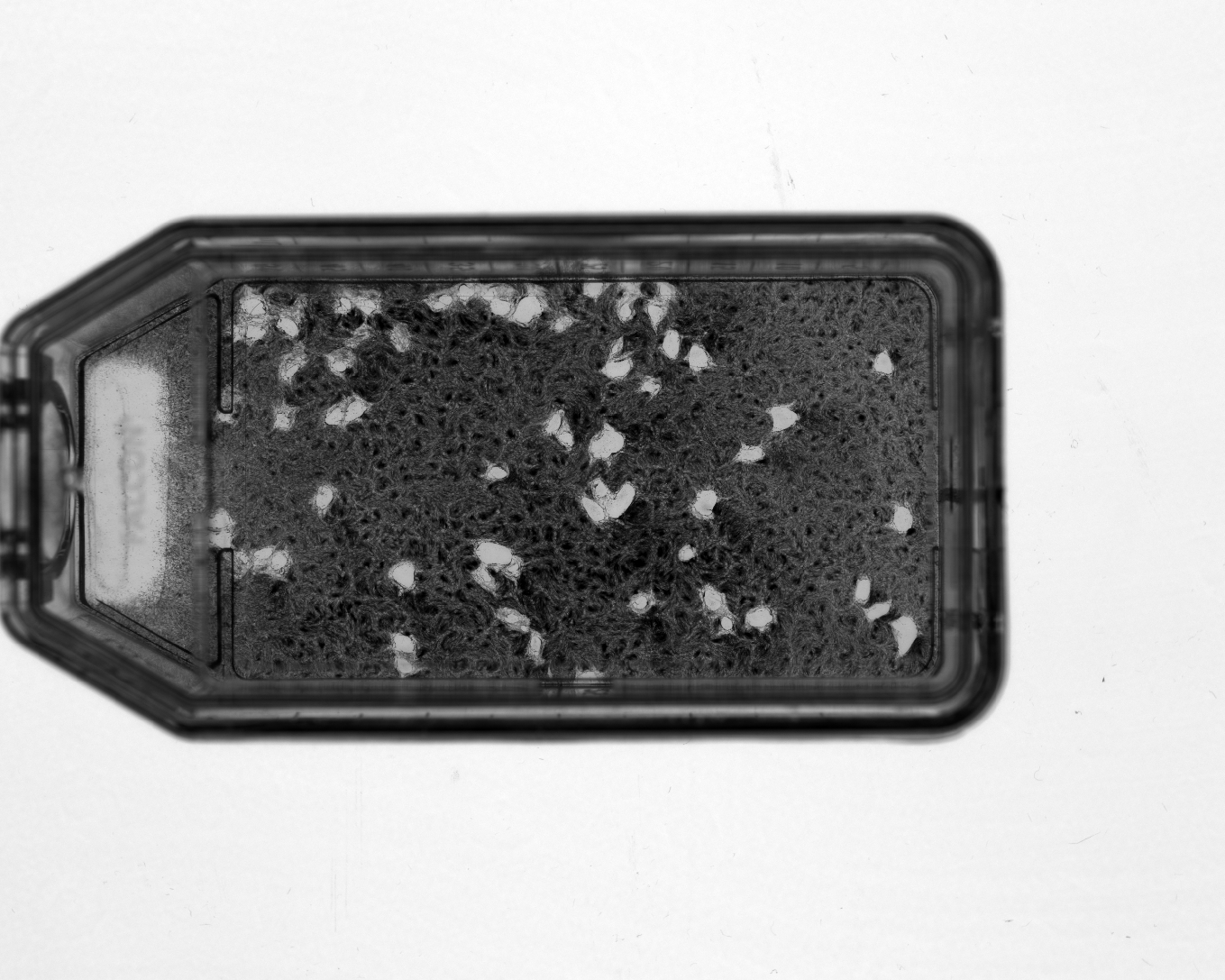

Supplement: Supplementary file 15 — Source data [file 41467_2025_58876_MOESM15_ESM.zip › Source suppl/Supplementary Figure 6_Source Data/Suppl Fig6g/new_gra_100(Silver Stain).jpg]

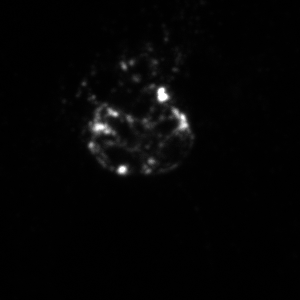

Supplement: Supplementary file 15 — Source data [file 41467_2025_58876_MOESM15_ESM.zip › Source suppl/Supplementary Figure 6_Source Data/Suppl Fig6a/MEF_COMPL_minus_3_HA.tif]

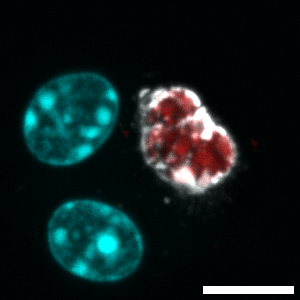

Supplement: Supplementary file 15 — Source data [file 41467_2025_58876_MOESM15_ESM.zip › Source suppl/Supplementary Figure 6_Source Data/Suppl Fig6a/BMDM_COMPL_plus_14_MERGE.tif]

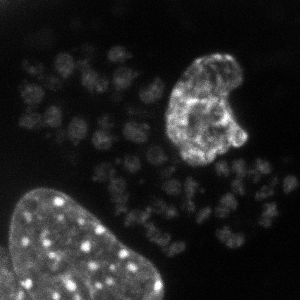

Supplement: Supplementary file 15 — Source data [file 41467_2025_58876_MOESM15_ESM.zip › Source suppl/Supplementary Figure 6_Source Data/Suppl Fig6a/MEF_COMPL_minus_6_DAPI.tif]

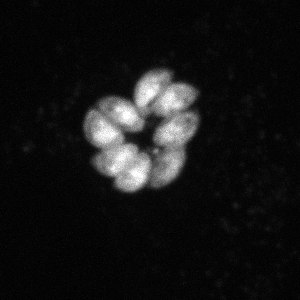

Supplement: Supplementary file 15 — Source data [file 41467_2025_58876_MOESM15_ESM.zip › Source suppl/Supplementary Figure 6_Source Data/Suppl Fig6a/MEF_COMPL_plus_12_toxo.tif]

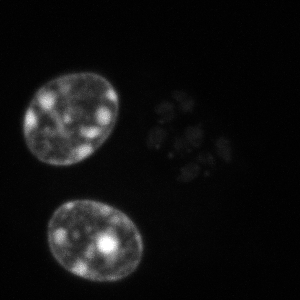

Supplement: Supplementary file 15 — Source data [file 41467_2025_58876_MOESM15_ESM.zip › Source suppl/Supplementary Figure 6_Source Data/Suppl Fig6a/BMDM_COMPL_plus_14_DAPI.tif]

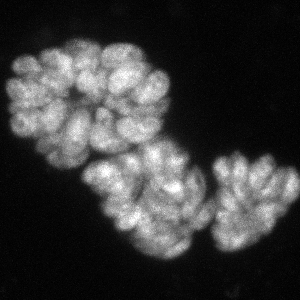

Supplement: Supplementary file 15 — Source data [file 41467_2025_58876_MOESM15_ESM.zip › Source suppl/Supplementary Figure 6_Source Data/Suppl Fig6a/MEF_COMPL_minus_6_toxo.tif]

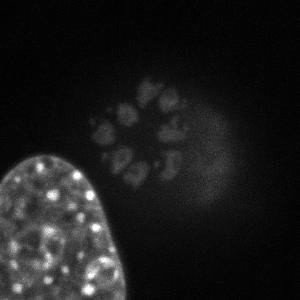

Supplement: Supplementary file 15 — Source data [file 41467_2025_58876_MOESM15_ESM.zip › Source suppl/Supplementary Figure 6_Source Data/Suppl Fig6a/MEF_COMPL_plus_12_DAPI.tif]

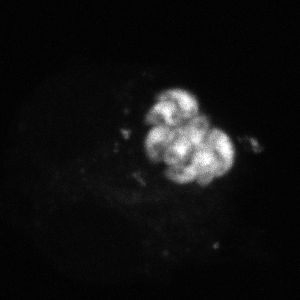

Supplement: Supplementary file 15 — Source data [file 41467_2025_58876_MOESM15_ESM.zip › Source suppl/Supplementary Figure 6_Source Data/Suppl Fig6a/BMDM_COMPL_plus_14_toxo.tif]

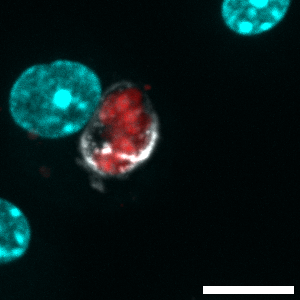

Supplement: Supplementary file 15 — Source data [file 41467_2025_58876_MOESM15_ESM.zip › Source suppl/Supplementary Figure 6_Source Data/Suppl Fig6a/BMDM_COMPL_minus_9_MERGE.tif]

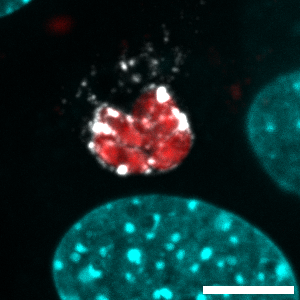

Supplement: Supplementary file 15 — Source data [file 41467_2025_58876_MOESM15_ESM.zip › Source suppl/Supplementary Figure 6_Source Data/Suppl Fig6a/MEF_COMPL_minus_3_MERGE.tif]

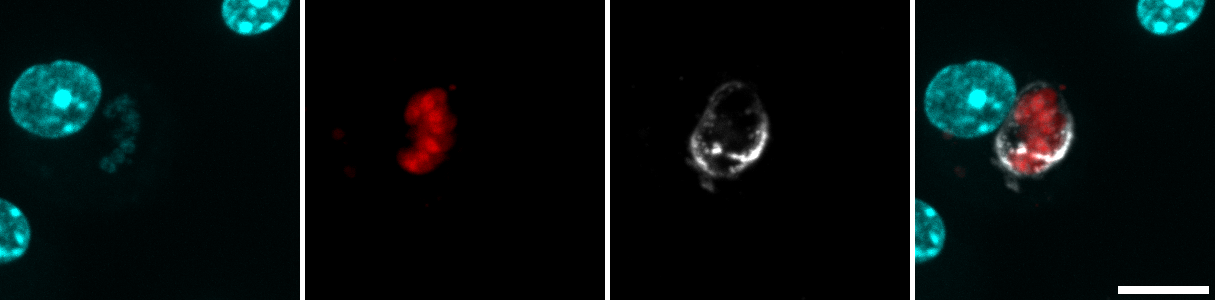

Supplement: Supplementary file 15 — Source data [file 41467_2025_58876_MOESM15_ESM.zip › Source suppl/Supplementary Figure 6_Source Data/Suppl Fig6a/BMDM_COMPL_minus_9_MONTAGE.tif]

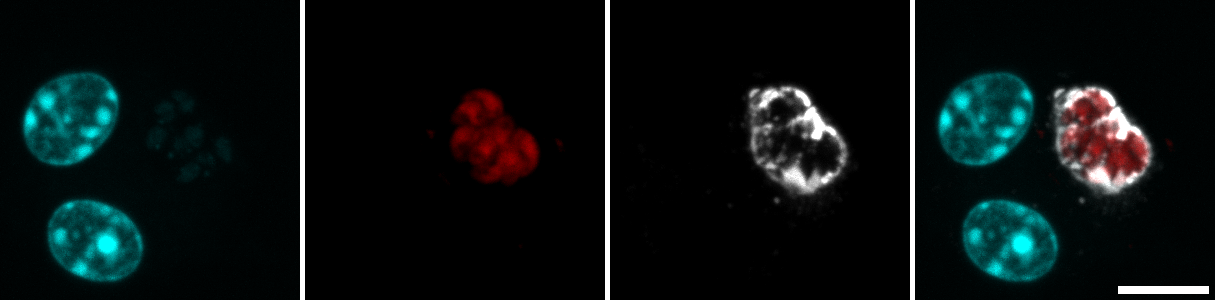

Supplement: Supplementary file 15 — Source data [file 41467_2025_58876_MOESM15_ESM.zip › Source suppl/Supplementary Figure 6_Source Data/Suppl Fig6a/BMDM_COMPL_plus_14_MONTAGE.tif]

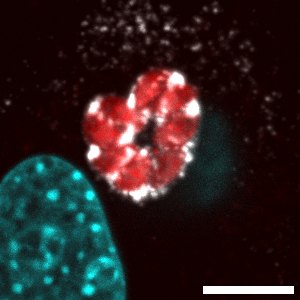

Supplement: Supplementary file 15 — Source data [file 41467_2025_58876_MOESM15_ESM.zip › Source suppl/Supplementary Figure 6_Source Data/Suppl Fig6a/MEF_COMPL_plus_12_MERGE.tif]

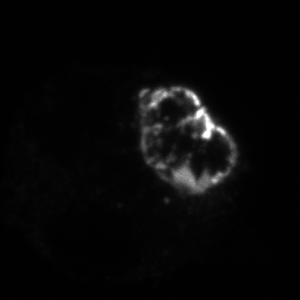

Supplement: Supplementary file 15 — Source data [file 41467_2025_58876_MOESM15_ESM.zip › Source suppl/Supplementary Figure 6_Source Data/Suppl Fig6a/BMDM_COMPL_plus_14_HA.tif]

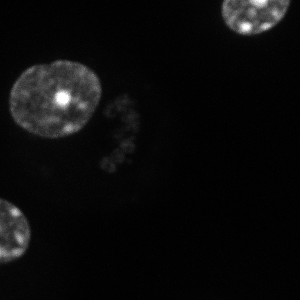

Supplement: Supplementary file 15 — Source data [file 41467_2025_58876_MOESM15_ESM.zip › Source suppl/Supplementary Figure 6_Source Data/Suppl Fig6a/BMDM_COMPL_minus_9_DAPI.tif]

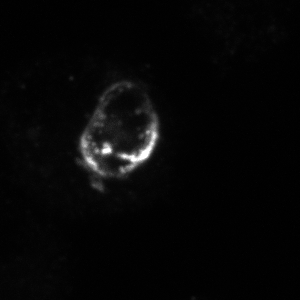

Supplement: Supplementary file 15 — Source data [file 41467_2025_58876_MOESM15_ESM.zip › Source suppl/Supplementary Figure 6_Source Data/Suppl Fig6a/BMDM_COMPL_minus_9_HA.tif]

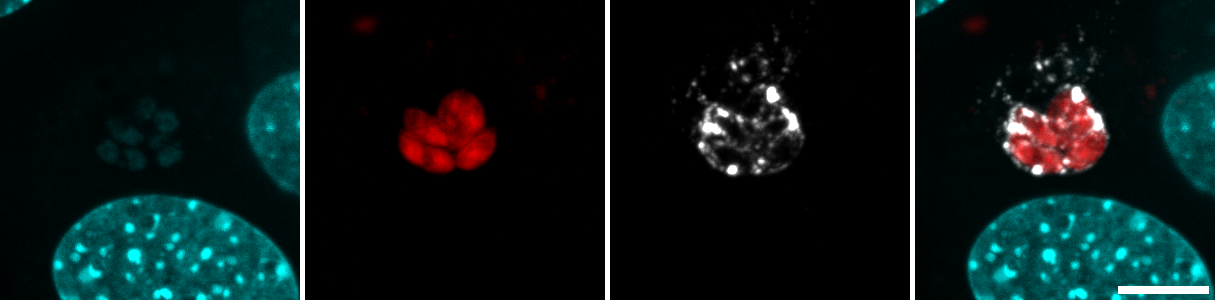

Supplement: Supplementary file 15 — Source data [file 41467_2025_58876_MOESM15_ESM.zip › Source suppl/Supplementary Figure 6_Source Data/Suppl Fig6a/MEF_COMPL_minus_3_MONTAGE.tif]

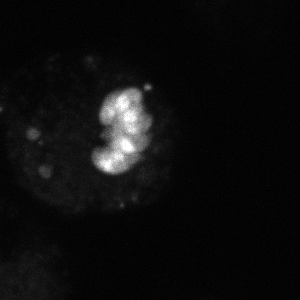

Supplement: Supplementary file 15 — Source data [file 41467_2025_58876_MOESM15_ESM.zip › Source suppl/Supplementary Figure 6_Source Data/Suppl Fig6a/BMDM_COMPL_minus_9_toxo.tif]

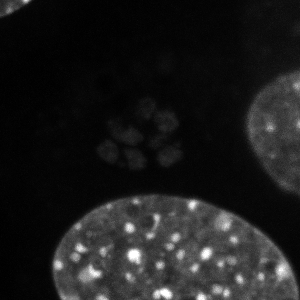

Supplement: Supplementary file 15 — Source data [file 41467_2025_58876_MOESM15_ESM.zip › Source suppl/Supplementary Figure 6_Source Data/Suppl Fig6a/MEF_COMPL_minus_3_DAPI.tif]

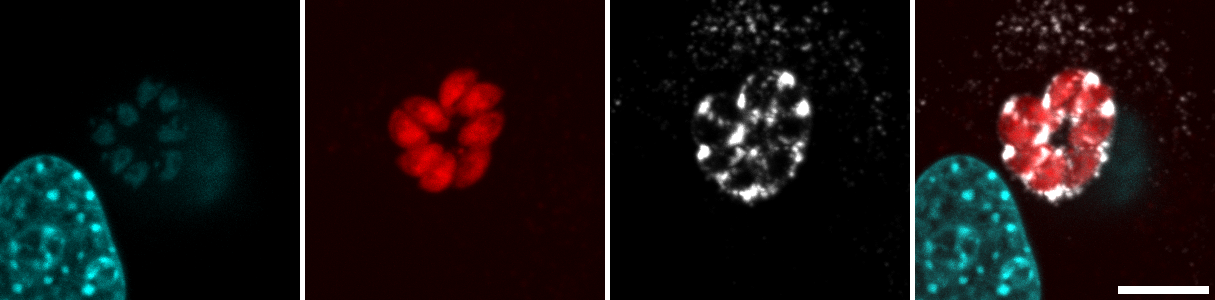

Supplement: Supplementary file 15 — Source data [file 41467_2025_58876_MOESM15_ESM.zip › Source suppl/Supplementary Figure 6_Source Data/Suppl Fig6a/MEF_COMPL_plus_12_MONTAGE.tif]

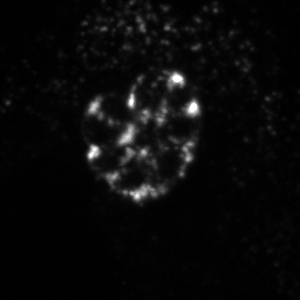

Supplement: Supplementary file 15 — Source data [file 41467_2025_58876_MOESM15_ESM.zip › Source suppl/Supplementary Figure 6_Source Data/Suppl Fig6a/MEF_COMPL_plus_12_HA.tif]

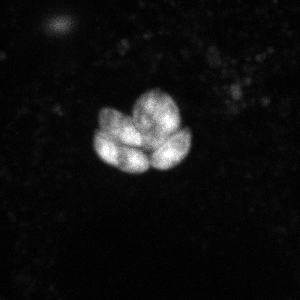

Supplement: Supplementary file 15 — Source data [file 41467_2025_58876_MOESM15_ESM.zip › Source suppl/Supplementary Figure 6_Source Data/Suppl Fig6a/MEF_COMPL_minus_3_toxo.tif]

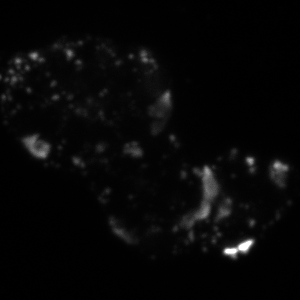

Supplement: Supplementary file 15 — Source data [file 41467_2025_58876_MOESM15_ESM.zip › Source suppl/Supplementary Figure 6_Source Data/Suppl Fig6a/MEF_COMPL_minus_6_HA.tif]

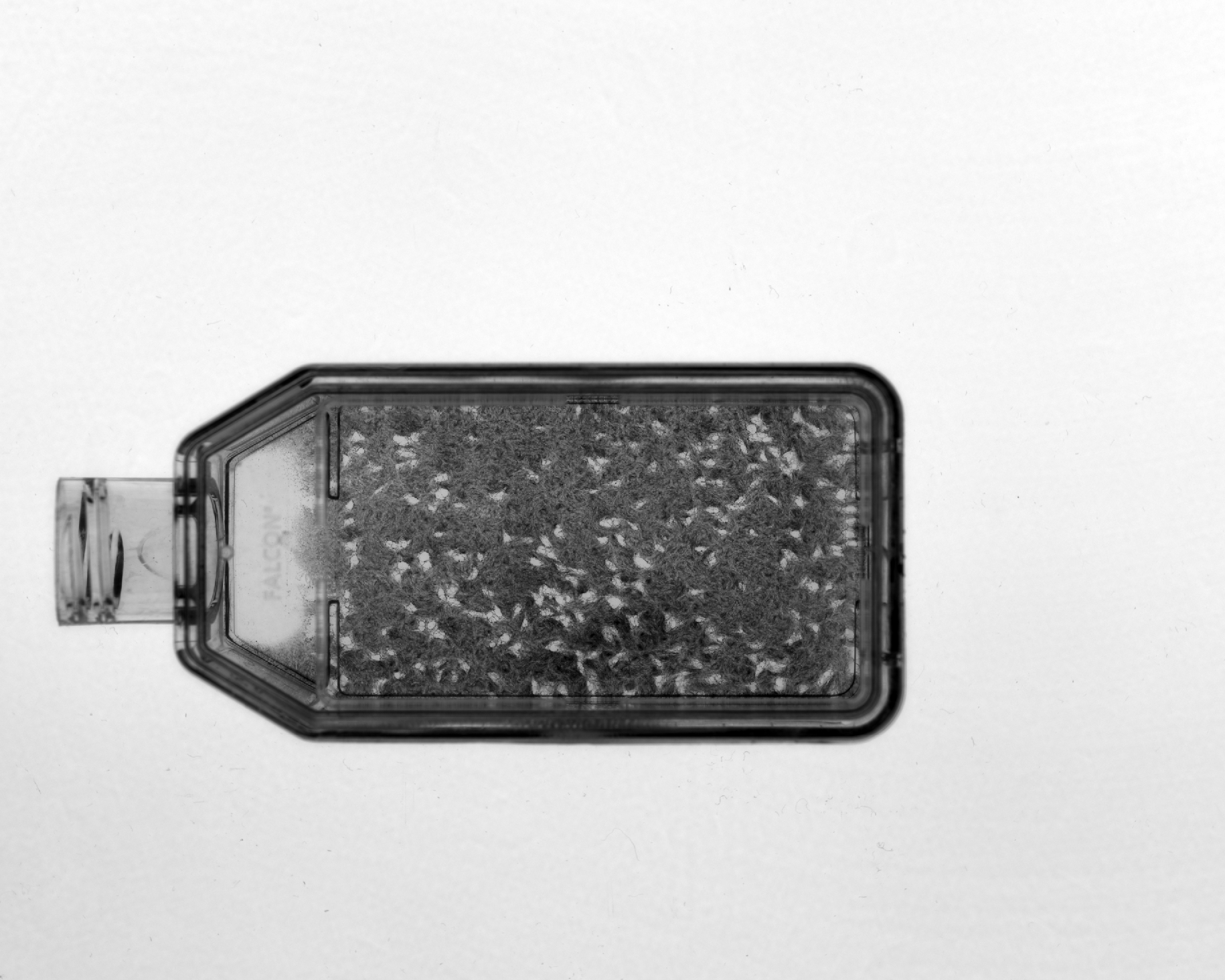

Supplement: Supplementary file 15 — Source data [file 41467_2025_58876_MOESM15_ESM.zip › Source suppl/Supplementary Figure 3_Source Data/Suppl Fig3g/vand uprt 500(Silver Stain).jpg]

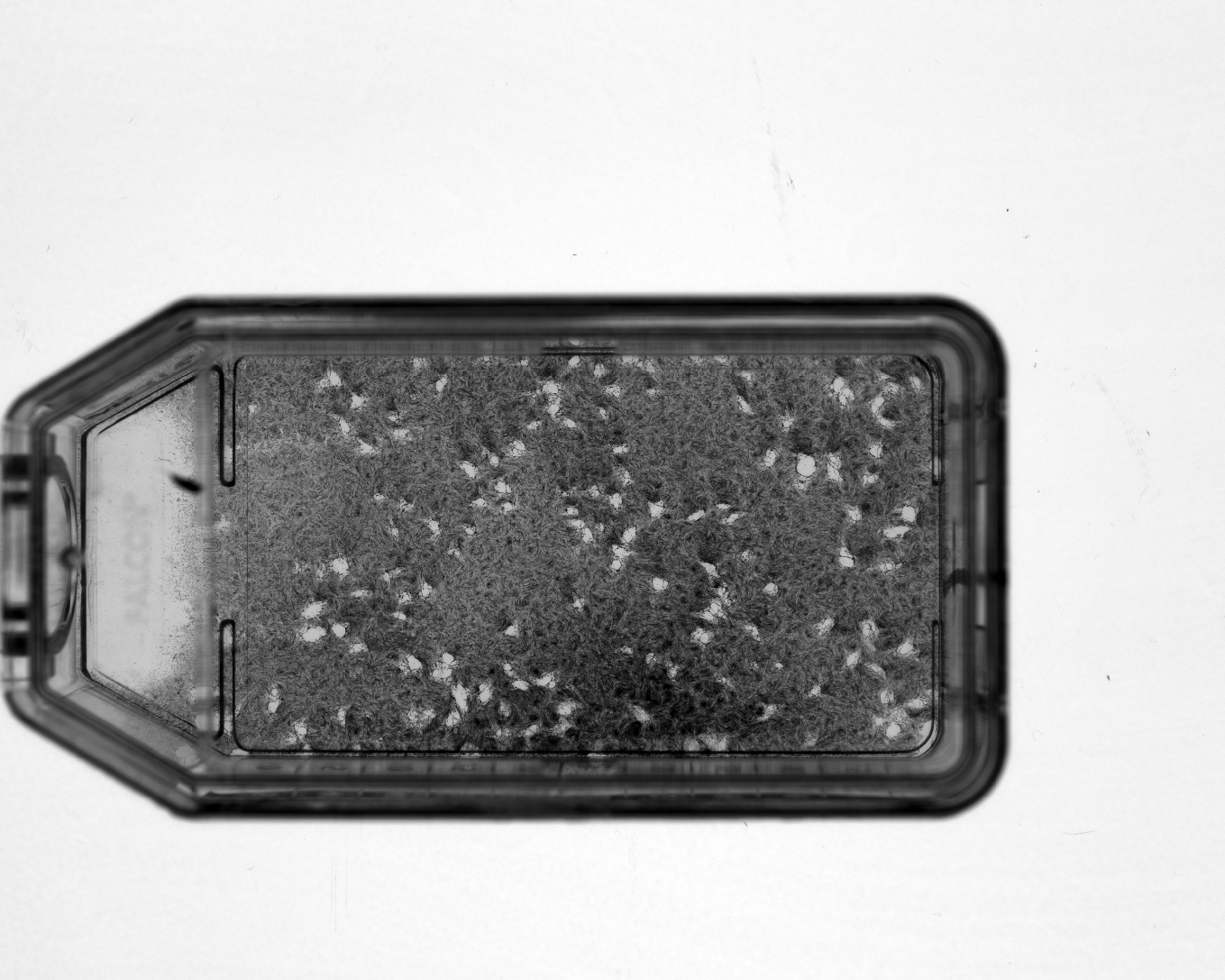

Supplement: Supplementary file 15 — Source data [file 41467_2025_58876_MOESM15_ESM.zip › Source suppl/Supplementary Figure 3_Source Data/Suppl Fig3g/VAND_ROP18_500(Silver Stain).jpg]

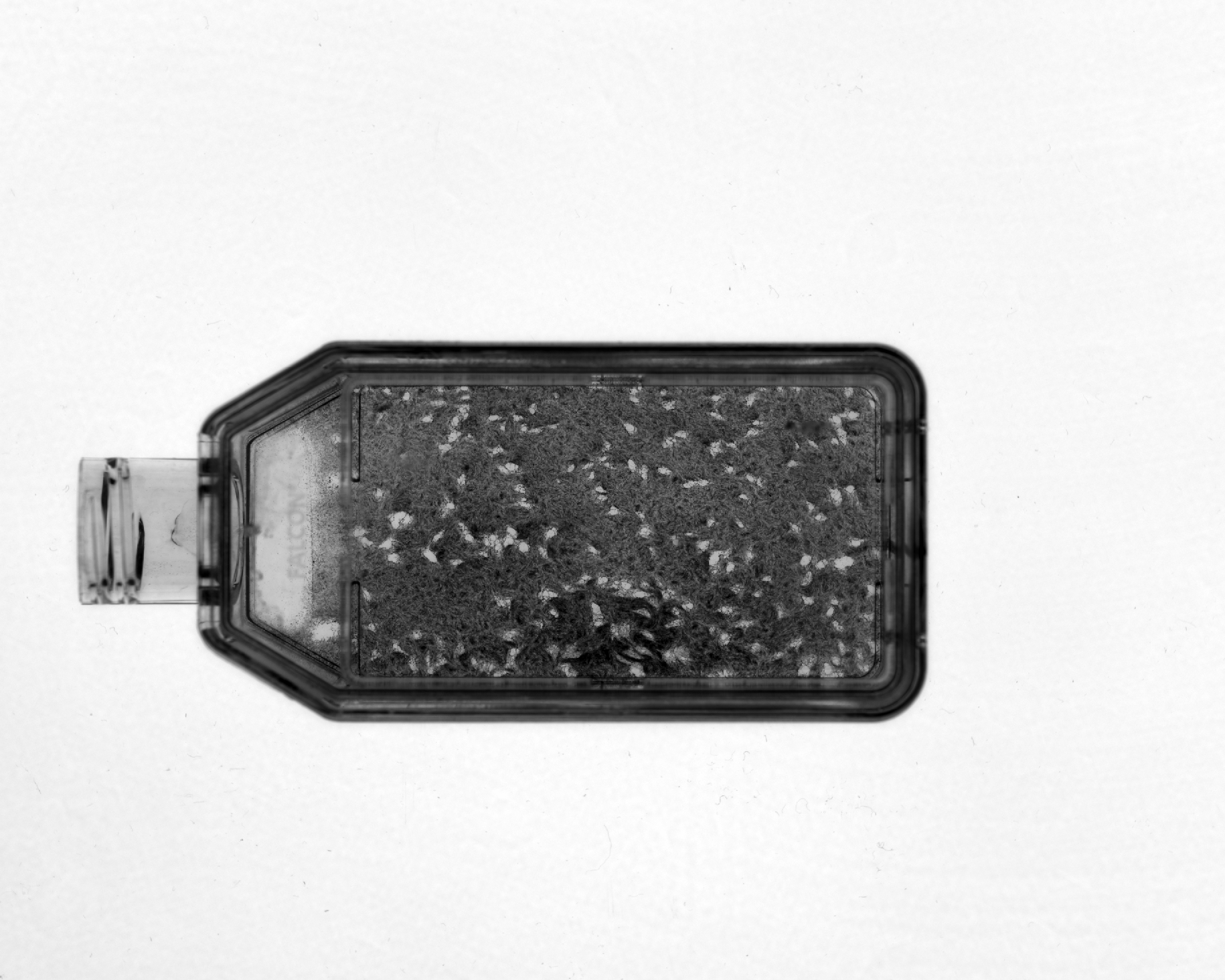

Supplement: Supplementary file 15 — Source data [file 41467_2025_58876_MOESM15_ESM.zip › Source suppl/Supplementary Figure 3_Source Data/Suppl Fig3g/vand ku 500(Silver Stain).jpg]

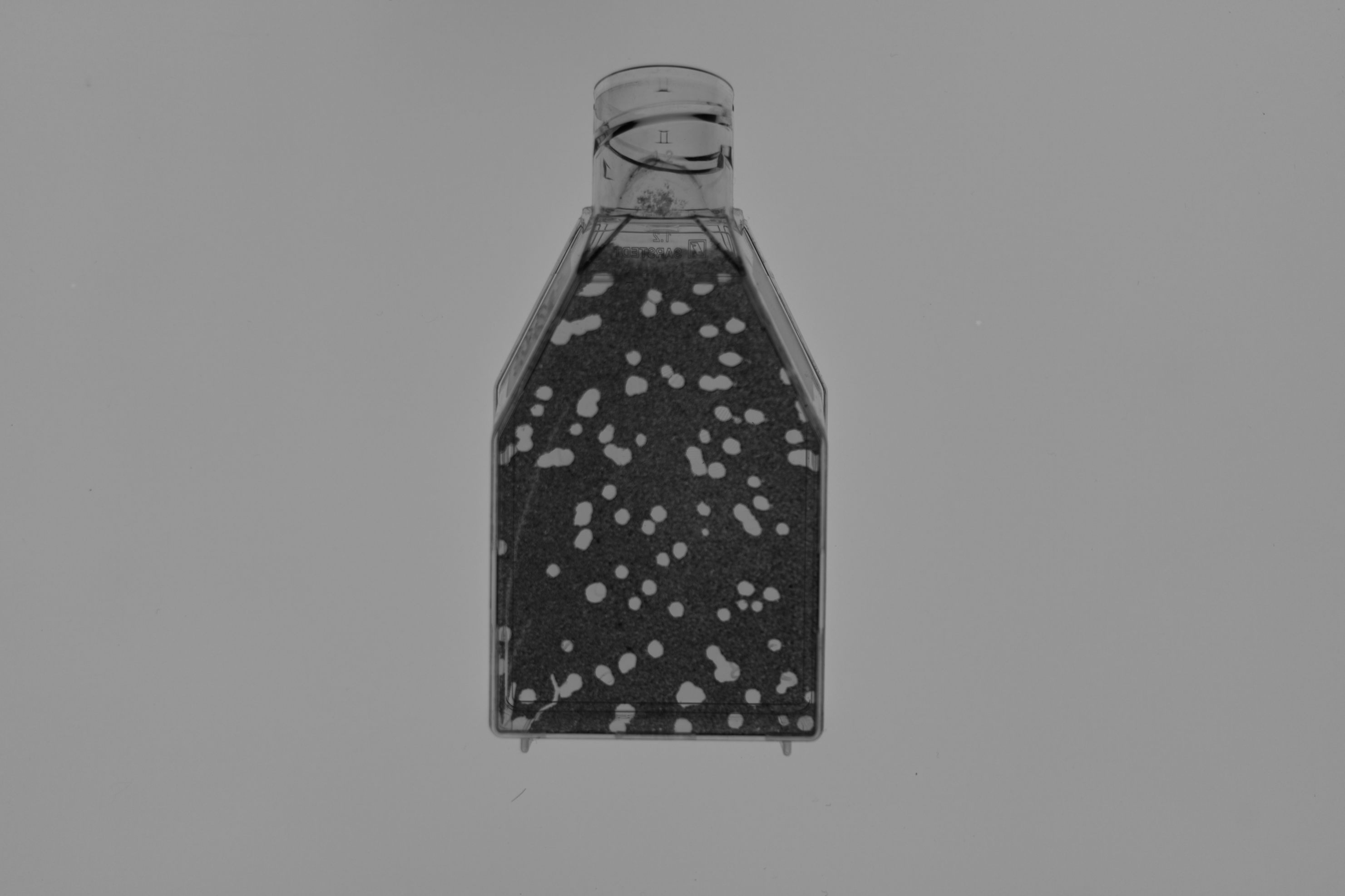

Supplement: Supplementary file 15 — Source data [file 41467_2025_58876_MOESM15_ESM.zip › Source suppl/Supplementary Figure 3_Source Data/Suppl Fig3d/igcuser 2024-12-16 16h56m58s(Silver Stain).raw16.tif]

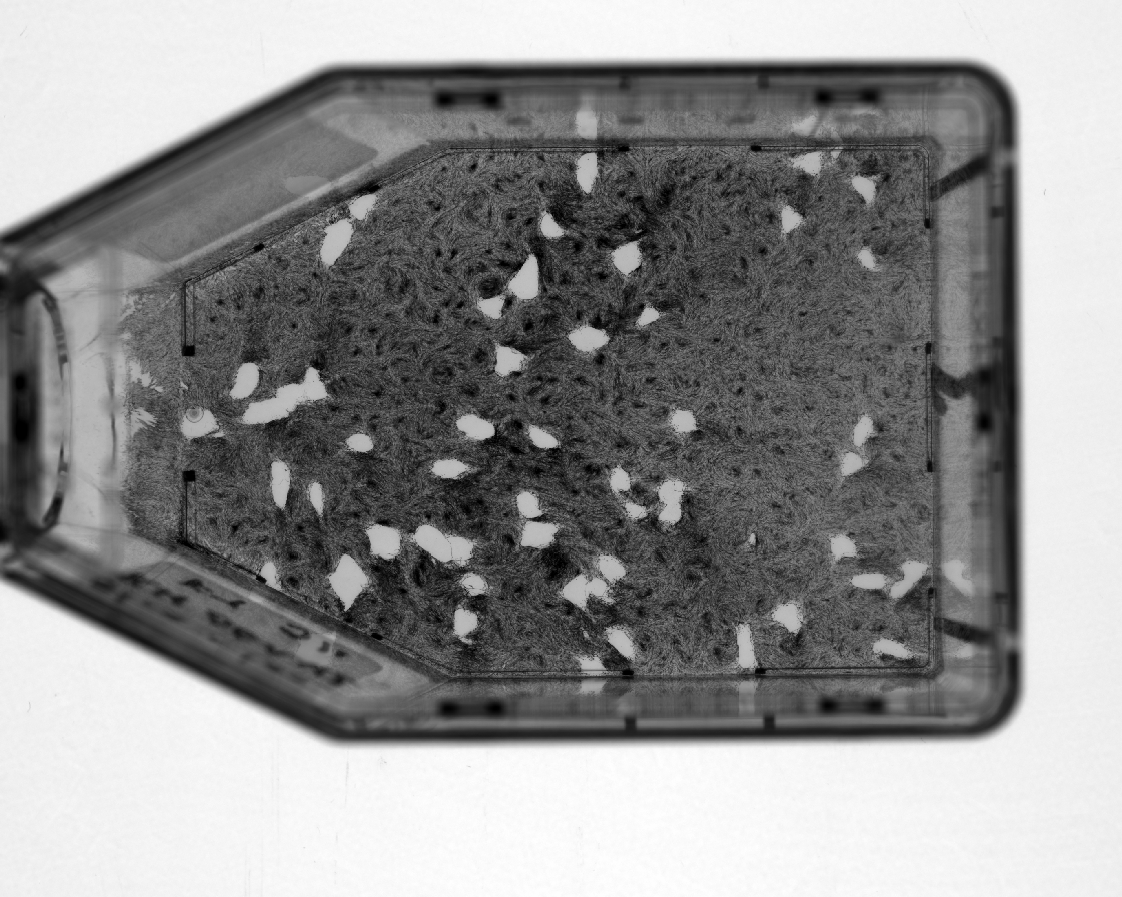

Supplement: Supplementary file 15 — Source data [file 41467_2025_58876_MOESM15_ESM.zip › Source suppl/Supplementary Figure 3_Source Data/Suppl Fig3d/jan_dgra12(Silver Stain).tif]

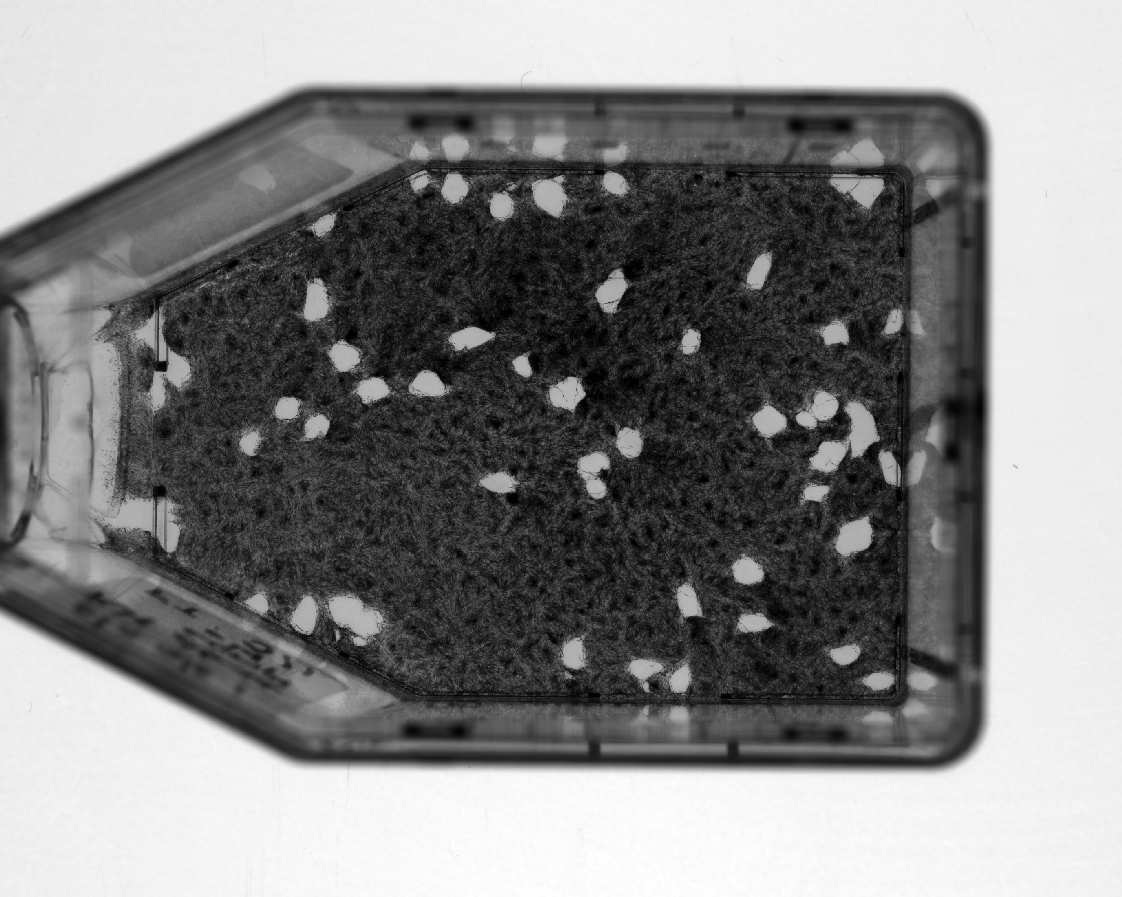

Supplement: Supplementary file 15 — Source data [file 41467_2025_58876_MOESM15_ESM.zip › Source suppl/Supplementary Figure 3_Source Data/Suppl Fig3d/jan_compl_old(Silver Stain).tif]

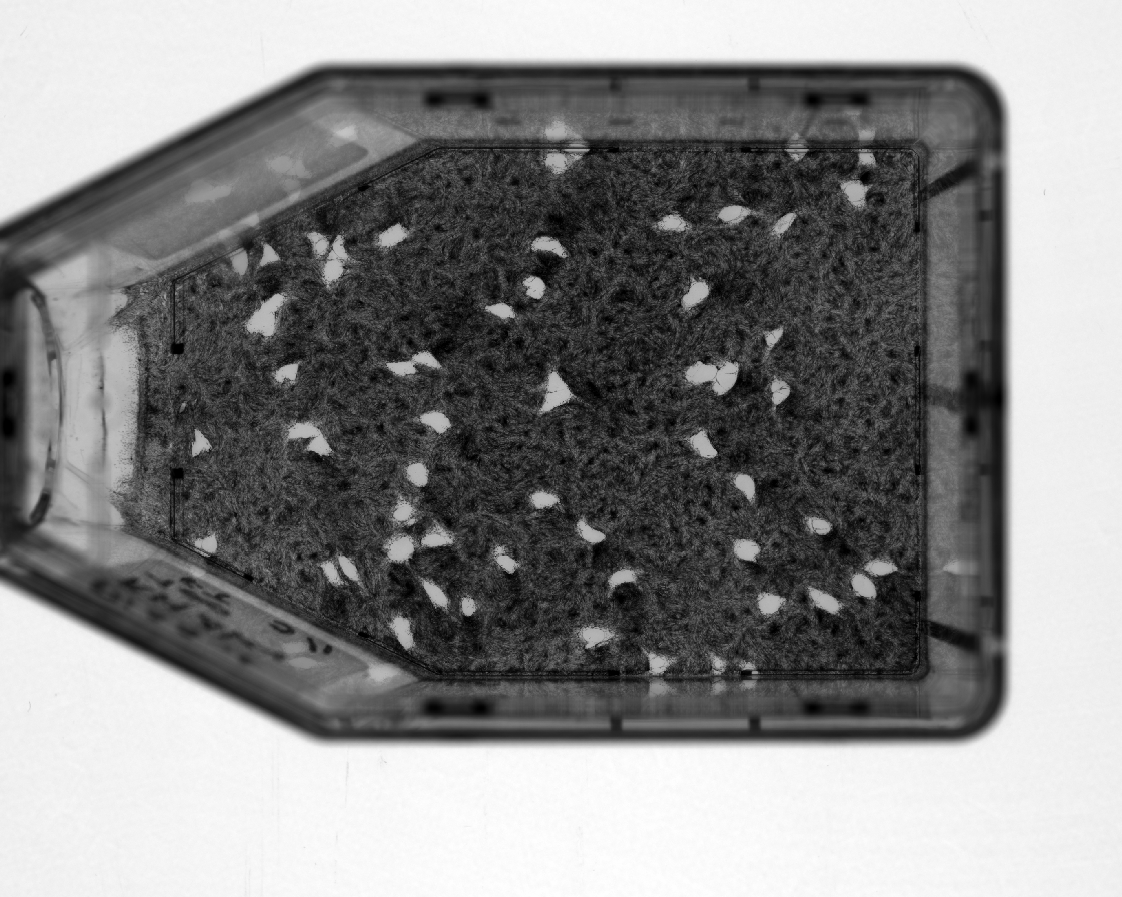

Supplement: Supplementary file 15 — Source data [file 41467_2025_58876_MOESM15_ESM.zip › Source suppl/Supplementary Figure 3_Source Data/Suppl Fig3d/jan_ku80(Silver Stain).tif]

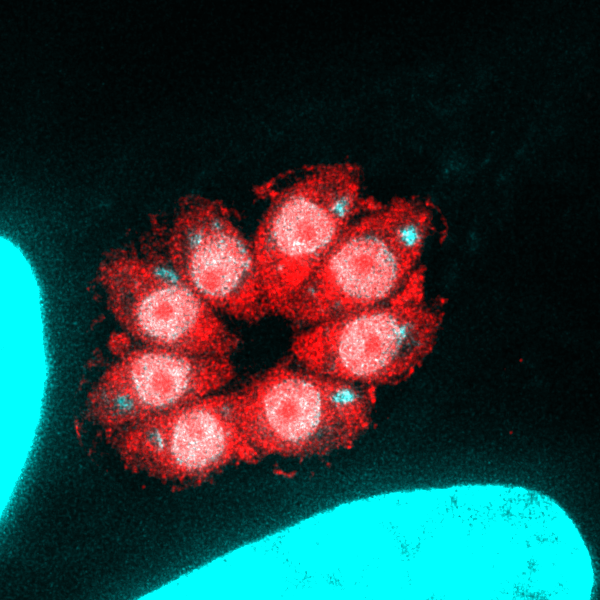

Supplement: Supplementary file 15 — Source data [file 41467_2025_58876_MOESM15_ESM.zip › Source suppl/Supplementary Figure 3_Source Data/Suppl Fig3e/FT_230201_dGRA12_HA488_GRA2647_150x_F_8_MMStack_Pos0.ome_crop-0003_adj_MERGE_NEW.tif]

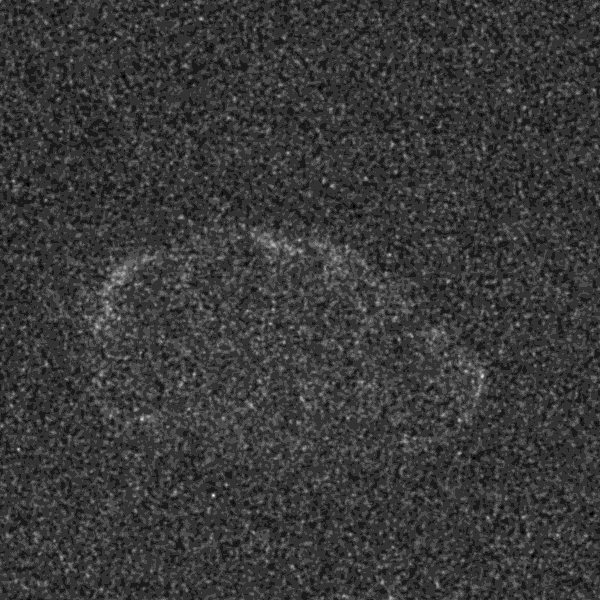

Supplement: Supplementary file 15 — Source data [file 41467_2025_58876_MOESM15_ESM.zip › Source suppl/Supplementary Figure 3_Source Data/Suppl Fig3e/FT_230201_Ku80_HA488_GRA3_647_150x_A_3_MMStack_Pos0.ome_crop-0004.tif]

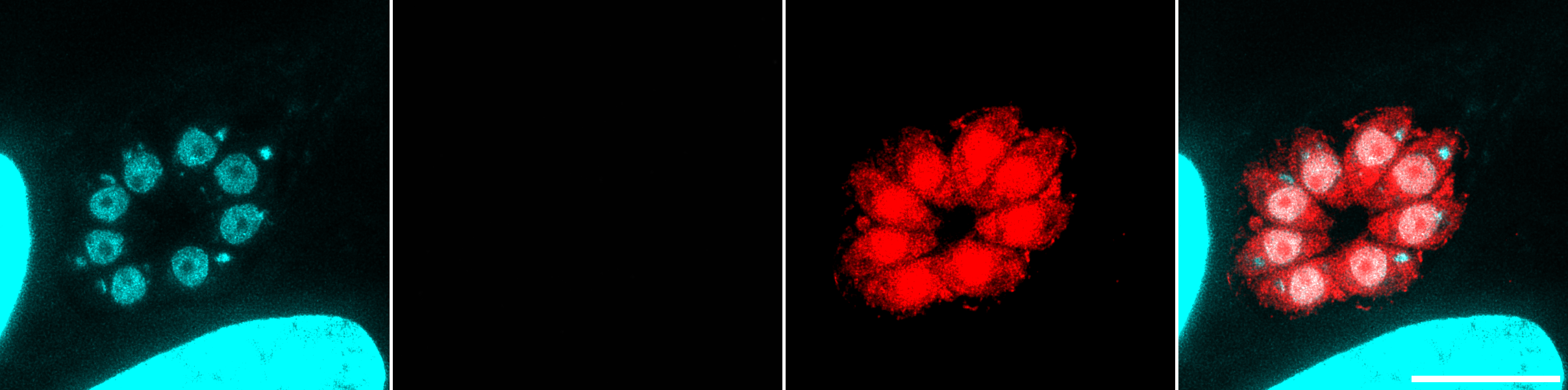

Supplement: Supplementary file 15 — Source data [file 41467_2025_58876_MOESM15_ESM.zip › Source suppl/Supplementary Figure 3_Source Data/Suppl Fig3e/FT_230201_dGRA12_HA488_GRA2647_150x_F_8_MMStack_Pos0.ome_crop-0003_adj_MERGE_NEW_scale_MONTAGE.tif]

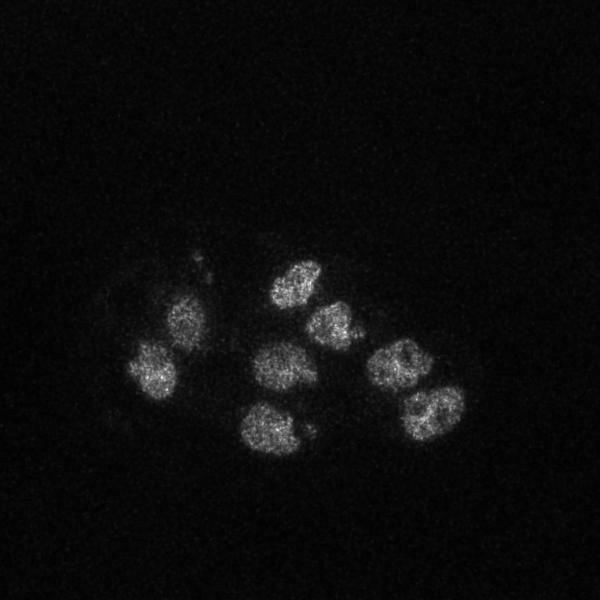

Supplement: Supplementary file 15 — Source data [file 41467_2025_58876_MOESM15_ESM.zip › Source suppl/Supplementary Figure 3_Source Data/Suppl Fig3e/FT_230201_Ku80_HA488_GRA3_647_150x_A_3_MMStack_Pos0.ome_crop-0002.tif]

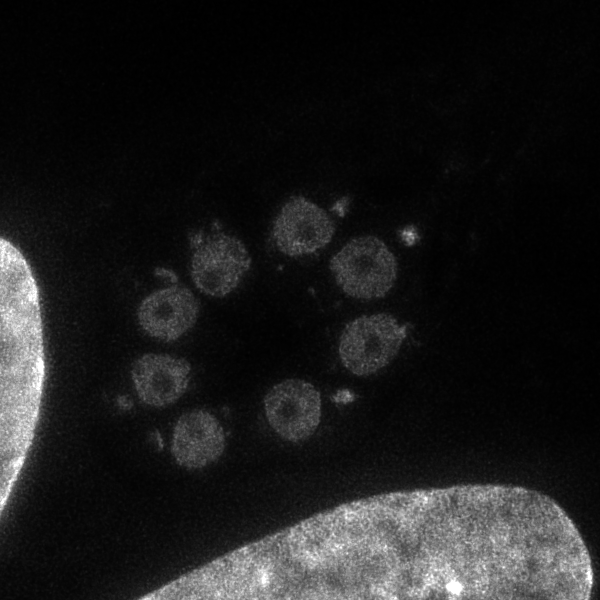

Supplement: Supplementary file 15 — Source data [file 41467_2025_58876_MOESM15_ESM.zip › Source suppl/Supplementary Figure 3_Source Data/Suppl Fig3e/FT_230201_dGRA12_HA488_GRA2647_150x_F_8_MMStack_Pos0.ome_crop-0002_adj.tif]

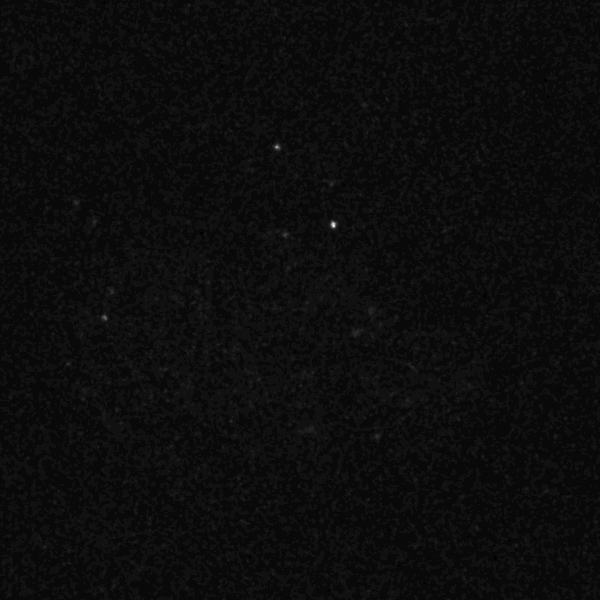

Supplement: Supplementary file 15 — Source data [file 41467_2025_58876_MOESM15_ESM.zip › Source suppl/Supplementary Figure 3_Source Data/Suppl Fig3e/FT_230201_Ku80_HA488_GRA3_647_150x_A_3_MMStack_Pos0.ome_crop-0001.tif]

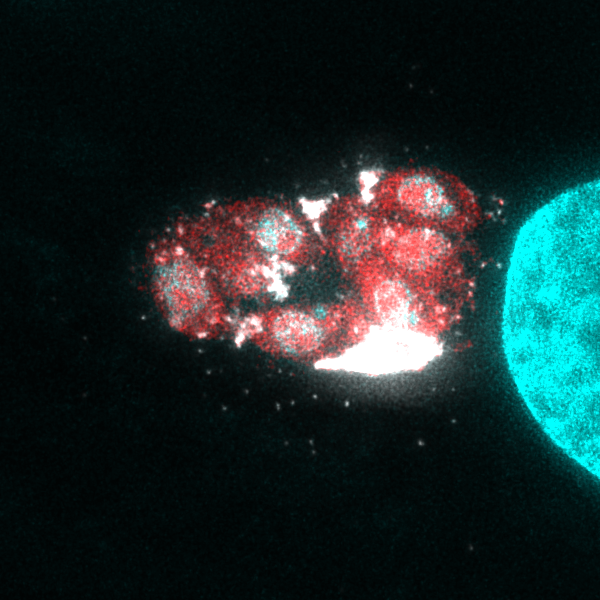

Supplement: Supplementary file 15 — Source data [file 41467_2025_58876_MOESM15_ESM.zip › Source suppl/Supplementary Figure 3_Source Data/Suppl Fig3e/FT_230201_dGRA12_HA488_GRA2647_150x_F_8_MMStack_Pos0.ome_crop-0001_adj_compl_MERGE_new.tif]

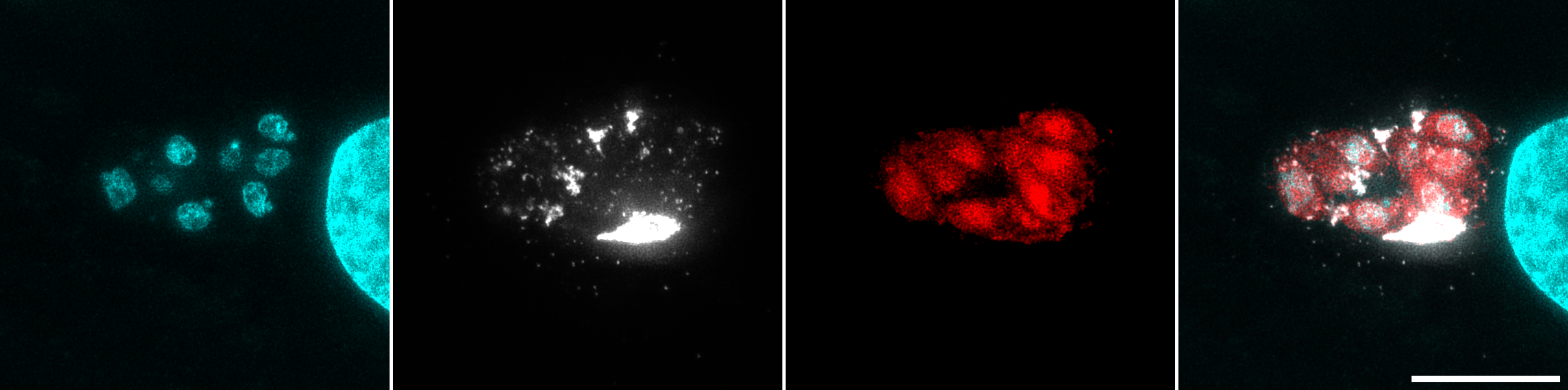

Supplement: Supplementary file 15 — Source data [file 41467_2025_58876_MOESM15_ESM.zip › Source suppl/Supplementary Figure 3_Source Data/Suppl Fig3e/FT_230201_dGRA12_HA488_GRA2647_150x_F_8_MMStack_Pos0.ome_crop-0001_adj_compl_MERGE_new_SCALE_MONTAGE.tif]

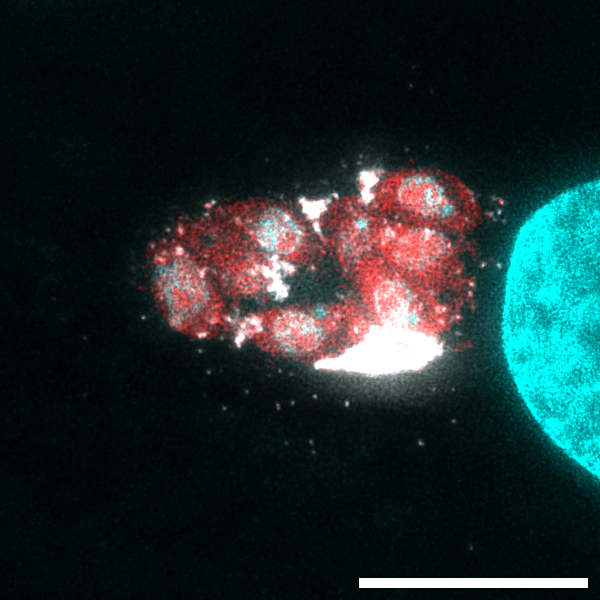

Supplement: Supplementary file 15 — Source data [file 41467_2025_58876_MOESM15_ESM.zip › Source suppl/Supplementary Figure 3_Source Data/Suppl Fig3e/FT_230201_dGRA12_HA488_GRA2647_150x_F_8_MMStack_Pos0.ome_crop-0001_adj_compl_MERGE_new_SCALE.tif]

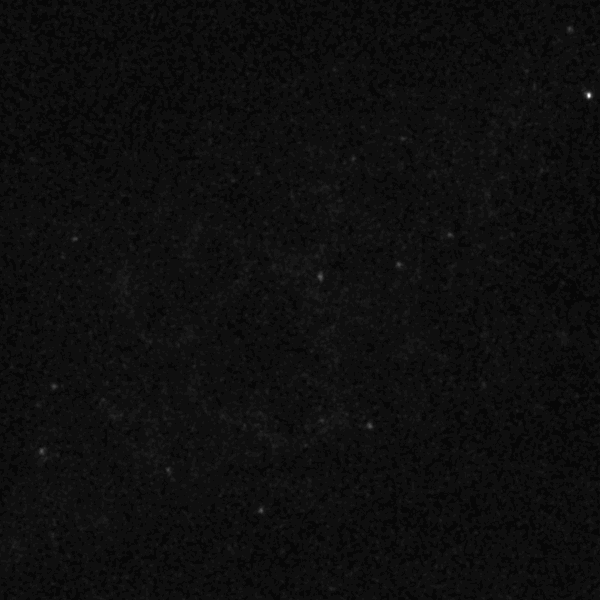

Supplement: Supplementary file 15 — Source data [file 41467_2025_58876_MOESM15_ESM.zip › Source suppl/Supplementary Figure 3_Source Data/Suppl Fig3e/FT_230201_dGRA12_HA488_GRA2647_150x_F_8_MMStack_Pos0.ome_crop-0001_adj.tif]

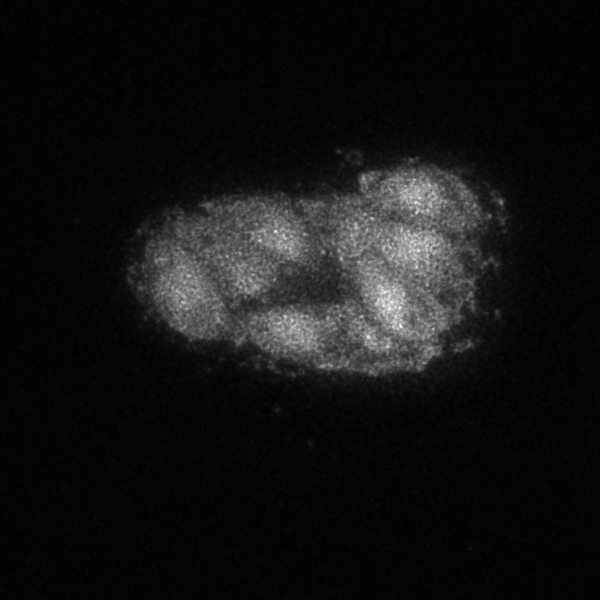

Supplement: Supplementary file 15 — Source data [file 41467_2025_58876_MOESM15_ESM.zip › Source suppl/Supplementary Figure 3_Source Data/Suppl Fig3e/FT_230206_dGRA12_HA488_GRA3_647_150x_B_8_MMStack_Pos0_crop.ome-0004_adj.tif]

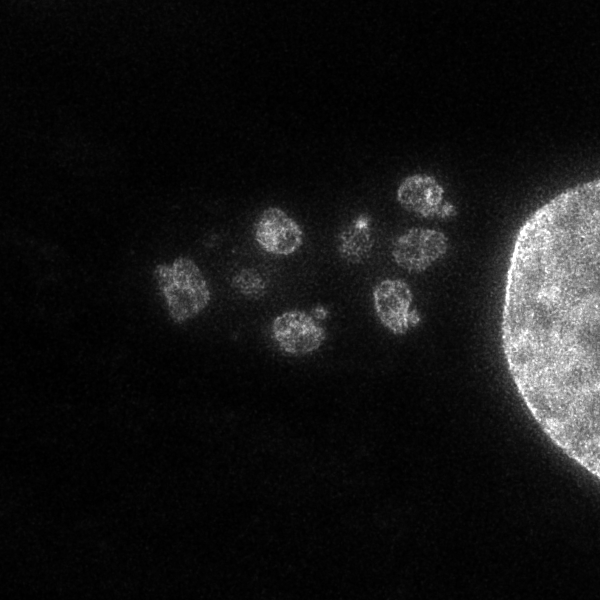

Supplement: Supplementary file 15 — Source data [file 41467_2025_58876_MOESM15_ESM.zip › Source suppl/Supplementary Figure 3_Source Data/Suppl Fig3e/FT_230206_dGRA12_HA488_GRA3_647_150x_B_8_MMStack_Pos0_crop.ome-0002.tif]

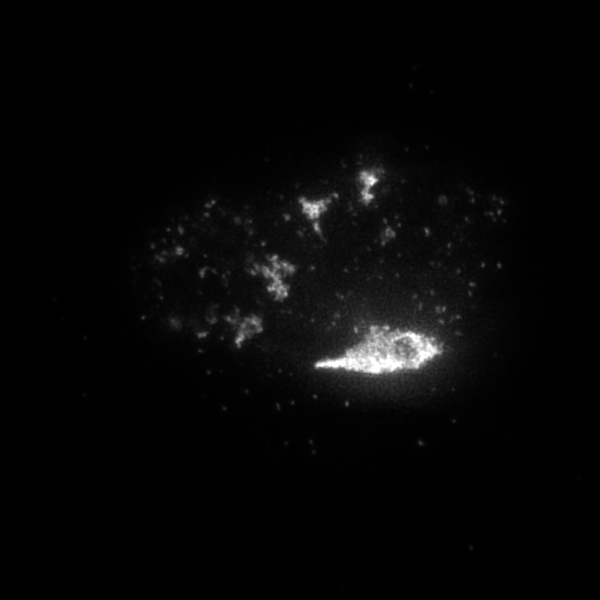

Supplement: Supplementary file 15 — Source data [file 41467_2025_58876_MOESM15_ESM.zip › Source suppl/Supplementary Figure 3_Source Data/Suppl Fig3e/FT_230206_dGRA12_HA488_GRA3_647_150x_B_8_MMStack_Pos0_crop.ome-0001_adj.tif]

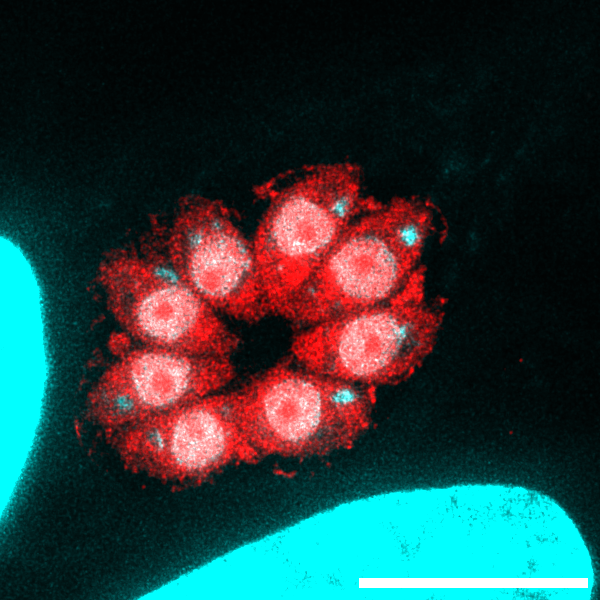

Supplement: Supplementary file 15 — Source data [file 41467_2025_58876_MOESM15_ESM.zip › Source suppl/Supplementary Figure 3_Source Data/Suppl Fig3e/FT_230201_dGRA12_HA488_GRA2647_150x_F_8_MMStack_Pos0.ome_crop-0003_adj_MERGE_NEW_scale.tif]

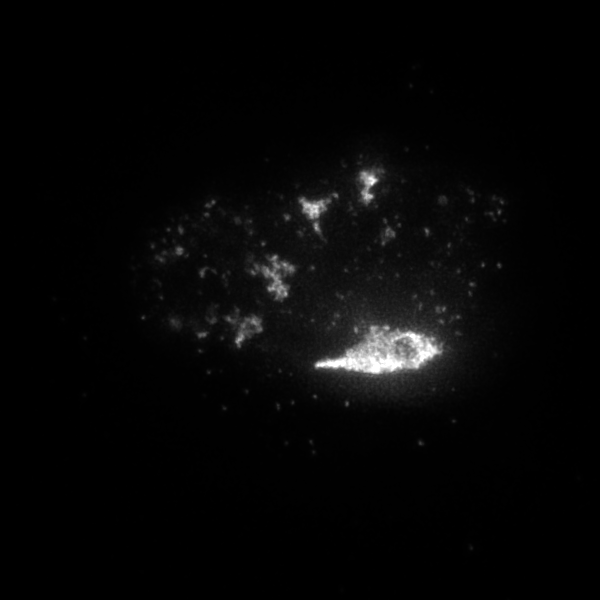

Supplement: Supplementary file 15 — Source data [file 41467_2025_58876_MOESM15_ESM.zip › Source suppl/Supplementary Figure 3_Source Data/Suppl Fig3e/FT_230206_dGRA12_HA488_GRA3_647_150x_B_8_MMStack_Pos0_crop.ome-0001.tif]

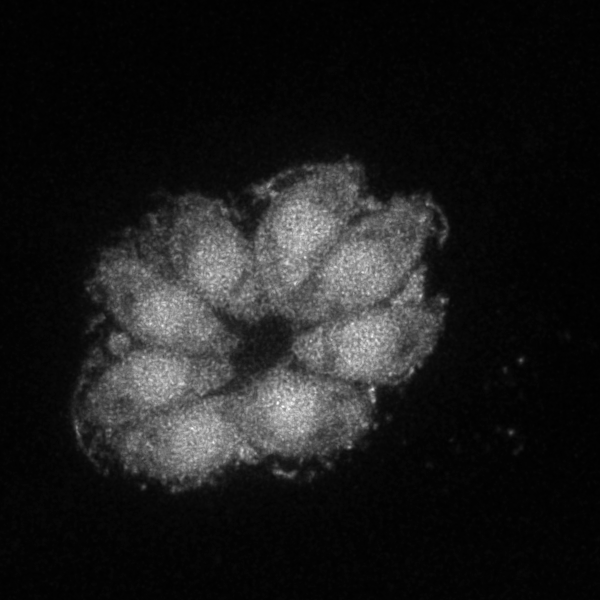

Supplement: Supplementary file 15 — Source data [file 41467_2025_58876_MOESM15_ESM.zip › Source suppl/Supplementary Figure 3_Source Data/Suppl Fig3e/FT_230201_dGRA12_HA488_GRA2647_150x_F_8_MMStack_Pos0.ome_crop-0004_adj.tif]

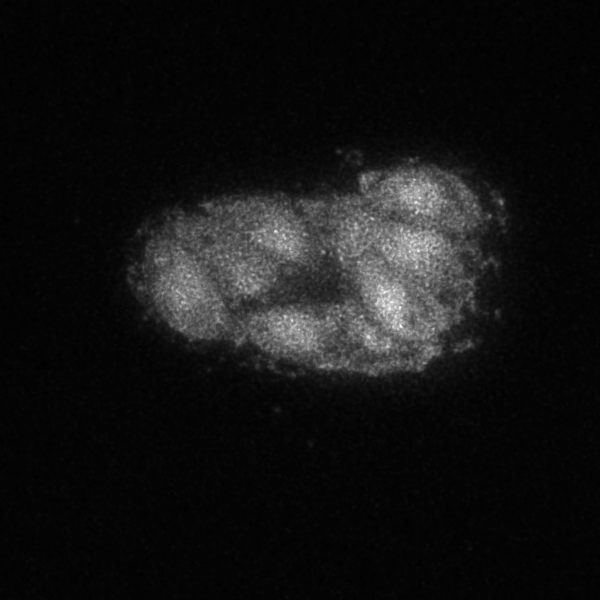

Supplement: Supplementary file 15 — Source data [file 41467_2025_58876_MOESM15_ESM.zip › Source suppl/Supplementary Figure 3_Source Data/Suppl Fig3e/FT_230206_dGRA12_HA488_GRA3_647_150x_B_8_MMStack_Pos0_crop.ome-0004.tif]

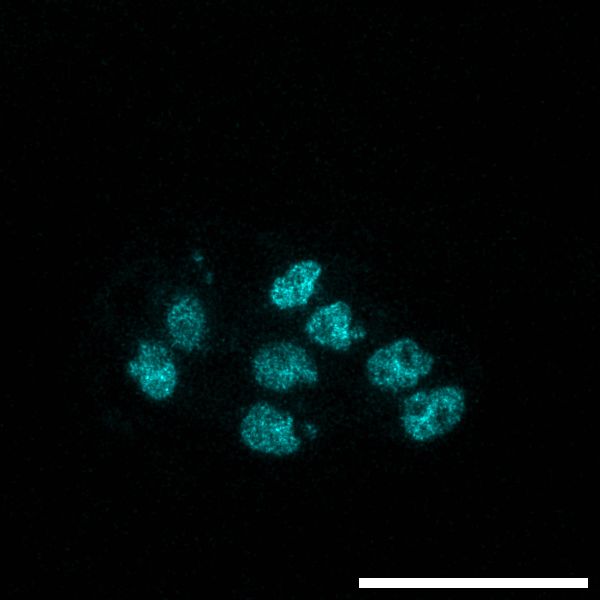

Supplement: Supplementary file 15 — Source data [file 41467_2025_58876_MOESM15_ESM.zip › Source suppl/Supplementary Figure 3_Source Data/Suppl Fig3e/FT_230201_Ku80_HA488_GRA3_647_150x_A_3_MMStack_Pos0.ome_crop-0003_MERGE_scale.tif]

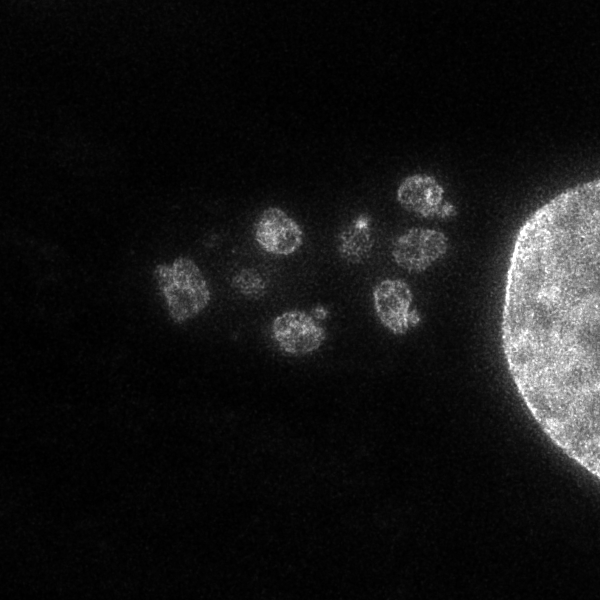

Supplement: Supplementary file 15 — Source data [file 41467_2025_58876_MOESM15_ESM.zip › Source suppl/Supplementary Figure 3_Source Data/Suppl Fig3e/FT_230206_dGRA12_HA488_GRA3_647_150x_B_8_MMStack_Pos0_crop.ome-0002_adj.tif]

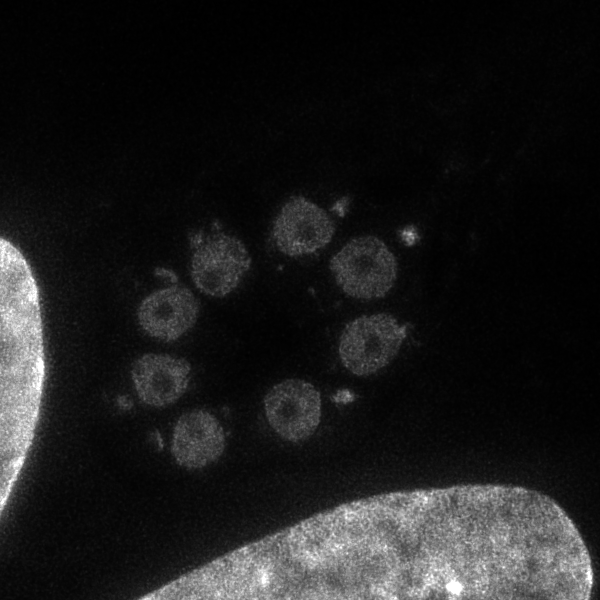

Supplement: Supplementary file 15 — Source data [file 41467_2025_58876_MOESM15_ESM.zip › Source suppl/Supplementary Figure 3_Source Data/Suppl Fig3e/FT_230201_dGRA12_HA488_GRA2647_150x_F_8_MMStack_Pos0.ome_crop-0002.tif]

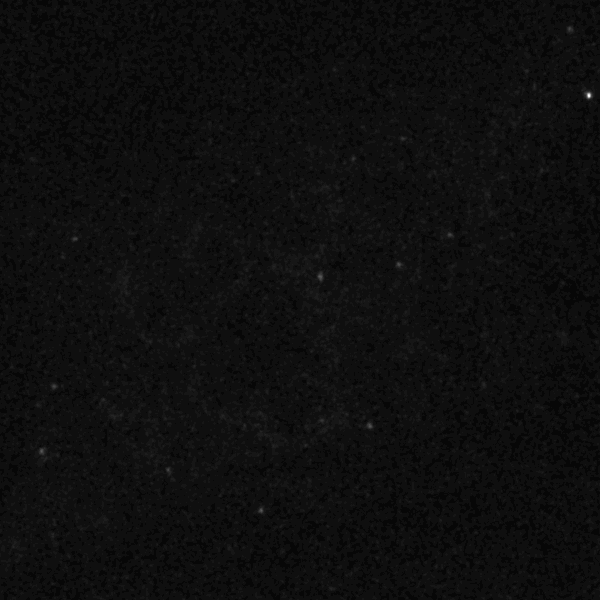

Supplement: Supplementary file 15 — Source data [file 41467_2025_58876_MOESM15_ESM.zip › Source suppl/Supplementary Figure 3_Source Data/Suppl Fig3e/FT_230201_dGRA12_HA488_GRA2647_150x_F_8_MMStack_Pos0.ome_crop-0001.tif]

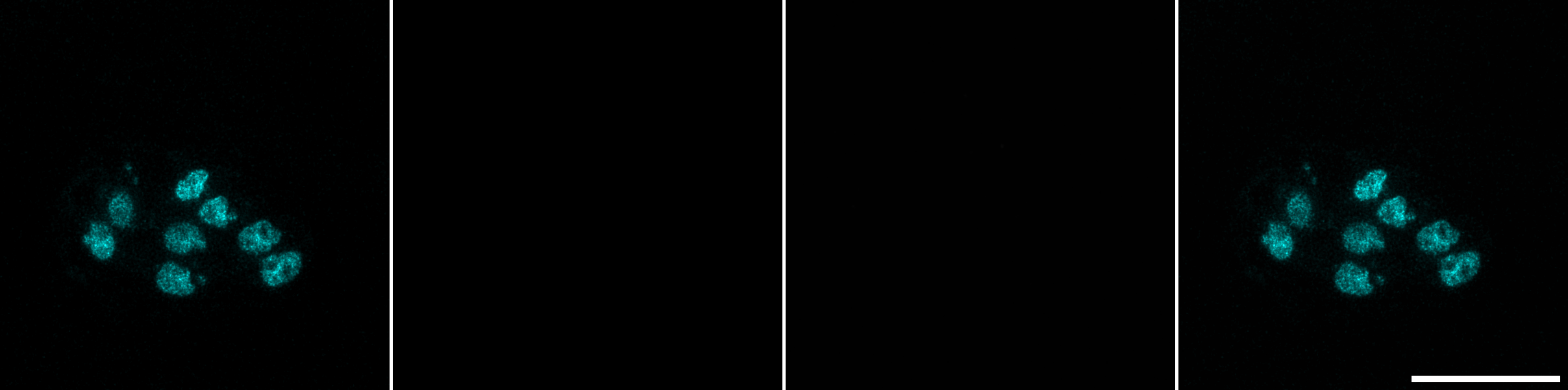

Supplement: Supplementary file 15 — Source data [file 41467_2025_58876_MOESM15_ESM.zip › Source suppl/Supplementary Figure 3_Source Data/Suppl Fig3e/FT_230201_Ku80_HA488_GRA3_647_150x_A_3_MMStack_Pos0.ome_crop-0003_MONTAGE_SCALE.tif]

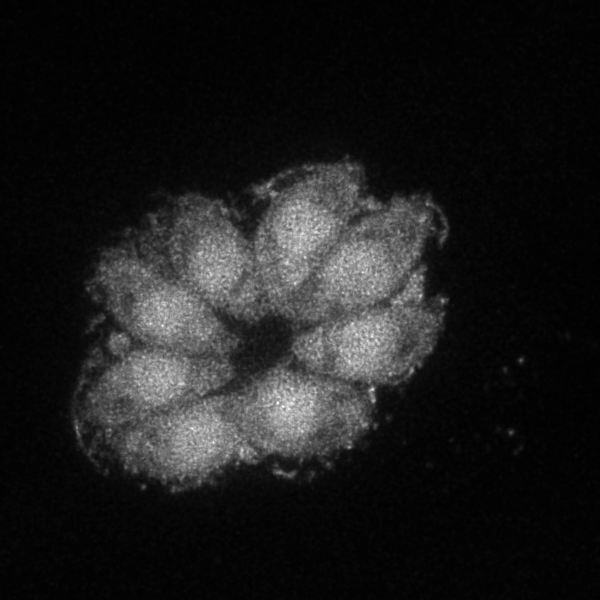

Supplement: Supplementary file 15 — Source data [file 41467_2025_58876_MOESM15_ESM.zip › Source suppl/Supplementary Figure 3_Source Data/Suppl Fig3e/FT_230201_dGRA12_HA488_GRA2647_150x_F_8_MMStack_Pos0.ome_crop-0004.tif]

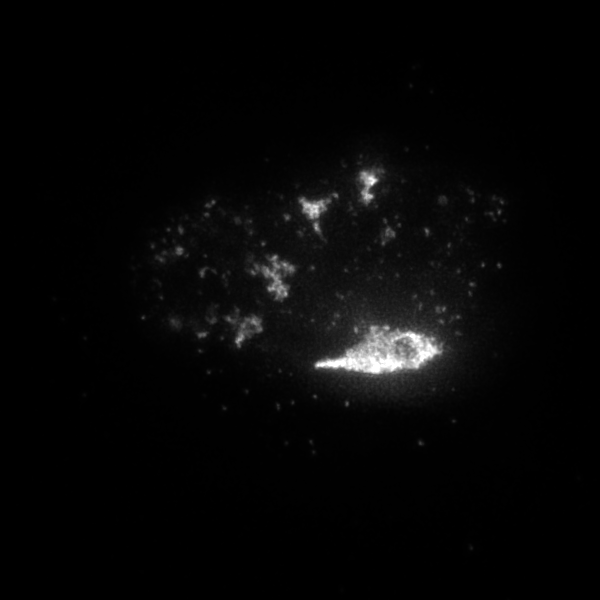

Supplement: Supplementary file 15 — Source data [file 41467_2025_58876_MOESM15_ESM.zip › Source suppl/Supplementary Figure 3_Source Data/Suppl Fig3e/FT_230206_dGRA12_HA488_GRA3_647_150x_B_8_MMStack_Pos0_crop.ome.tif]

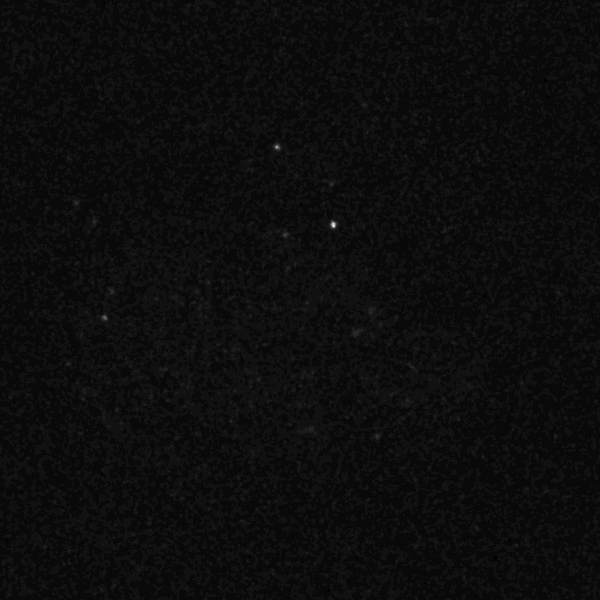

Supplement: Supplementary file 15 — Source data [file 41467_2025_58876_MOESM15_ESM.zip › Source suppl/Supplementary Figure 3_Source Data/Suppl Fig3e/FT_230201_Ku80_HA488_GRA3_647_150x_A_3_MMStack_Pos0.ome_crop.tif]

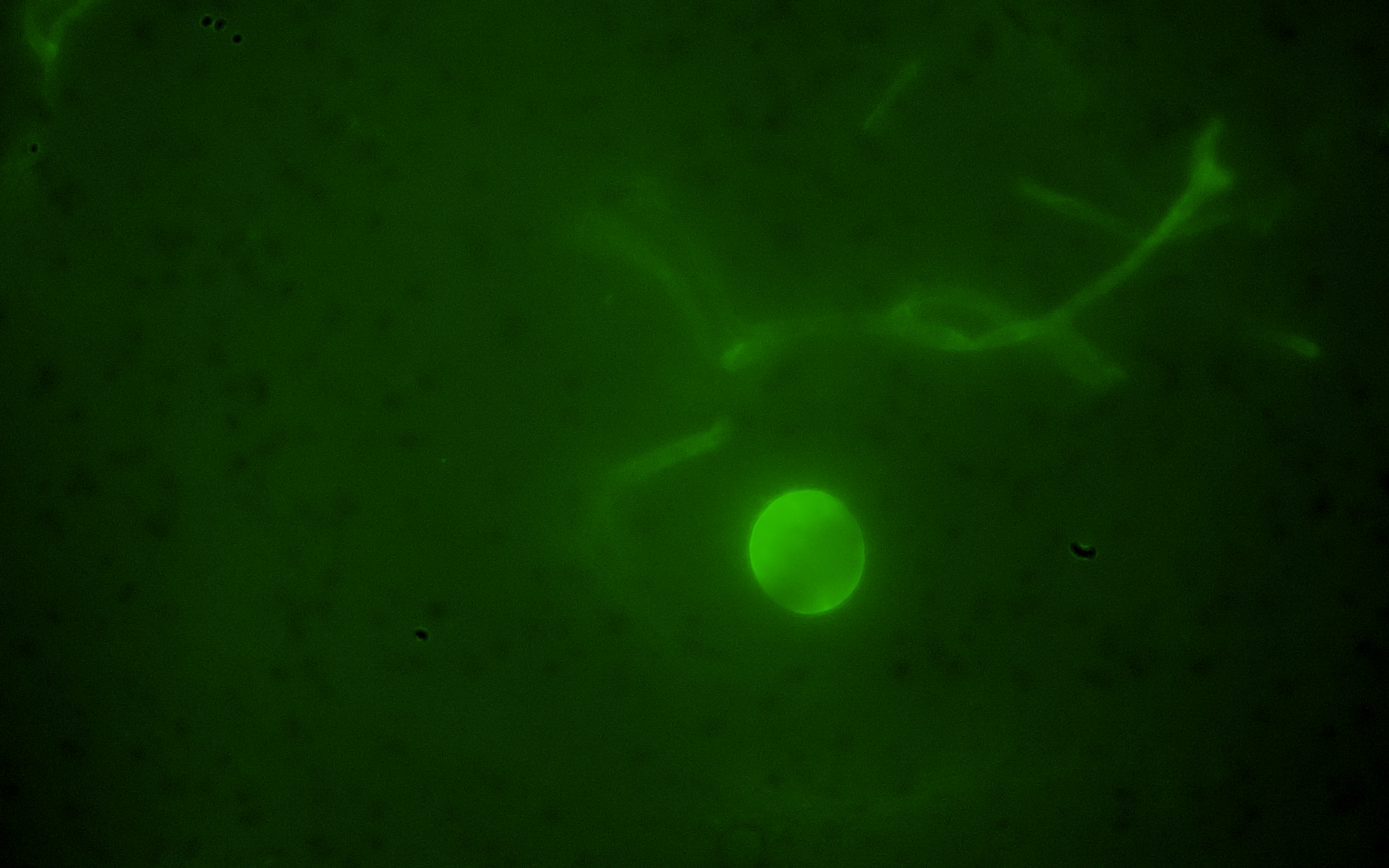

Supplement: Supplementary file 16 — Supplementary Figs. Source data [file 41467_2025_58876_MOESM16_ESM.zip › Source_mian_figures/Figure 2_Source Data/Fig 2d/FT_211118_VANDdGRA12_50000_1_DBA488_40x_B_GFP.tif]

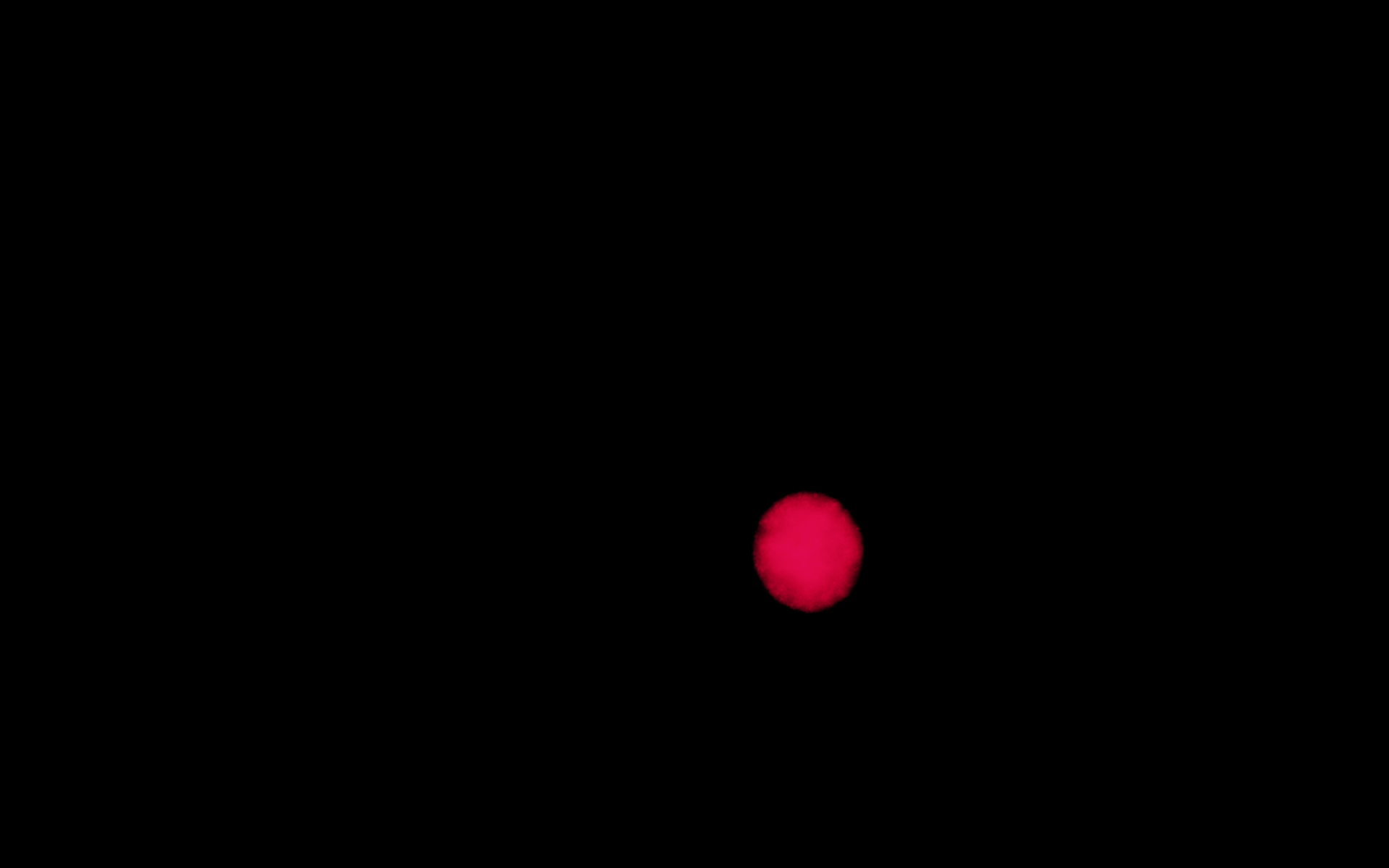

Supplement: Supplementary file 16 — Supplementary Figs. Source data [file 41467_2025_58876_MOESM16_ESM.zip › Source_mian_figures/Figure 2_Source Data/Fig 2d/FT_211118_VANDdGRA12_50000_1_DBA488_40x_B_mCh.tif]

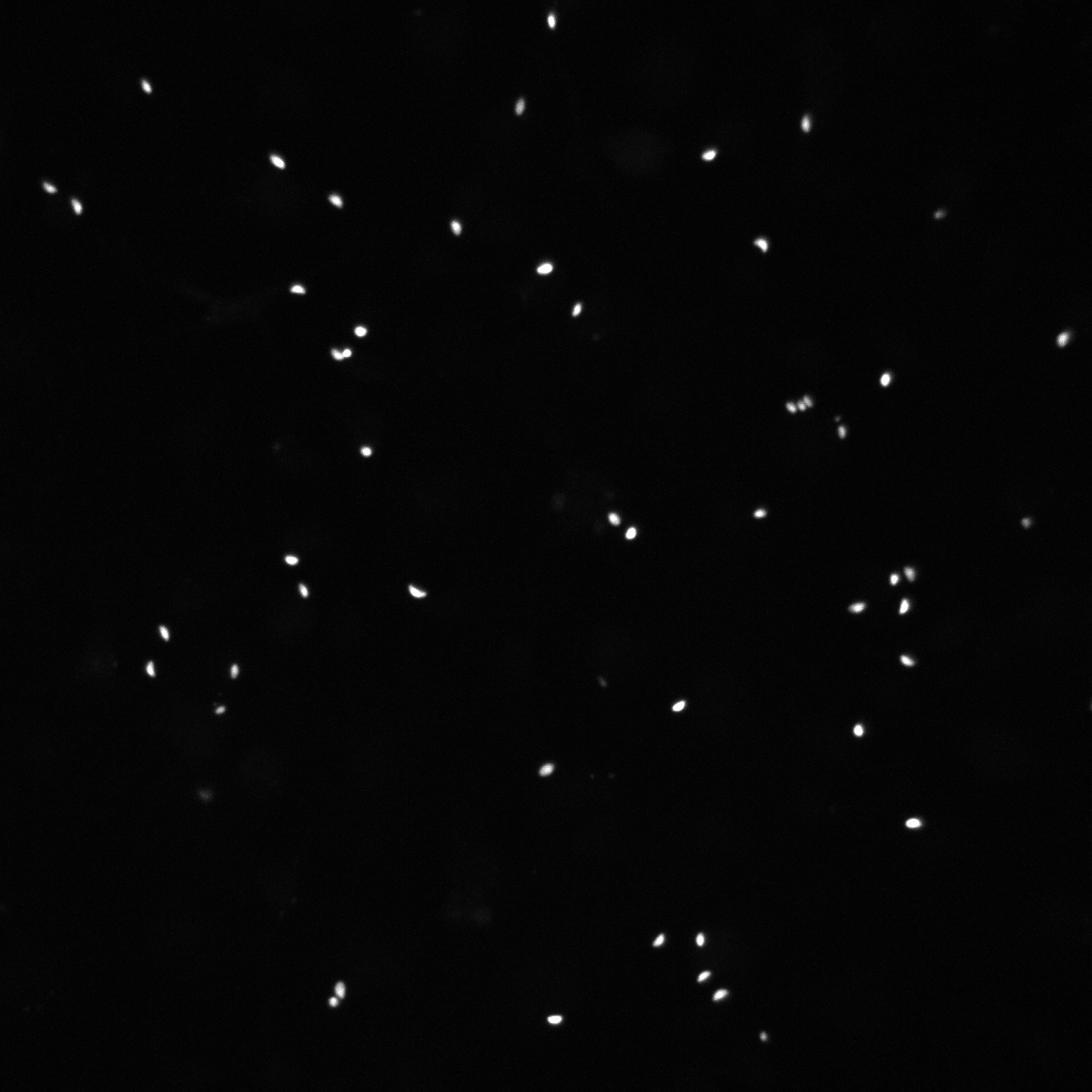

Supplement: Supplementary file 16 — Supplementary Figs. Source data [file 41467_2025_58876_MOESM16_ESM.zip › Source_mian_figures/Figure 5_Source Data/Fig 5f/FT_240927_PRU_EXP3_IRGd_IRGb10-0002_dGRA12_IRGd_A_IFNg_toxo_adj.tif]

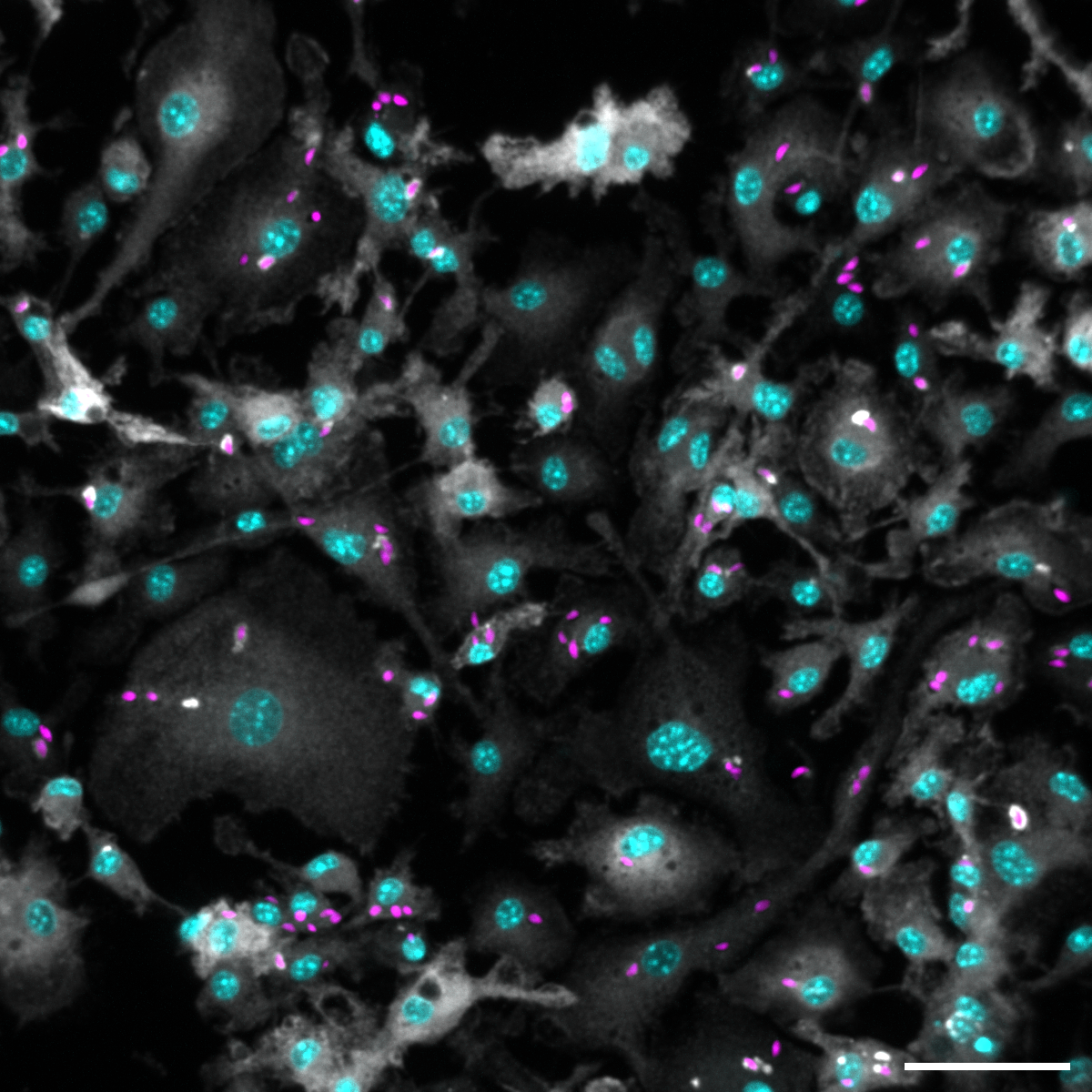

Supplement: Supplementary file 16 — Supplementary Figs. Source data [file 41467_2025_58876_MOESM16_ESM.zip › Source_mian_figures/Figure 5_Source Data/Fig 5f/FT_240927_PRU_EXP3_IRGd_IRGb10-0002_UPRT_IFNg_C_IRGd_adj_MER.tif]

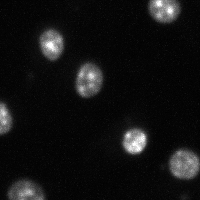

Supplement: Supplementary file 16 — Supplementary Figs. Source data [file 41467_2025_58876_MOESM16_ESM.zip › Source_mian_figures/Figure 5_Source Data/Fig 5f/FT_240927_PRU_EXP3_IRGd_IRGb10-0002_dGRA12_IRGd_A_IFNg_DAPI_crop.tif]

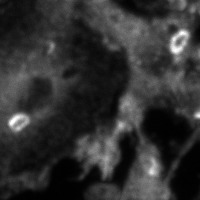

Supplement: Supplementary file 16 — Supplementary Figs. Source data [file 41467_2025_58876_MOESM16_ESM.zip › Source_mian_figures/Figure 5_Source Data/Fig 5f/FT_240927_PRU_EXP3_IRGd_IRGb10-0002_UPRT_IFNg_C_IRGd_adj_crop.tif]

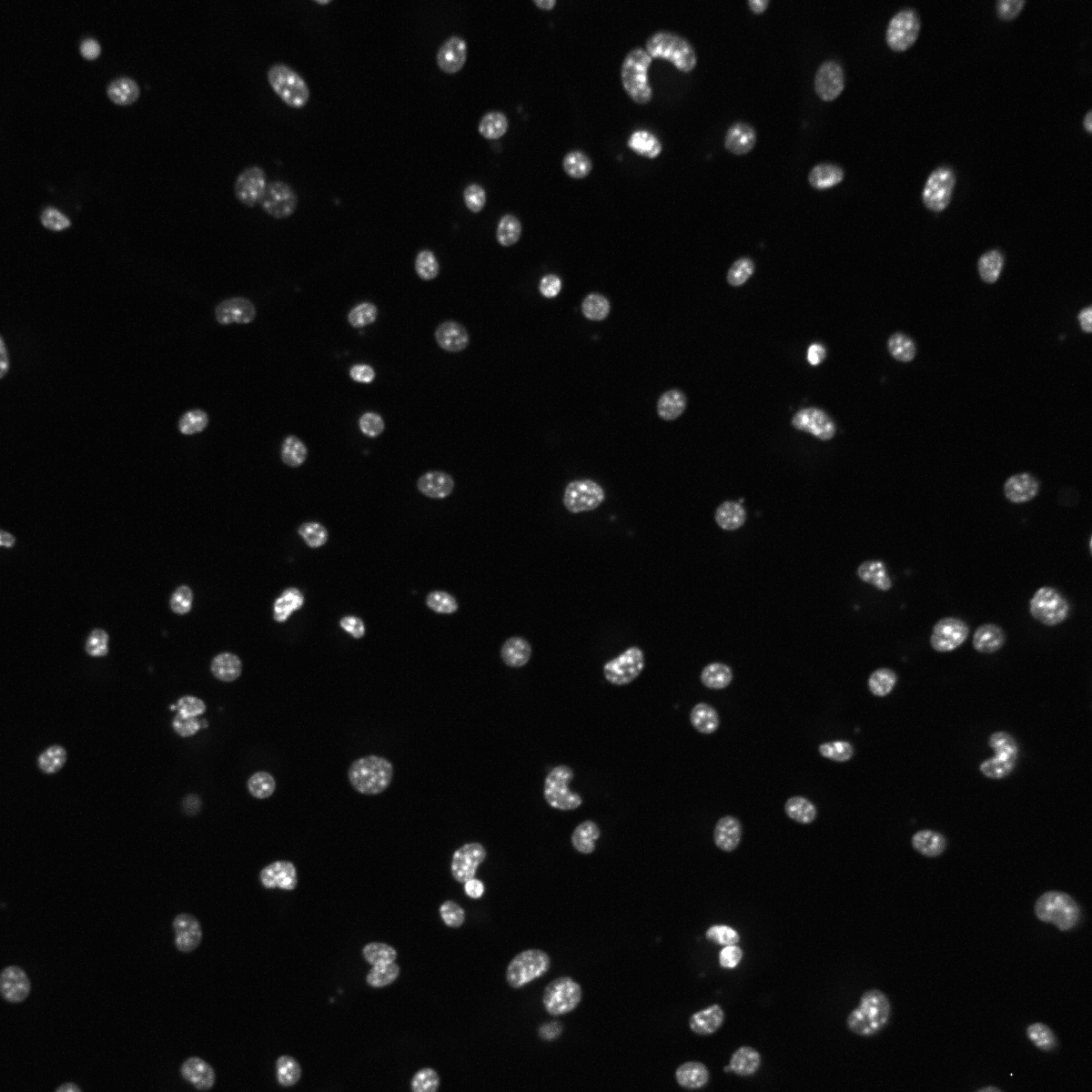

Supplement: Supplementary file 16 — Supplementary Figs. Source data [file 41467_2025_58876_MOESM16_ESM.zip › Source_mian_figures/Figure 5_Source Data/Fig 5f/FT_240927_PRU_EXP3_IRGd_IRGb10-0002_dGRA12_IRGd_A_IFNg_DAPI_adj.tif]

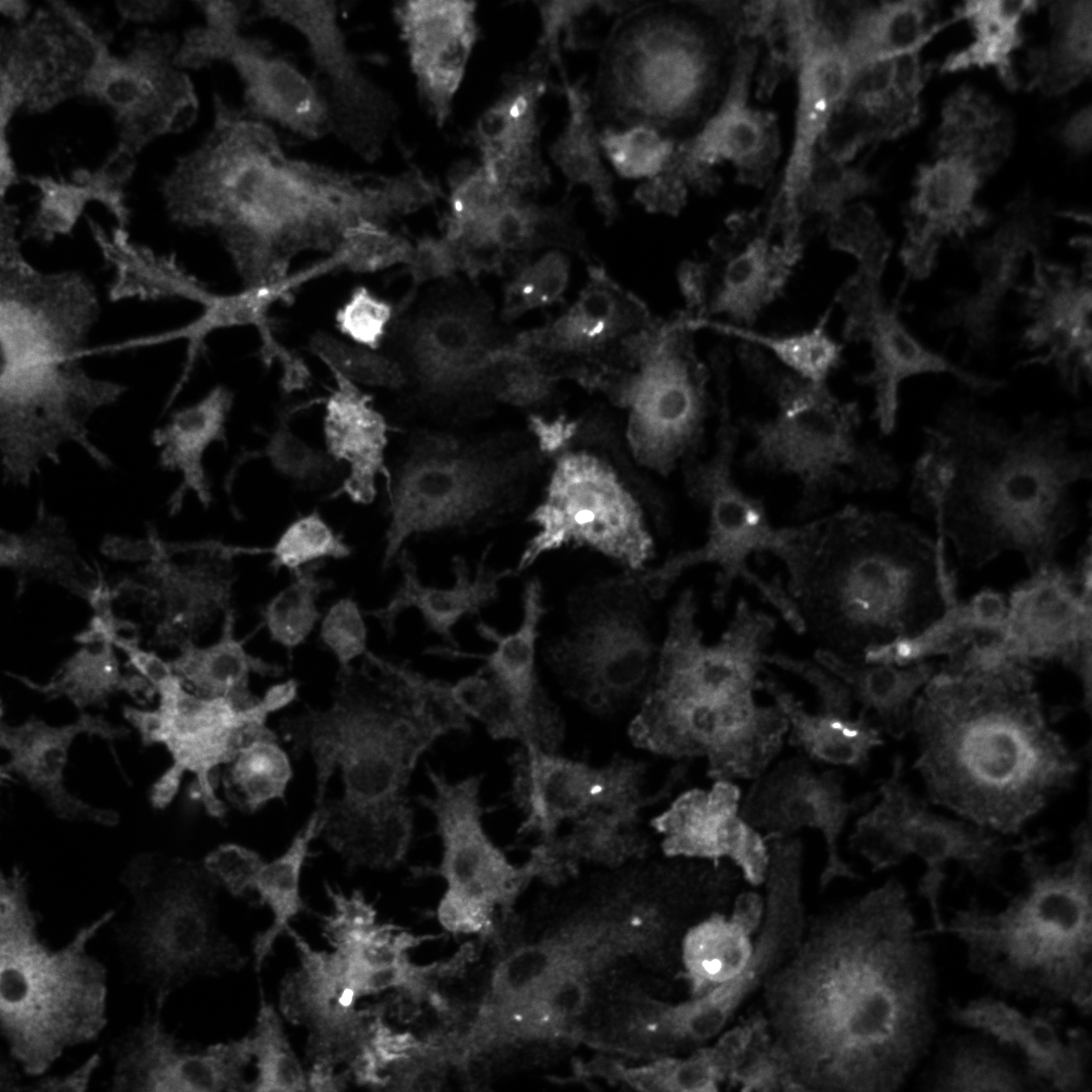

Supplement: Supplementary file 16 — Supplementary Figs. Source data [file 41467_2025_58876_MOESM16_ESM.zip › Source_mian_figures/Figure 5_Source Data/Fig 5f/FT_240927_PRU_EXP3_IRGd_IRGb10-0002_dGRA12_IRGd_A_IFNg_IRGd_adj.tif]

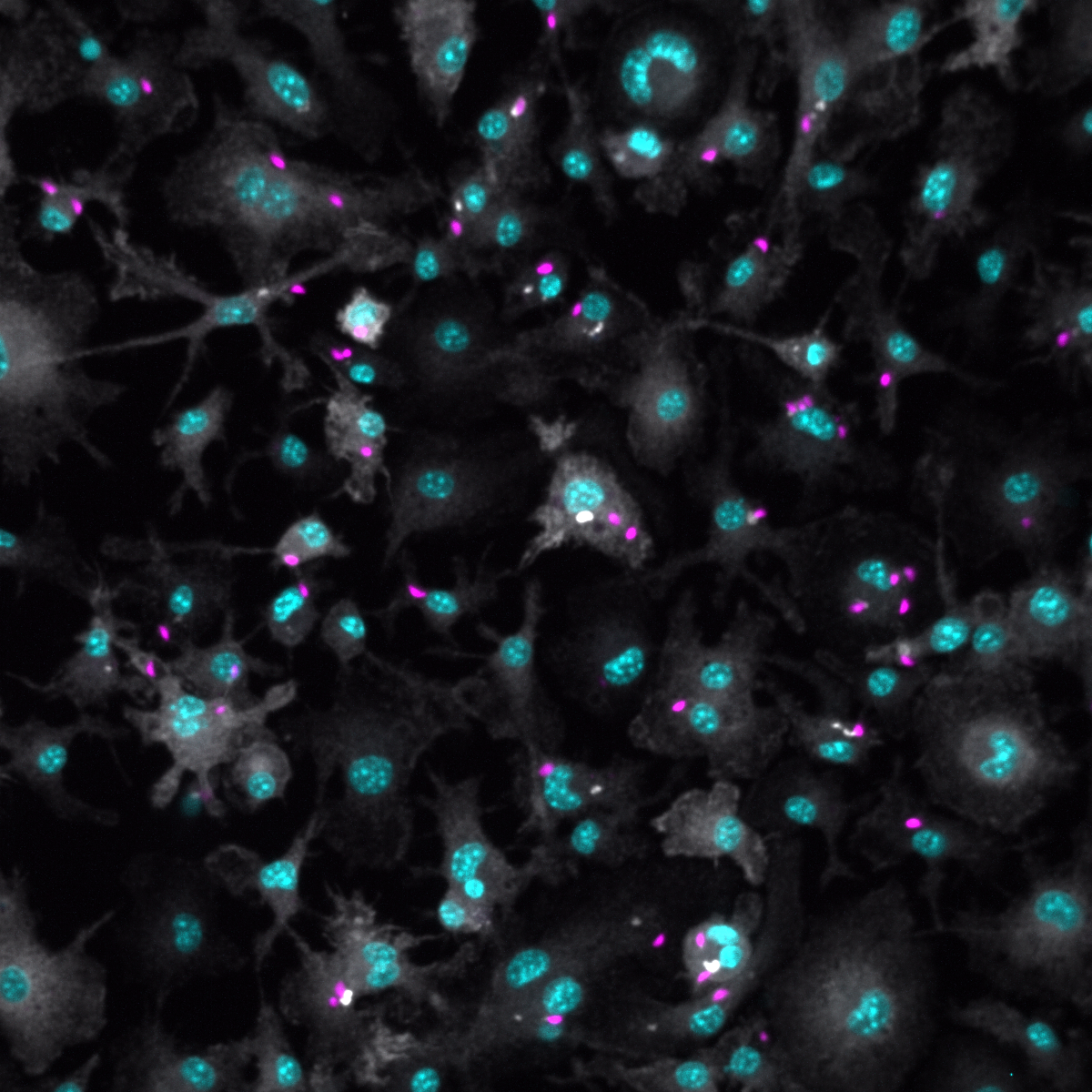

Supplement: Supplementary file 16 — Supplementary Figs. Source data [file 41467_2025_58876_MOESM16_ESM.zip › Source_mian_figures/Figure 5_Source Data/Fig 5f/FT_240927_PRU_EXP3_IRGd_IRGb10-0002_dGRA12_IRGd_A_IFNg_DAPI_adj_MERGE.tif]

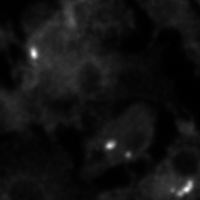

Supplement: Supplementary file 16 — Supplementary Figs. Source data [file 41467_2025_58876_MOESM16_ESM.zip › Source_mian_figures/Figure 5_Source Data/Fig 5f/FT_240927_PRU_EXP3_IRGd_IRGb10-0002_dGRA12_IRGd_A_IFNg_IRGd_crop.tif]

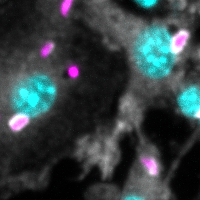

Supplement: Supplementary file 16 — Supplementary Figs. Source data [file 41467_2025_58876_MOESM16_ESM.zip › Source_mian_figures/Figure 5_Source Data/Fig 5f/FT_240927_PRU_EXP3_IRGd_IRGb10-0002_UPRT_IFNg_C_IRGd_adj_MERGE_CROP.tif]

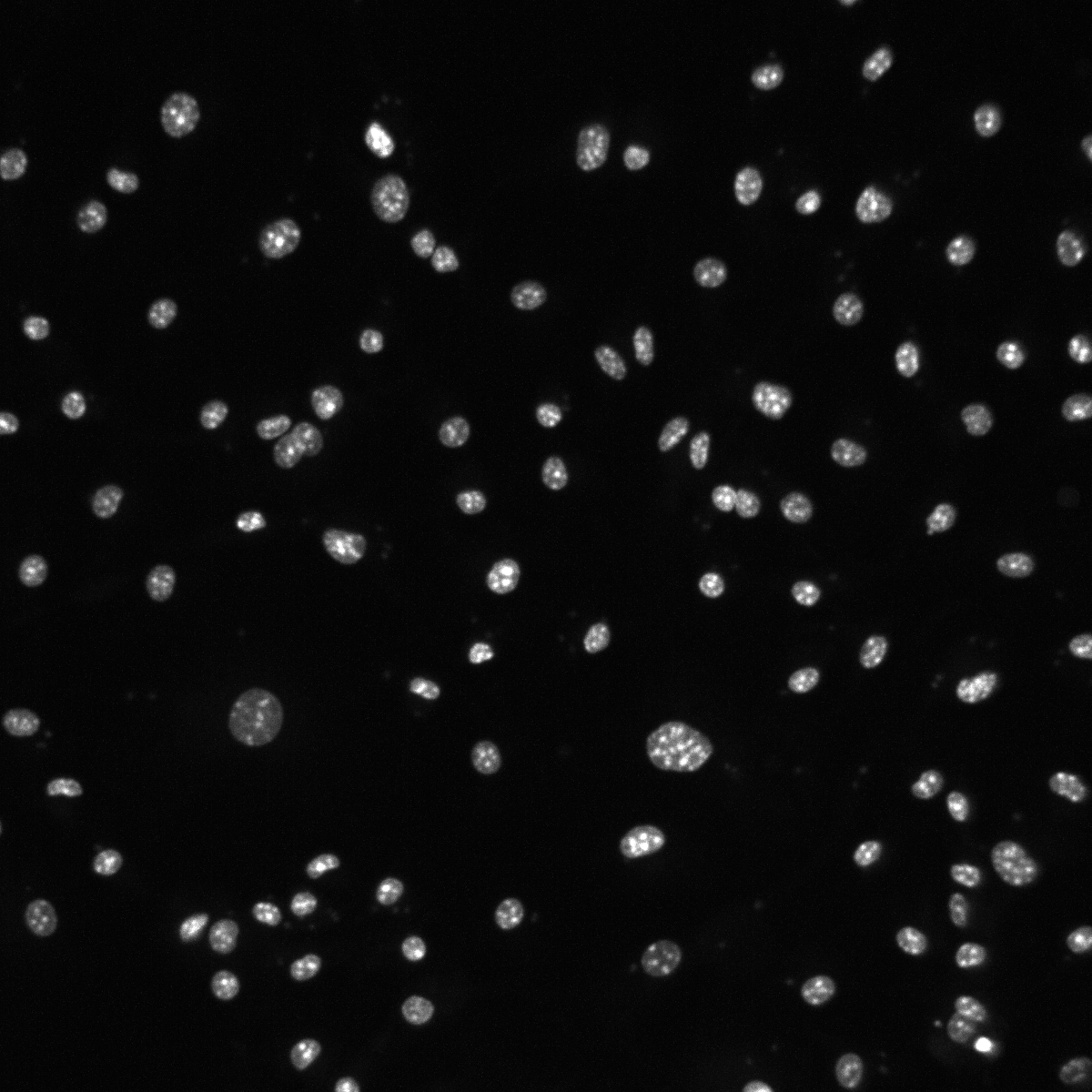

Supplement: Supplementary file 16 — Supplementary Figs. Source data [file 41467_2025_58876_MOESM16_ESM.zip › Source_mian_figures/Figure 5_Source Data/Fig 5f/FT_240927_PRU_EXP3_IRGd_IRGb10-0002_UPRT_IFNg_C_DAPI_adj.tif]

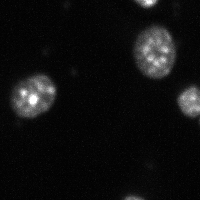

Supplement: Supplementary file 16 — Supplementary Figs. Source data [file 41467_2025_58876_MOESM16_ESM.zip › Source_mian_figures/Figure 5_Source Data/Fig 5f/FT_240927_PRU_EXP3_IRGd_IRGb10-0002_UPRT_IFNg_C_DAPI_adj_crop.tif]

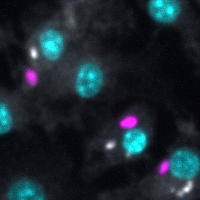

Supplement: Supplementary file 16 — Supplementary Figs. Source data [file 41467_2025_58876_MOESM16_ESM.zip › Source_mian_figures/Figure 5_Source Data/Fig 5f/FT_240927_PRU_EXP3_IRGd_IRGb10-0002_dGRA12_IRGd_A_IFNg_DAPI_adj_MERGE_crop.tif]

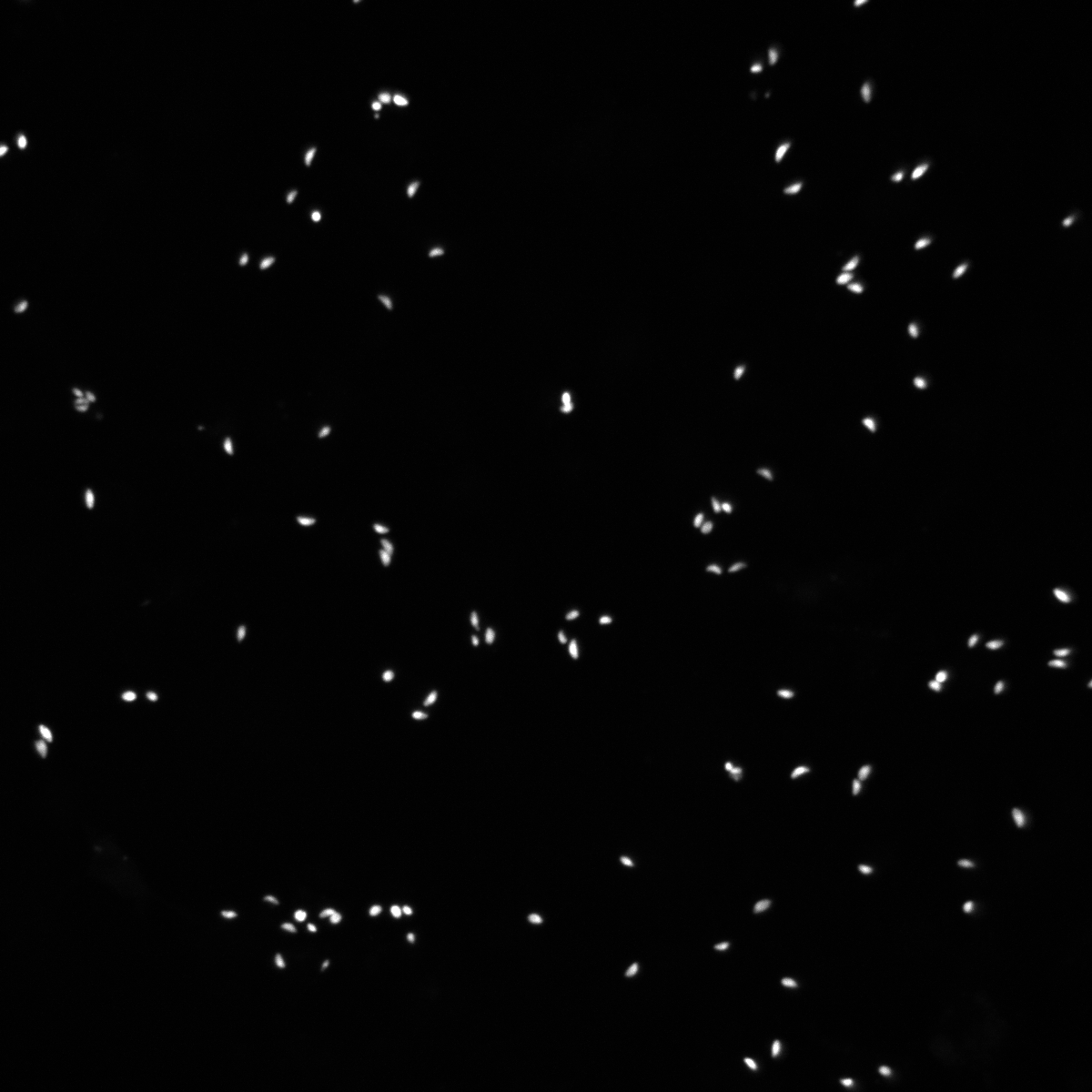

Supplement: Supplementary file 16 — Supplementary Figs. Source data [file 41467_2025_58876_MOESM16_ESM.zip › Source_mian_figures/Figure 5_Source Data/Fig 5f/FT_240927_PRU_EXP3_IRGd_IRGb10-0002_UPRT_IFNg_C_toxo_adj.tif]

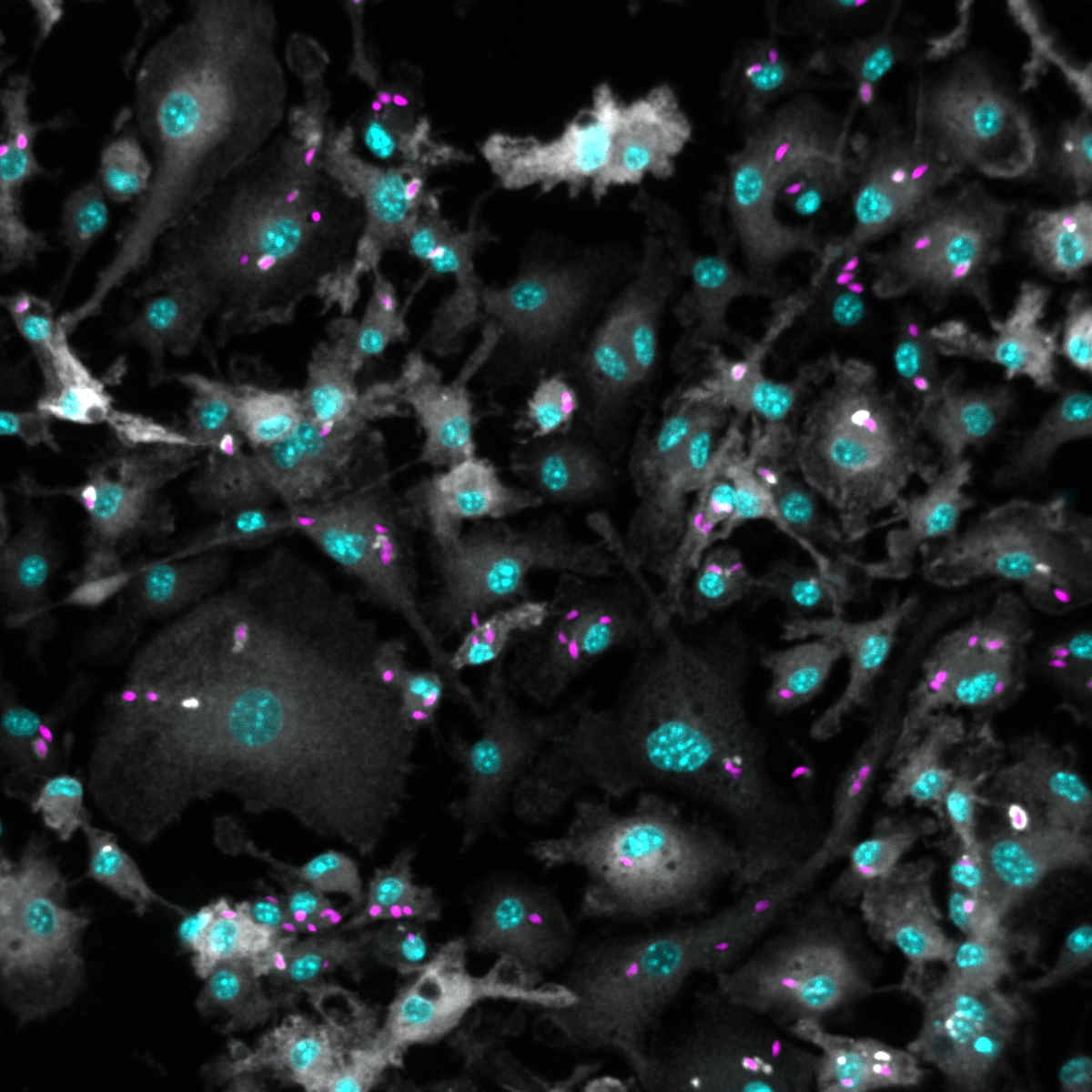

Supplement: Supplementary file 16 — Supplementary Figs. Source data [file 41467_2025_58876_MOESM16_ESM.zip › Source_mian_figures/Figure 5_Source Data/Fig 5f/FT_240927_PRU_EXP3_IRGd_IRGb10-0002_UPRT_IFNg_C_IRGd_adj_MERGE.tif]

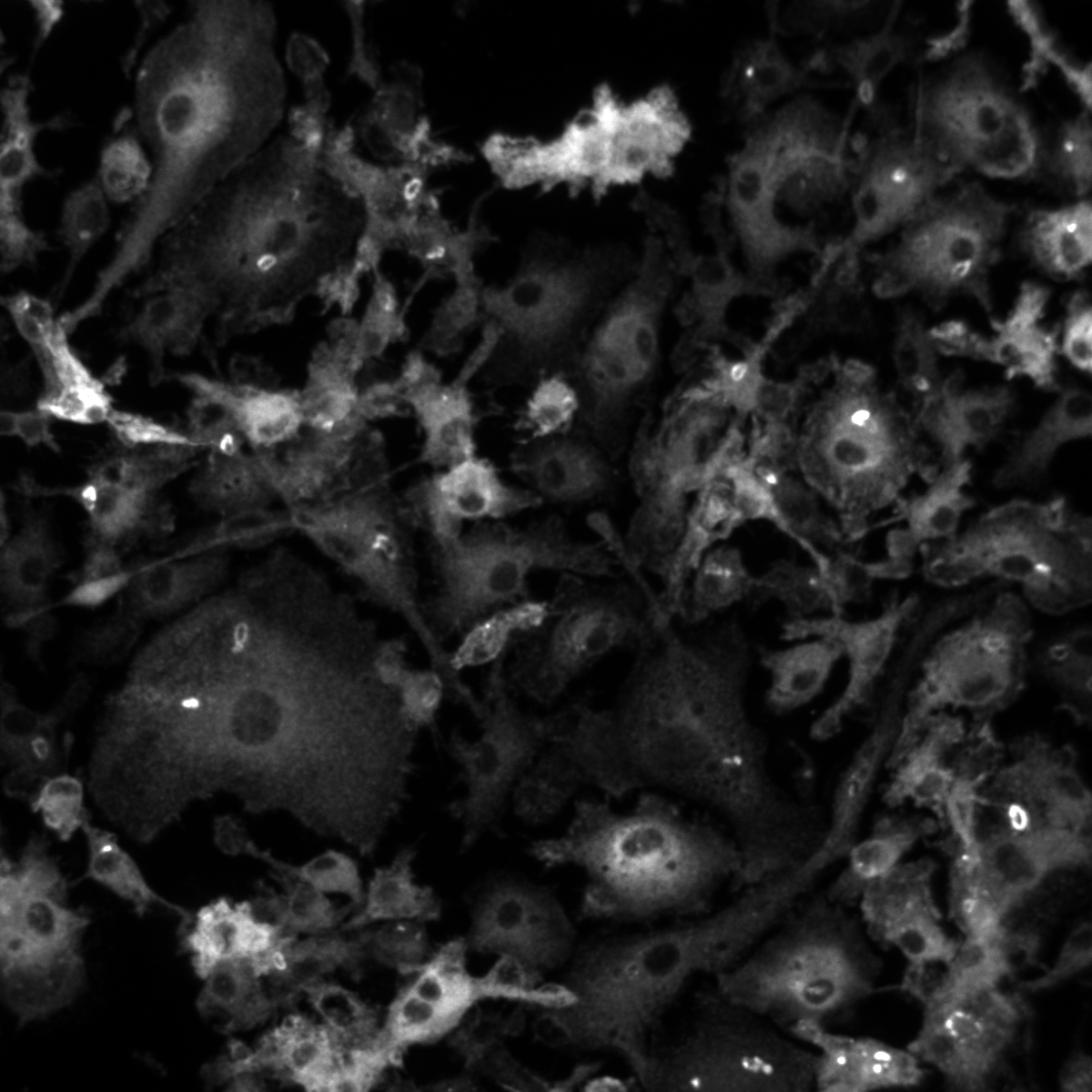

Supplement: Supplementary file 16 — Supplementary Figs. Source data [file 41467_2025_58876_MOESM16_ESM.zip › Source_mian_figures/Figure 5_Source Data/Fig 5f/FT_240927_PRU_EXP3_IRGd_IRGb10-0002_UPRT_IFNg_C_IRGd_adj.tif]

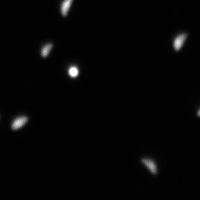

Supplement: Supplementary file 16 — Supplementary Figs. Source data [file 41467_2025_58876_MOESM16_ESM.zip › Source_mian_figures/Figure 5_Source Data/Fig 5f/FT_240927_PRU_EXP3_IRGd_IRGb10-0002_UPRT_IFNg_C_toxo_adj_crop.tif]

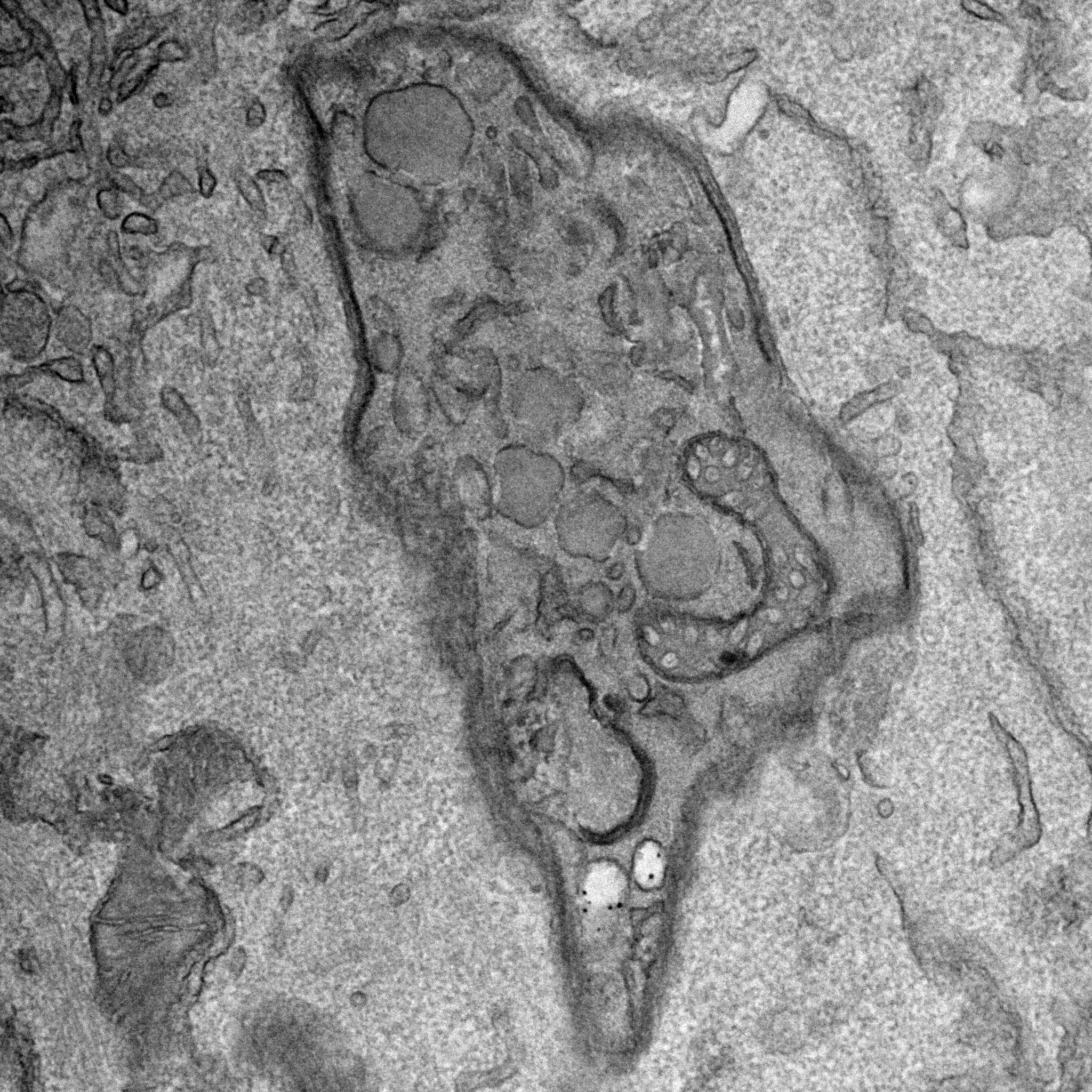

Supplement: Supplementary file 16 — Supplementary Figs. Source data [file 41467_2025_58876_MOESM16_ESM.zip › Source_mian_figures/Figure 5_Source Data/Fig 5b,c/EM04576_04_033_SA-MAG_X12k.tif]

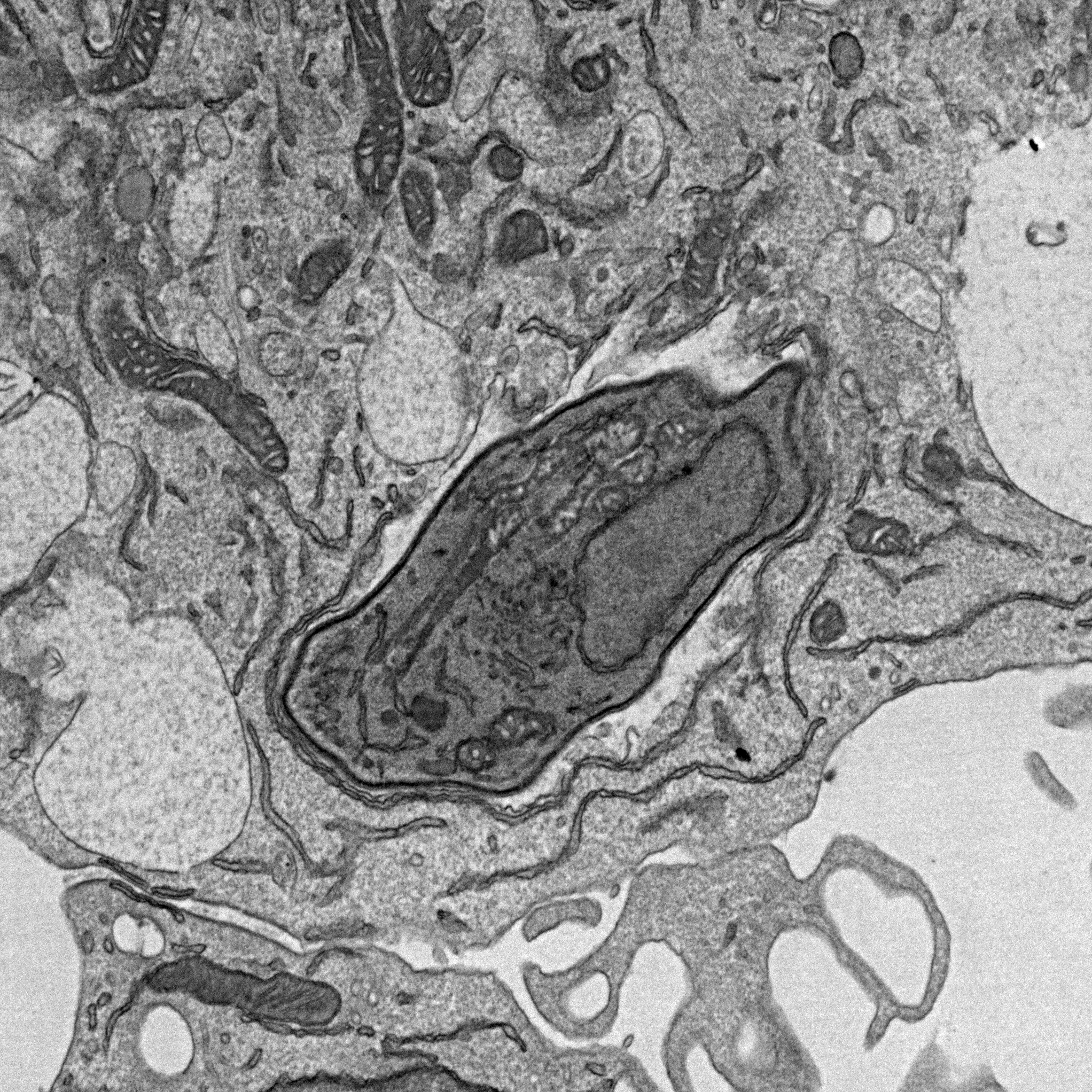

Supplement: Supplementary file 16 — Supplementary Figs. Source data [file 41467_2025_58876_MOESM16_ESM.zip › Source_mian_figures/Figure 5_Source Data/Fig 5b,c/EM04576_06_230523_007_SA-MAG_X6000.tif]

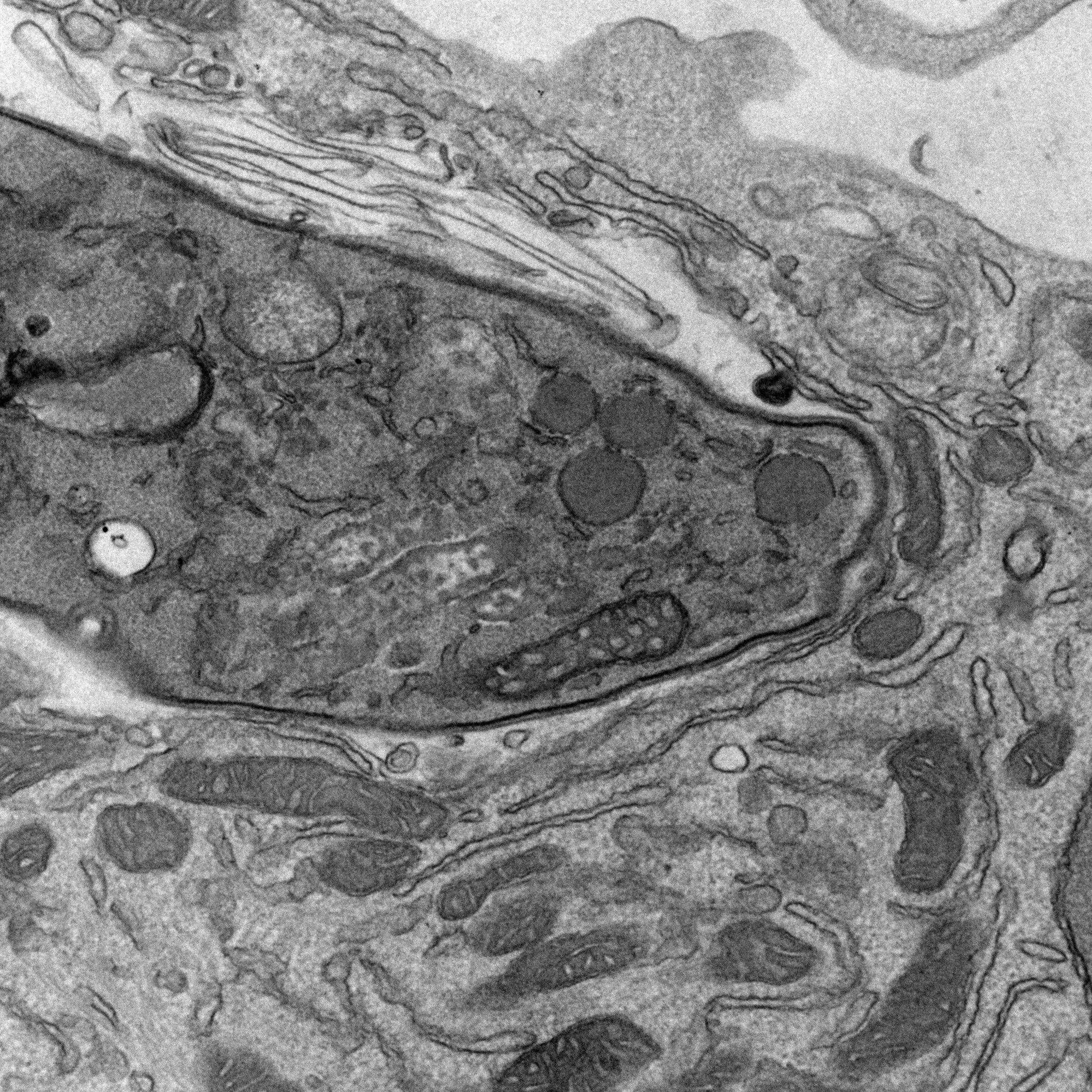

Supplement: Supplementary file 16 — Supplementary Figs. Source data [file 41467_2025_58876_MOESM16_ESM.zip › Source_mian_figures/Figure 5_Source Data/Fig 5b,c/EM04576_02_03_SA-MAG_X10k_001.tif]

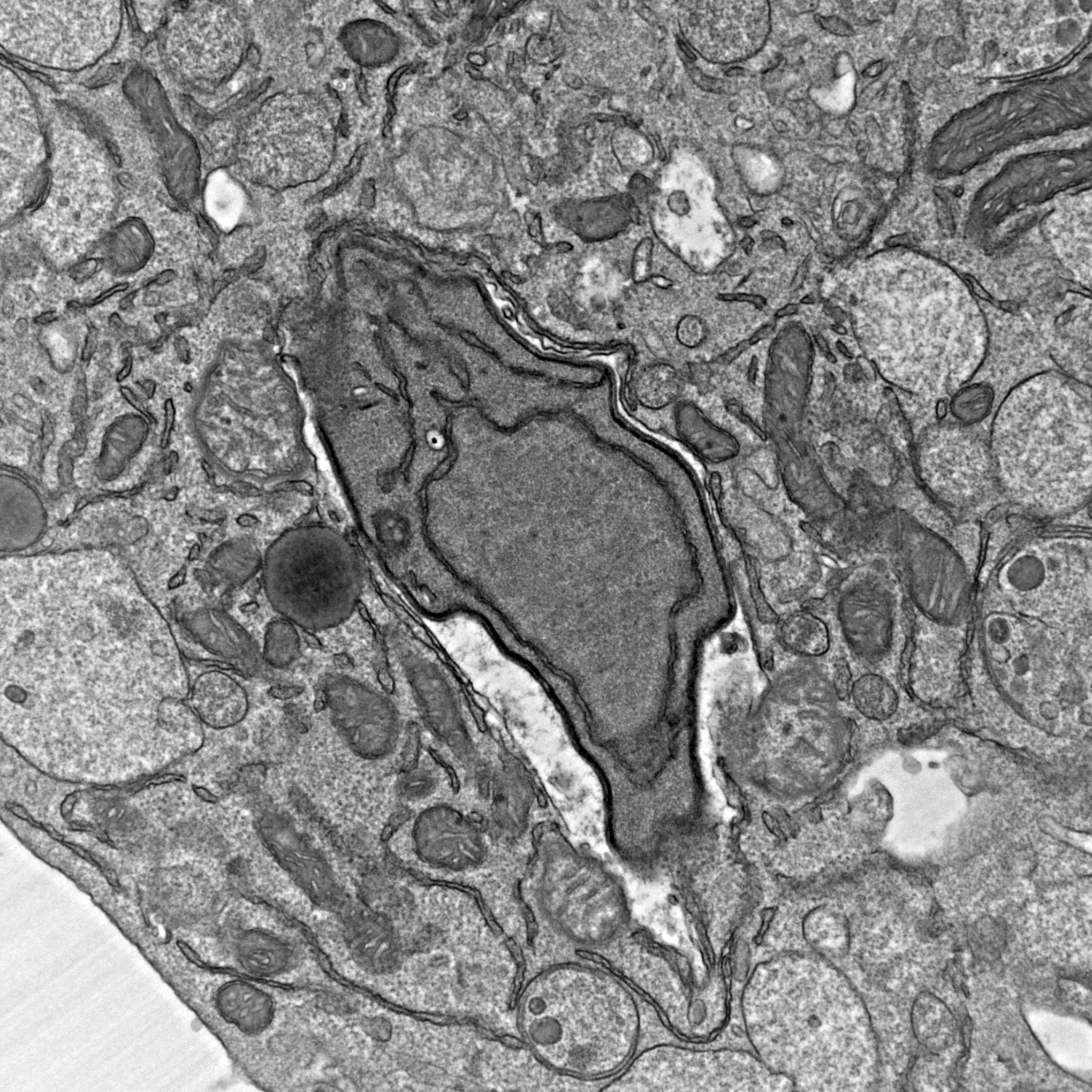

Supplement: Supplementary file 16 — Supplementary Figs. Source data [file 41467_2025_58876_MOESM16_ESM.zip › Source_mian_figures/Figure 5_Source Data/Fig 5b,c/EM04576_02_130623_001_SA-MAG_X8000.tif]

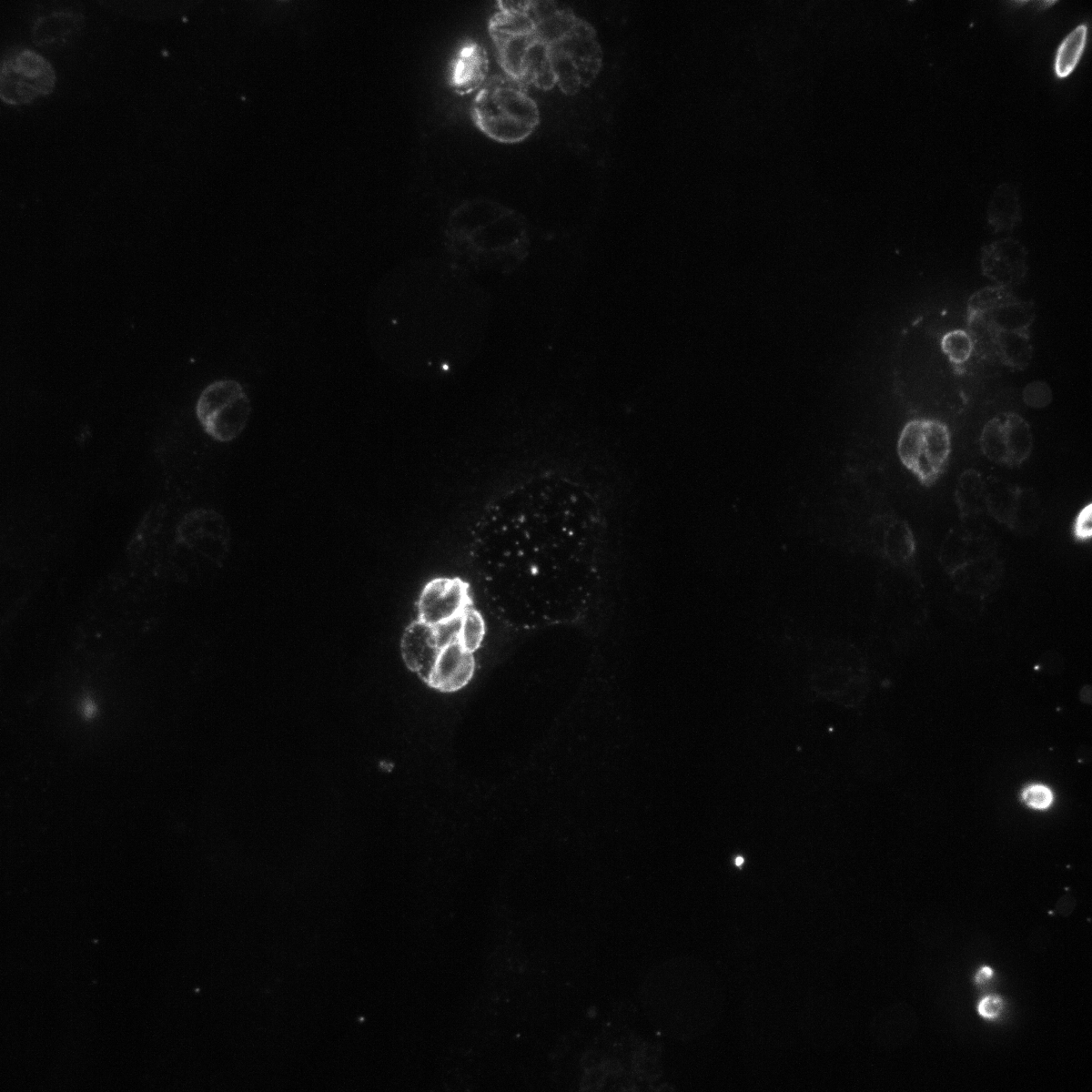

Supplement: Supplementary file 16 — Supplementary Figs. Source data [file 41467_2025_58876_MOESM16_ESM.zip › Source_mian_figures/Figure 5_Source Data/Fig 5a/FT 240226 coinf 15s 100x project C term I/MAX_FT_240226_topology-0002_toxo.tif]

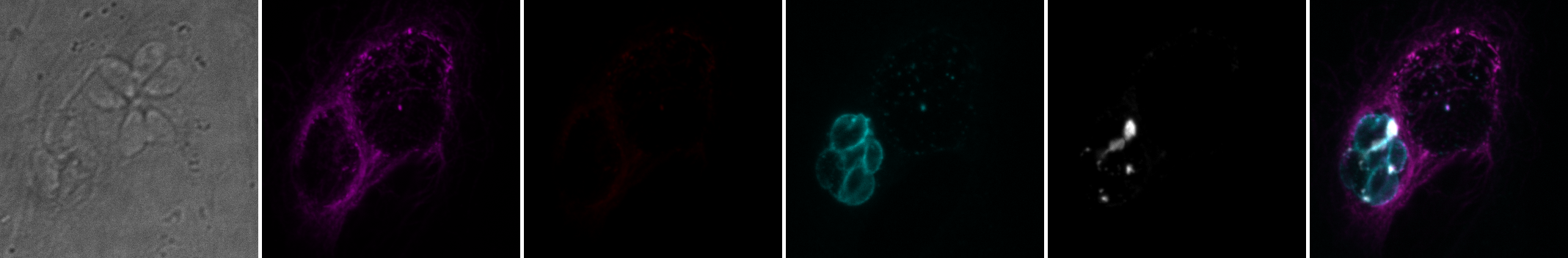

Supplement: Supplementary file 16 — Supplementary Figs. Source data [file 41467_2025_58876_MOESM16_ESM.zip › Source_mian_figures/Figure 5_Source Data/Fig 5a/FT 240226 coinf 15s 100x project C term I/Montage.tif]

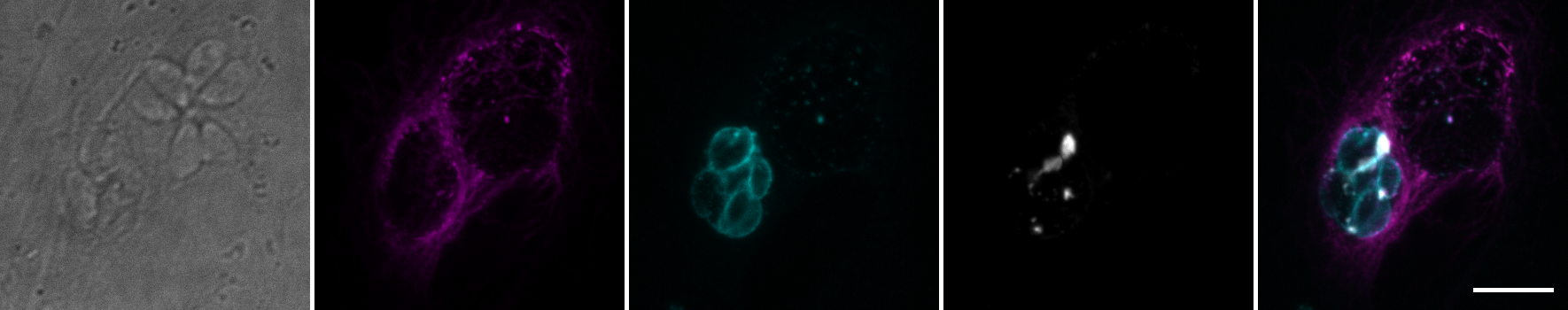

Supplement: Supplementary file 16 — Supplementary Figs. Source data [file 41467_2025_58876_MOESM16_ESM.zip › Source_mian_figures/Figure 5_Source Data/Fig 5a/FT 240226 coinf 15s 100x project C term I/Montage_scale_thin.tif]

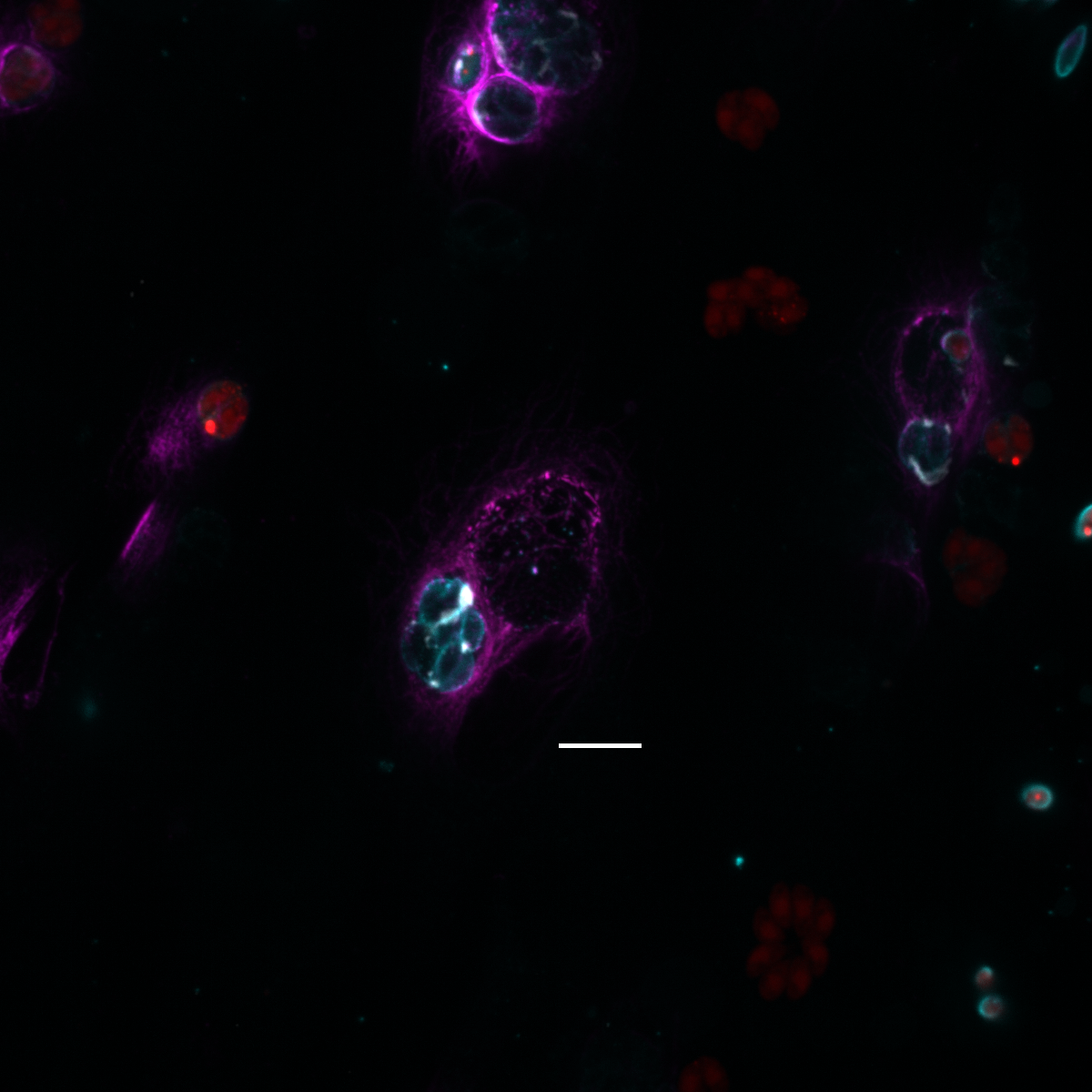

Supplement: Supplementary file 16 — Supplementary Figs. Source data [file 41467_2025_58876_MOESM16_ESM.zip › Source_mian_figures/Figure 5_Source Data/Fig 5a/FT 240226 coinf 15s 100x project C term I/merge_scale_thin.tif]

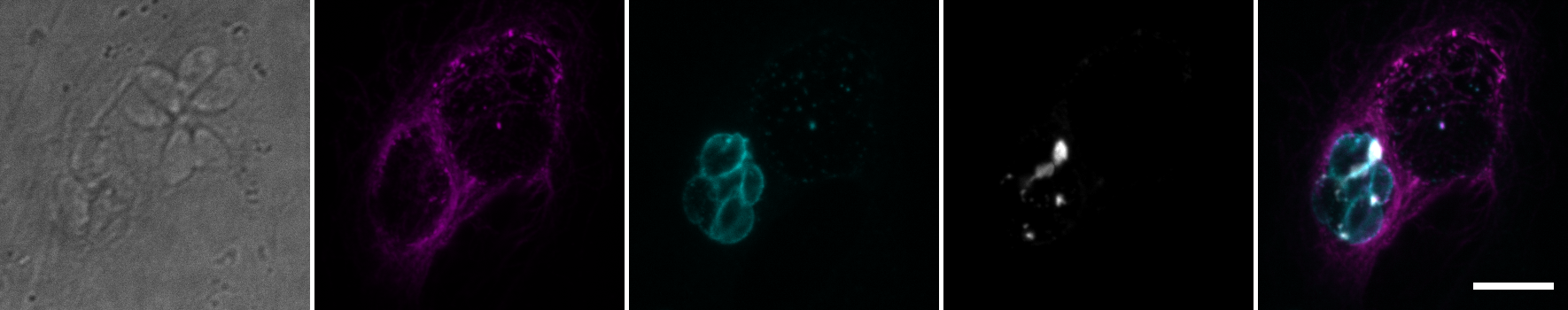

Supplement: Supplementary file 16 — Supplementary Figs. Source data [file 41467_2025_58876_MOESM16_ESM.zip › Source_mian_figures/Figure 5_Source Data/Fig 5a/FT 240226 coinf 15s 100x project C term I/Montage_SCALE.tif]

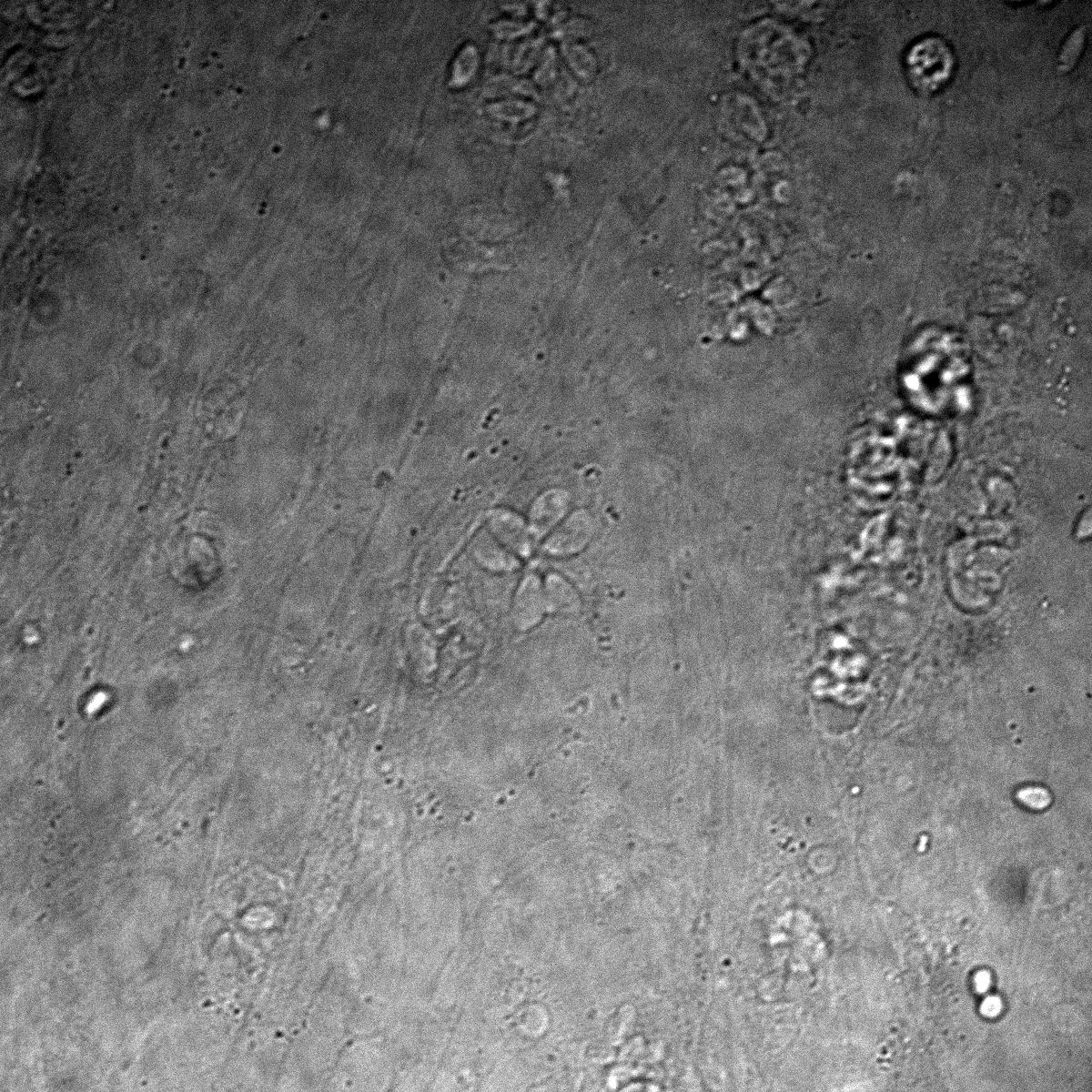

Supplement: Supplementary file 16 — Supplementary Figs. Source data [file 41467_2025_58876_MOESM16_ESM.zip › Source_mian_figures/Figure 5_Source Data/Fig 5a/FT 240226 coinf 15s 100x project C term I/MAX_FT_240226_topology-0001_BF.tif]

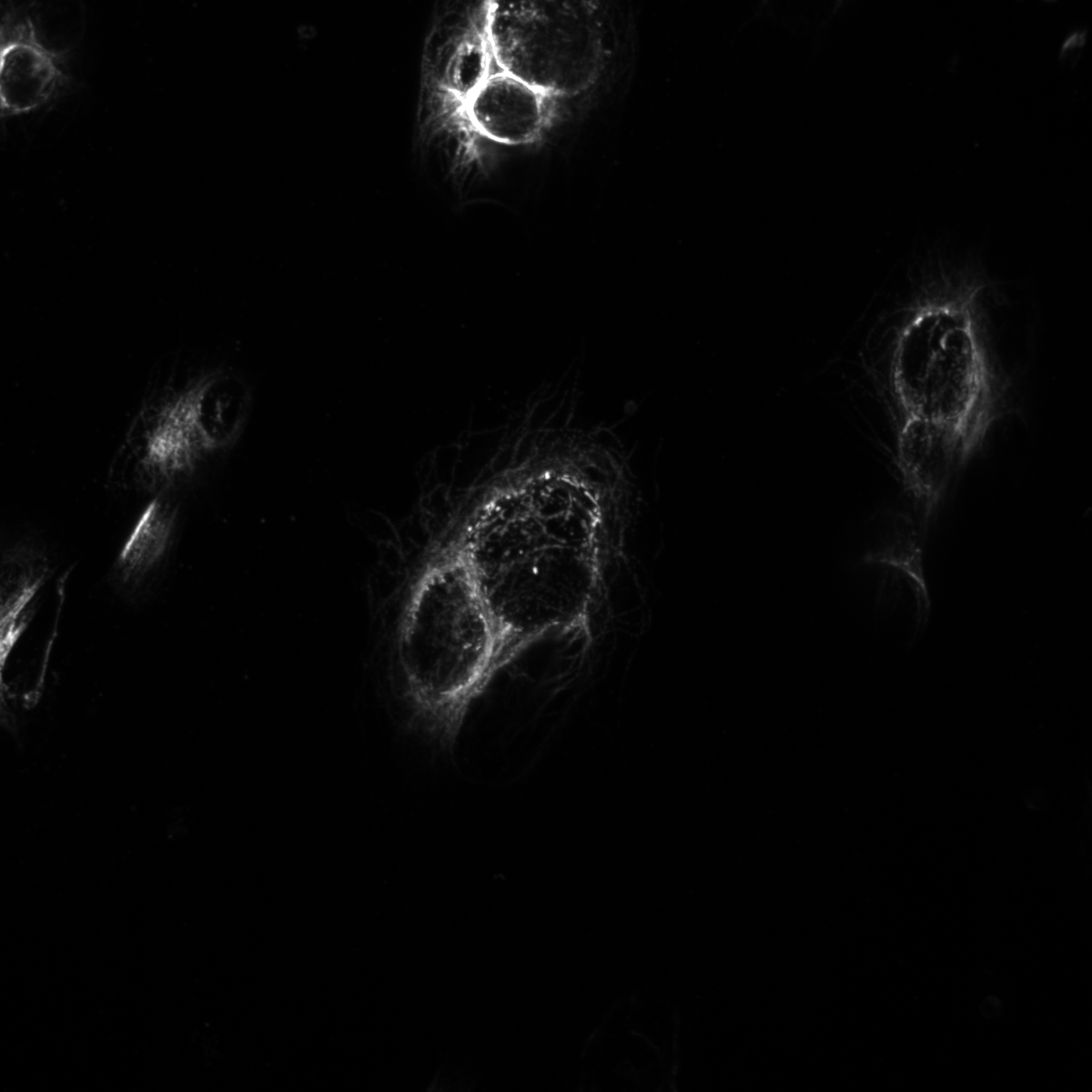

Supplement: Supplementary file 16 — Supplementary Figs. Source data [file 41467_2025_58876_MOESM16_ESM.zip › Source_mian_figures/Figure 5_Source Data/Fig 5a/FT 240226 coinf 15s 100x project C term I/MAX_FT_240226_topology-0005_tubulin.tif]

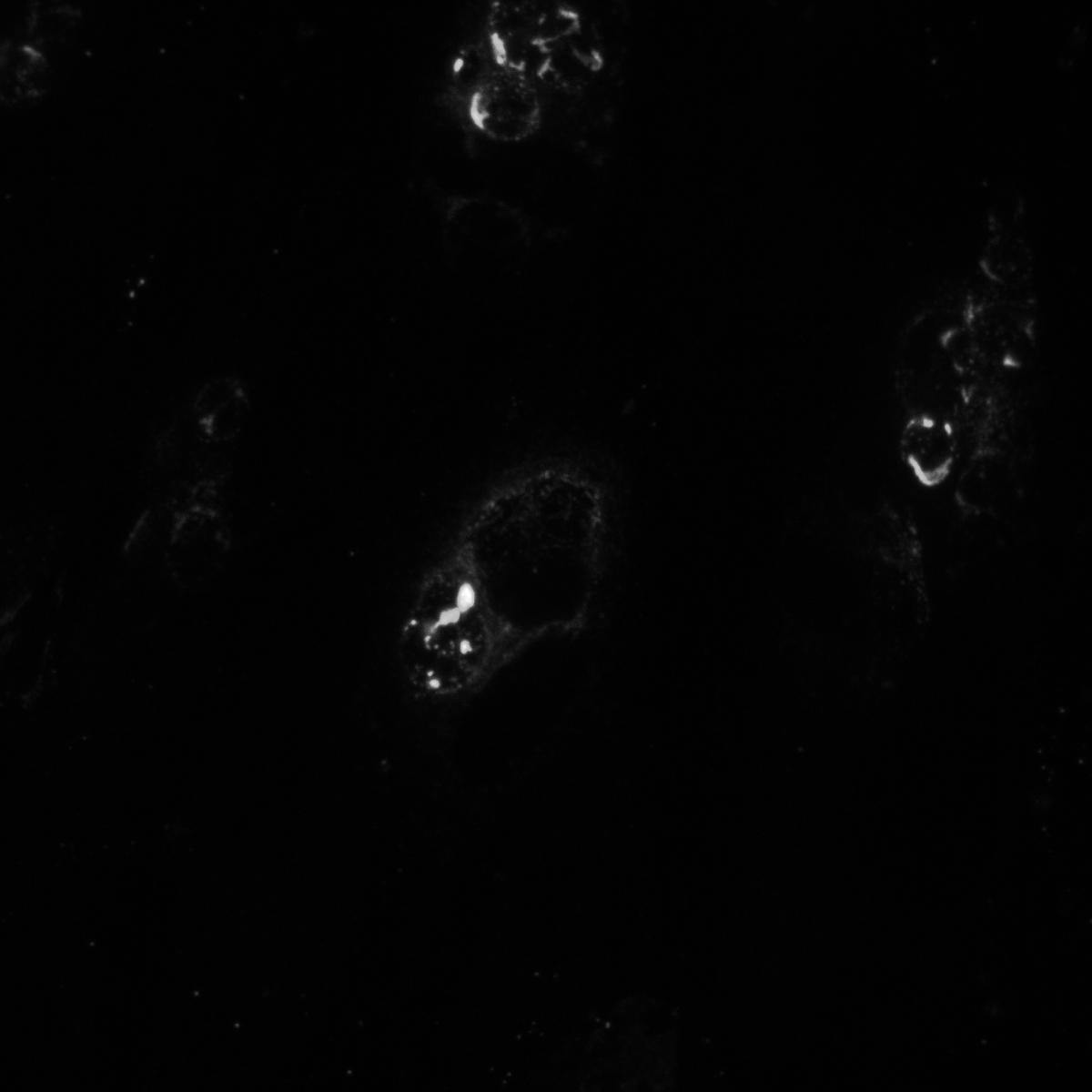

Supplement: Supplementary file 16 — Supplementary Figs. Source data [file 41467_2025_58876_MOESM16_ESM.zip › Source_mian_figures/Figure 5_Source Data/Fig 5a/FT 240226 coinf 15s 100x project C term I/MAX_FT_240226_topology-0003_HA.tif]

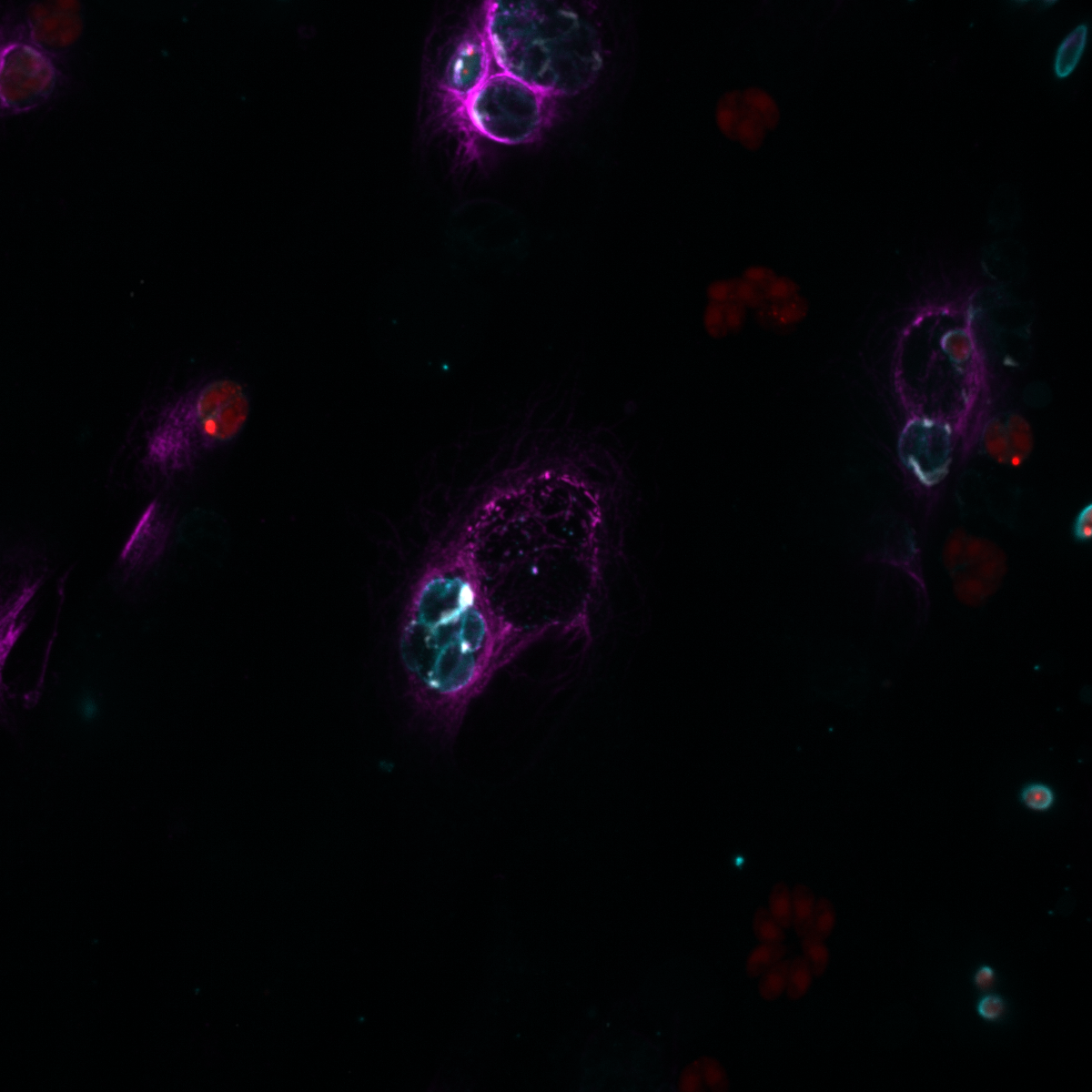

Supplement: Supplementary file 16 — Supplementary Figs. Source data [file 41467_2025_58876_MOESM16_ESM.zip › Source_mian_figures/Figure 5_Source Data/Fig 5a/FT 240226 coinf 15s 100x project C term I/merge.tif]

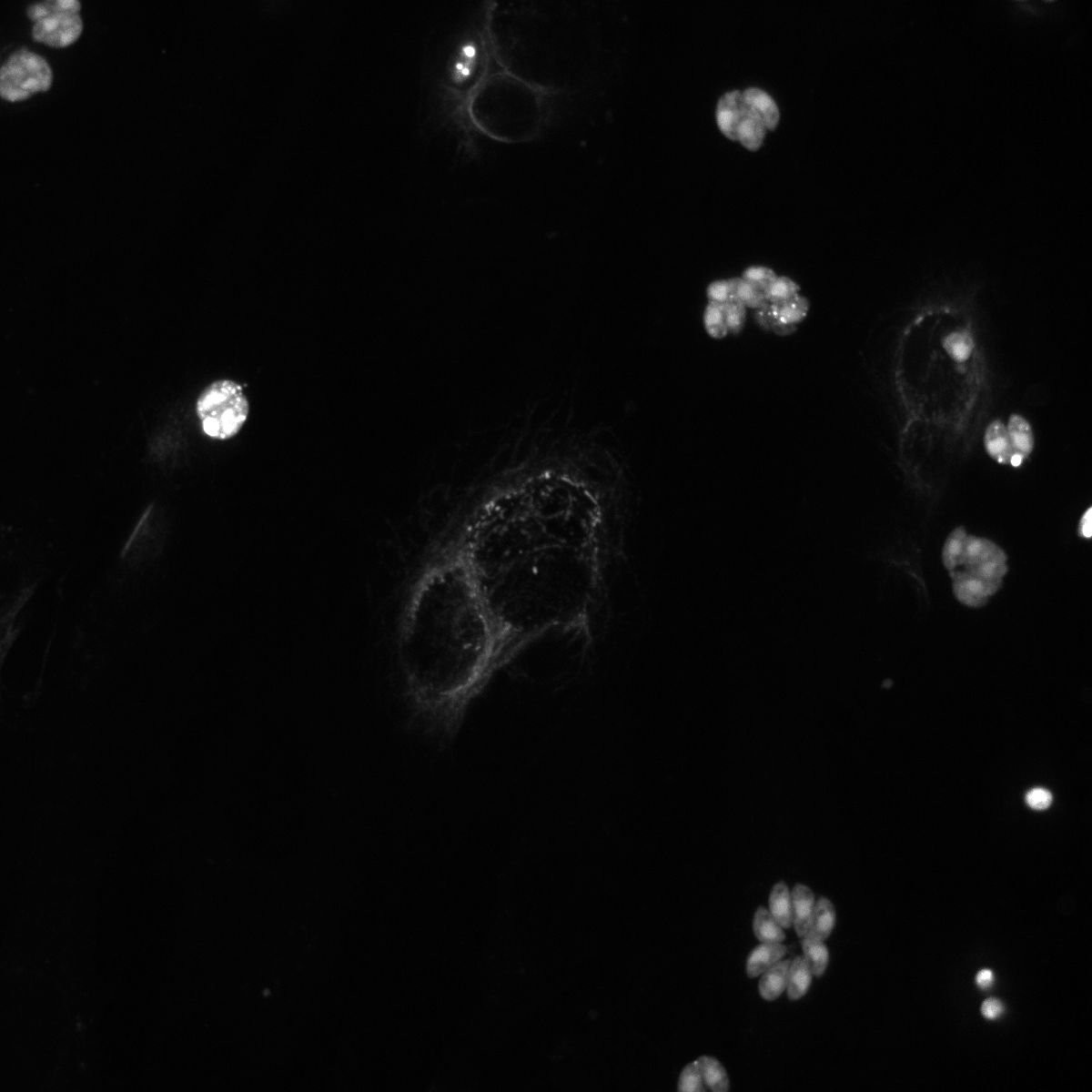

Supplement: Supplementary file 16 — Supplementary Figs. Source data [file 41467_2025_58876_MOESM16_ESM.zip › Source_mian_figures/Figure 5_Source Data/Fig 5a/FT 240226 coinf 15s 100x project C term I/MAX_FT_240226_topology-0004_mch.tif]
