# Supplementary figures and images for: GRA12 is a common virulence factor across Toxoplasma gondii strains and mouse subspecies (part 1 of 3)
Source: Nat Commun. 2025 Apr 16;16:3570. doi: 10.1038/s41467-025-58876-2 (PMC12003902; doi:10.1038/s41467-025-58876-2)

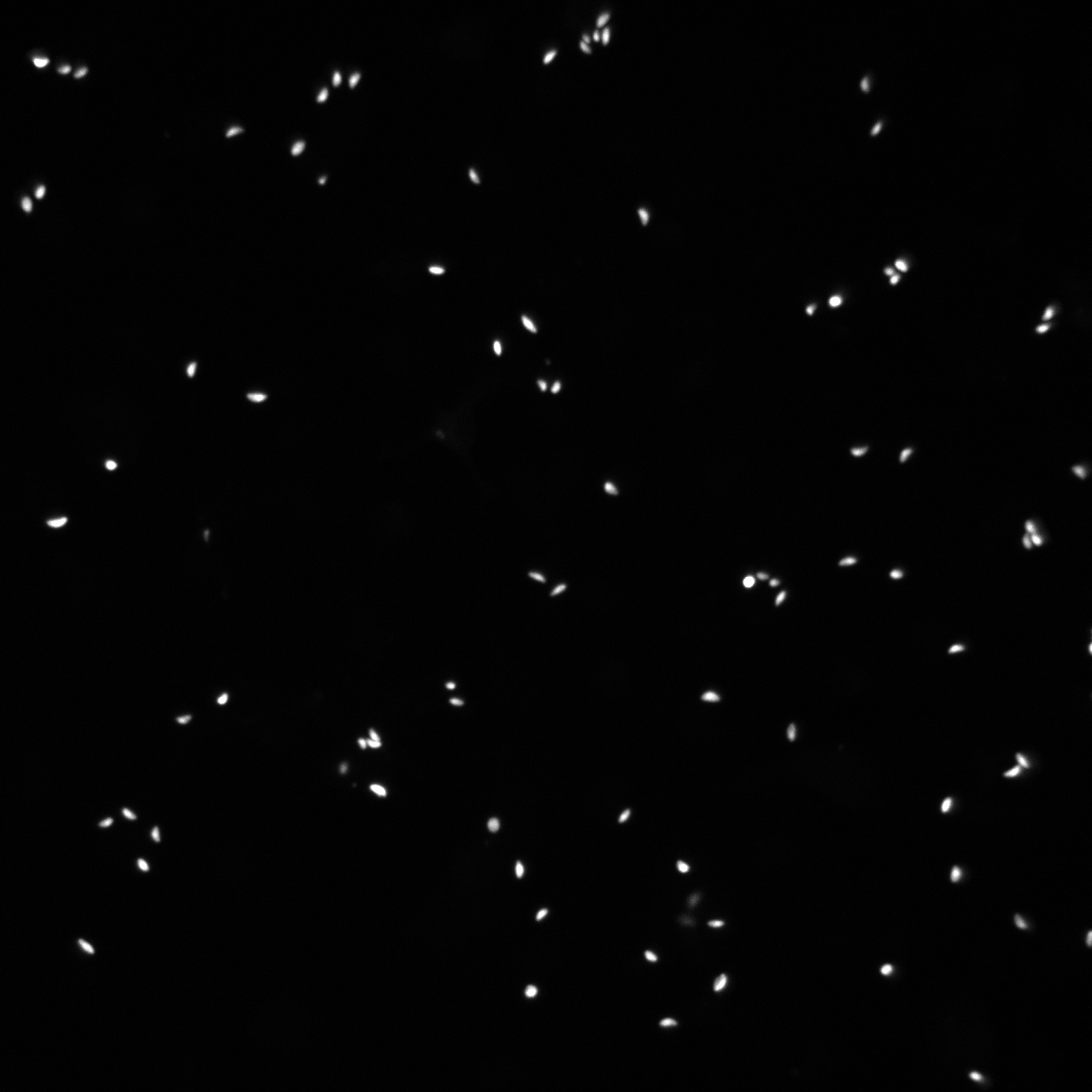

Supplement: Supplementary file 15 — Source data [file 41467_2025_58876_MOESM15_ESM.zip › Source suppl/Supplementary Figure 8_Source Data/Suppl Fig8a/FT_240927_PRU_EXP3_IRGd_IRGb10-0002_dUPRT_FK2_D_IFNg_toxo.tif]

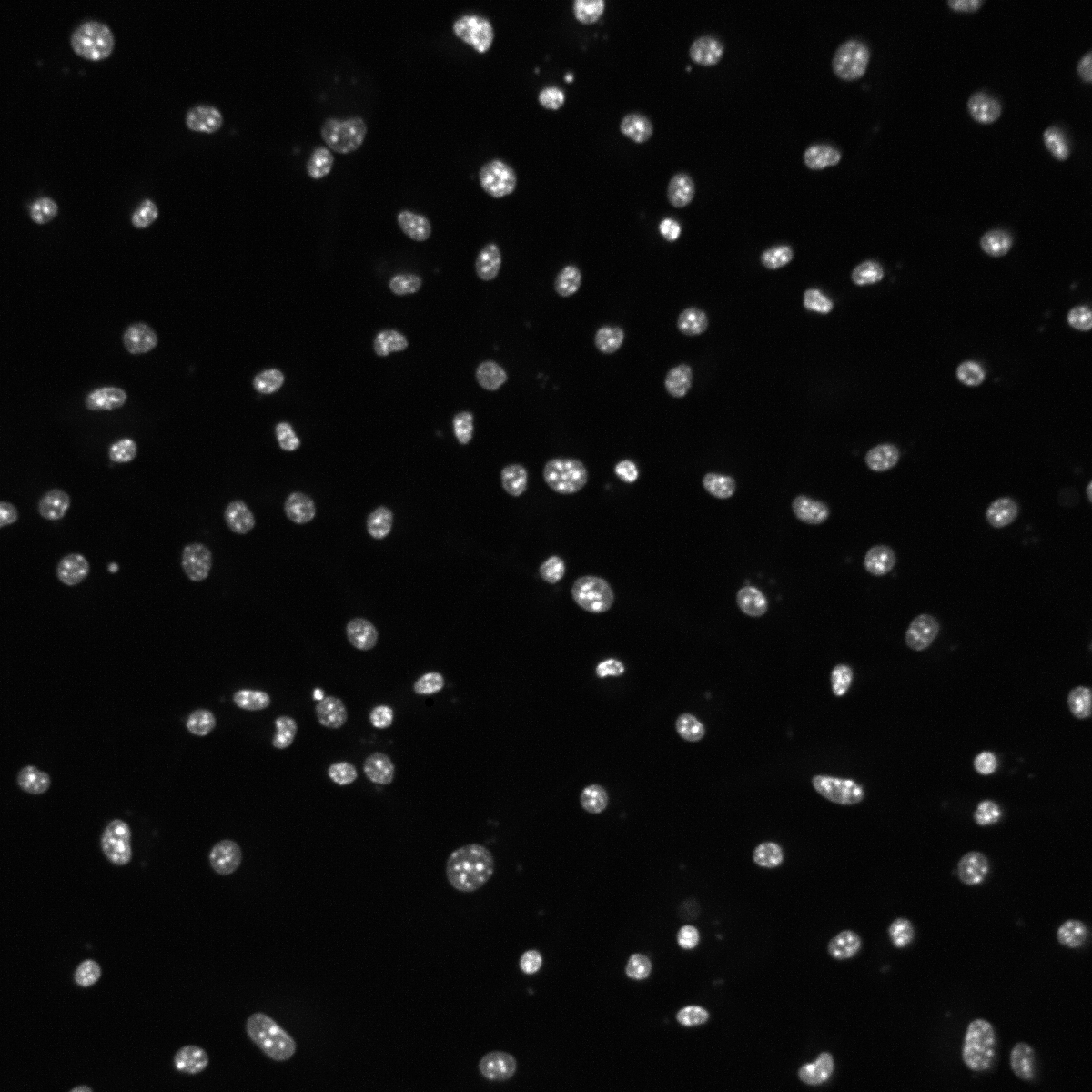

Supplement: Supplementary file 15 — Source data [file 41467_2025_58876_MOESM15_ESM.zip › Source suppl/Supplementary Figure 8_Source Data/Suppl Fig8a/FT_240927_PRU_EXP3_IRGd_IRGb10-0002_dUPRT_FK2_D_IFNg_DAPI.tif]

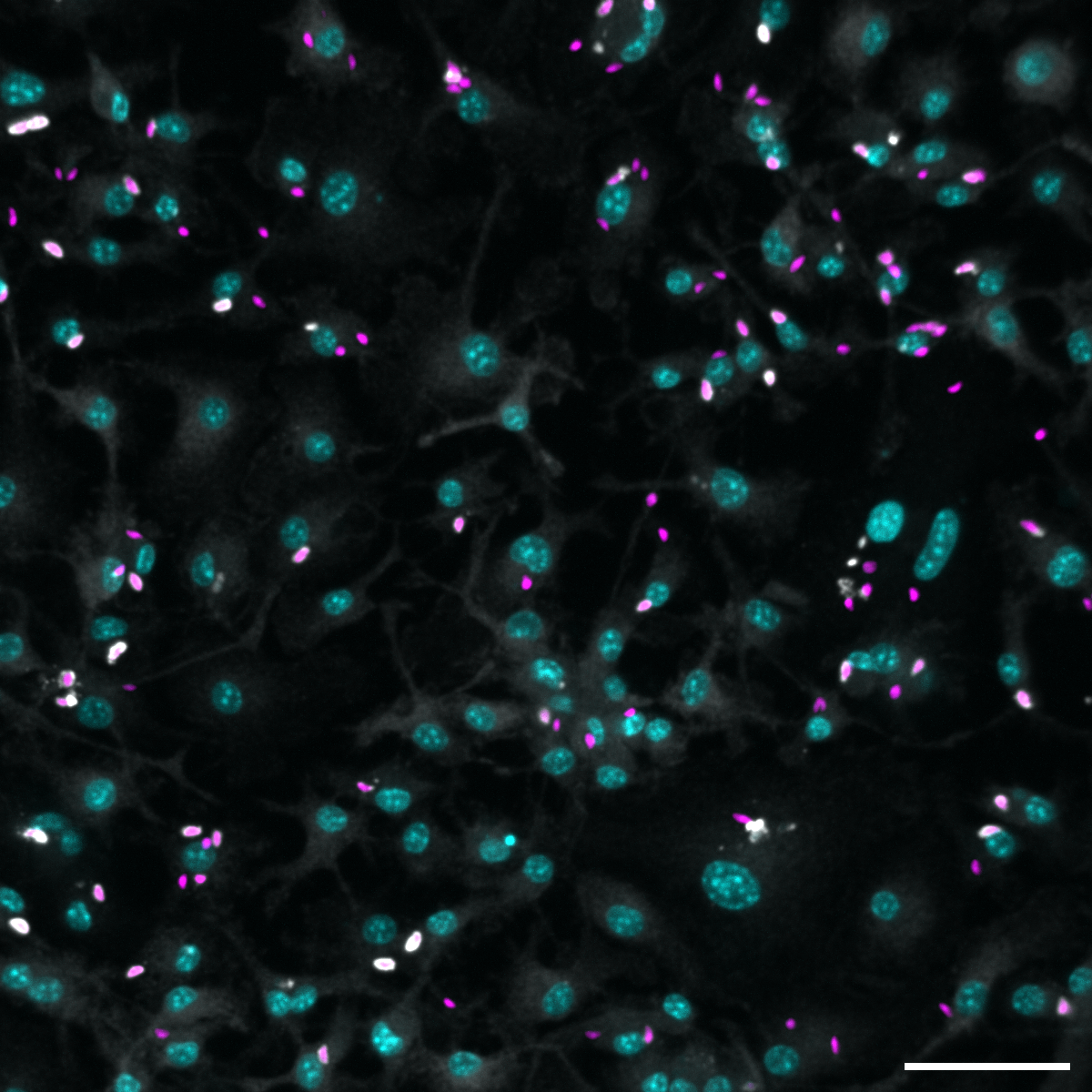

Supplement: Supplementary file 15 — Source data [file 41467_2025_58876_MOESM15_ESM.zip › Source suppl/Supplementary Figure 8_Source Data/Suppl Fig8a/FT_240927_PRU_EXP3_IRGd_IRGb10-0003_UPRT_IFNg_IRGb10_D_toxo_MERGE.tif]

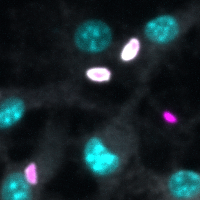

Supplement: Supplementary file 15 — Source data [file 41467_2025_58876_MOESM15_ESM.zip › Source suppl/Supplementary Figure 8_Source Data/Suppl Fig8a/FT_240927_PRU_EXP3_IRGd_IRGb10-0003_UPRT_IFNg_IRGb10_D_IRGb1_MERGE_crop.tif]

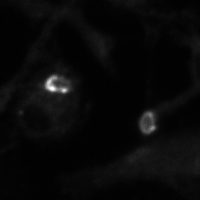

Supplement: Supplementary file 15 — Source data [file 41467_2025_58876_MOESM15_ESM.zip › Source suppl/Supplementary Figure 8_Source Data/Suppl Fig8a/FT_240927_PRU_EXP3_IRGd_IRGb10-0002_dUPRT_FK2_D_IFNg_FK2.tif]

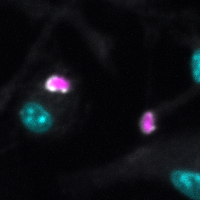

Supplement: Supplementary file 15 — Source data [file 41467_2025_58876_MOESM15_ESM.zip › Source suppl/Supplementary Figure 8_Source Data/Suppl Fig8a/FT_240927_PRU_EXP3_IRGd_IRGb10-0002_dUPRT_FK2_D_IFNg_FK2_MERGE_crop.tif]

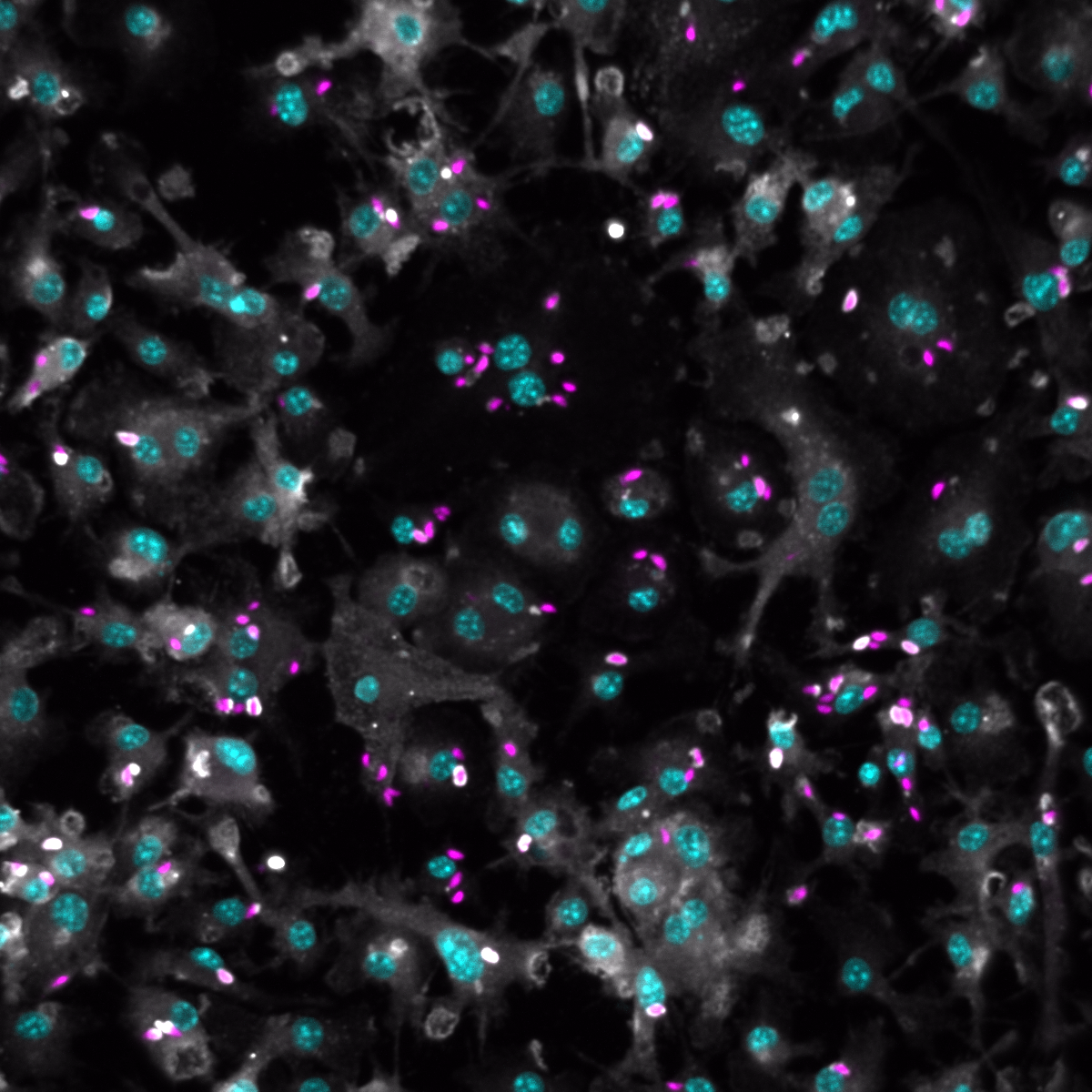

Supplement: Supplementary file 15 — Source data [file 41467_2025_58876_MOESM15_ESM.zip › Source suppl/Supplementary Figure 8_Source Data/Suppl Fig8a/FT_240927_PRU_EXP3_IRGd_IRGb10-0002_dUPRT_GBP2_C_IFNg_DAPI_MERGE.tif]

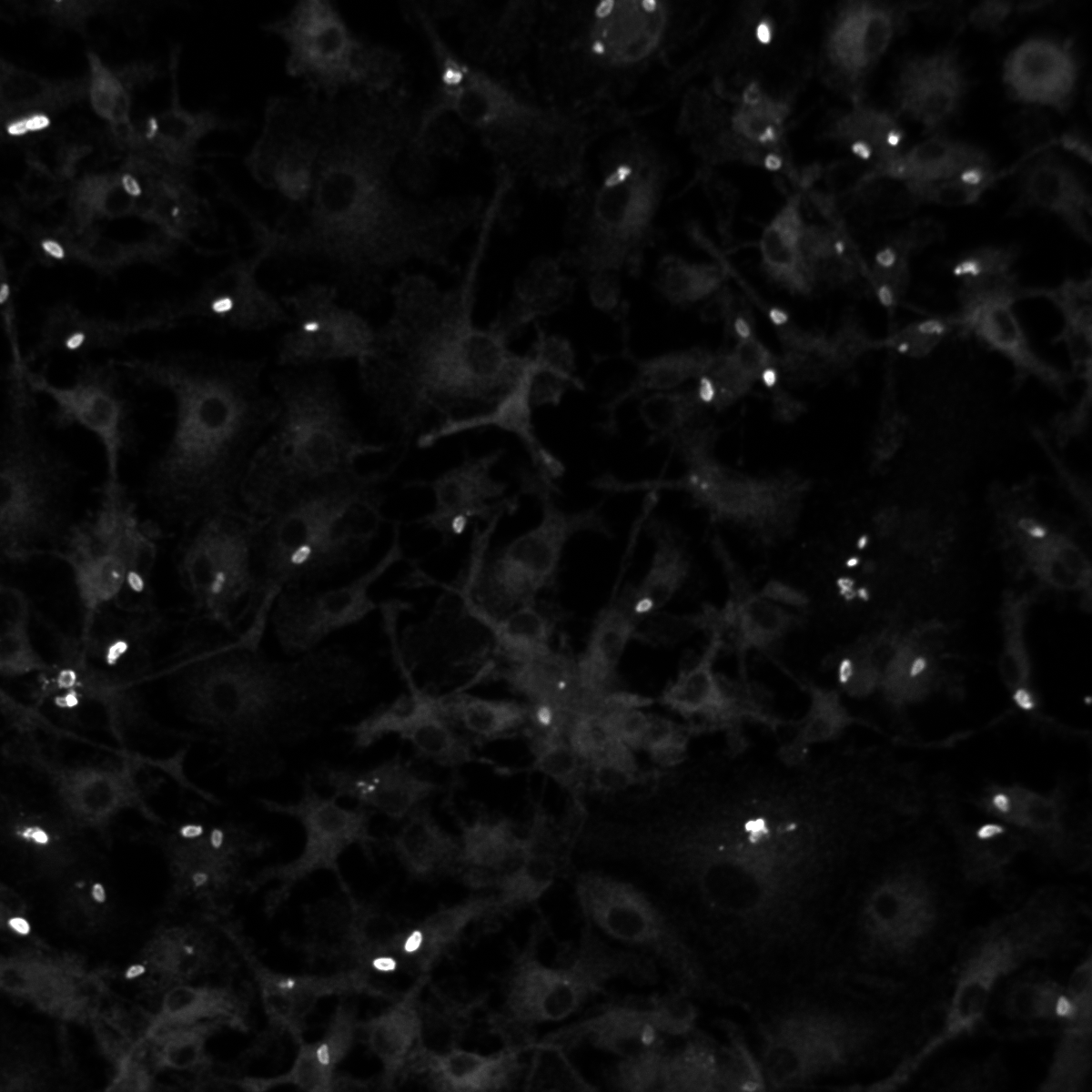

Supplement: Supplementary file 15 — Source data [file 41467_2025_58876_MOESM15_ESM.zip › Source suppl/Supplementary Figure 8_Source Data/Suppl Fig8a/FT_240927_PRU_EXP3_IRGd_IRGb10-0003_UPRT_IFNg_IRGb10_D_IRGb10.tif]

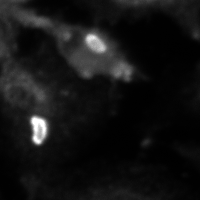

Supplement: Supplementary file 15 — Source data [file 41467_2025_58876_MOESM15_ESM.zip › Source suppl/Supplementary Figure 8_Source Data/Suppl Fig8a/FT_240927_PRU_EXP3_IRGd_IRGb10-0002_dUPRT_GBP2_D_IFNg_GBP2_crop.tif]

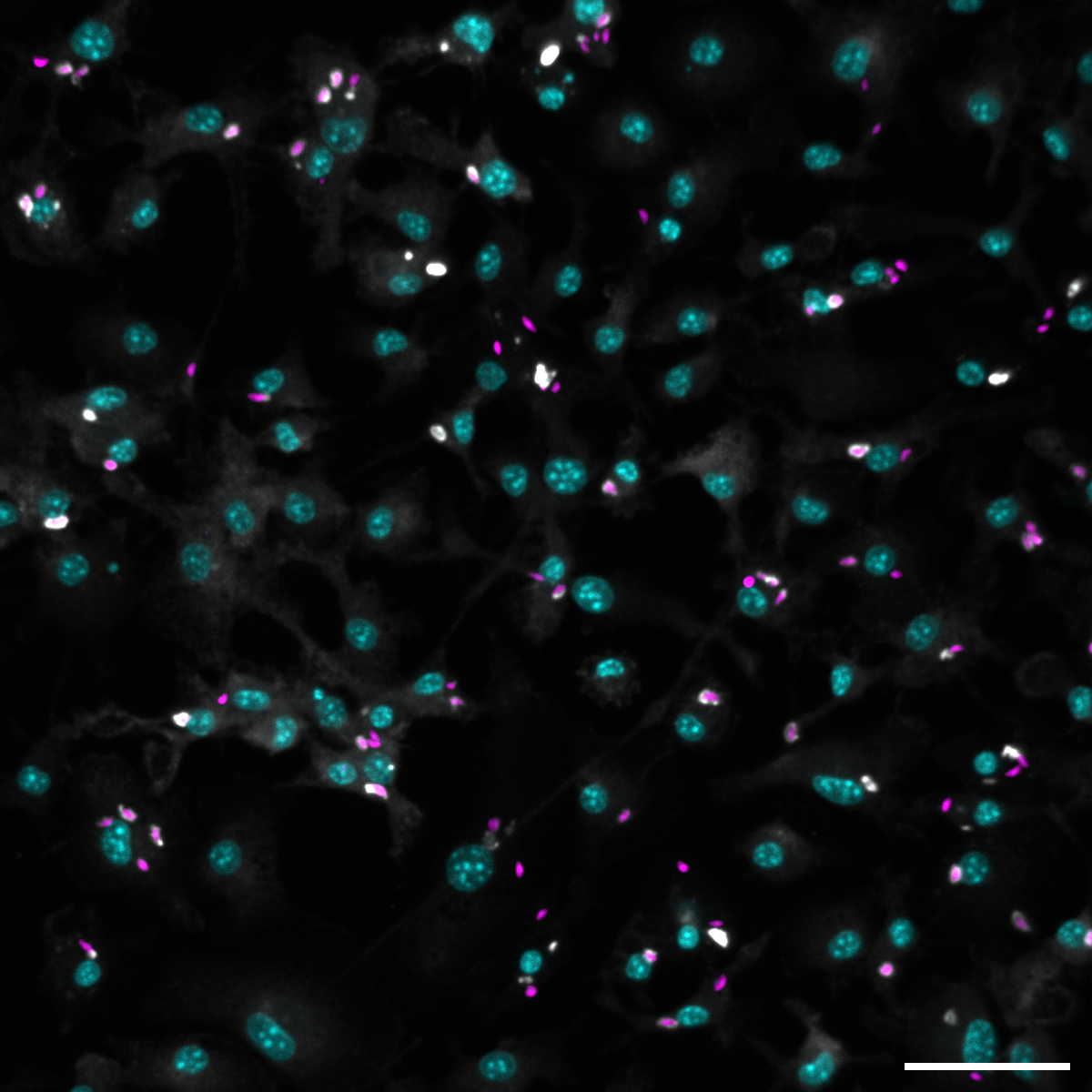

Supplement: Supplementary file 15 — Source data [file 41467_2025_58876_MOESM15_ESM.zip › Source suppl/Supplementary Figure 8_Source Data/Suppl Fig8a/FT_240927_PRU_EXP3_IRGd_IRGb10-0002_dUPRT_FK2_D_IFNg_FK2_MERGE.tif]

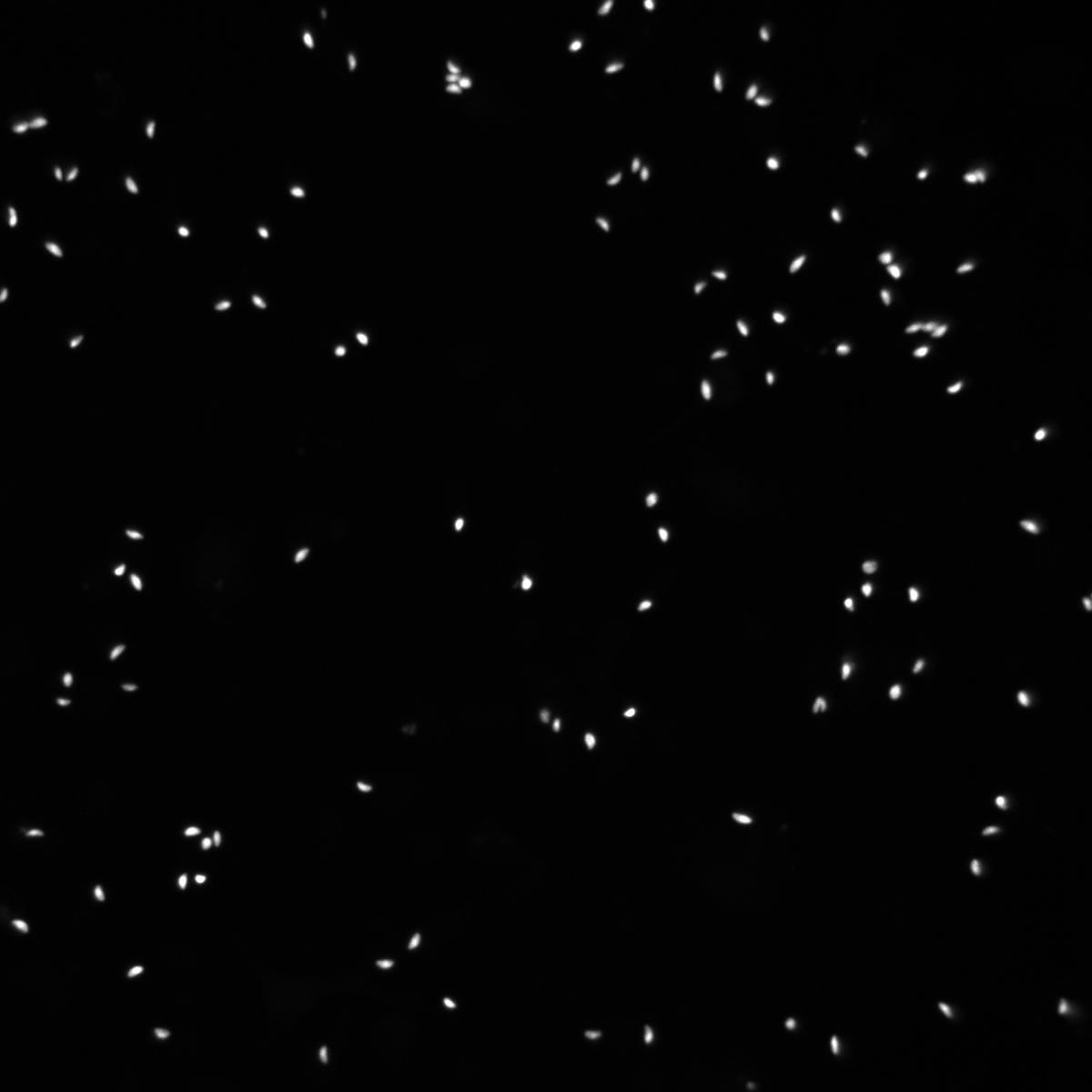

Supplement: Supplementary file 15 — Source data [file 41467_2025_58876_MOESM15_ESM.zip › Source suppl/Supplementary Figure 8_Source Data/Suppl Fig8a/FT_240927_PRU_EXP3_IRGd_IRGb10-0003_UPRT_IFNg_IRGb10_D_toxo.tif]

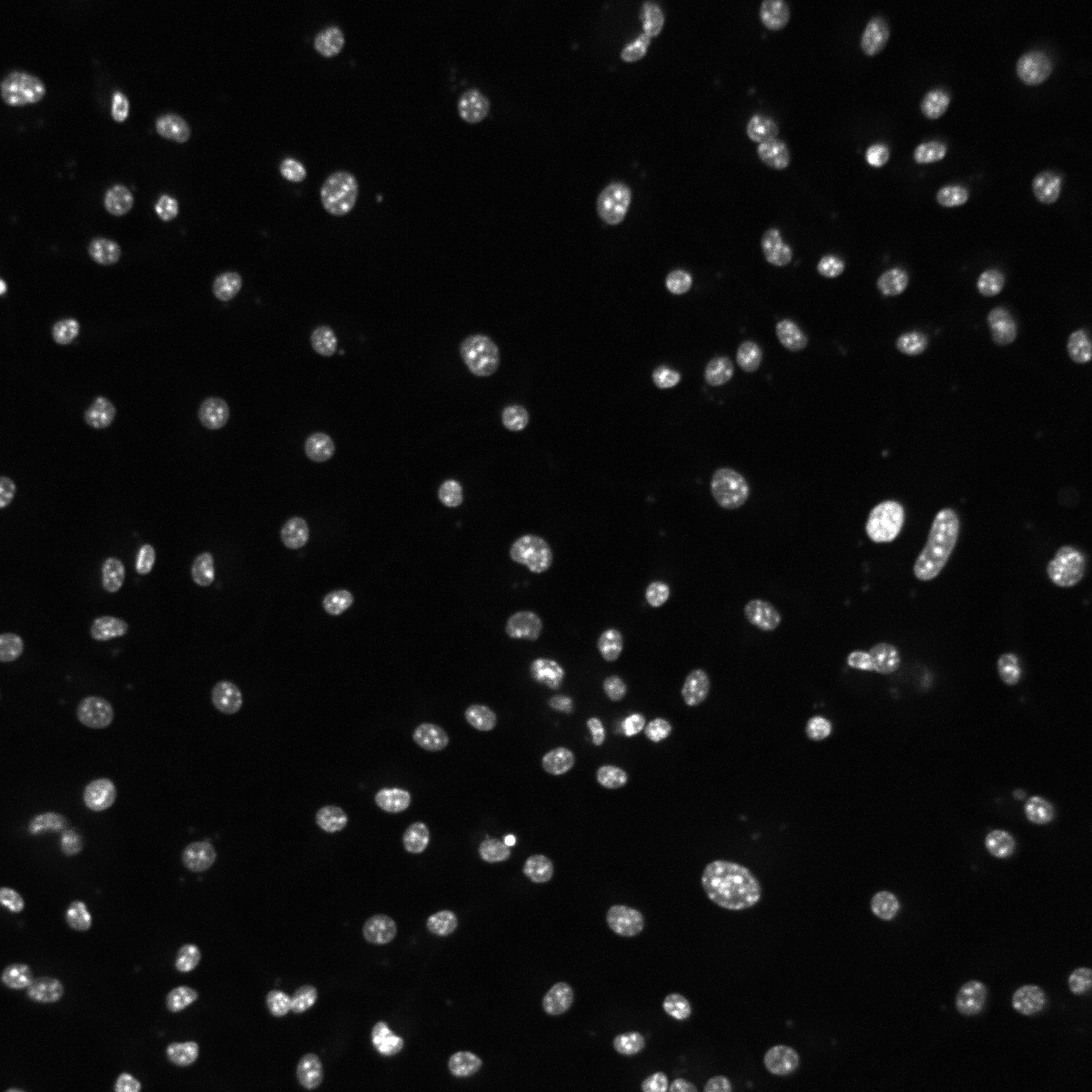

Supplement: Supplementary file 15 — Source data [file 41467_2025_58876_MOESM15_ESM.zip › Source suppl/Supplementary Figure 8_Source Data/Suppl Fig8a/FT_240927_PRU_EXP3_IRGd_IRGb10-0003_UPRT_IFNg_IRGb10_D_DAPI.tif]

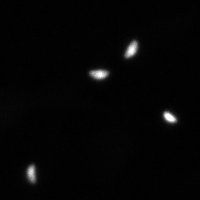

Supplement: Supplementary file 15 — Source data [file 41467_2025_58876_MOESM15_ESM.zip › Source suppl/Supplementary Figure 8_Source Data/Suppl Fig8a/FT_240927_PRU_EXP3_IRGd_IRGb10-0003_UPRT_IFNg_IRGb10_D_toxo_crop.tif]

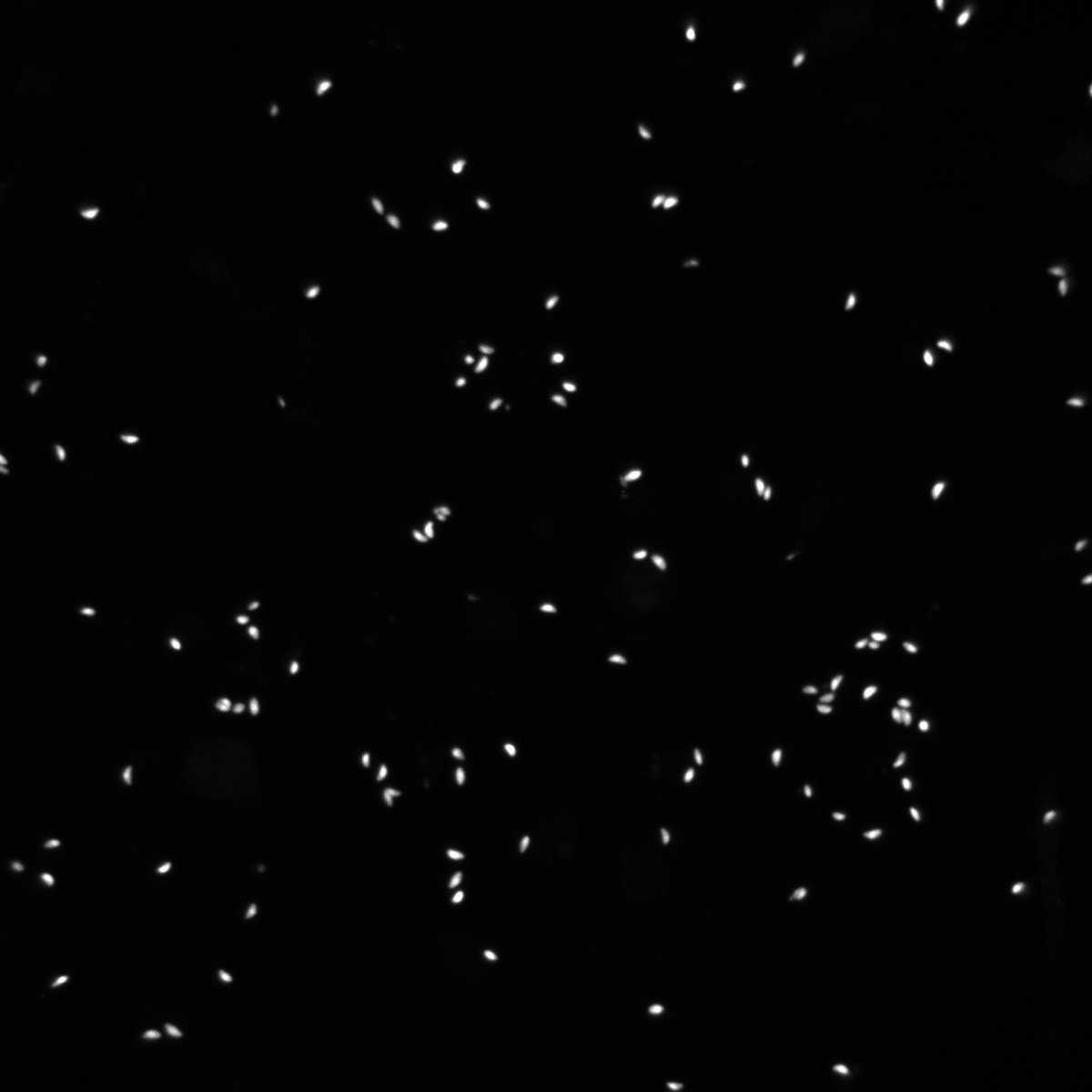

Supplement: Supplementary file 15 — Source data [file 41467_2025_58876_MOESM15_ESM.zip › Source suppl/Supplementary Figure 8_Source Data/Suppl Fig8a/FT_240927_PRU_EXP3_IRGd_IRGb10-0002_dUPRT_GBP2_C_IFNg_toxo.tif]

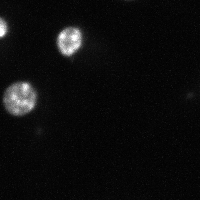

Supplement: Supplementary file 15 — Source data [file 41467_2025_58876_MOESM15_ESM.zip › Source suppl/Supplementary Figure 8_Source Data/Suppl Fig8a/FT_240927_PRU_EXP3_IRGd_IRGb10-0002_dUPRT_GBP2_D_IFNg_DAPI_crop.tif]

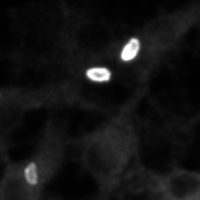

Supplement: Supplementary file 15 — Source data [file 41467_2025_58876_MOESM15_ESM.zip › Source suppl/Supplementary Figure 8_Source Data/Suppl Fig8a/FT_240927_PRU_EXP3_IRGd_IRGb10-0003_UPRT_IFNg_IRGb10_D_IRGb1_crop.tif]

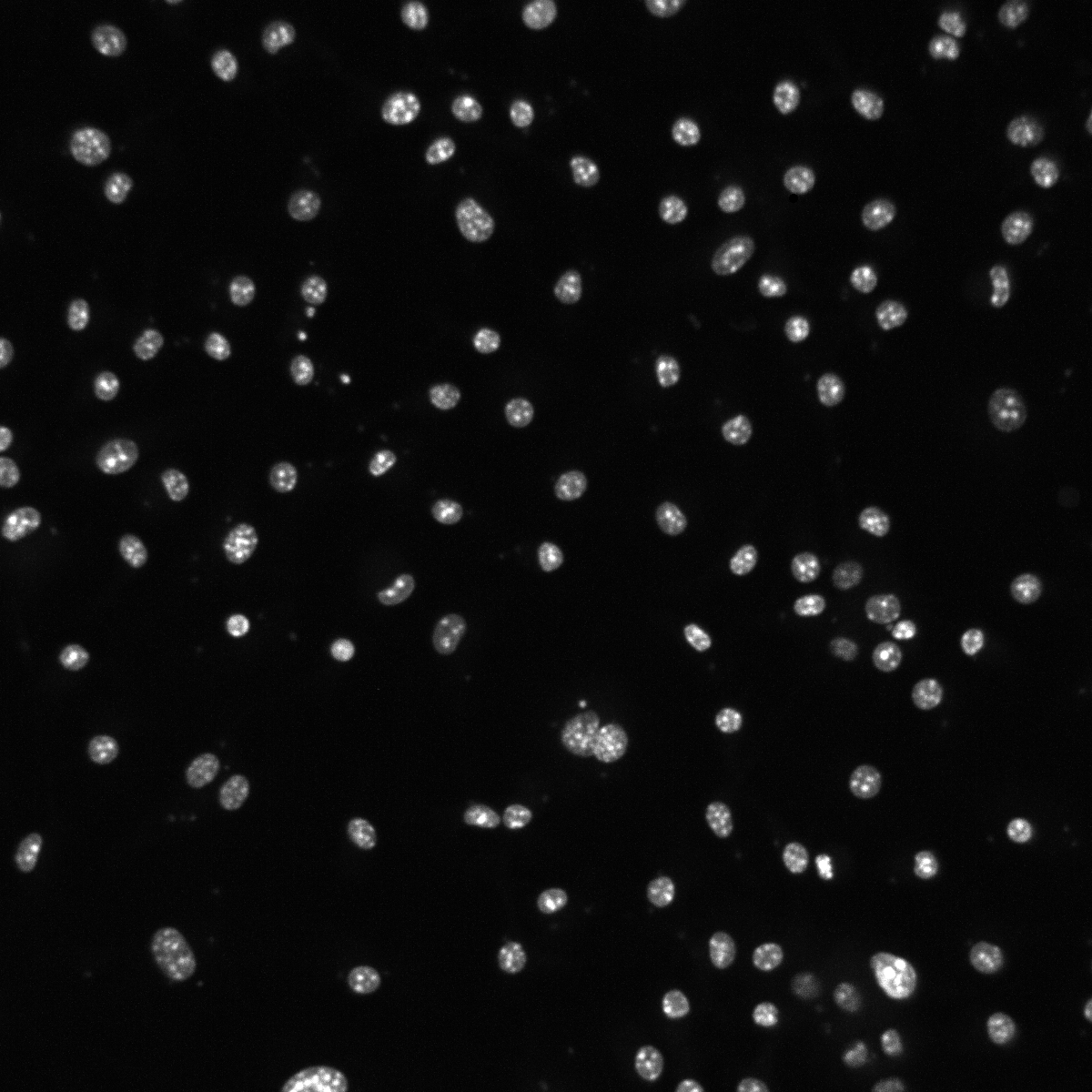

Supplement: Supplementary file 15 — Source data [file 41467_2025_58876_MOESM15_ESM.zip › Source suppl/Supplementary Figure 8_Source Data/Suppl Fig8a/FT_240927_PRU_EXP3_IRGd_IRGb10-0002_dUPRT_GBP2_D_IFNg_DAPI.tif]

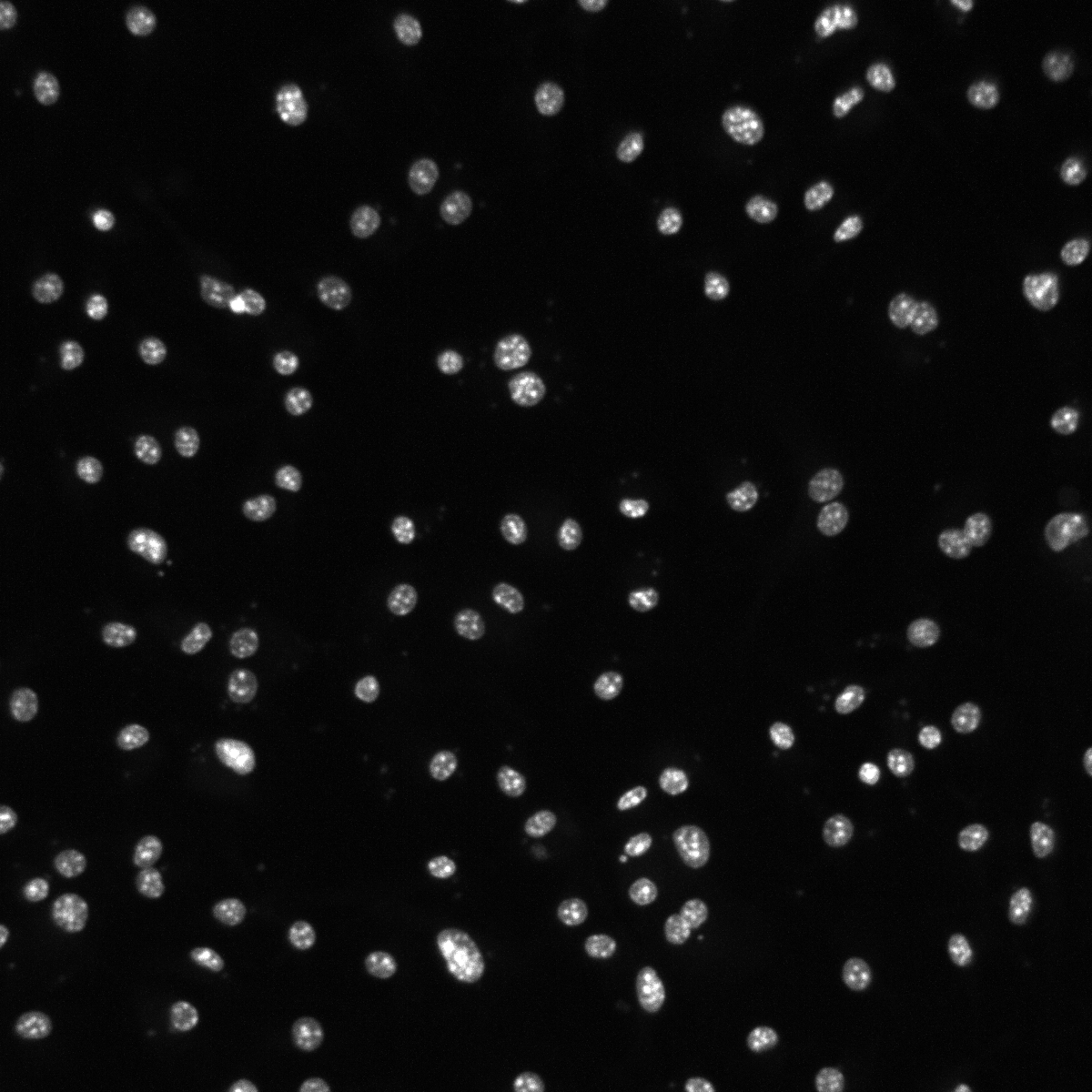

Supplement: Supplementary file 15 — Source data [file 41467_2025_58876_MOESM15_ESM.zip › Source suppl/Supplementary Figure 8_Source Data/Suppl Fig8a/FT_240927_PRU_EXP3_IRGd_IRGb10-0002_dUPRT_GBP2_C_IFNg_DAPI.tif]

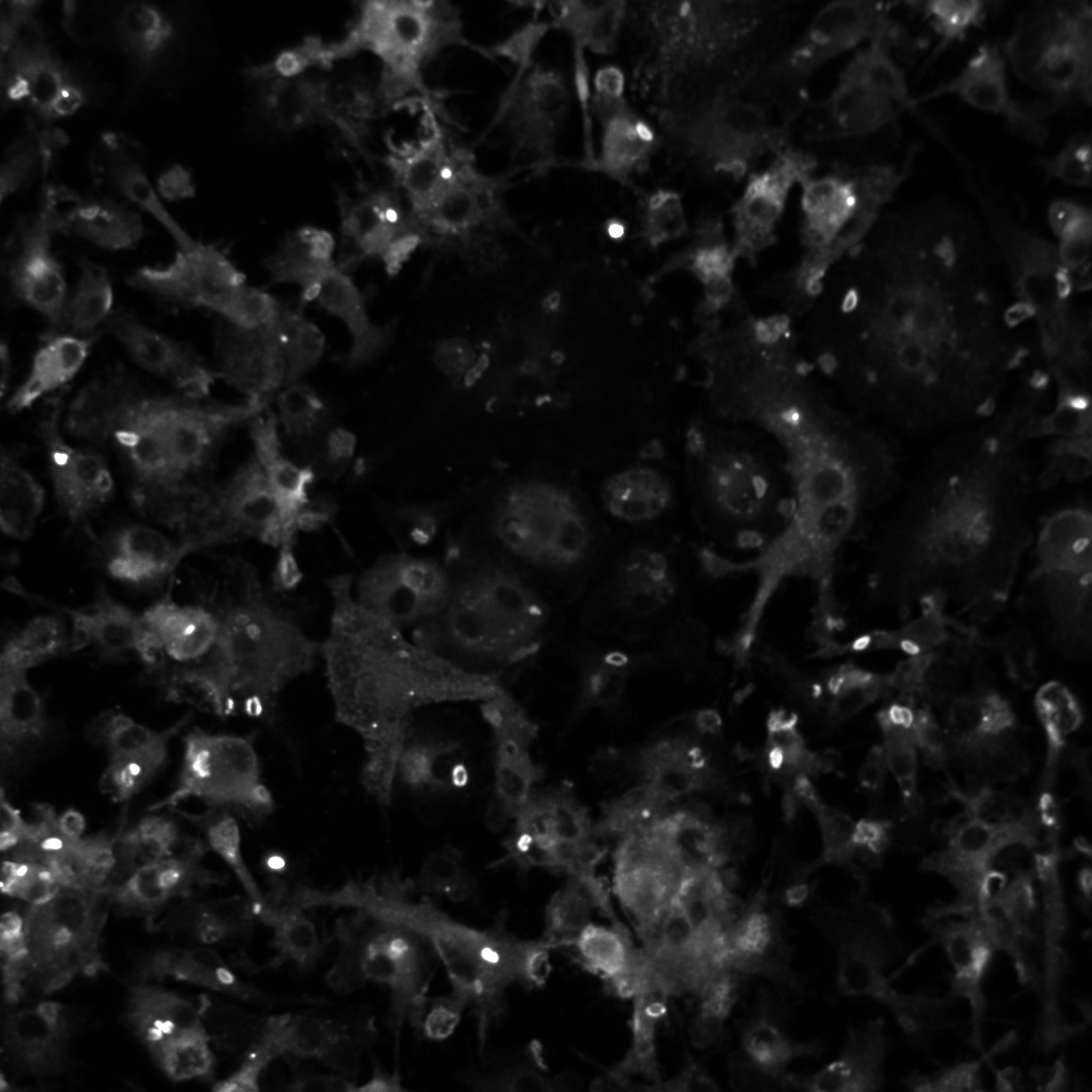

Supplement: Supplementary file 15 — Source data [file 41467_2025_58876_MOESM15_ESM.zip › Source suppl/Supplementary Figure 8_Source Data/Suppl Fig8a/FT_240927_PRU_EXP3_IRGd_IRGb10-0002_dUPRT_GBP2_C_IFNg_GBP2.tif]

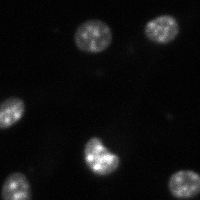

Supplement: Supplementary file 15 — Source data [file 41467_2025_58876_MOESM15_ESM.zip › Source suppl/Supplementary Figure 8_Source Data/Suppl Fig8a/FT_240927_PRU_EXP3_IRGd_IRGb10-0003_UPRT_IFNg_IRGb10_D_DAPI_crop.tif]

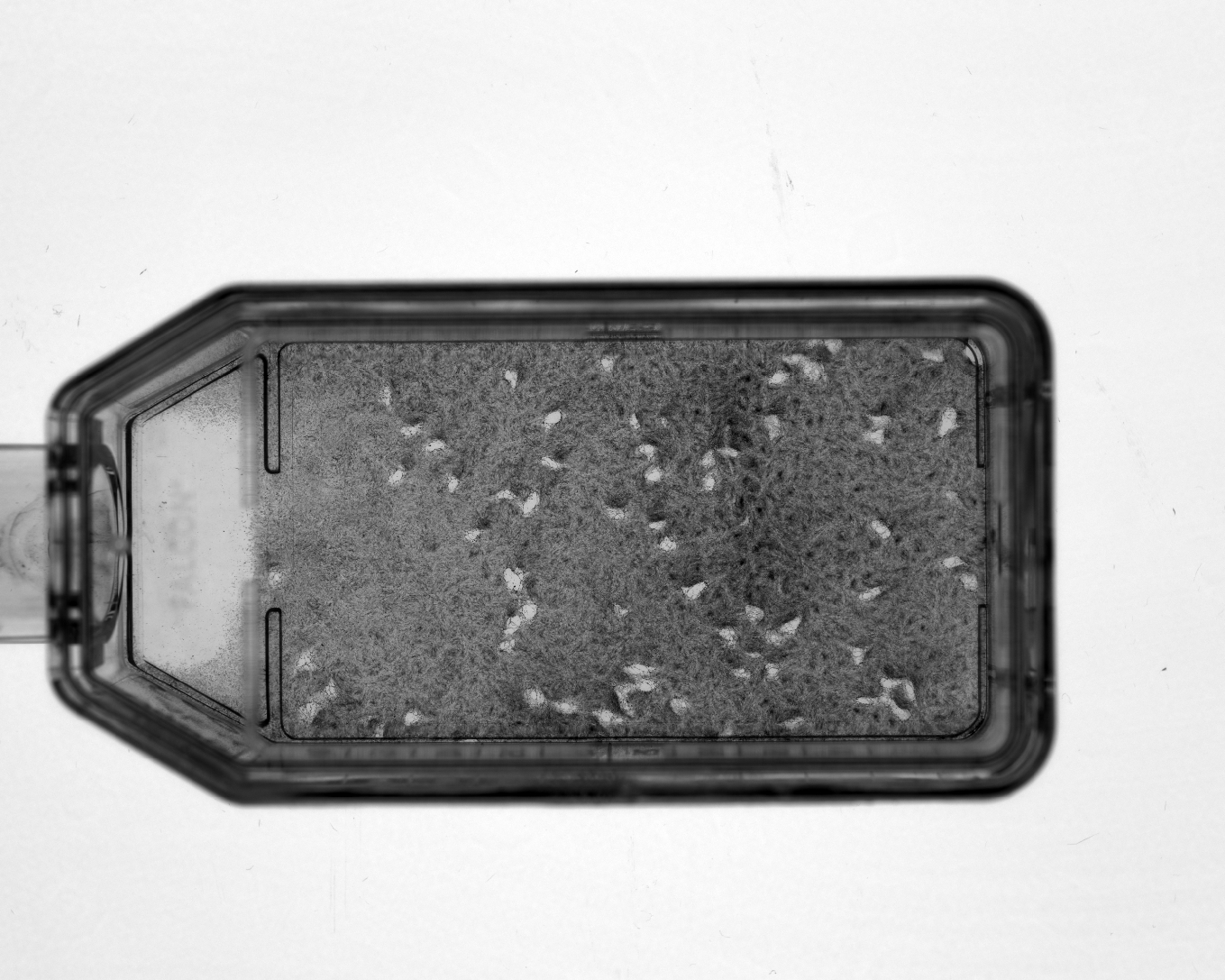

Supplement: Supplementary file 15 — Source data [file 41467_2025_58876_MOESM15_ESM.zip › Source suppl/Supplementary Figure 2_Source Data/Suppl Fig 2d/vand_ku_500_n(Silver Stain).jpg]

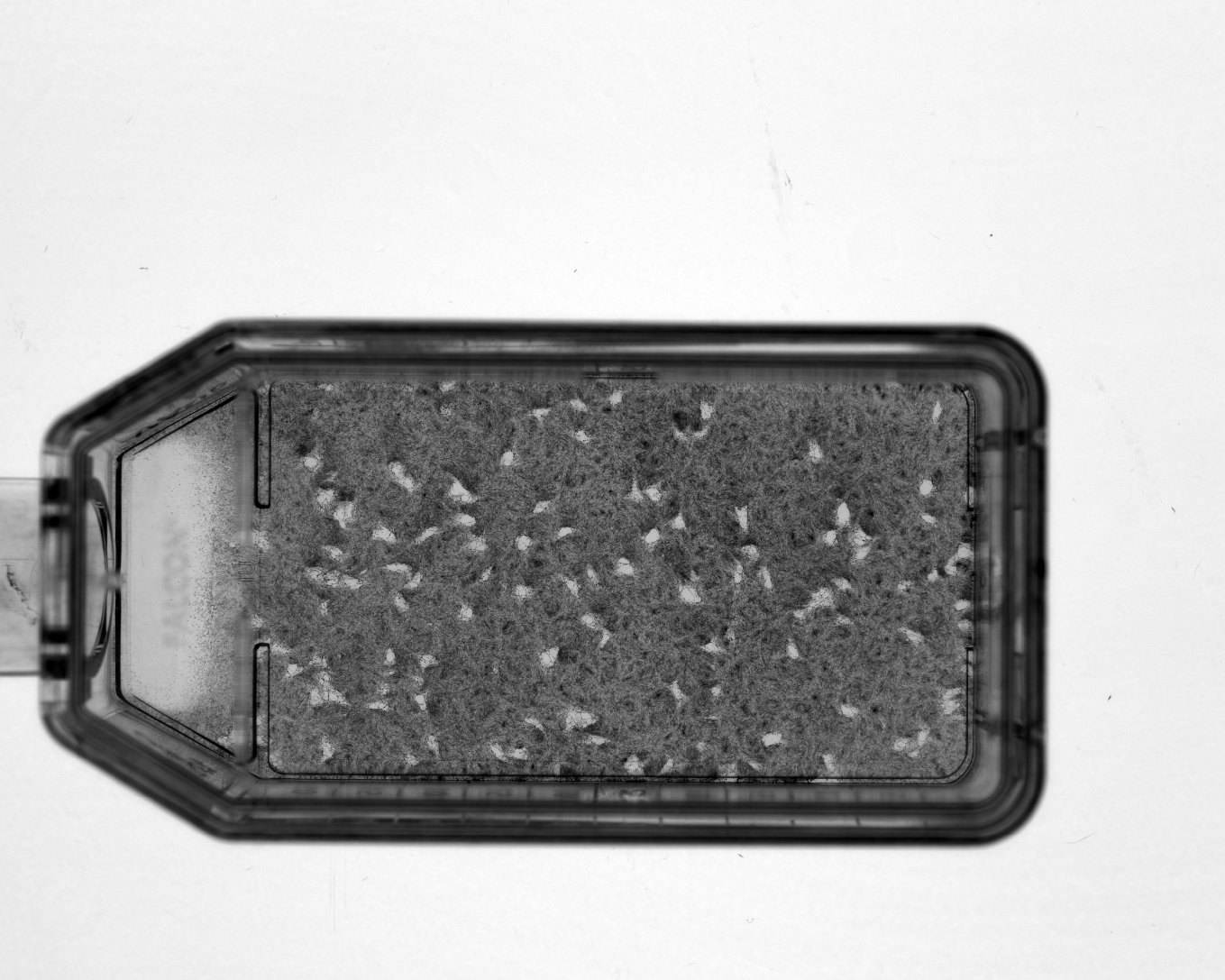

Supplement: Supplementary file 15 — Source data [file 41467_2025_58876_MOESM15_ESM.zip › Source suppl/Supplementary Figure 2_Source Data/Suppl Fig 2d/vandz_GRA12_COMPL_C2_500(Silver Stain).jpg]

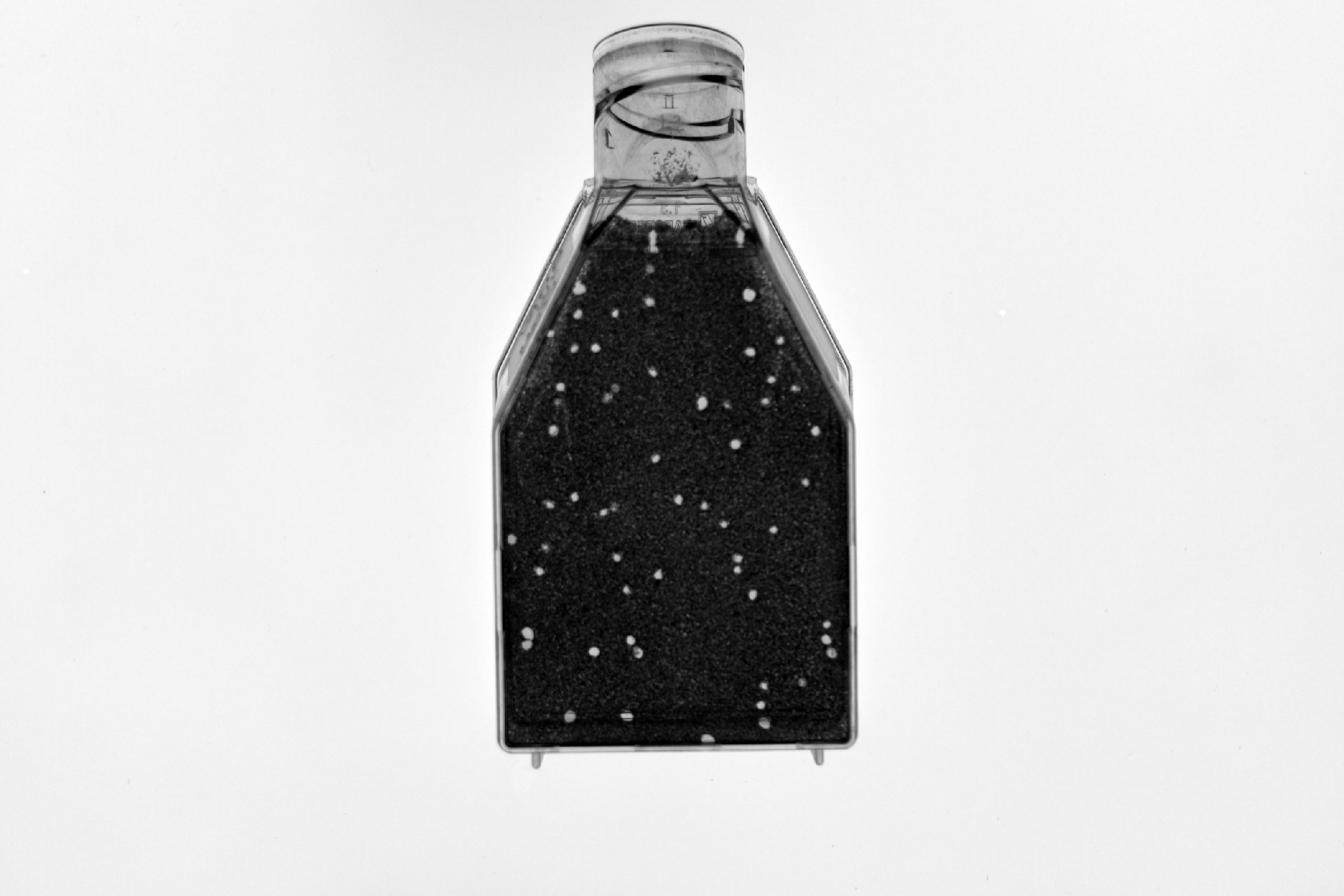

Supplement: Supplementary file 15 — Source data [file 41467_2025_58876_MOESM15_ESM.zip › Source suppl/Supplementary Figure 2_Source Data/Suppl Fig 2d/igcuser 2024-12-19 17h11m36s(Silver Stain).jpg]

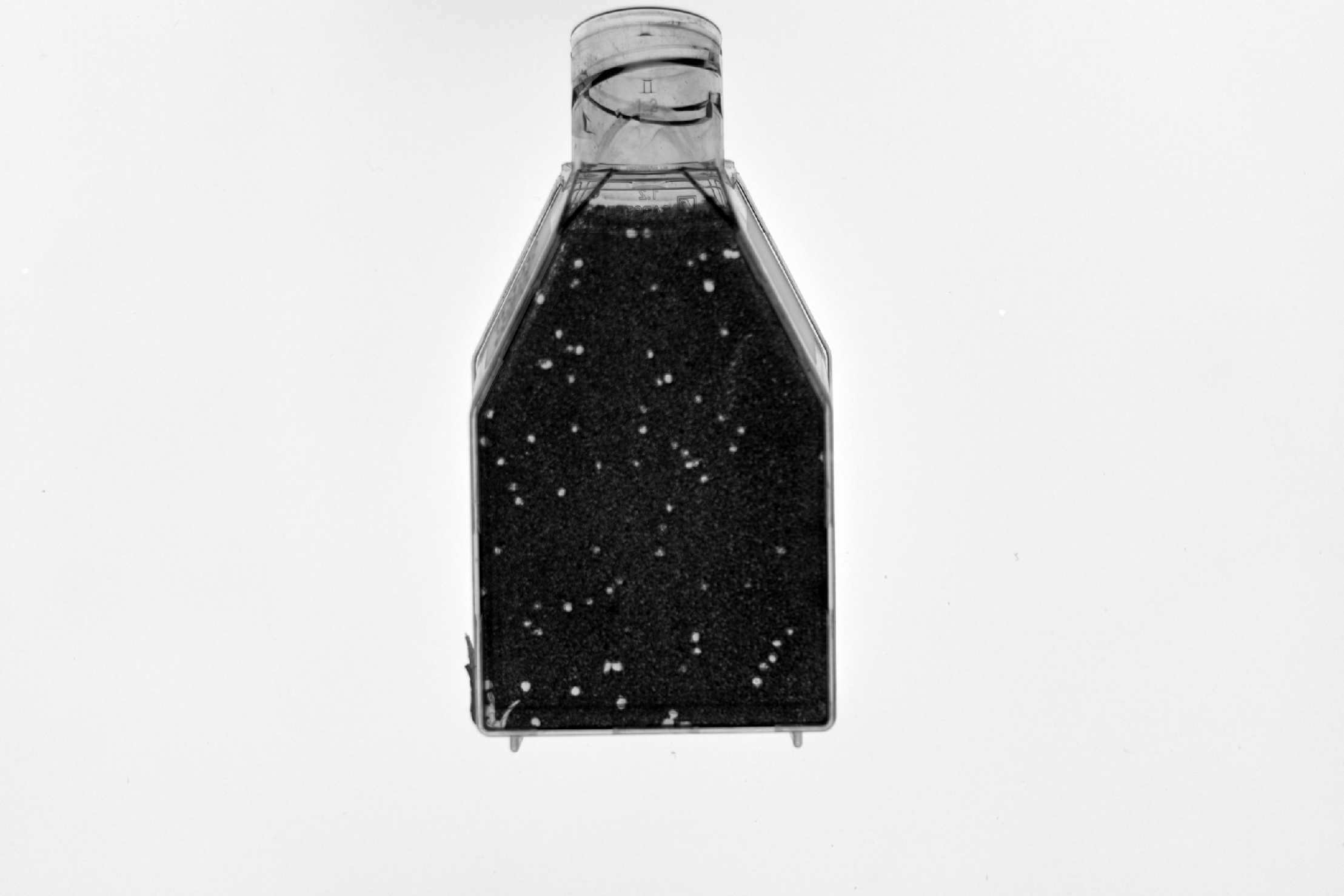

Supplement: Supplementary file 15 — Source data [file 41467_2025_58876_MOESM15_ESM.zip › Source suppl/Supplementary Figure 2_Source Data/Suppl Fig 2d/igcuser 2024-12-19 17h14m33s(Silver Stain).tif]

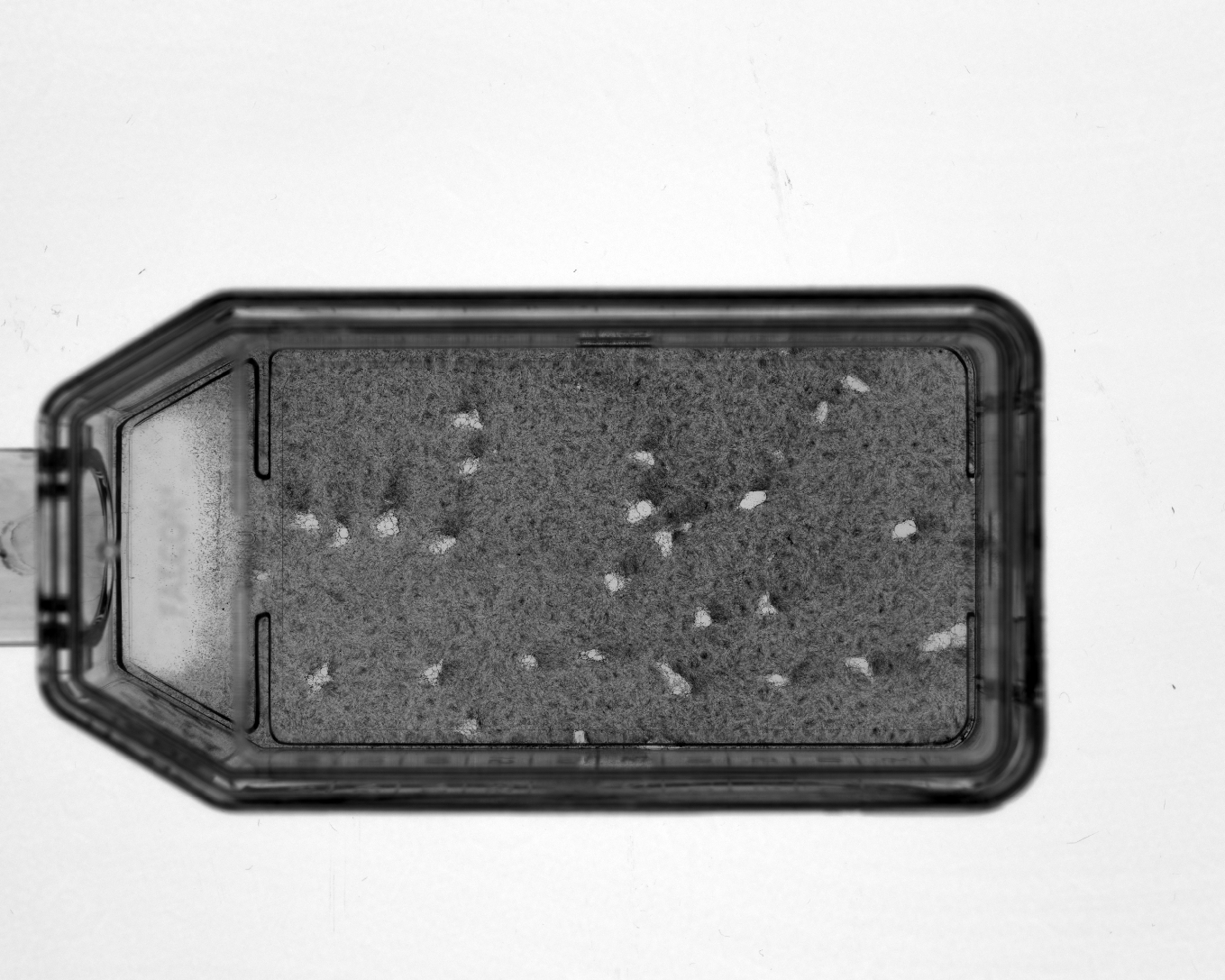

Supplement: Supplementary file 15 — Source data [file 41467_2025_58876_MOESM15_ESM.zip › Source suppl/Supplementary Figure 2_Source Data/Suppl Fig 2d/vand_gra12_200_N(Silver Stain).jpg]

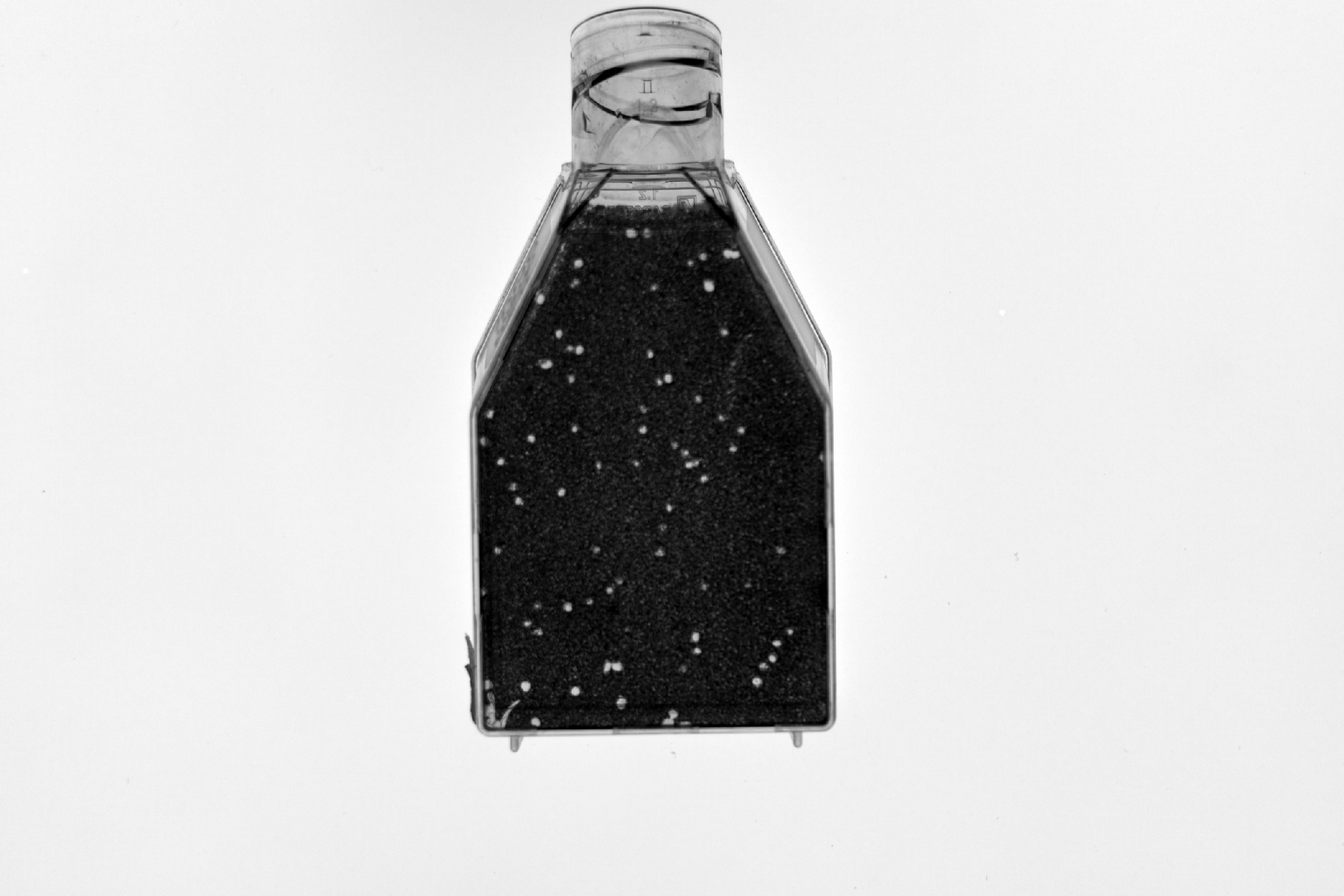

Supplement: Supplementary file 15 — Source data [file 41467_2025_58876_MOESM15_ESM.zip › Source suppl/Supplementary Figure 2_Source Data/Suppl Fig 2d/igcuser 2024-12-19 17h14m33s(Silver Stain).jpg]

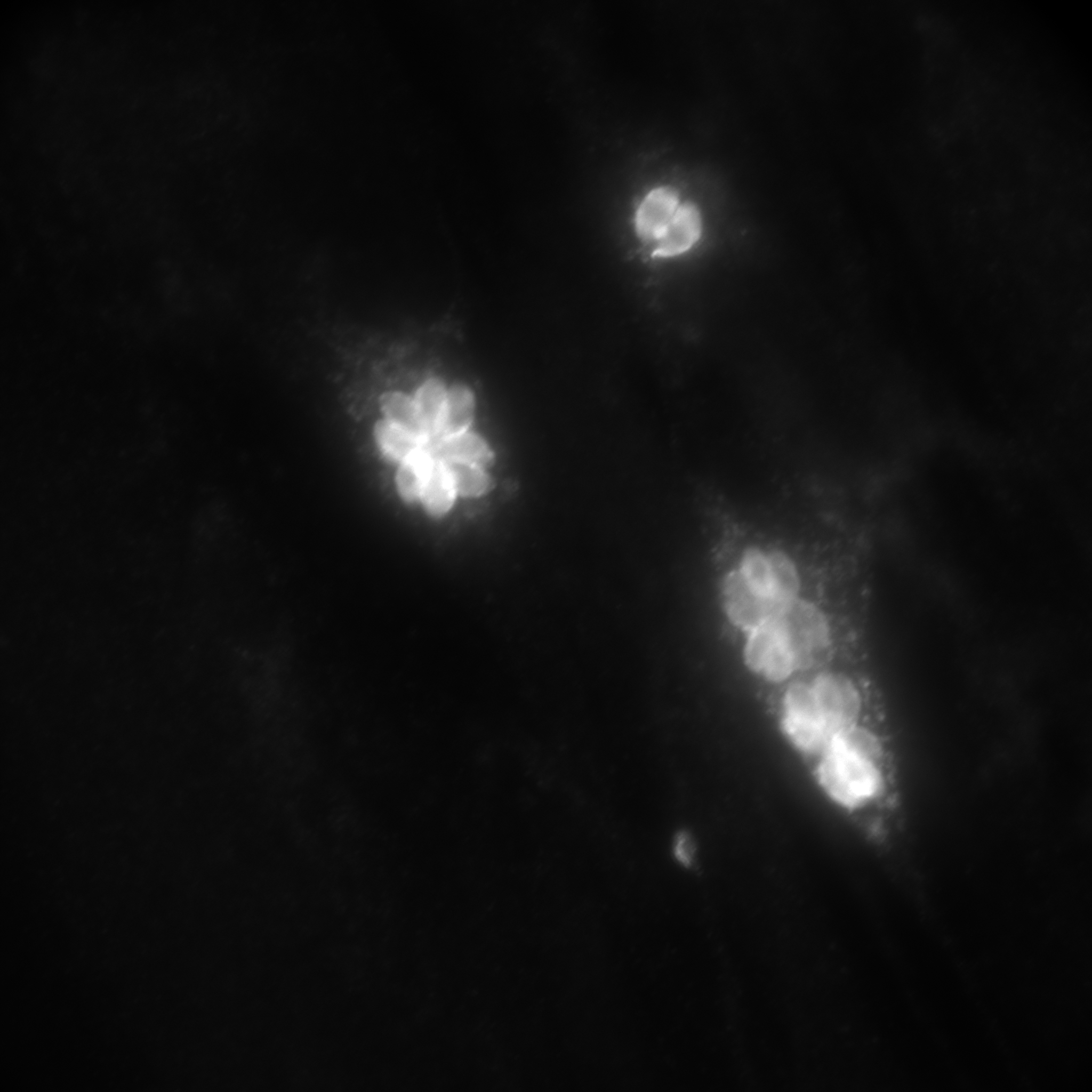

Supplement: Supplementary file 15 — Source data [file 41467_2025_58876_MOESM15_ESM.zip › Source suppl/Supplementary Figure 2_Source Data/Suppl Fig 2b/FT_210607_VAND_dGRA12_C2_GRA12_594_toxo488_A-0002_toxo_adj.tif]

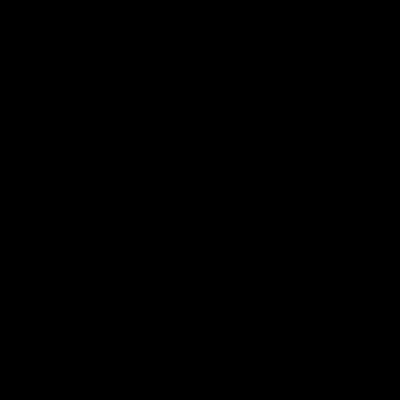

Supplement: Supplementary file 15 — Source data [file 41467_2025_58876_MOESM15_ESM.zip › Source suppl/Supplementary Figure 2_Source Data/Suppl Fig 2b/FT_210607_VAND_dGRA12_C2_GRA12_594_toxo488_A-0003_GRA12_adj_crop.tif]

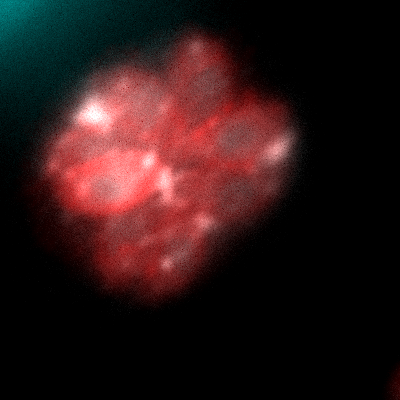

Supplement: Supplementary file 15 — Source data [file 41467_2025_58876_MOESM15_ESM.zip › Source suppl/Supplementary Figure 2_Source Data/Suppl Fig 2b/dKU80_MERGE_red_crop_MERGE.tif]

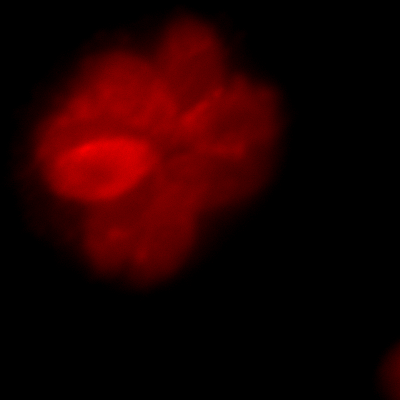

Supplement: Supplementary file 15 — Source data [file 41467_2025_58876_MOESM15_ESM.zip › Source suppl/Supplementary Figure 2_Source Data/Suppl Fig 2b/FT_210607_VANDdKu80_GRA12_594_toxo488_A-0002_toxo_adj_red_crop.tif]

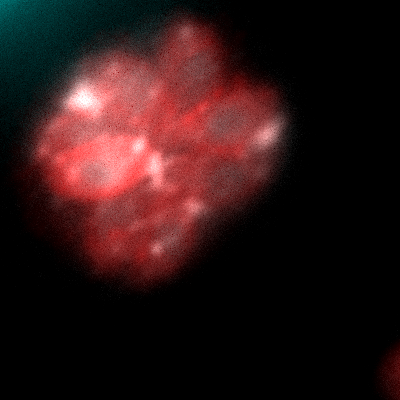

Supplement: Supplementary file 15 — Source data [file 41467_2025_58876_MOESM15_ESM.zip › Source suppl/Supplementary Figure 2_Source Data/Suppl Fig 2b/dKU80_MERGE_red_crop.tif]

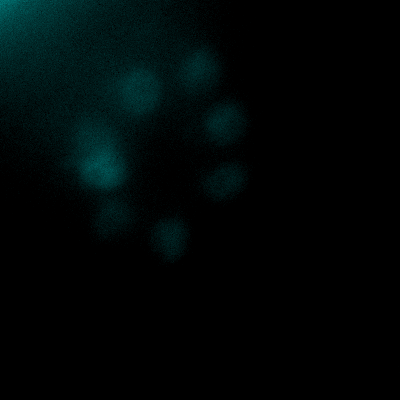

Supplement: Supplementary file 15 — Source data [file 41467_2025_58876_MOESM15_ESM.zip › Source suppl/Supplementary Figure 2_Source Data/Suppl Fig 2b/FT_210607_VANDdKu80_GRA12_594_toxo488_A-0001_DAPi_adj_crop.tif]

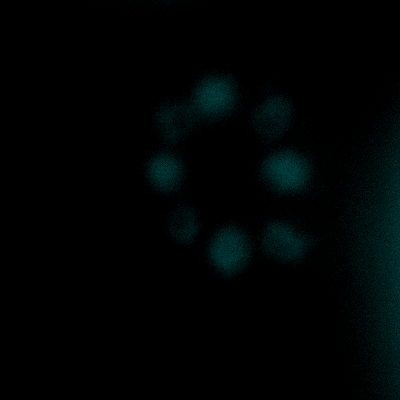

Supplement: Supplementary file 15 — Source data [file 41467_2025_58876_MOESM15_ESM.zip › Source suppl/Supplementary Figure 2_Source Data/Suppl Fig 2b/FT_210607_VAND_dGRA12_C2_GRA12_594_toxo488_A-0001_DAPI_adj_crop.tif]

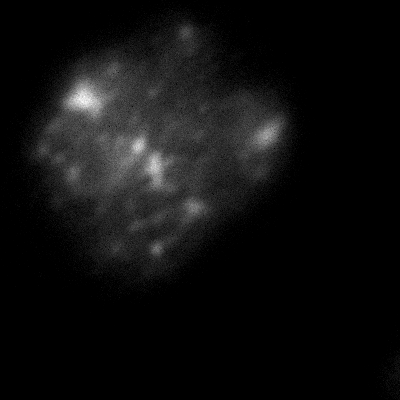

Supplement: Supplementary file 15 — Source data [file 41467_2025_58876_MOESM15_ESM.zip › Source suppl/Supplementary Figure 2_Source Data/Suppl Fig 2b/FT_210607_VANDdKu80_GRA12_594_toxo488_A-0003_GRA12_adj_crop.tif]

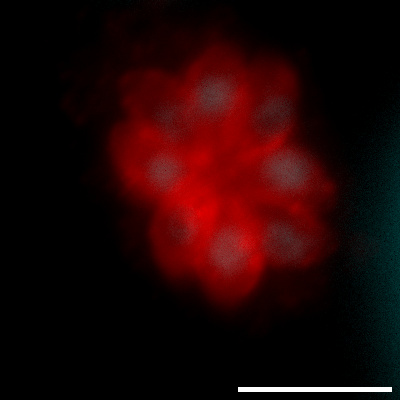

Supplement: Supplementary file 15 — Source data [file 41467_2025_58876_MOESM15_ESM.zip › Source suppl/Supplementary Figure 2_Source Data/Suppl Fig 2b/dGRA12_MERGE_scale.tif]

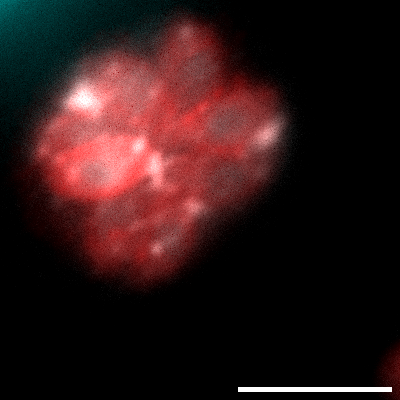

Supplement: Supplementary file 15 — Source data [file 41467_2025_58876_MOESM15_ESM.zip › Source suppl/Supplementary Figure 2_Source Data/Suppl Fig 2b/dKU80_MERGE_red_crop_scale.tif]

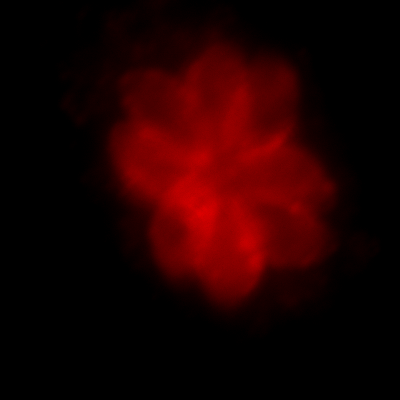

Supplement: Supplementary file 15 — Source data [file 41467_2025_58876_MOESM15_ESM.zip › Source suppl/Supplementary Figure 2_Source Data/Suppl Fig 2b/FT_210607_VAND_dGRA12_C2_GRA12_594_toxo488_A-0002_toxo_adj_crop.tif]

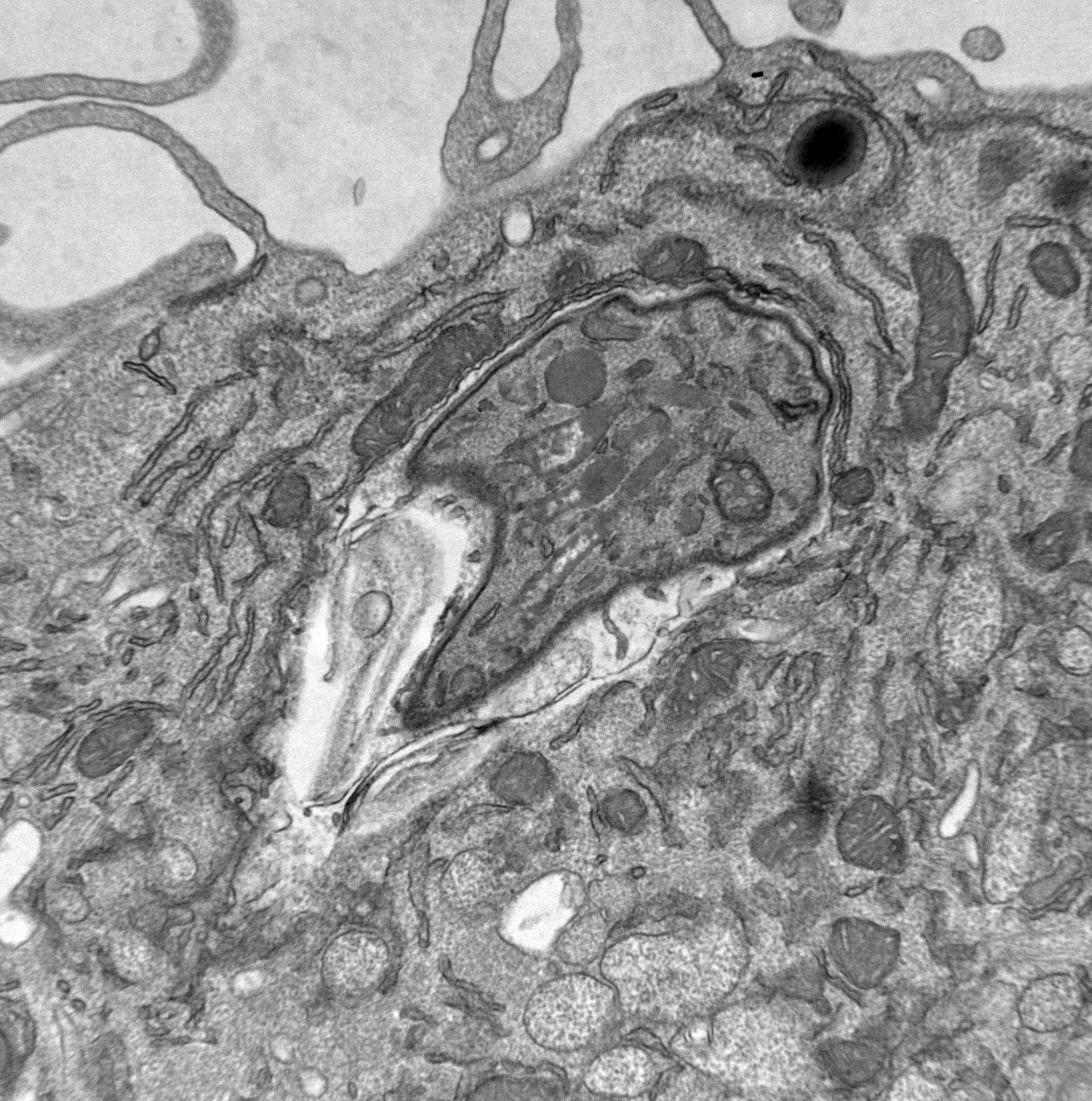

Supplement: Supplementary file 15 — Source data [file 41467_2025_58876_MOESM15_ESM.zip › Source suppl/Supplementary Figure 7_Source Data/Suppl Fig7c/EM04576_05_060423_007_SA-MAG_X8000.tif]

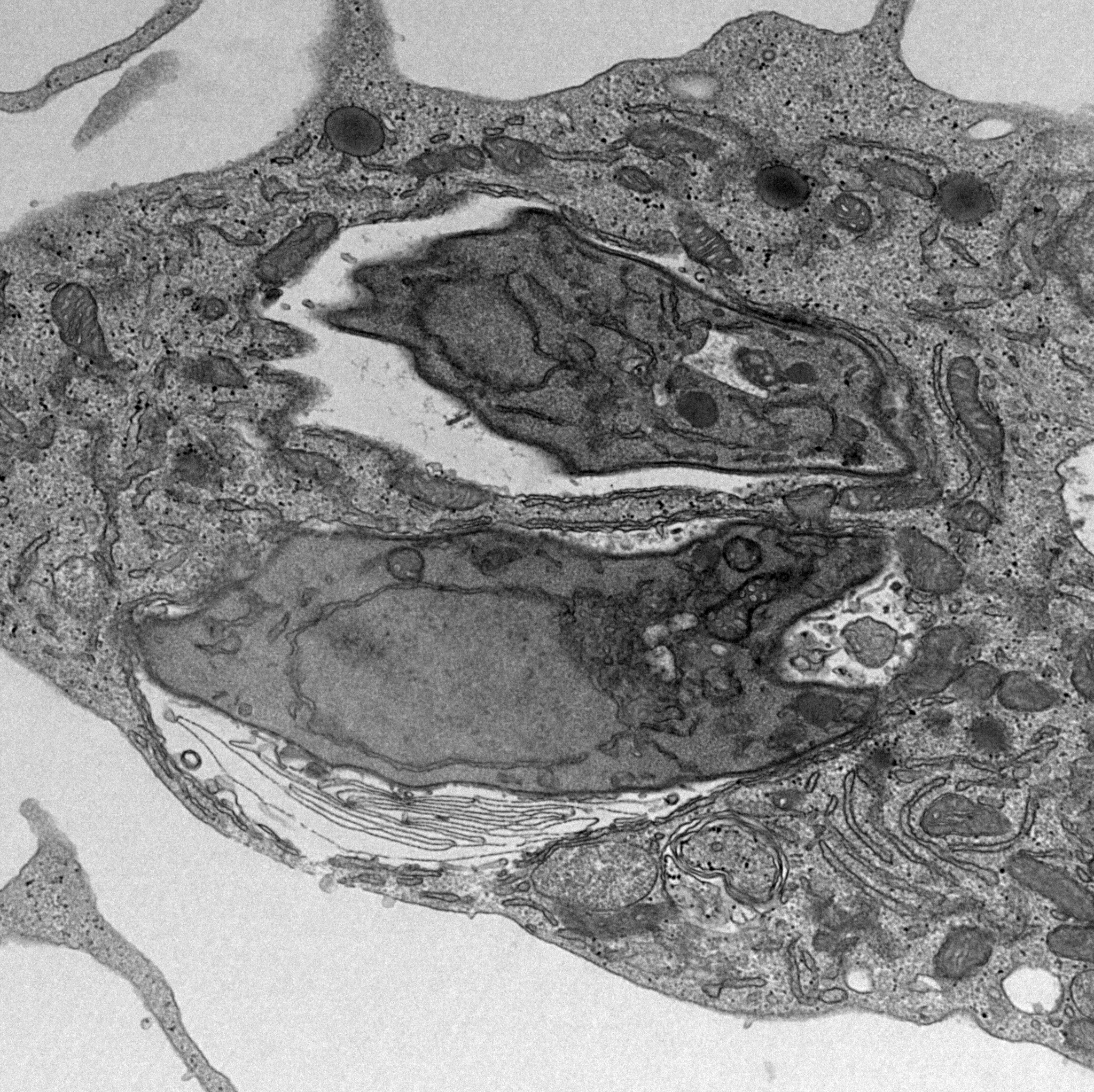

Supplement: Supplementary file 15 — Source data [file 41467_2025_58876_MOESM15_ESM.zip › Source suppl/Supplementary Figure 7_Source Data/Suppl Fig7c/EM04576_03_300623_SA-MAG_X6000_006.tif]

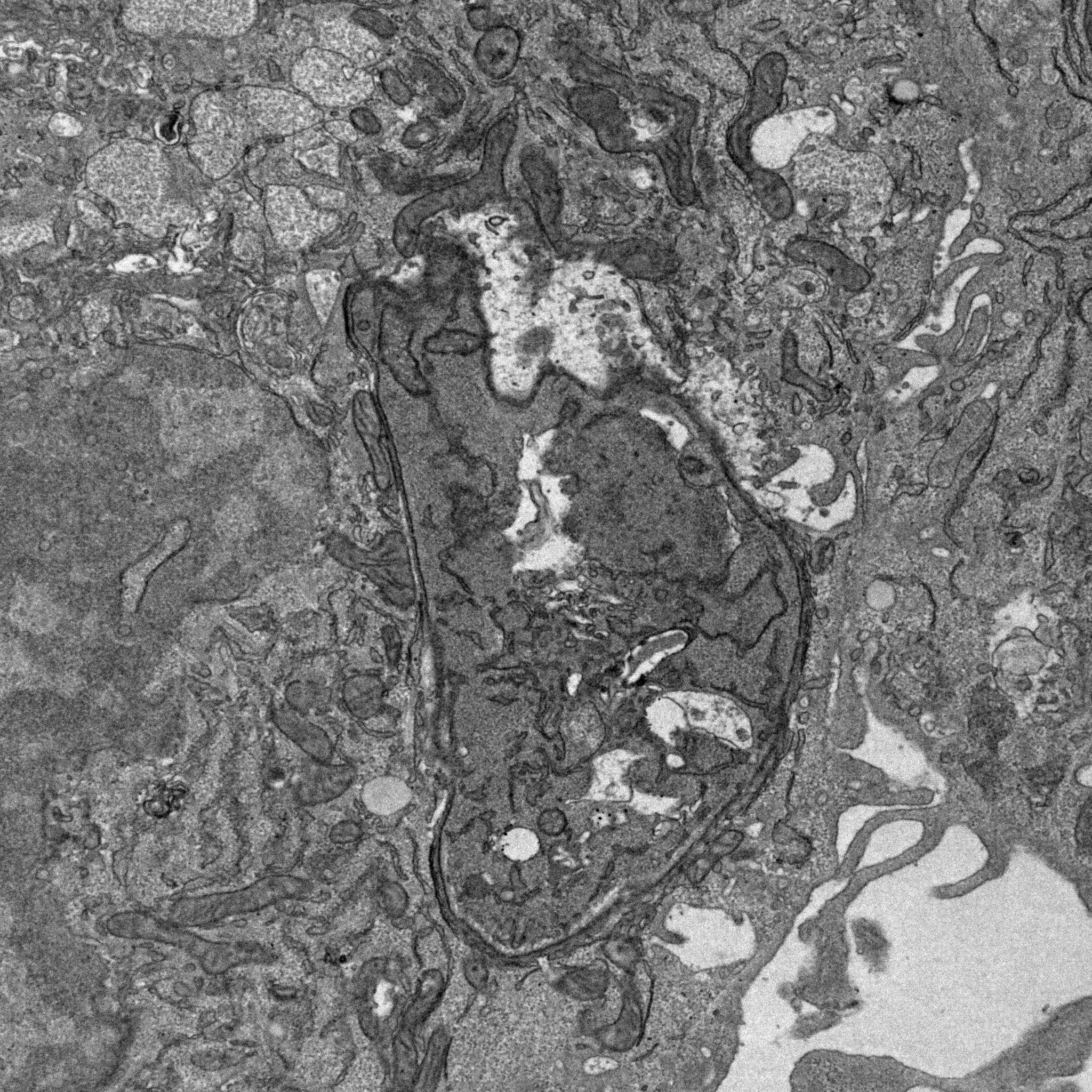

Supplement: Supplementary file 15 — Source data [file 41467_2025_58876_MOESM15_ESM.zip › Source suppl/Supplementary Figure 7_Source Data/Suppl Fig7c/EM04576_01_060223_blindedexp_007_SA-MAG_X5000.tif]

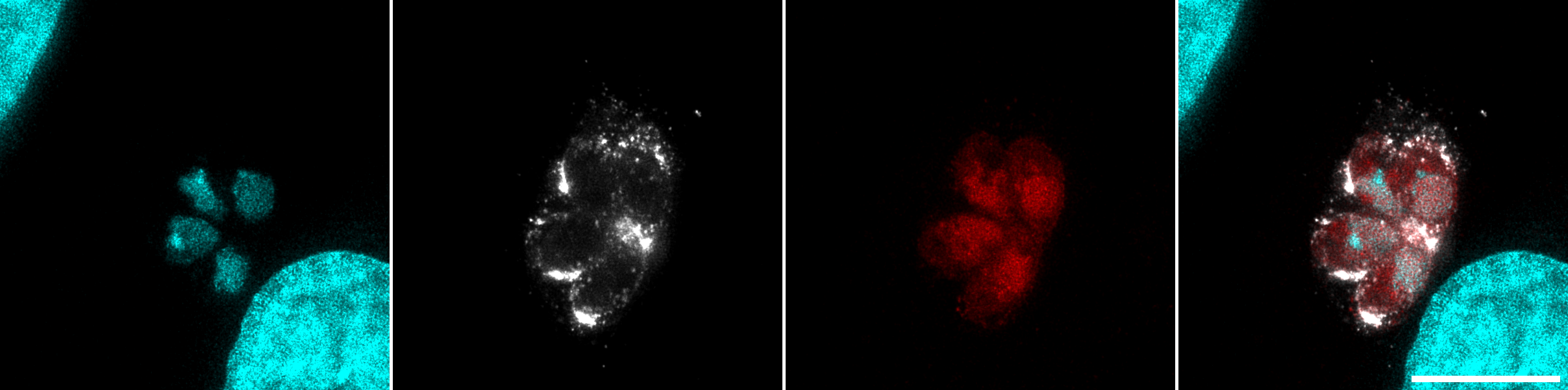

Supplement: Supplementary file 15 — Source data [file 41467_2025_58876_MOESM15_ESM.zip › Source suppl/Supplementary Figure 9_Source Data/Suppl Fig9c/Nc_MERGE_noGRA3_scale_montage.tif]

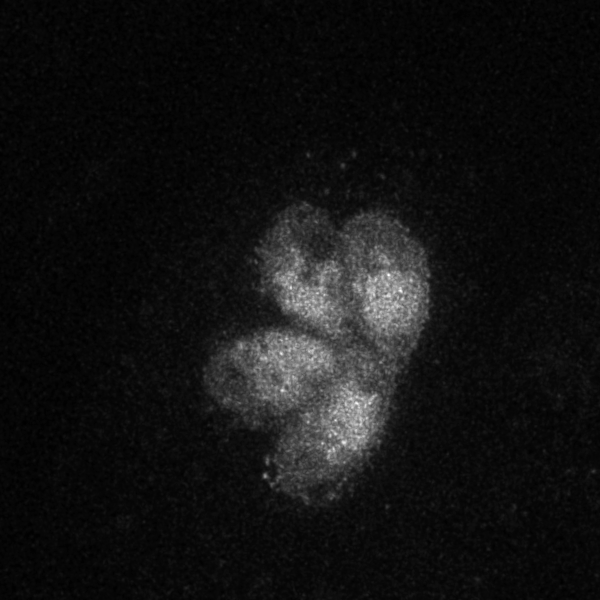

Supplement: Supplementary file 15 — Source data [file 41467_2025_58876_MOESM15_ESM.zip › Source suppl/Supplementary Figure 9_Source Data/Suppl Fig9c/FT_230301_Nc_HA488_GRA3_647_150x_A_2_MMStack_Pos0.ome_crop-0004.tif]

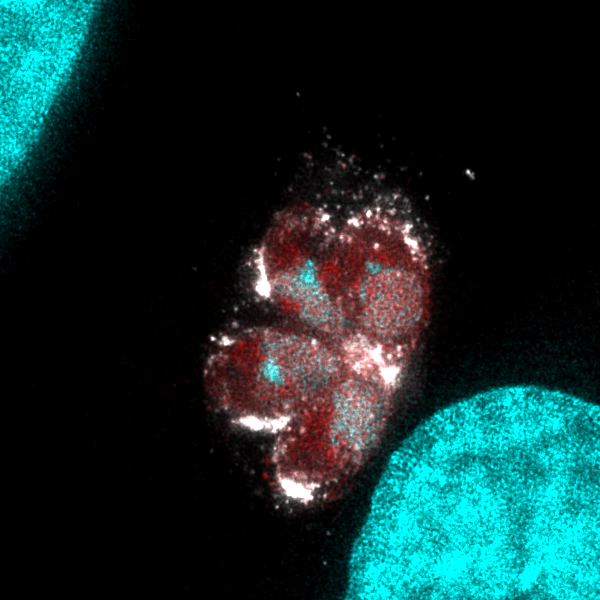

Supplement: Supplementary file 15 — Source data [file 41467_2025_58876_MOESM15_ESM.zip › Source suppl/Supplementary Figure 9_Source Data/Suppl Fig9c/Nc_MERGE_240304.tif]

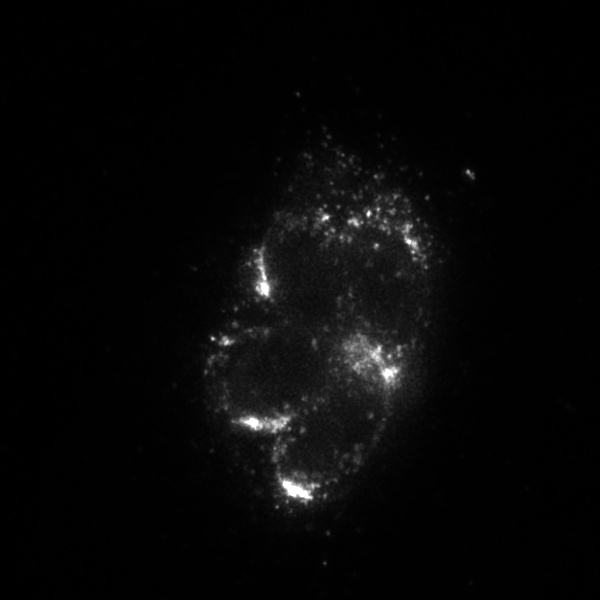

Supplement: Supplementary file 15 — Source data [file 41467_2025_58876_MOESM15_ESM.zip › Source suppl/Supplementary Figure 9_Source Data/Suppl Fig9c/FT_230301_Nc_HA488_GRA3_647_150x_A_2_MMStack_Pos0.ome_crop-0001.tif]

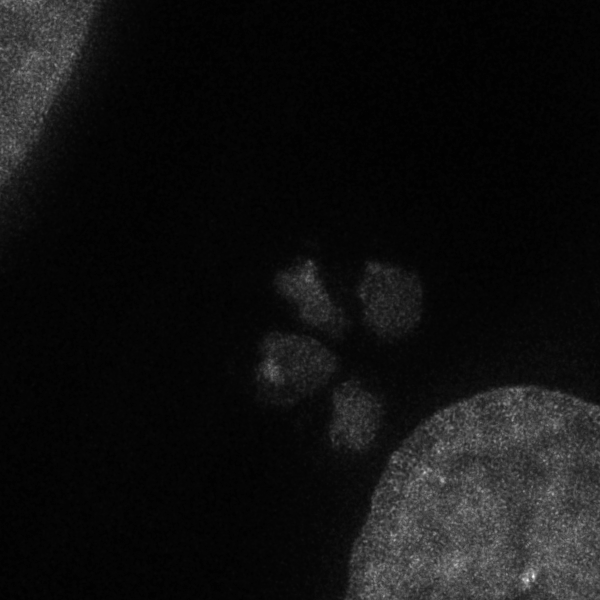

Supplement: Supplementary file 15 — Source data [file 41467_2025_58876_MOESM15_ESM.zip › Source suppl/Supplementary Figure 9_Source Data/Suppl Fig9c/FT_230301_Nc_HA488_GRA3_647_150x_A_2_MMStack_Pos0.ome_crop-0002.tif]

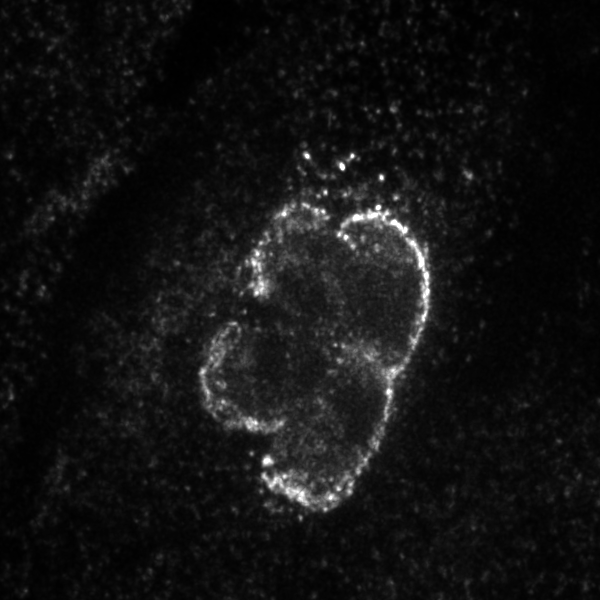

Supplement: Supplementary file 15 — Source data [file 41467_2025_58876_MOESM15_ESM.zip › Source suppl/Supplementary Figure 9_Source Data/Suppl Fig9c/FT_230301_Nc_HA488_GRA3_647_150x_A_2_MMStack_Pos0.ome_crop-0003.tif]

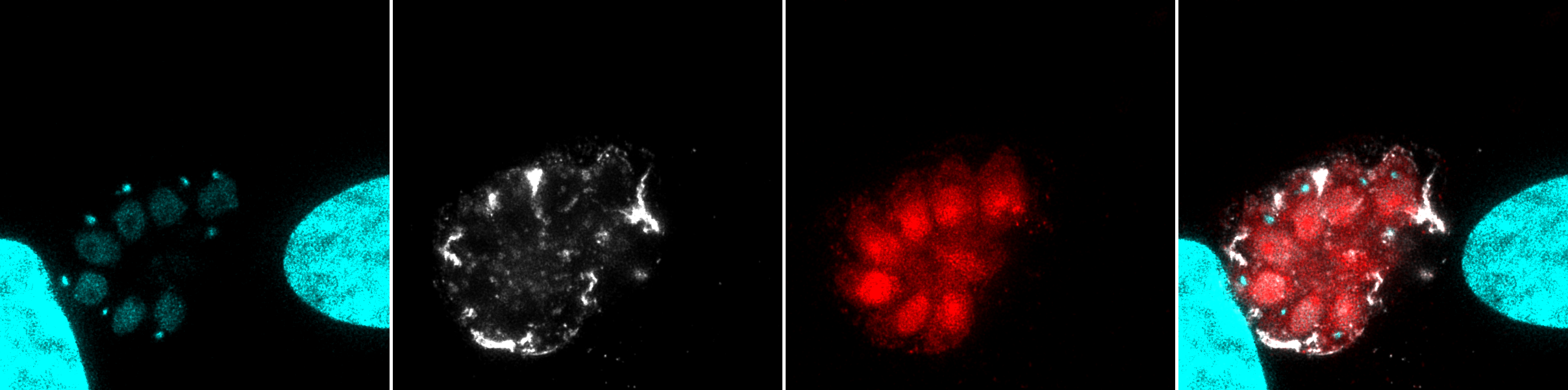

Supplement: Supplementary file 15 — Source data [file 41467_2025_58876_MOESM15_ESM.zip › Source suppl/Supplementary Figure 9_Source Data/Suppl Fig9c/Hh_MERGE_noGRA3.tif]

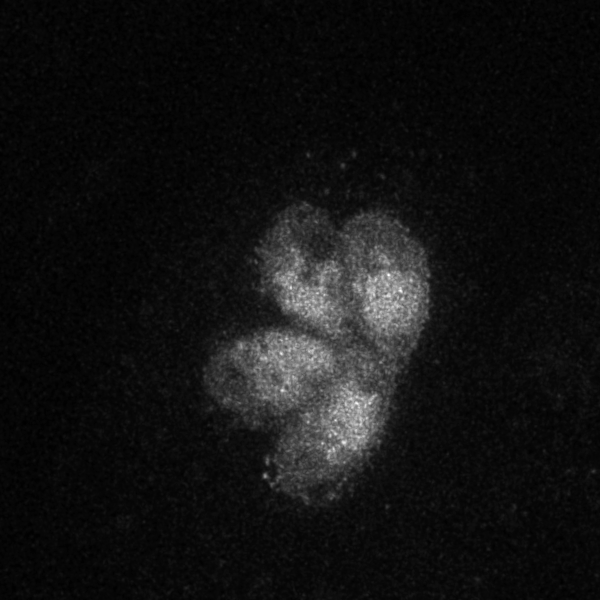

Supplement: Supplementary file 15 — Source data [file 41467_2025_58876_MOESM15_ESM.zip › Source suppl/Supplementary Figure 9_Source Data/Suppl Fig9c/FT_230301_Nc_HA488_GRA3_647_150x_A_2_MMStack_Pos0.ome_crop-0004_adj.tif]

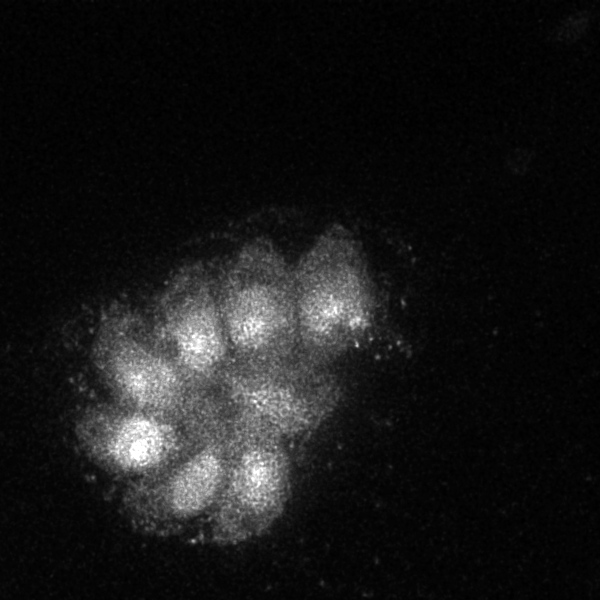

Supplement: Supplementary file 15 — Source data [file 41467_2025_58876_MOESM15_ESM.zip › Source suppl/Supplementary Figure 9_Source Data/Suppl Fig9c/FT_230301_hh_HA488_GRA3_647_150x_A_1_MMStack_Pos0.ome_crop-0004_adj.tif]

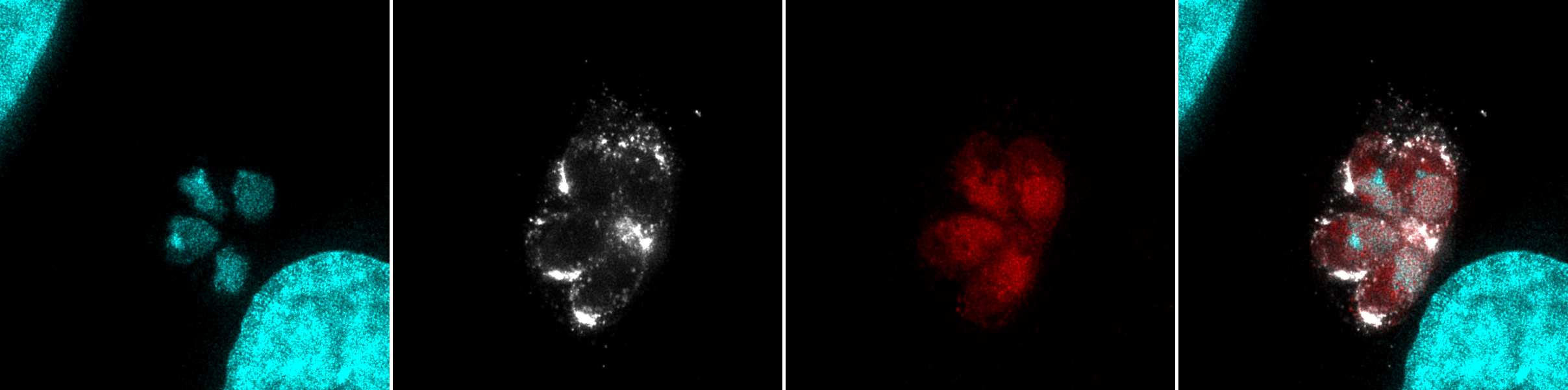

Supplement: Supplementary file 15 — Source data [file 41467_2025_58876_MOESM15_ESM.zip › Source suppl/Supplementary Figure 9_Source Data/Suppl Fig9c/Nc_MERGE_240304_MONTAGE.tif]

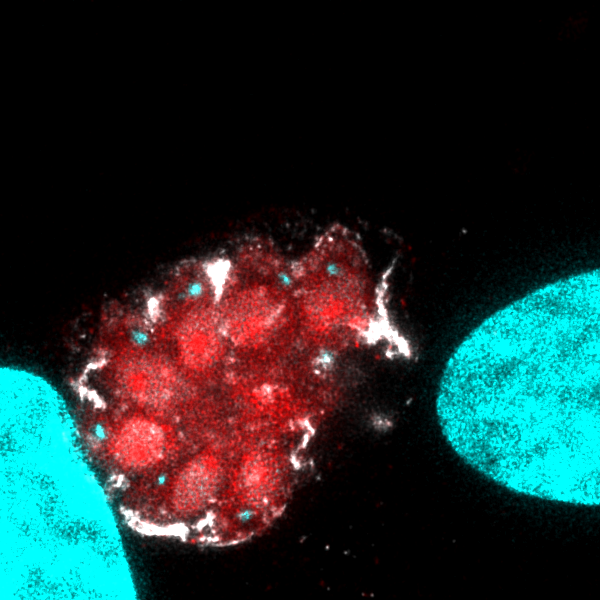

Supplement: Supplementary file 15 — Source data [file 41467_2025_58876_MOESM15_ESM.zip › Source suppl/Supplementary Figure 9_Source Data/Suppl Fig9c/Hh_MERGE_NEW_noGRA3.tif]

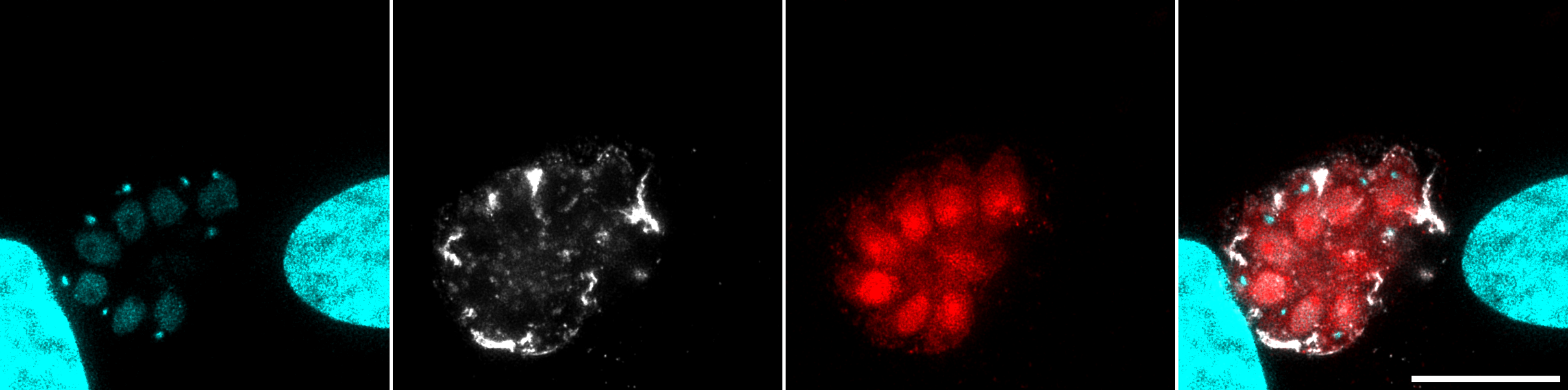

Supplement: Supplementary file 15 — Source data [file 41467_2025_58876_MOESM15_ESM.zip › Source suppl/Supplementary Figure 9_Source Data/Suppl Fig9c/Hh_MERGE_NEW_noGRA3_montage_scale.tif]

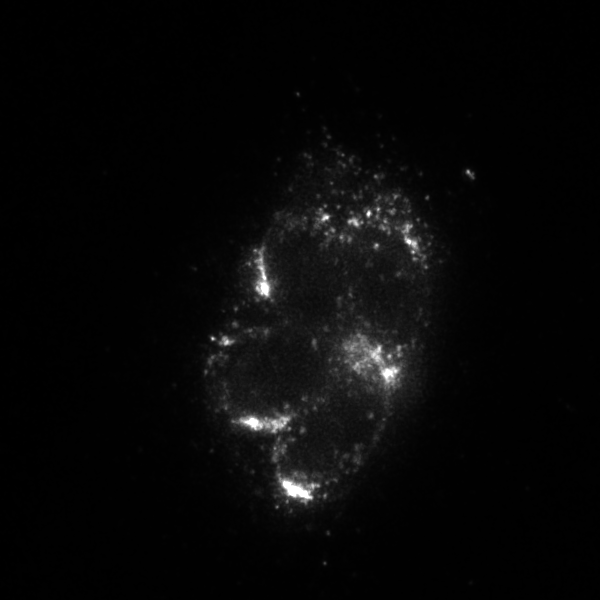

Supplement: Supplementary file 15 — Source data [file 41467_2025_58876_MOESM15_ESM.zip › Source suppl/Supplementary Figure 9_Source Data/Suppl Fig9c/FT_230301_Nc_HA488_GRA3_647_150x_A_2_MMStack_Pos0.ome_crop-0001_adj.tif]

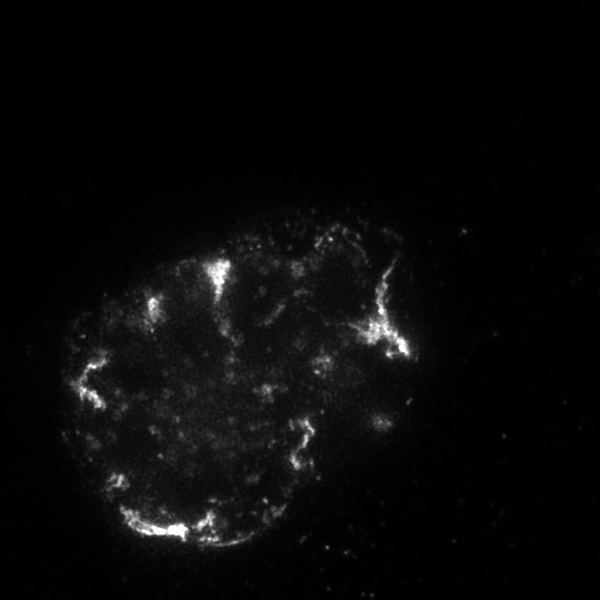

Supplement: Supplementary file 15 — Source data [file 41467_2025_58876_MOESM15_ESM.zip › Source suppl/Supplementary Figure 9_Source Data/Suppl Fig9c/FT_230301_hh_HA488_GRA3_647_150x_A_1_MMStack_Pos0.ome_crop-0001_adj.tif]

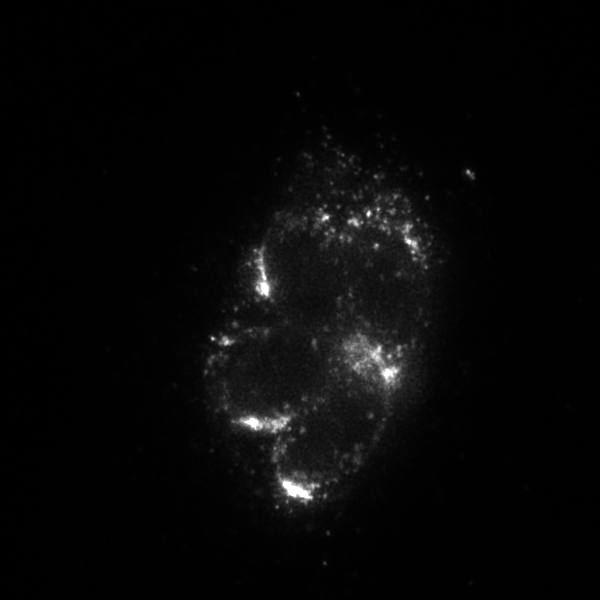

Supplement: Supplementary file 15 — Source data [file 41467_2025_58876_MOESM15_ESM.zip › Source suppl/Supplementary Figure 9_Source Data/Suppl Fig9c/FT_230301_Nc_HA488_GRA3_647_150x_A_2_MMStack_Pos0.ome_crop.tif]

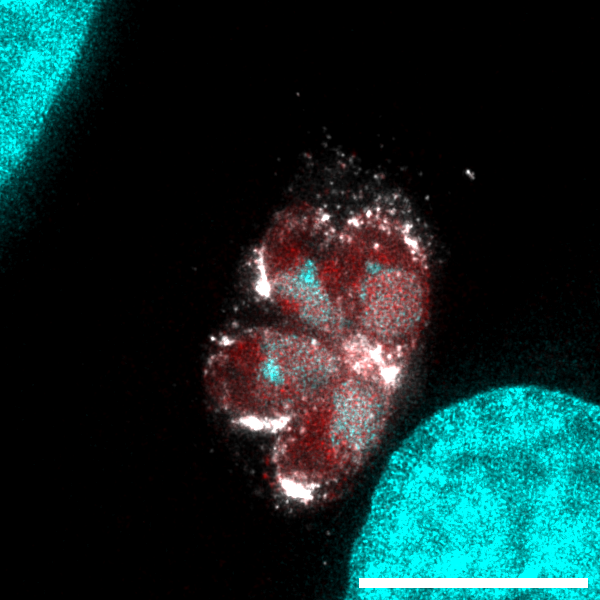

Supplement: Supplementary file 15 — Source data [file 41467_2025_58876_MOESM15_ESM.zip › Source suppl/Supplementary Figure 9_Source Data/Suppl Fig9c/Nc_MERGE_noGRA3_scale.tif]

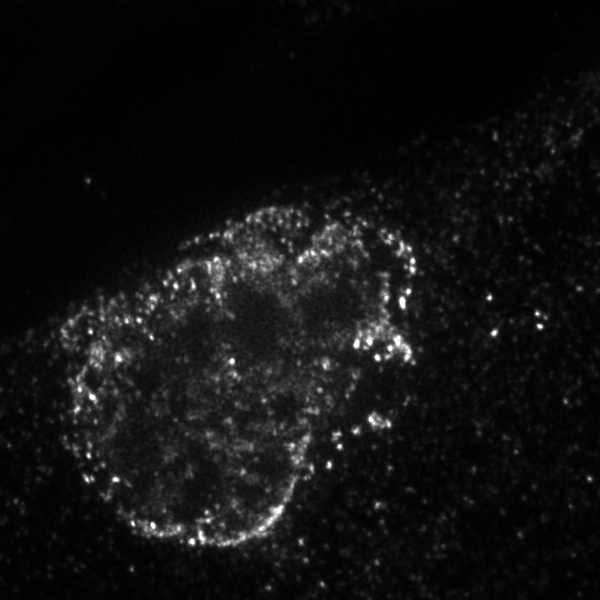

Supplement: Supplementary file 15 — Source data [file 41467_2025_58876_MOESM15_ESM.zip › Source suppl/Supplementary Figure 9_Source Data/Suppl Fig9c/FT_230301_hh_HA488_GRA3_647_150x_A_1_MMStack_Pos0.ome_crop-0003_adj.tif]

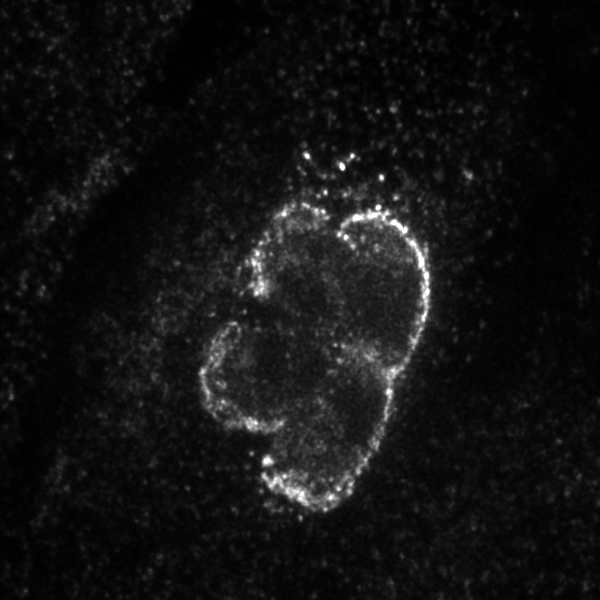

Supplement: Supplementary file 15 — Source data [file 41467_2025_58876_MOESM15_ESM.zip › Source suppl/Supplementary Figure 9_Source Data/Suppl Fig9c/FT_230301_Nc_HA488_GRA3_647_150x_A_2_MMStack_Pos0.ome_crop-0003_adj.tif]

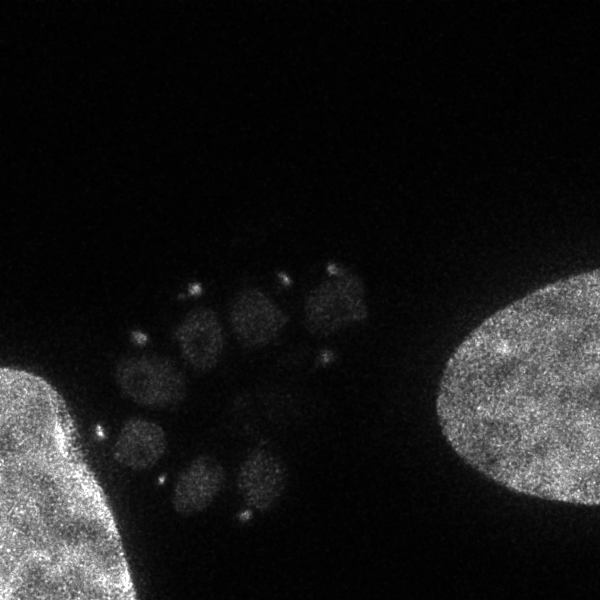

Supplement: Supplementary file 15 — Source data [file 41467_2025_58876_MOESM15_ESM.zip › Source suppl/Supplementary Figure 9_Source Data/Suppl Fig9c/FT_230301_hh_HA488_GRA3_647_150x_A_1_MMStack_Pos0.ome_crop-0002_adj.tif]

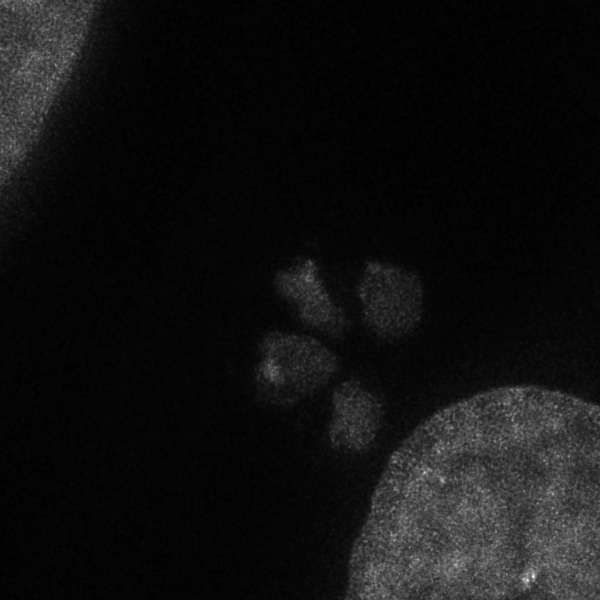

Supplement: Supplementary file 15 — Source data [file 41467_2025_58876_MOESM15_ESM.zip › Source suppl/Supplementary Figure 9_Source Data/Suppl Fig9c/FT_230301_Nc_HA488_GRA3_647_150x_A_2_MMStack_Pos0.ome_crop-0002_adj.tif]

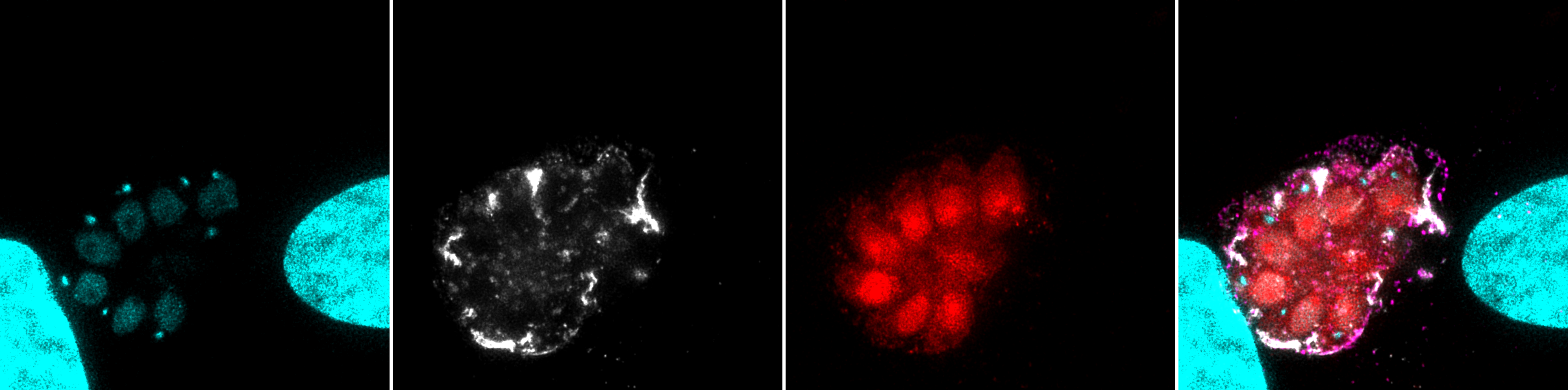

Supplement: Supplementary file 15 — Source data [file 41467_2025_58876_MOESM15_ESM.zip › Source suppl/Supplementary Figure 9_Source Data/Suppl Fig9c/HH_montage_NEW.tif]

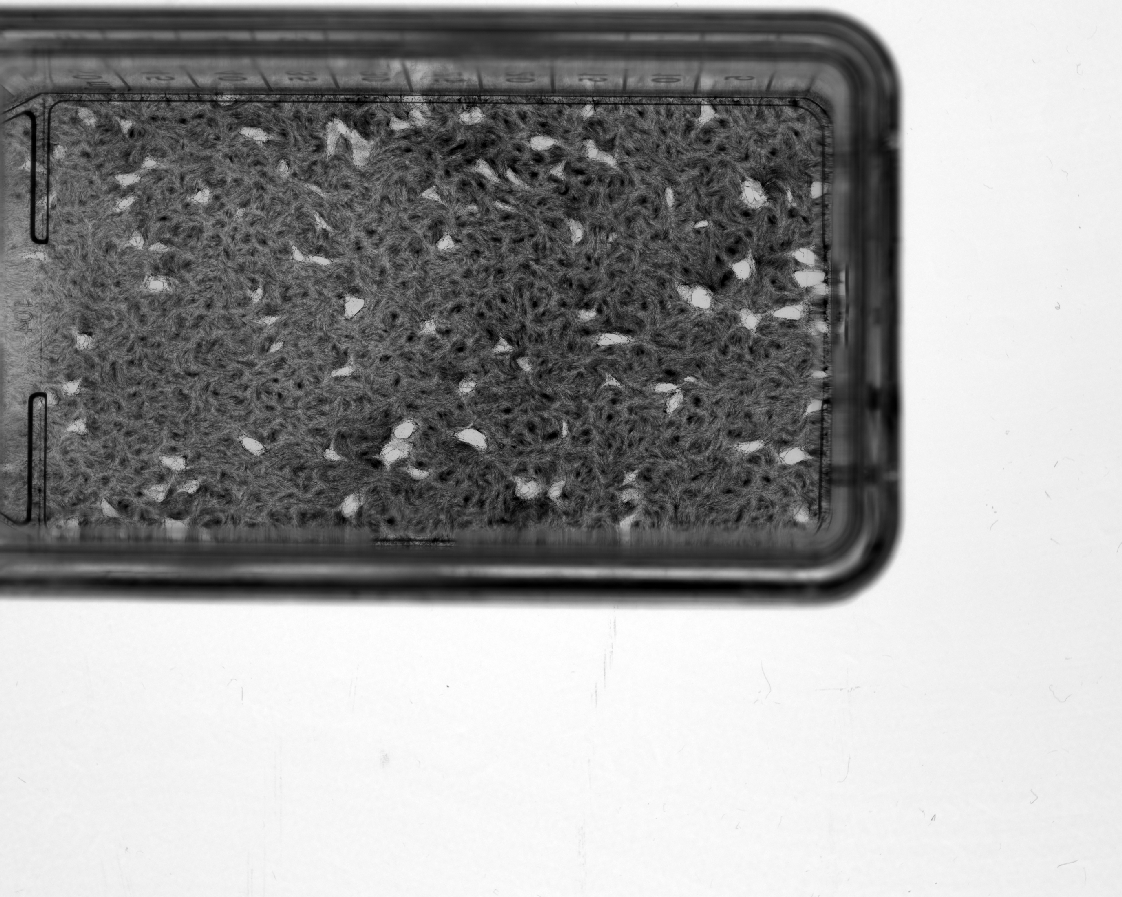

Supplement: Supplementary file 15 — Source data [file 41467_2025_58876_MOESM15_ESM.zip › Source suppl/Supplementary Figure 9_Source Data/Suppl Fig9b/rh dGRA COMPL NC PL200(Silver Stain).jpg]

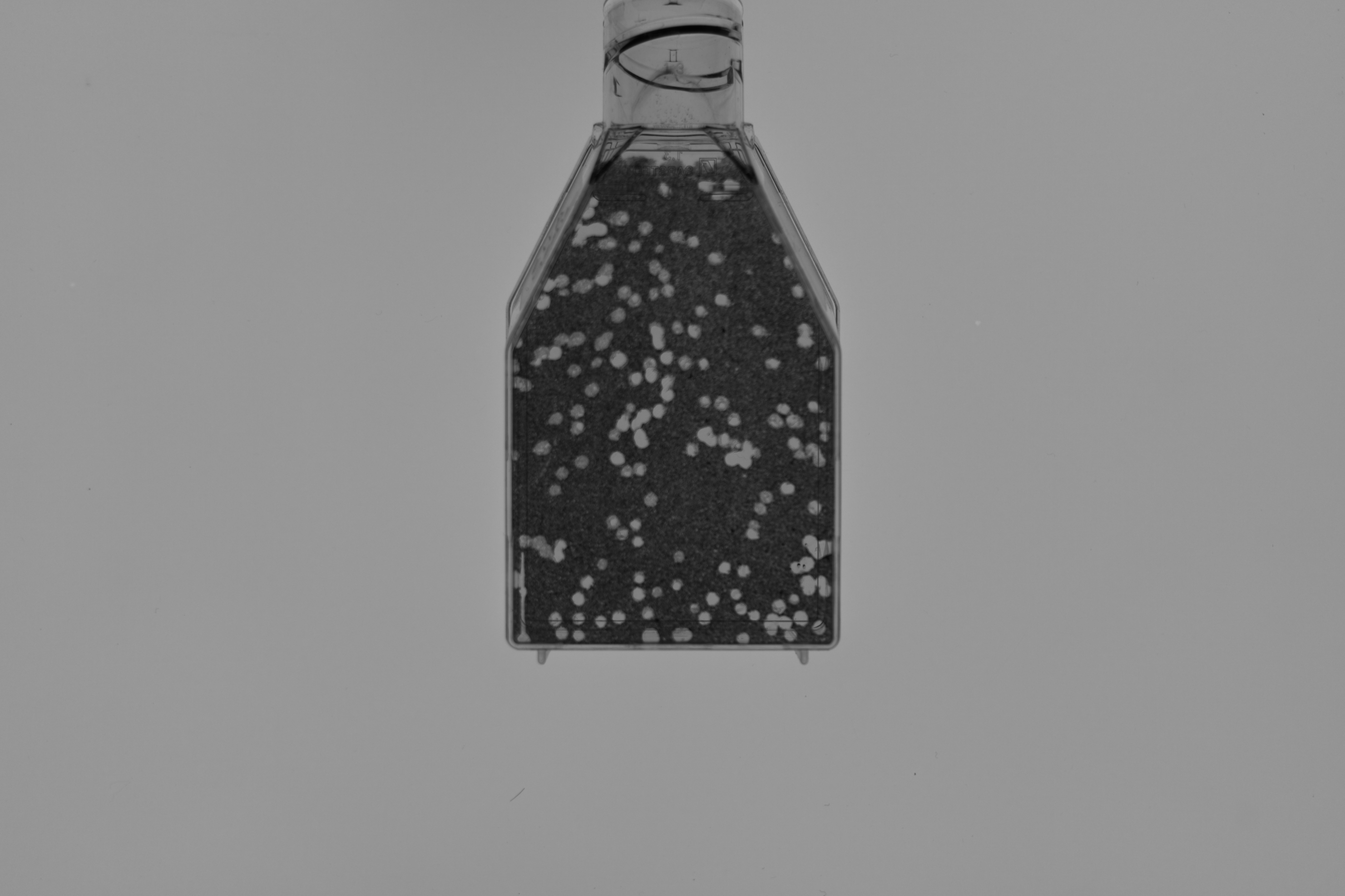

Supplement: Supplementary file 15 — Source data [file 41467_2025_58876_MOESM15_ESM.zip › Source suppl/Supplementary Figure 9_Source Data/Suppl Fig9b/igcuser 2024-12-16 16h55m48s(Silver Stain).raw16.tif]

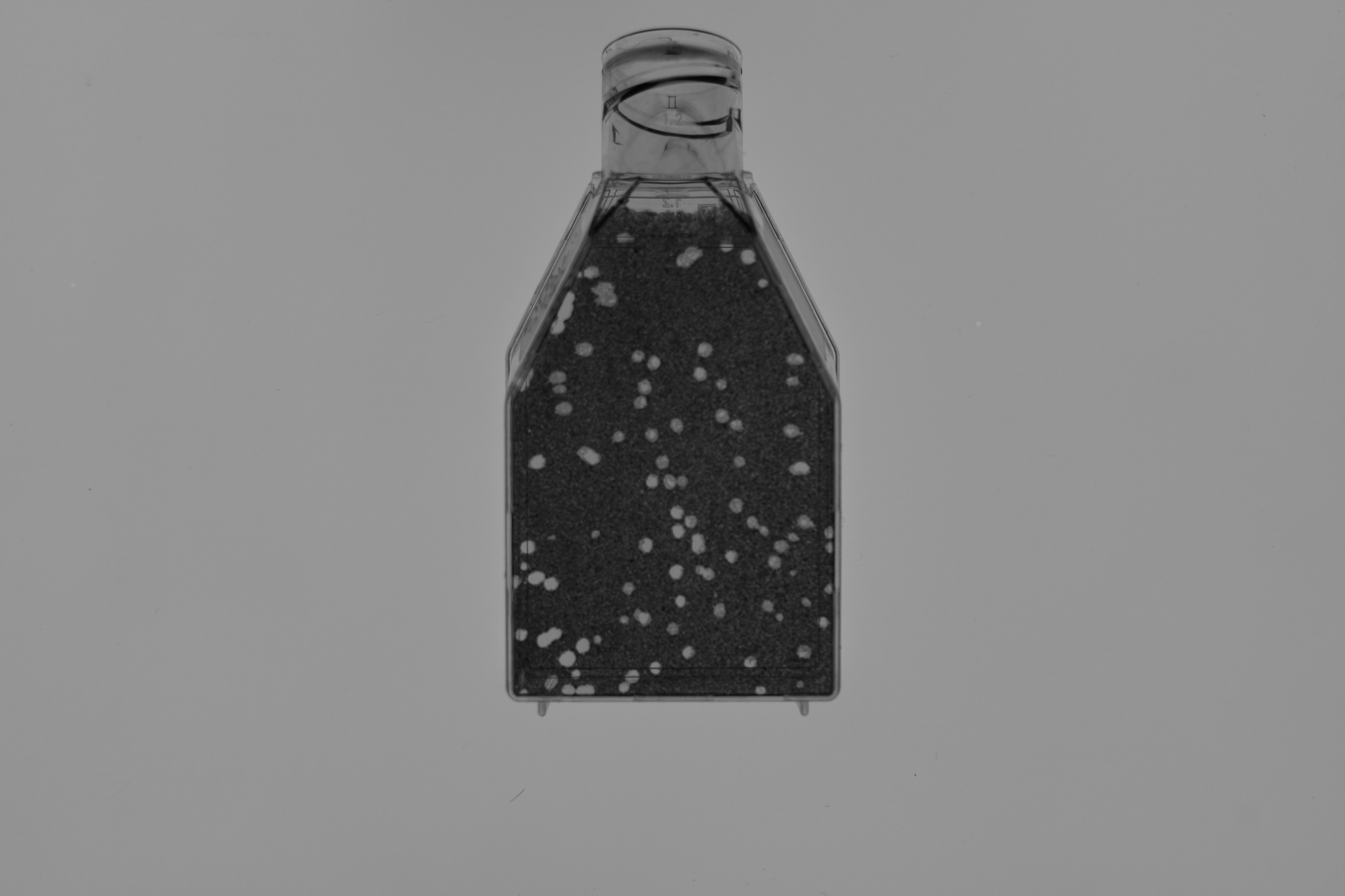

Supplement: Supplementary file 15 — Source data [file 41467_2025_58876_MOESM15_ESM.zip › Source suppl/Supplementary Figure 9_Source Data/Suppl Fig9b/igcuser 2024-12-16 17h00m30s(Silver Stain).raw16.tif]

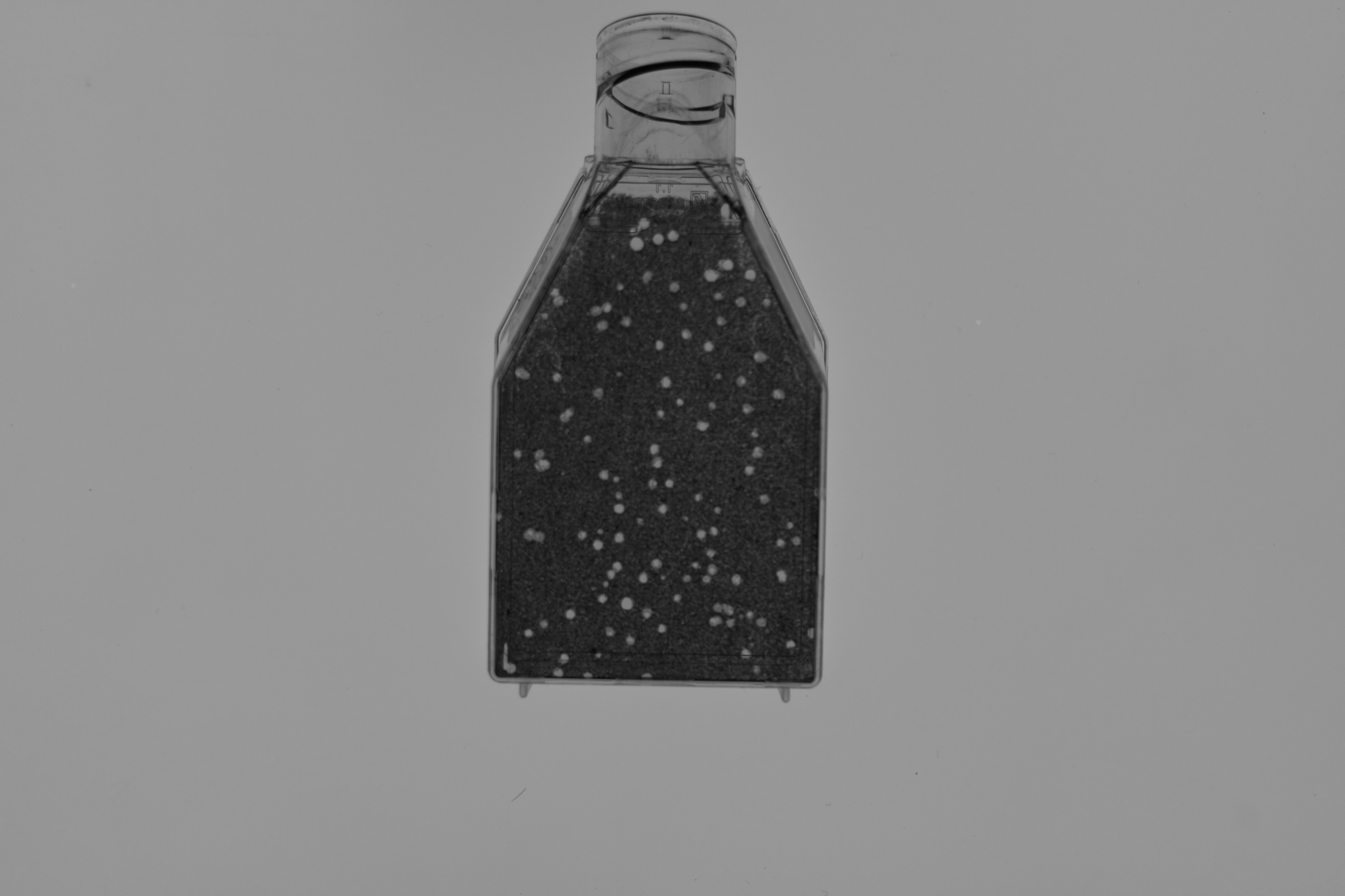

Supplement: Supplementary file 15 — Source data [file 41467_2025_58876_MOESM15_ESM.zip › Source suppl/Supplementary Figure 9_Source Data/Suppl Fig9b/igcuser 2024-12-16 17h01m36s(Silver Stain).raw16.tif]

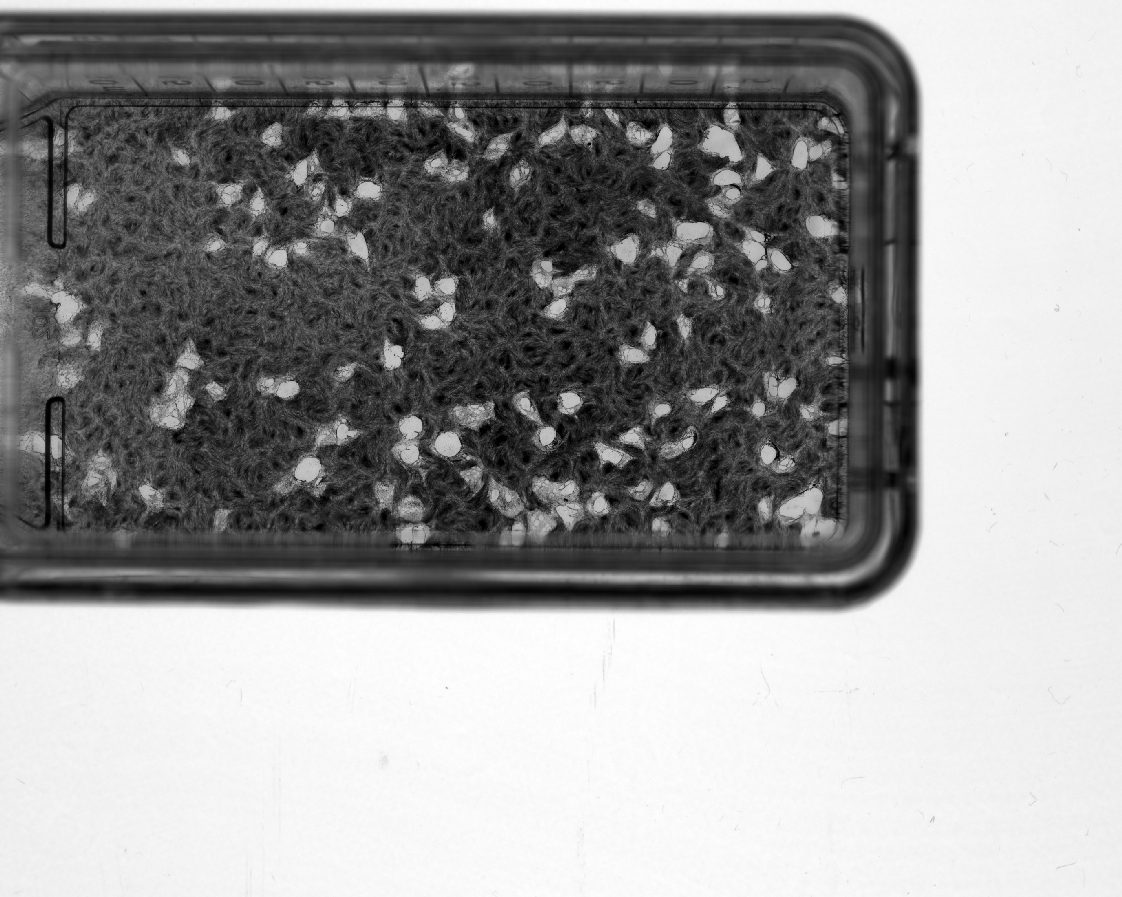

Supplement: Supplementary file 15 — Source data [file 41467_2025_58876_MOESM15_ESM.zip › Source suppl/Supplementary Figure 9_Source Data/Suppl Fig9b/rh dGRA COMPL HH PL100(Silver Stain).jpg]

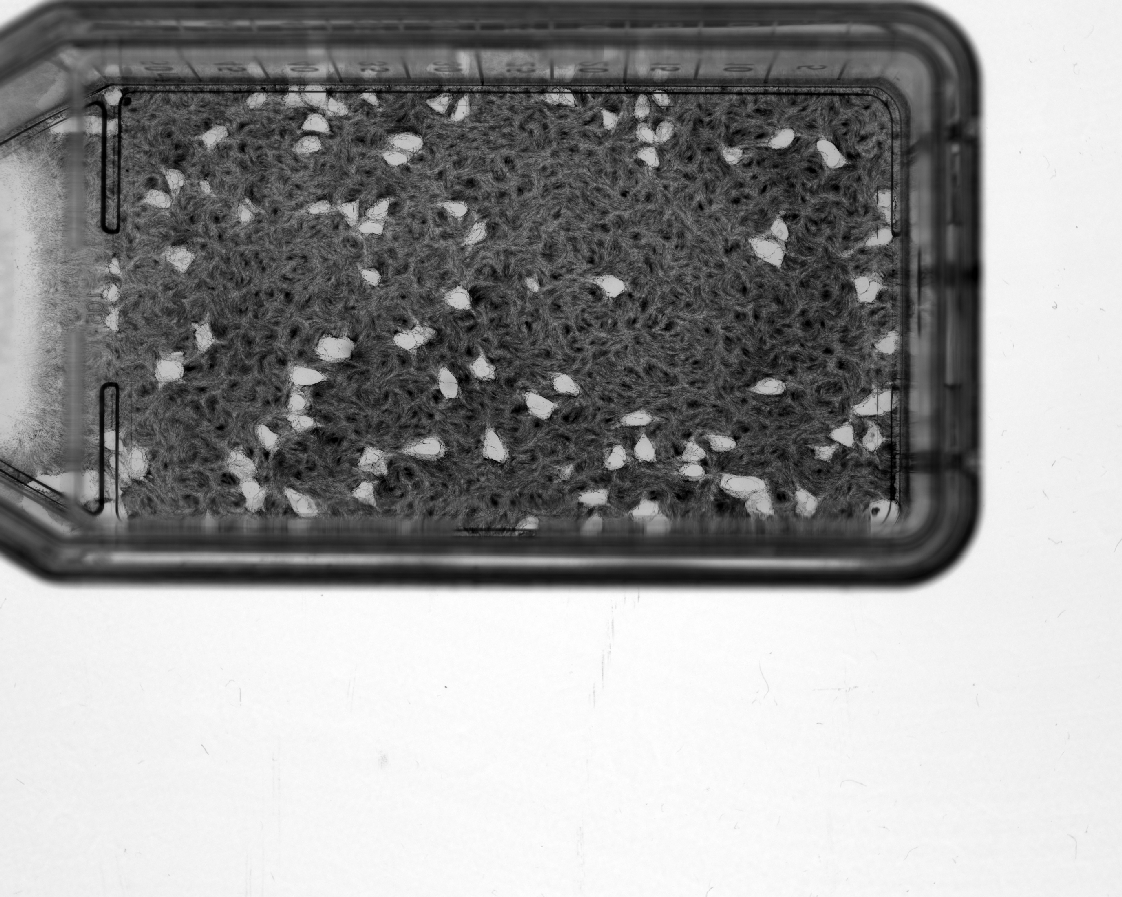

Supplement: Supplementary file 15 — Source data [file 41467_2025_58876_MOESM15_ESM.zip › Source suppl/Supplementary Figure 9_Source Data/Suppl Fig9b/rh dGRA PL 100(Silver Stain).jpg]

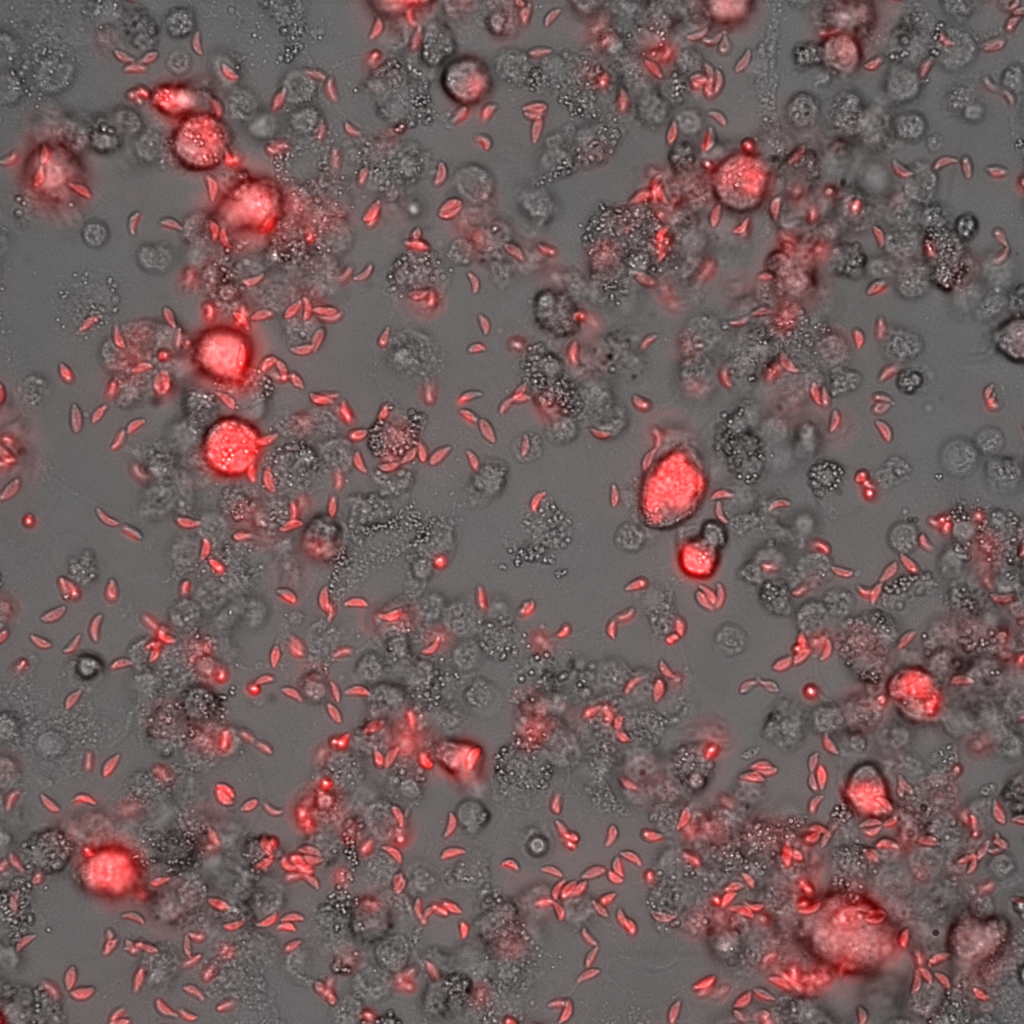

Supplement: Supplementary file 15 — Source data [file 41467_2025_58876_MOESM15_ESM.zip › Source suppl/Supplementary Figure 5_Source Data/Suppl Fig5d/BMDM_IFNG_GRA12_MERGE.tif]

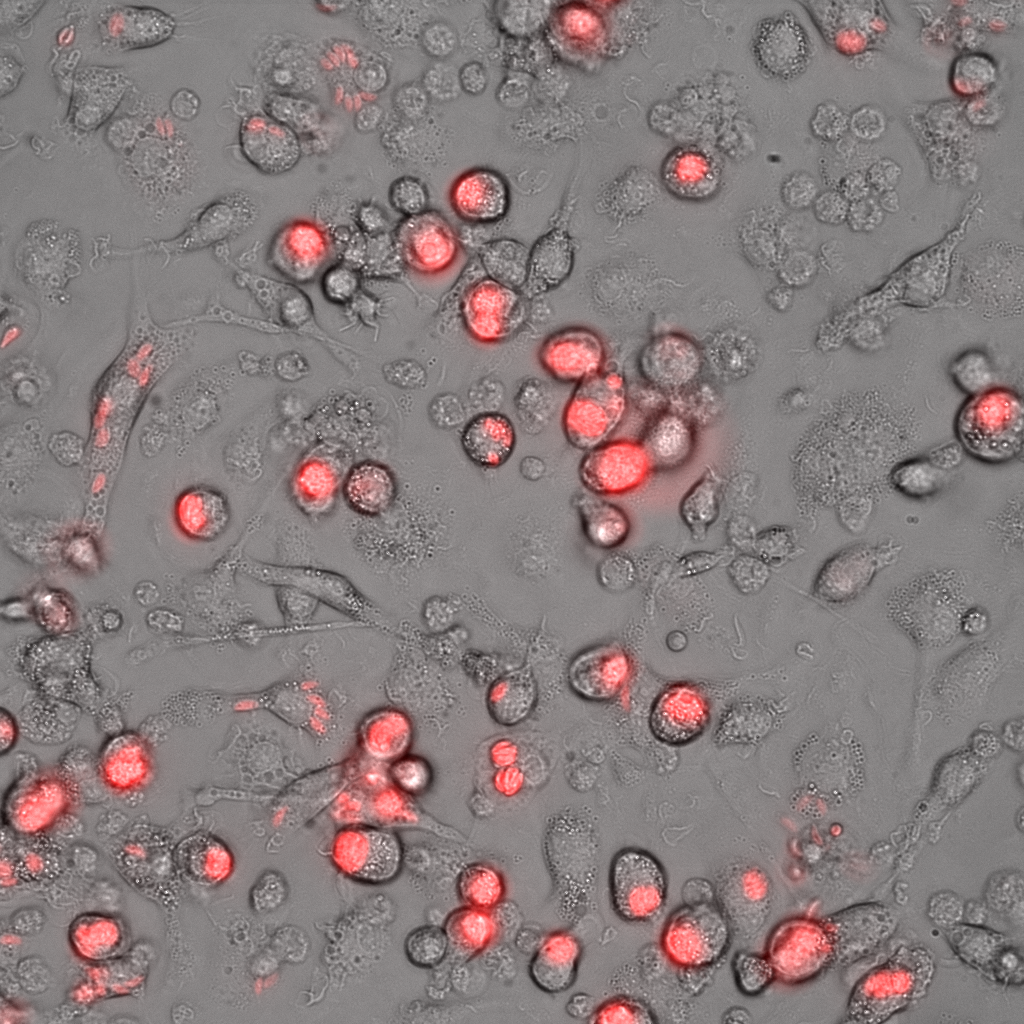

Supplement: Supplementary file 15 — Source data [file 41467_2025_58876_MOESM15_ESM.zip › Source suppl/Supplementary Figure 5_Source Data/Suppl Fig5d/BMDM_noIFNG_COMPL_MERGE.tif]

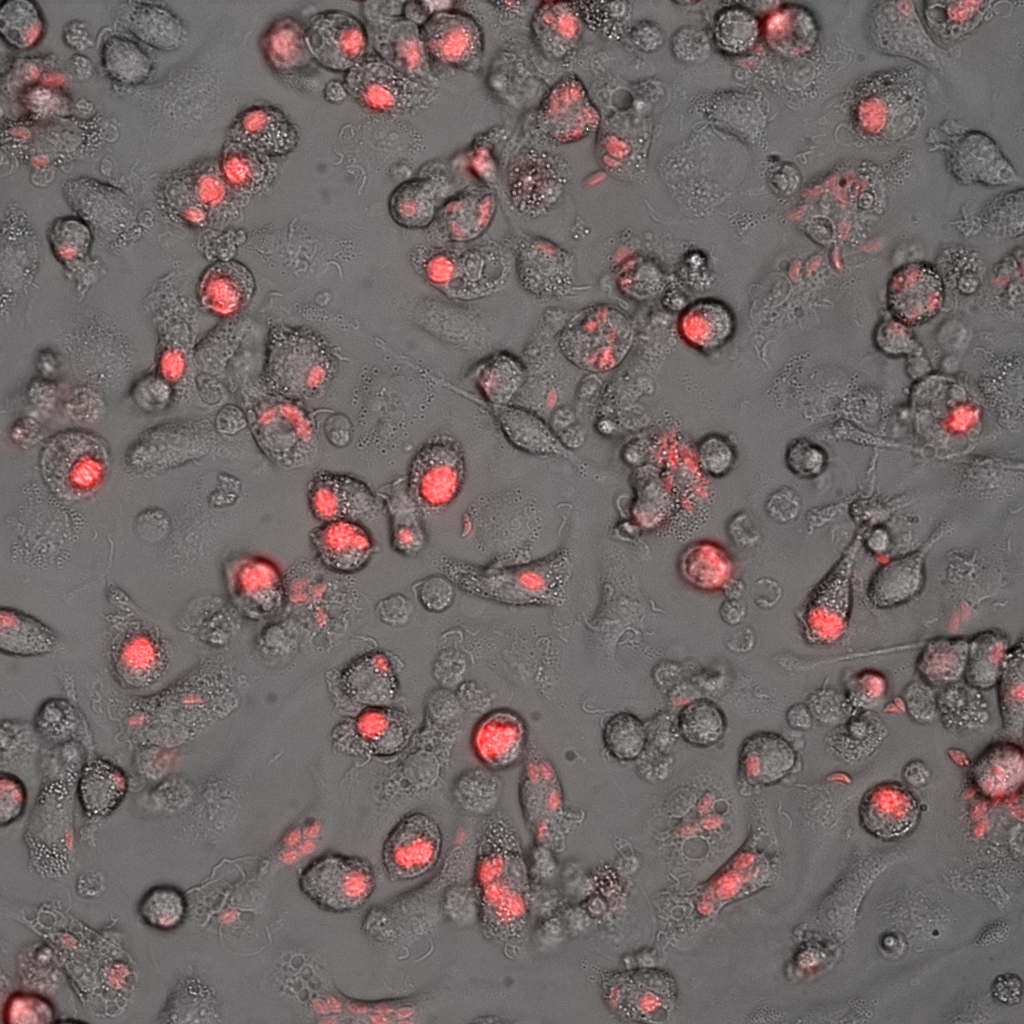

Supplement: Supplementary file 15 — Source data [file 41467_2025_58876_MOESM15_ESM.zip › Source suppl/Supplementary Figure 5_Source Data/Suppl Fig5d/BMDM_noIFNG_GRA12_MERGE.tif]

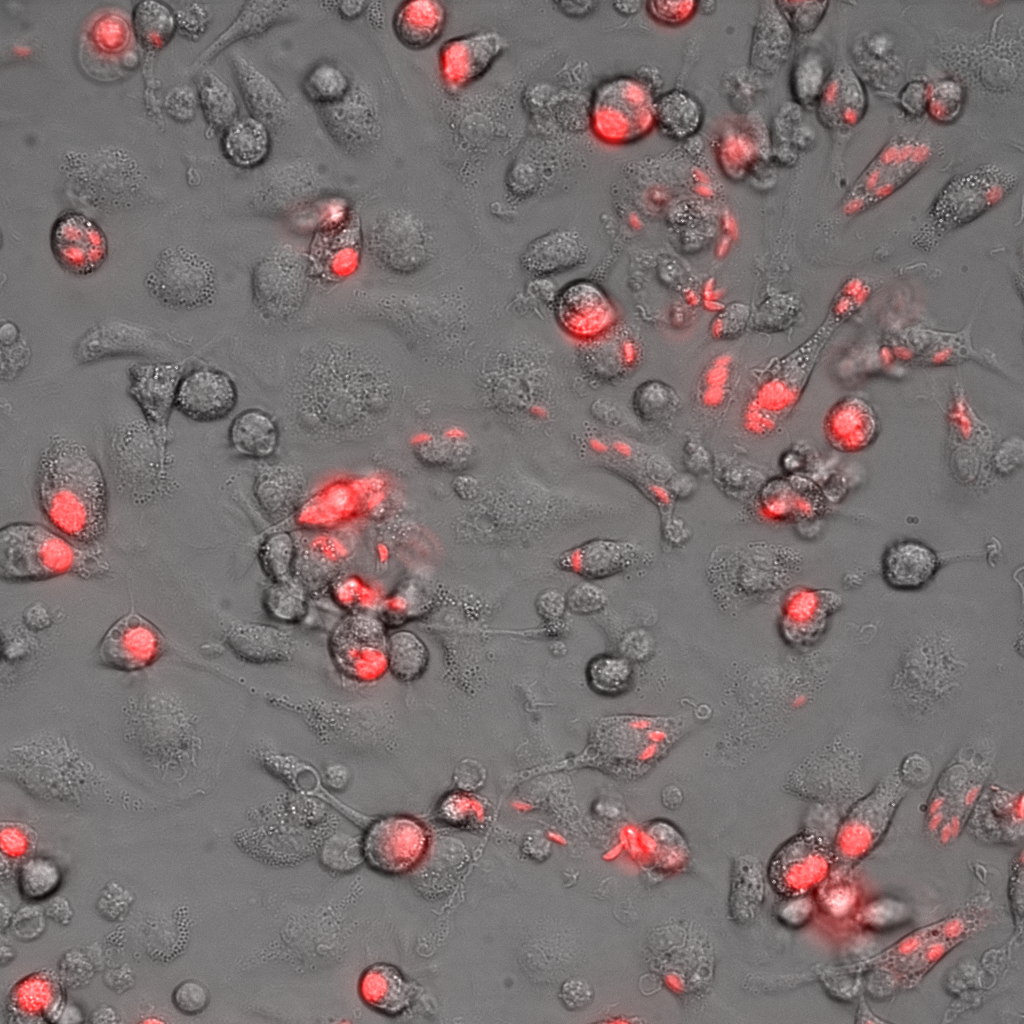

Supplement: Supplementary file 15 — Source data [file 41467_2025_58876_MOESM15_ESM.zip › Source suppl/Supplementary Figure 5_Source Data/Suppl Fig5d/BMDM_noIFNG_UPRT_MERGE.tif]

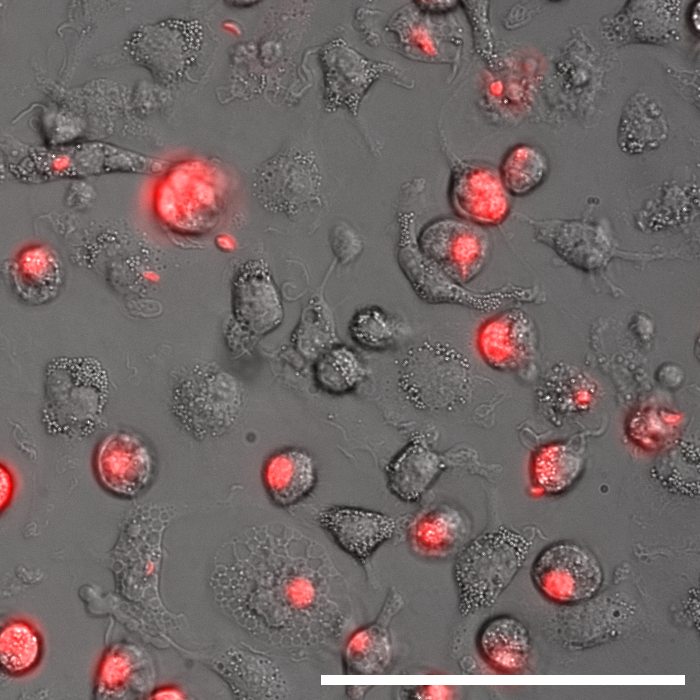

Supplement: Supplementary file 15 — Source data [file 41467_2025_58876_MOESM15_ESM.zip › Source suppl/Supplementary Figure 5_Source Data/Suppl Fig5d/BMDM_IFNG_UPRT_MERGE_scale.png]

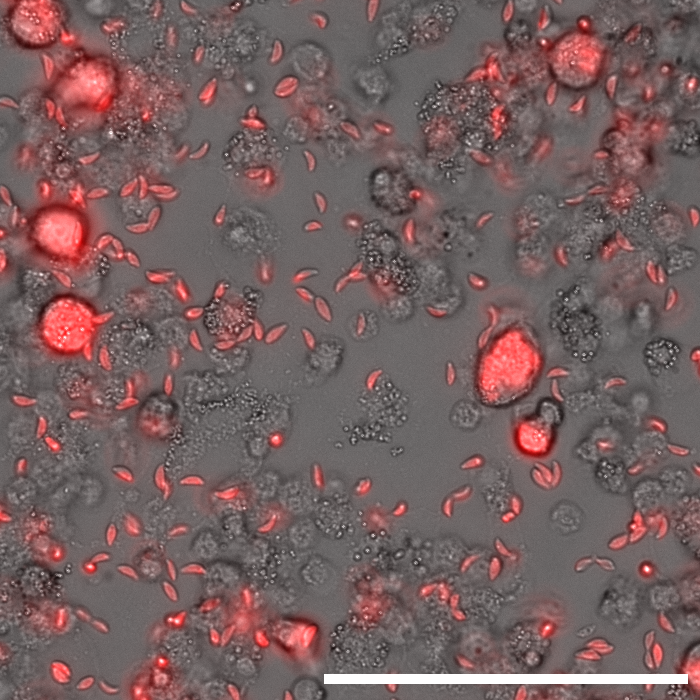

Supplement: Supplementary file 15 — Source data [file 41467_2025_58876_MOESM15_ESM.zip › Source suppl/Supplementary Figure 5_Source Data/Suppl Fig5d/BMDM_IFNG_GRA12_MERGE_scale.png]

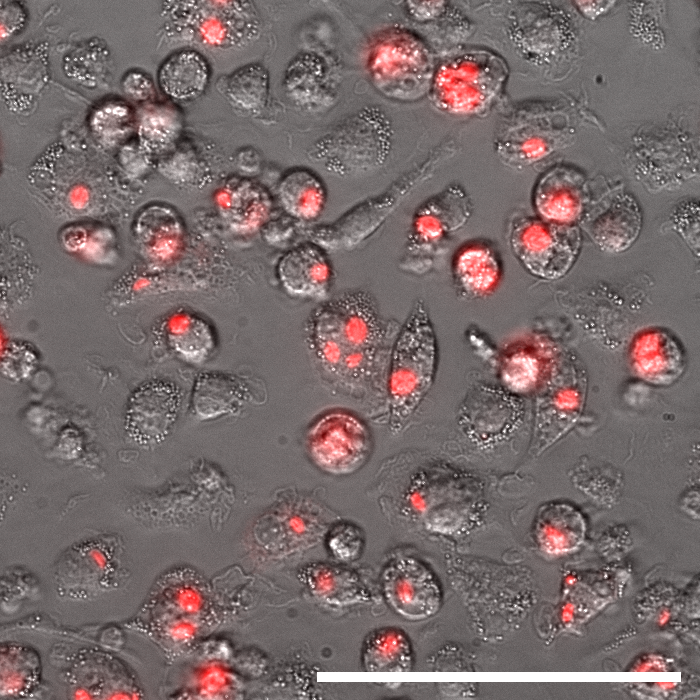

Supplement: Supplementary file 15 — Source data [file 41467_2025_58876_MOESM15_ESM.zip › Source suppl/Supplementary Figure 5_Source Data/Suppl Fig5d/BMDM_IFNG_COMPL_MERGE_scale.png]

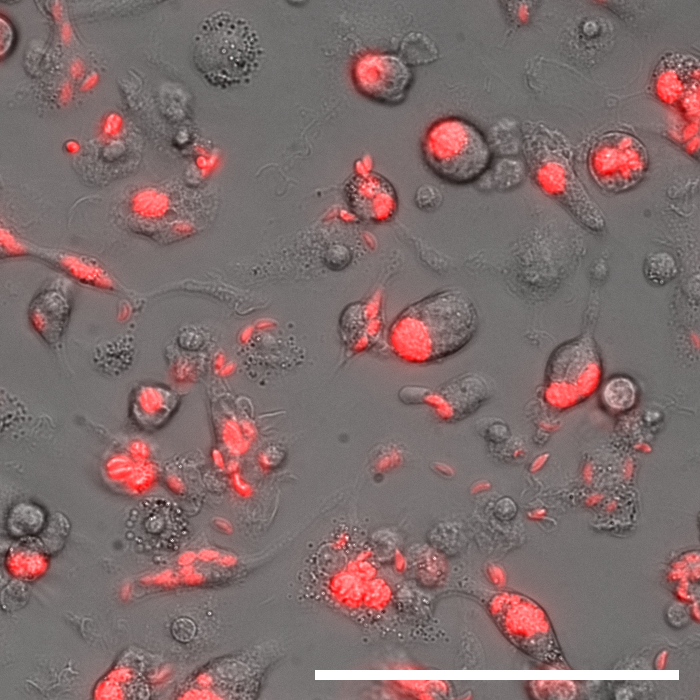

Supplement: Supplementary file 15 — Source data [file 41467_2025_58876_MOESM15_ESM.zip › Source suppl/Supplementary Figure 5_Source Data/Suppl Fig5d/BMDM_noIFNG_UPRT_MERGE_scale_2.png]

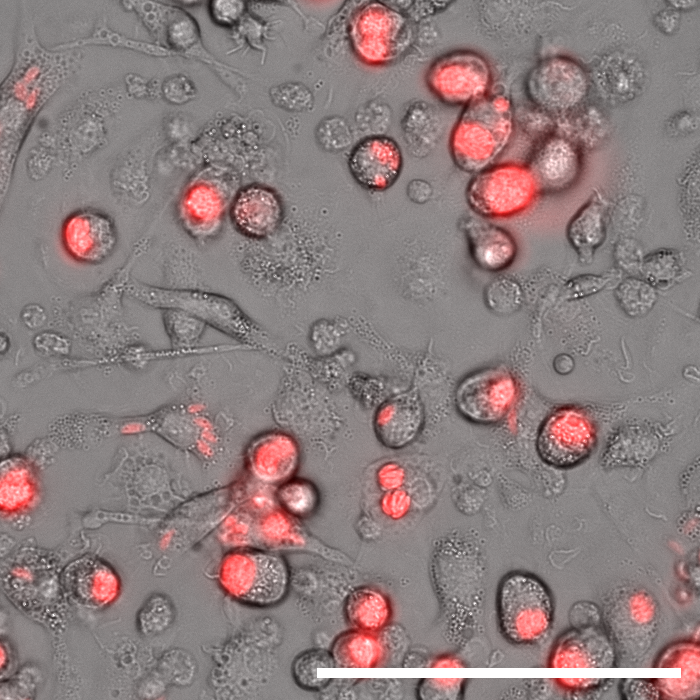

Supplement: Supplementary file 15 — Source data [file 41467_2025_58876_MOESM15_ESM.zip › Source suppl/Supplementary Figure 5_Source Data/Suppl Fig5d/BMDM_noIFNG_COMPL_MERGE_scale.png]

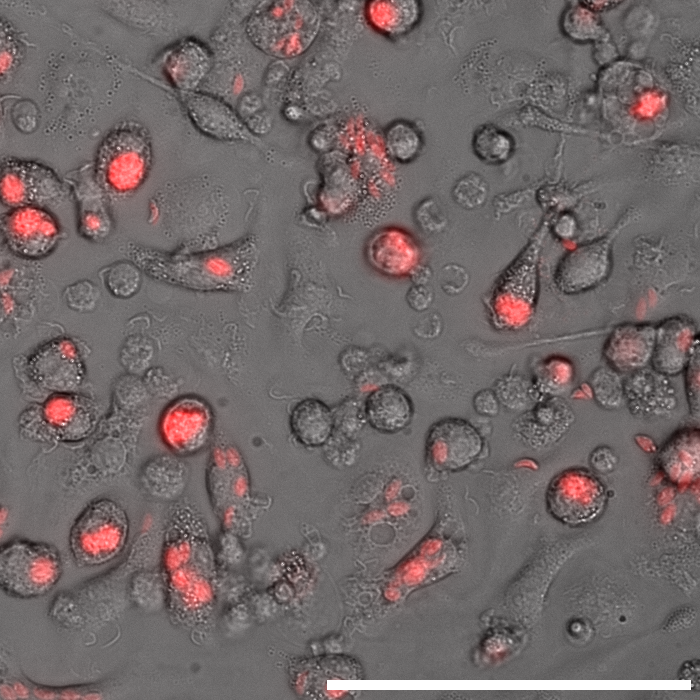

Supplement: Supplementary file 15 — Source data [file 41467_2025_58876_MOESM15_ESM.zip › Source suppl/Supplementary Figure 5_Source Data/Suppl Fig5d/BMDM_noIFNG_GRA12_MERGE-1_scale.png]

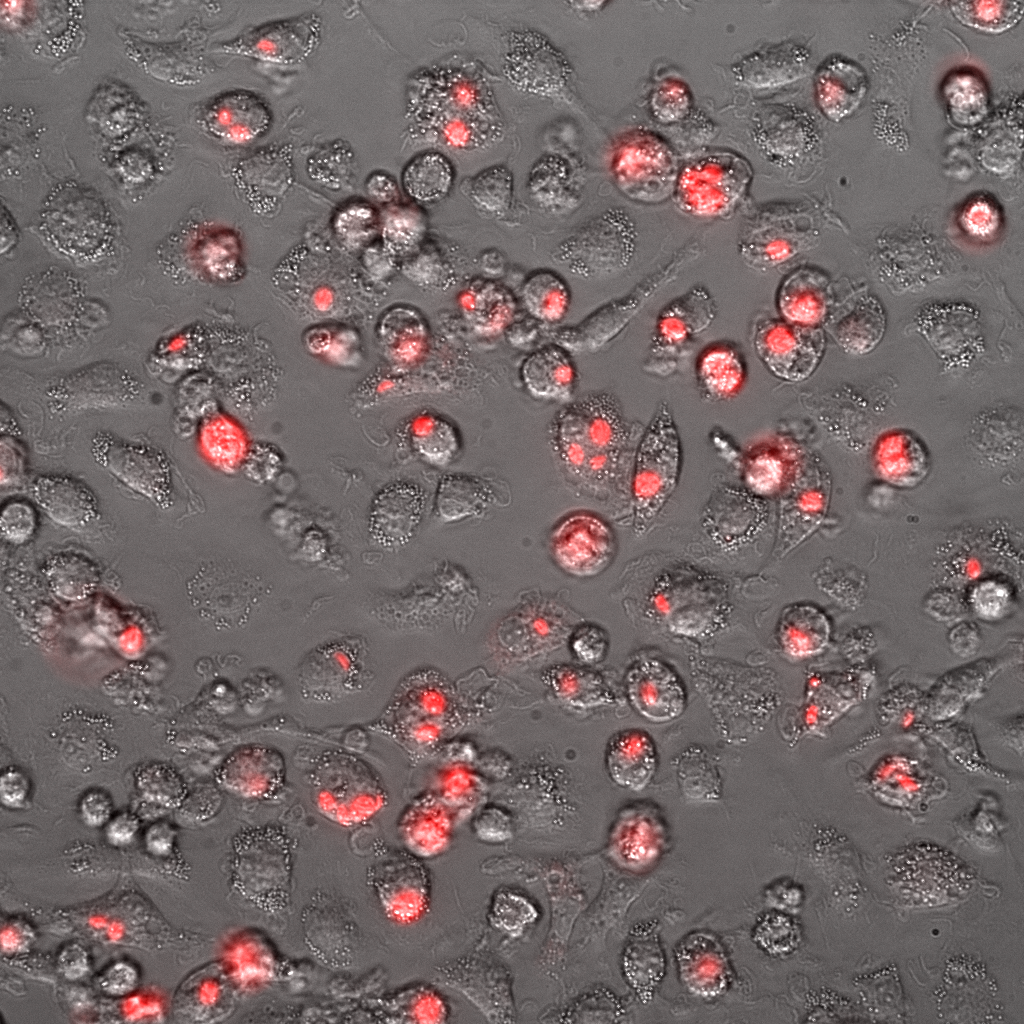

Supplement: Supplementary file 15 — Source data [file 41467_2025_58876_MOESM15_ESM.zip › Source suppl/Supplementary Figure 5_Source Data/Suppl Fig5d/BMDM_IFNG_COMPL_MERGE.tif]

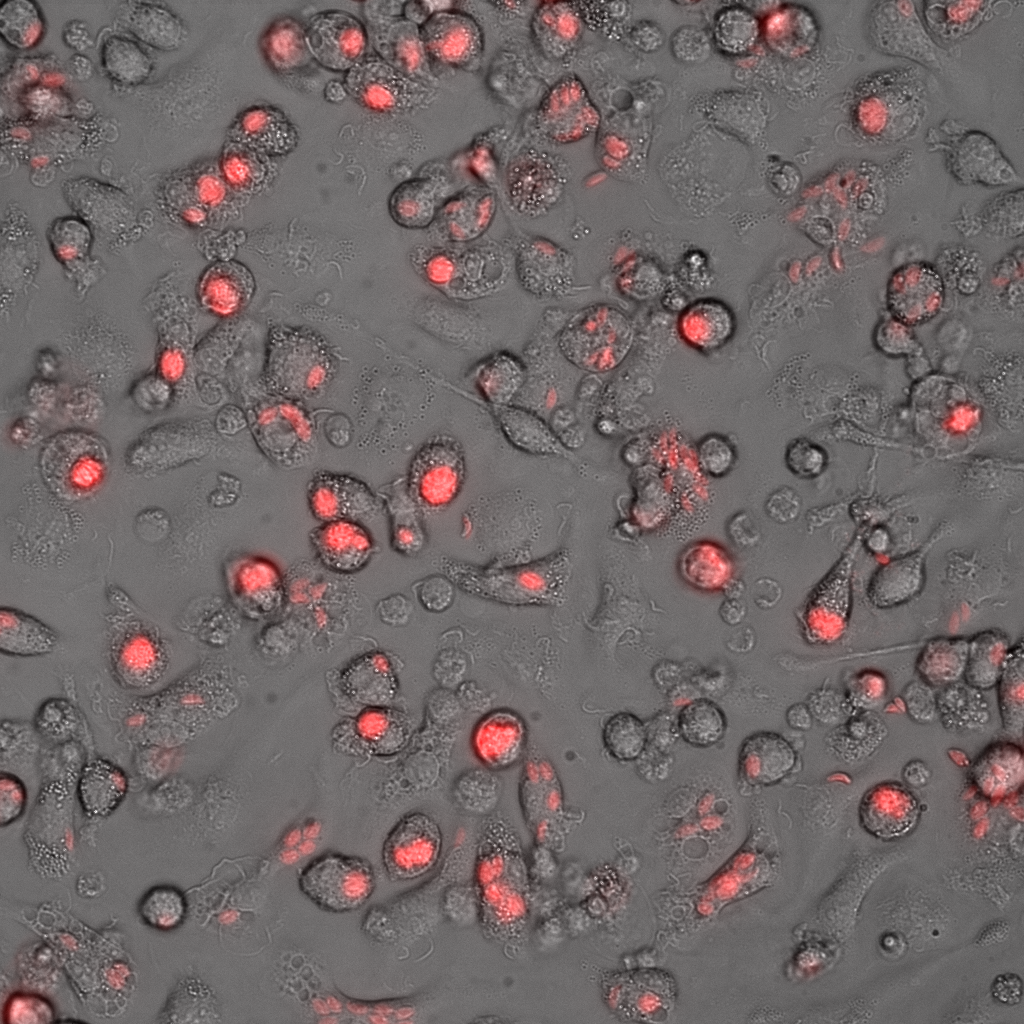

Supplement: Supplementary file 15 — Source data [file 41467_2025_58876_MOESM15_ESM.zip › Source suppl/Supplementary Figure 5_Source Data/Suppl Fig5d/BMDM_noIFNG_GRA12_MERGE_scale.tif]

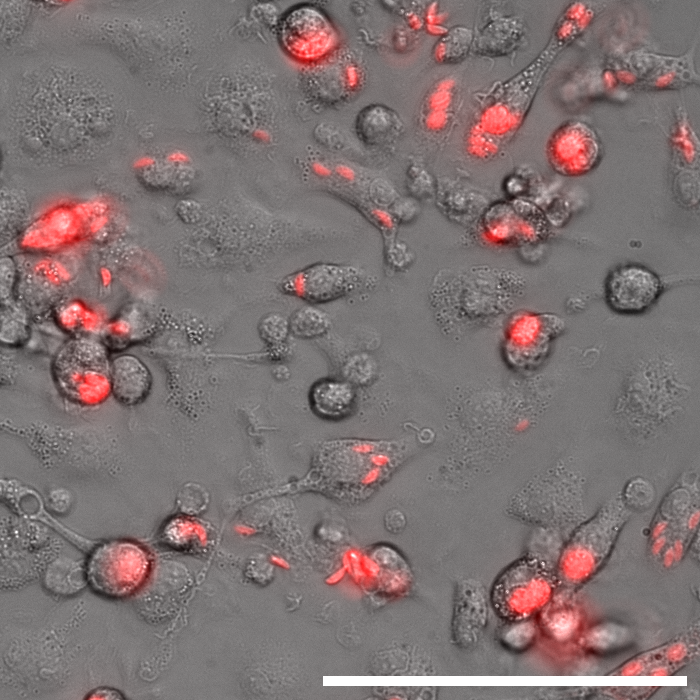

Supplement: Supplementary file 15 — Source data [file 41467_2025_58876_MOESM15_ESM.zip › Source suppl/Supplementary Figure 5_Source Data/Suppl Fig5d/BMDM_noIFNG_UPRT_MERGE_scale.png]

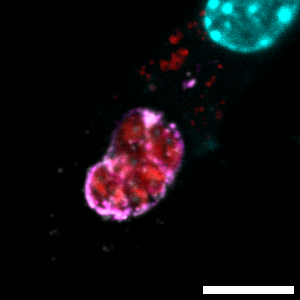

Supplement: Supplementary file 15 — Source data [file 41467_2025_58876_MOESM15_ESM.zip › Source suppl/Supplementary Figure 6_Source Data/Suppl Fig6c/BMDM_plus_UPRT_1_MERGE_crop.tif]

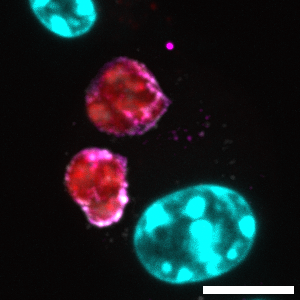

Supplement: Supplementary file 15 — Source data [file 41467_2025_58876_MOESM15_ESM.zip › Source suppl/Supplementary Figure 6_Source Data/Suppl Fig6c/BMDM_minus_dGRA12_1_MERGE_crop.tif]

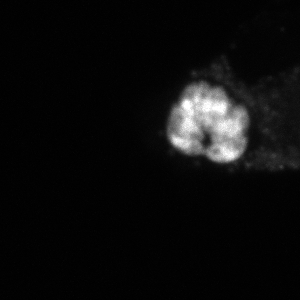

Supplement: Supplementary file 15 — Source data [file 41467_2025_58876_MOESM15_ESM.zip › Source suppl/Supplementary Figure 6_Source Data/Suppl Fig6c/BMDM_plus_dGRA12_5_toxo_crop.tif]

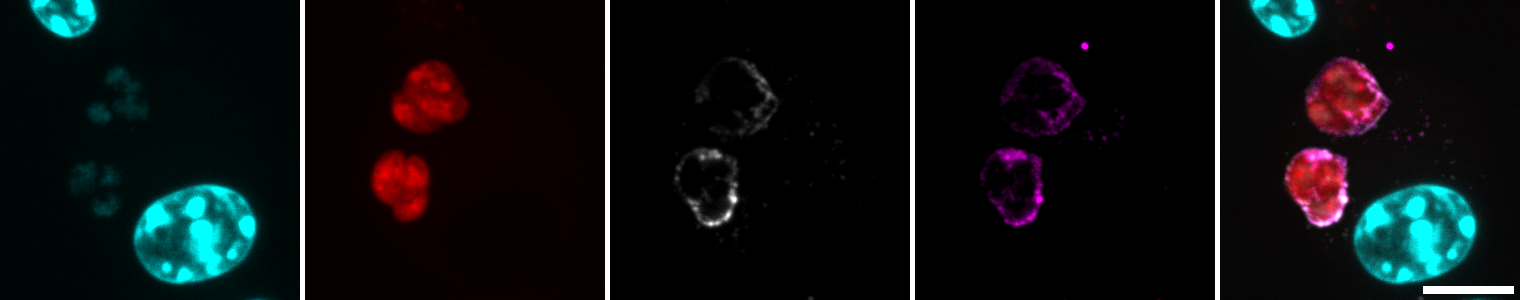

Supplement: Supplementary file 15 — Source data [file 41467_2025_58876_MOESM15_ESM.zip › Source suppl/Supplementary Figure 6_Source Data/Suppl Fig6c/BMDM_minus_dGRA12_1_MONTAGE_crop.tif]

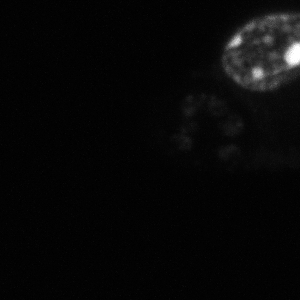

Supplement: Supplementary file 15 — Source data [file 41467_2025_58876_MOESM15_ESM.zip › Source suppl/Supplementary Figure 6_Source Data/Suppl Fig6c/BMDM_plus_dGRA12_5_DAPI_crop.tif]

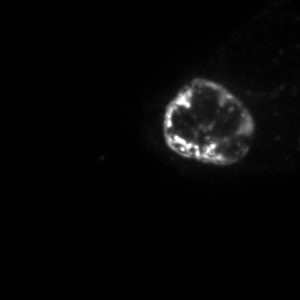

Supplement: Supplementary file 15 — Source data [file 41467_2025_58876_MOESM15_ESM.zip › Source suppl/Supplementary Figure 6_Source Data/Suppl Fig6c/BMDM_plus_dGRA12_5_GRA3_crop.tif]

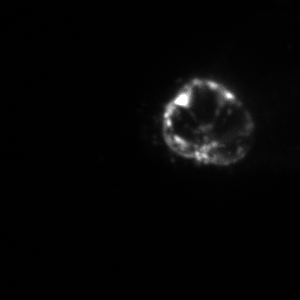

Supplement: Supplementary file 15 — Source data [file 41467_2025_58876_MOESM15_ESM.zip › Source suppl/Supplementary Figure 6_Source Data/Suppl Fig6c/BMDM_plus_dGRA12_5_GRA2_crop.tif]

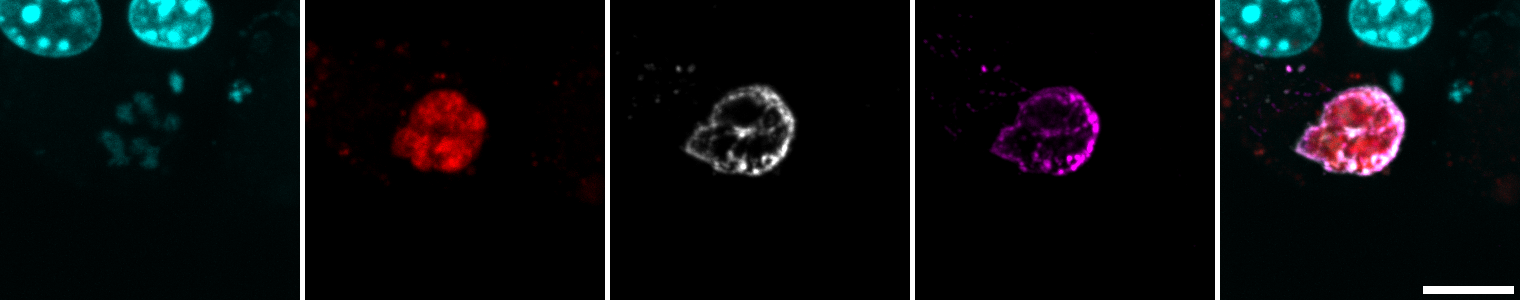

Supplement: Supplementary file 15 — Source data [file 41467_2025_58876_MOESM15_ESM.zip › Source suppl/Supplementary Figure 6_Source Data/Suppl Fig6c/BMDM_minus_UPRT_1_MONTAGE_crop.tif]

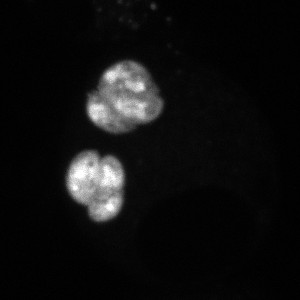

Supplement: Supplementary file 15 — Source data [file 41467_2025_58876_MOESM15_ESM.zip › Source suppl/Supplementary Figure 6_Source Data/Suppl Fig6c/BMDM_minus_dGRA12_1_toxo_crop.tif]

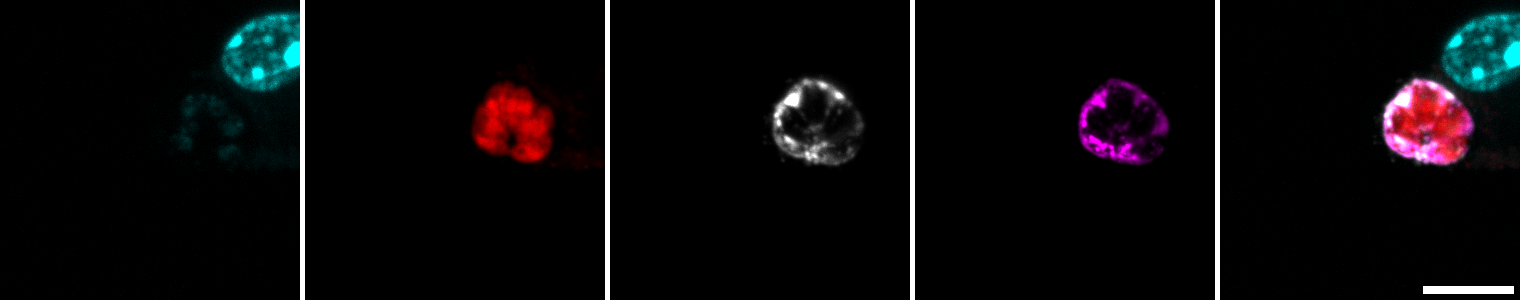

Supplement: Supplementary file 15 — Source data [file 41467_2025_58876_MOESM15_ESM.zip › Source suppl/Supplementary Figure 6_Source Data/Suppl Fig6c/BMDM_plus_dGRA12_5_MONTAGE_crop.tif]

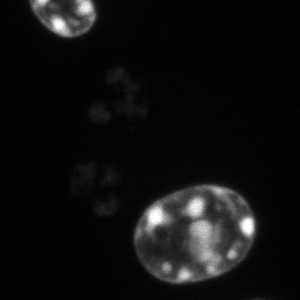

Supplement: Supplementary file 15 — Source data [file 41467_2025_58876_MOESM15_ESM.zip › Source suppl/Supplementary Figure 6_Source Data/Suppl Fig6c/BMDM_minus_dGRA12_1_DAPI_crop.tif]

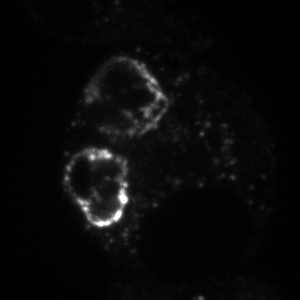

Supplement: Supplementary file 15 — Source data [file 41467_2025_58876_MOESM15_ESM.zip › Source suppl/Supplementary Figure 6_Source Data/Suppl Fig6c/BMDM_minus_dGRA12_1_GRA2_crop.tif]

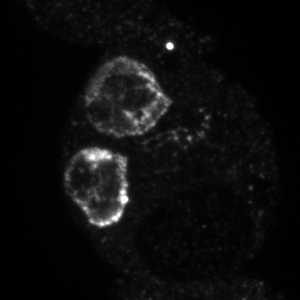

Supplement: Supplementary file 15 — Source data [file 41467_2025_58876_MOESM15_ESM.zip › Source suppl/Supplementary Figure 6_Source Data/Suppl Fig6c/BMDM_minus_dGRA12_1_GRA3_crop.tif]

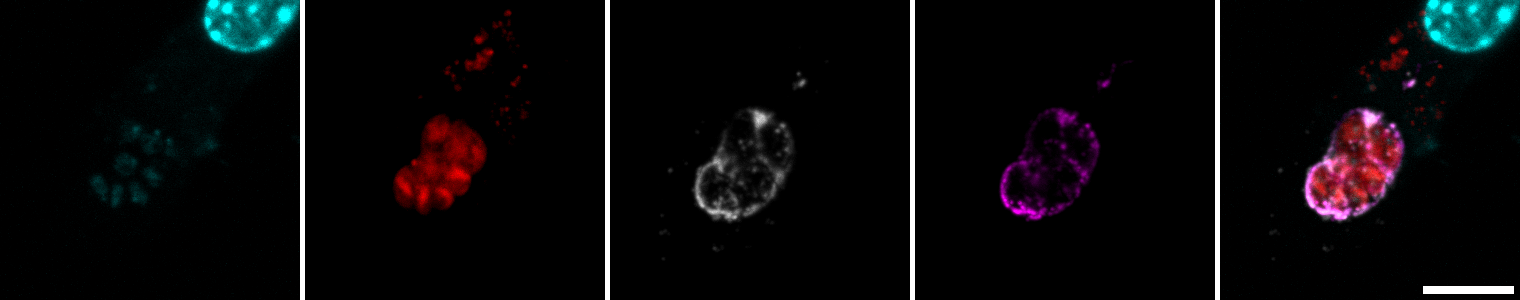

Supplement: Supplementary file 15 — Source data [file 41467_2025_58876_MOESM15_ESM.zip › Source suppl/Supplementary Figure 6_Source Data/Suppl Fig6c/BMDM_plus_UPRT_1_MONTAGE_crop.tif]

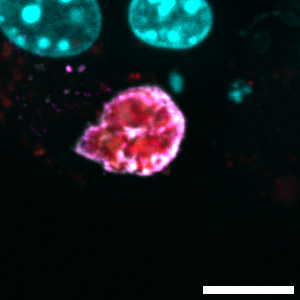

Supplement: Supplementary file 15 — Source data [file 41467_2025_58876_MOESM15_ESM.zip › Source suppl/Supplementary Figure 6_Source Data/Suppl Fig6c/BMDM_minus_UPRT_1_MERGE_crop.tif]

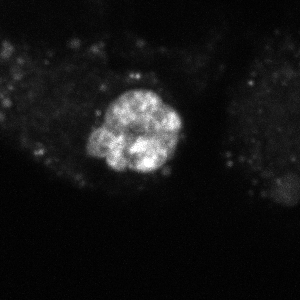

Supplement: Supplementary file 15 — Source data [file 41467_2025_58876_MOESM15_ESM.zip › Source suppl/Supplementary Figure 6_Source Data/Suppl Fig6c/BMDM_minus_UPRT_1_toxo_crop.tif]

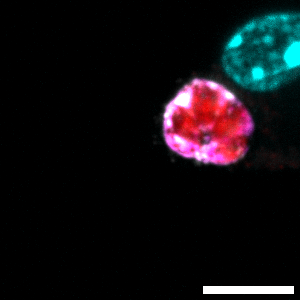

Supplement: Supplementary file 15 — Source data [file 41467_2025_58876_MOESM15_ESM.zip › Source suppl/Supplementary Figure 6_Source Data/Suppl Fig6c/BMDM_plus_dGRA12_5_MERGE_crop.tif]

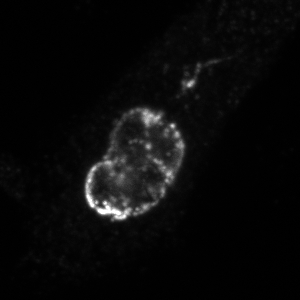

Supplement: Supplementary file 15 — Source data [file 41467_2025_58876_MOESM15_ESM.zip › Source suppl/Supplementary Figure 6_Source Data/Suppl Fig6c/BMDM_plus_UPRT_1_GRA3_crop.tif]

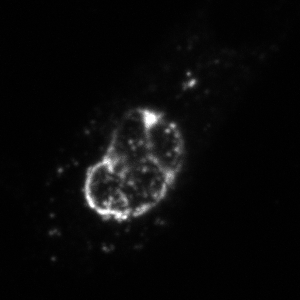

Supplement: Supplementary file 15 — Source data [file 41467_2025_58876_MOESM15_ESM.zip › Source suppl/Supplementary Figure 6_Source Data/Suppl Fig6c/BMDM_plus_UPRT_1_GRA2_crop.tif]

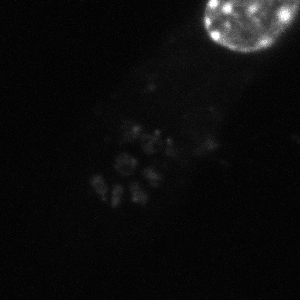

Supplement: Supplementary file 15 — Source data [file 41467_2025_58876_MOESM15_ESM.zip › Source suppl/Supplementary Figure 6_Source Data/Suppl Fig6c/BMDM_plus_UPRT_1_DAPI_crop.tif]
