# Supplementary figures and images for: GRA12 is a common virulence factor across Toxoplasma gondii strains and mouse subspecies (part 3 of 3)
Source: Nat Commun. 2025 Apr 16;16:3570. doi: 10.1038/s41467-025-58876-2 (PMC12003902; doi:10.1038/s41467-025-58876-2)

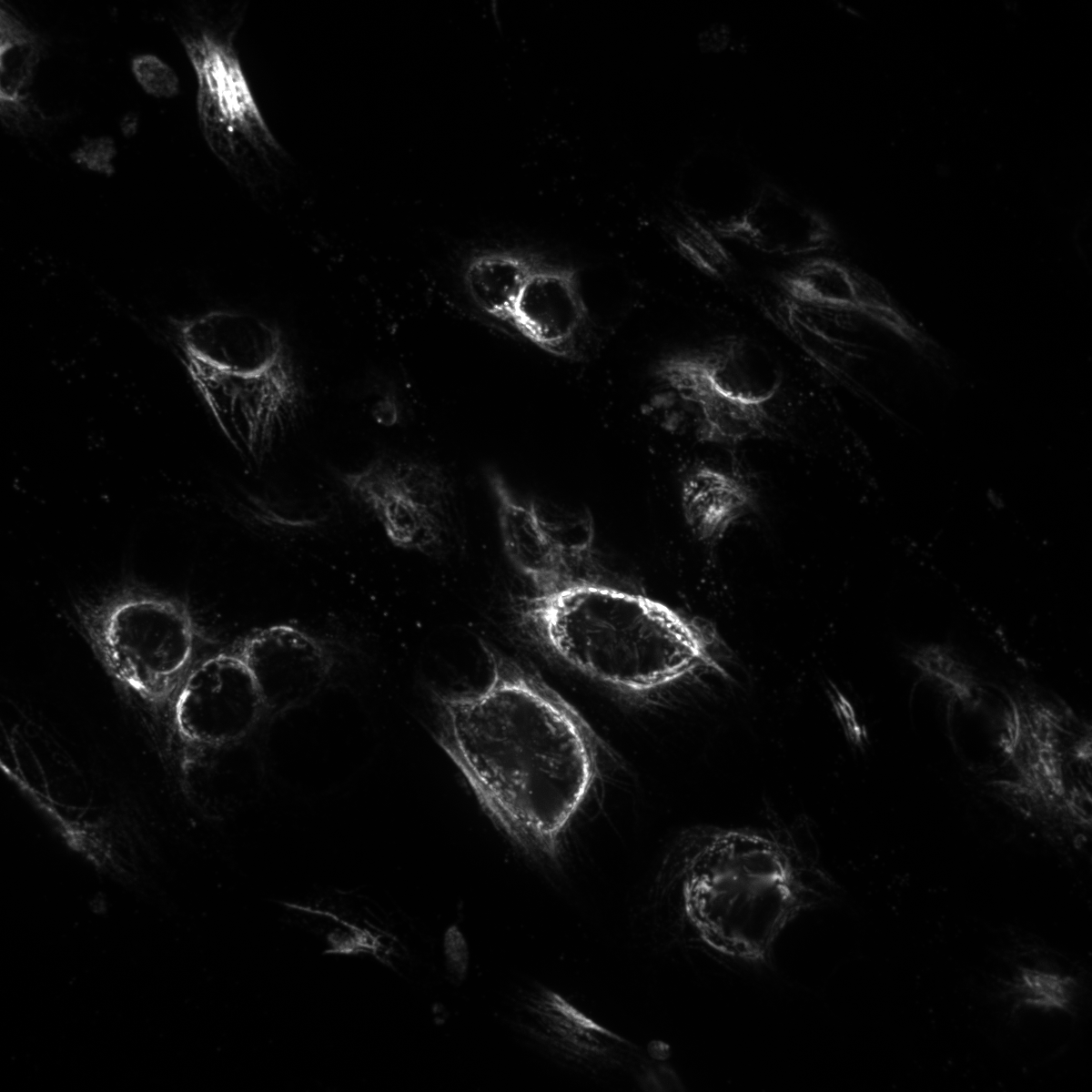

Supplement: Supplementary file 16 — Supplementary Figs. Source data [file 41467_2025_58876_MOESM16_ESM.zip › Source_mian_figures/Figure 5_Source Data/Fig 5a/FT 240226 coinf 15s 100x project/FT_240226_topology-0005_atubulin_adj.tif]

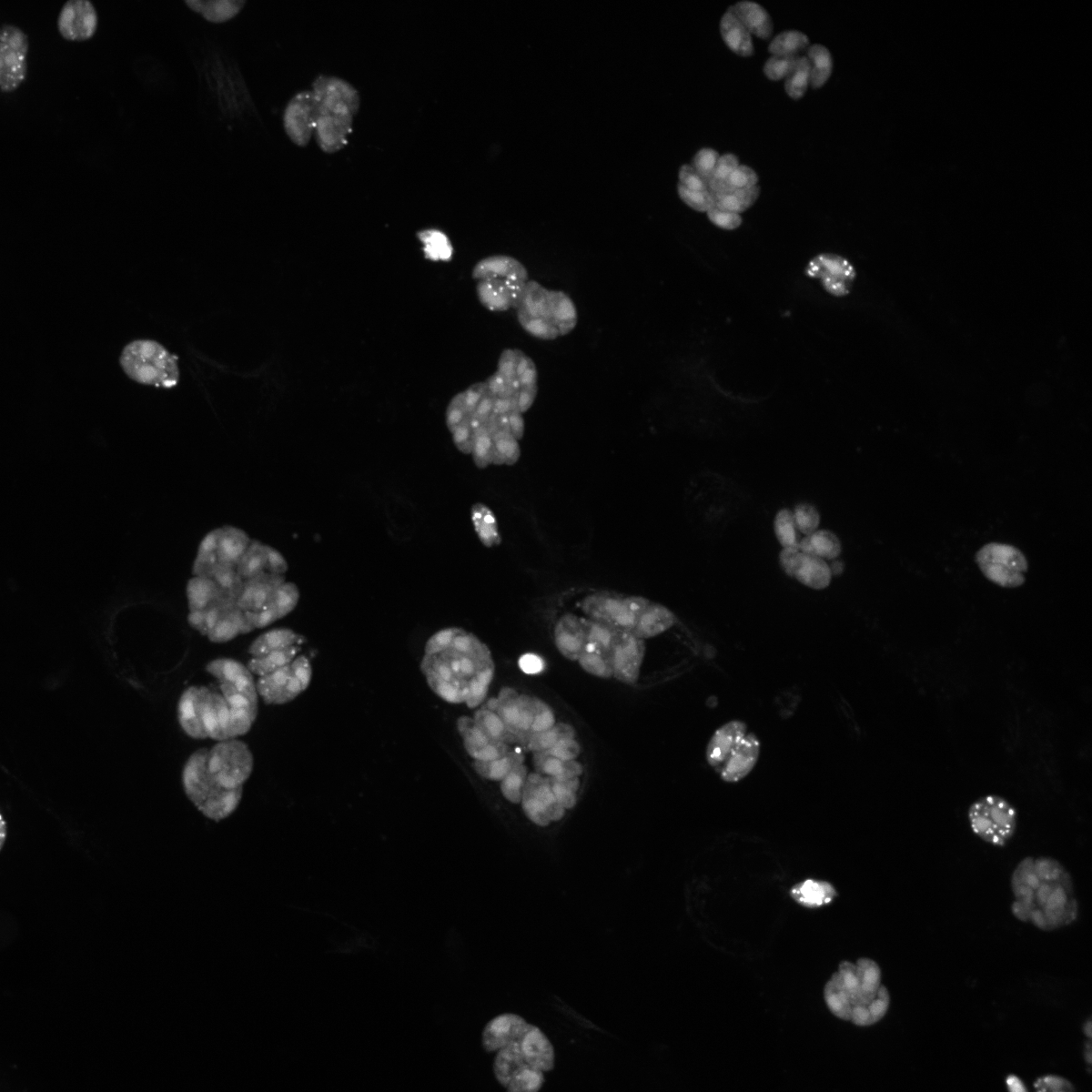

Supplement: Supplementary file 16 — Supplementary Figs. Source data [file 41467_2025_58876_MOESM16_ESM.zip › Source_mian_figures/Figure 5_Source Data/Fig 5a/FT 240226 coinf 15s 100x project/FT_240226_topology-0004_mCH_adj.tif]

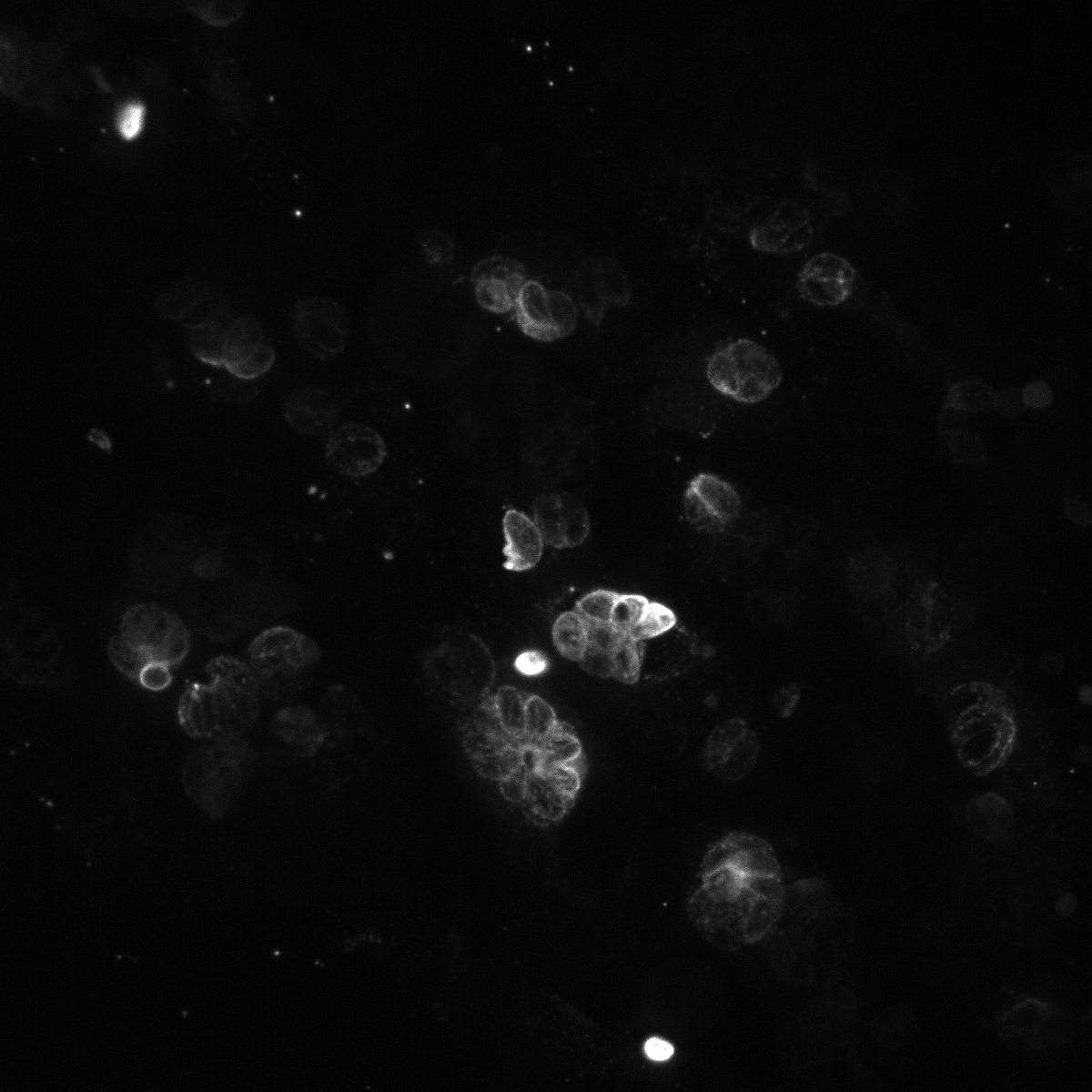

Supplement: Supplementary file 16 — Supplementary Figs. Source data [file 41467_2025_58876_MOESM16_ESM.zip › Source_mian_figures/Figure 5_Source Data/Fig 5a/FT 240226 coinf 15s 100x project/FT_240226_topology-0002_toxo.tif]

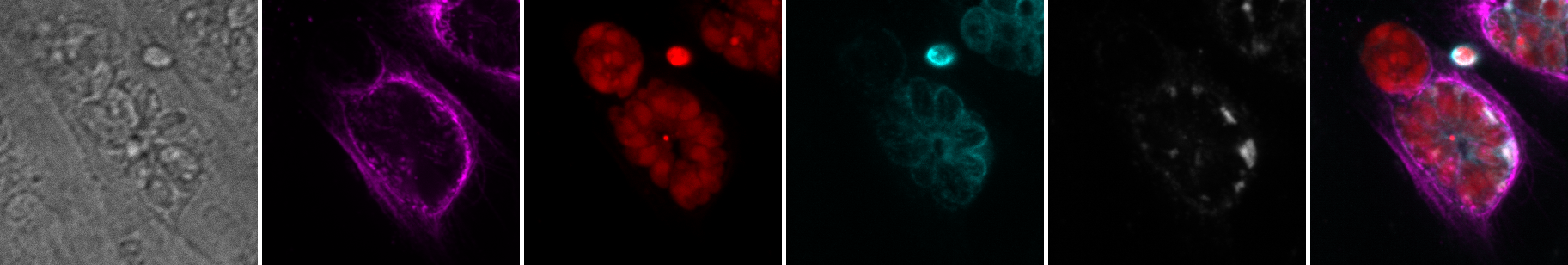

Supplement: Supplementary file 16 — Supplementary Figs. Source data [file 41467_2025_58876_MOESM16_ESM.zip › Source_mian_figures/Figure 5_Source Data/Fig 5a/FT 240226 coinf 15s 100x project/Montage.tif]

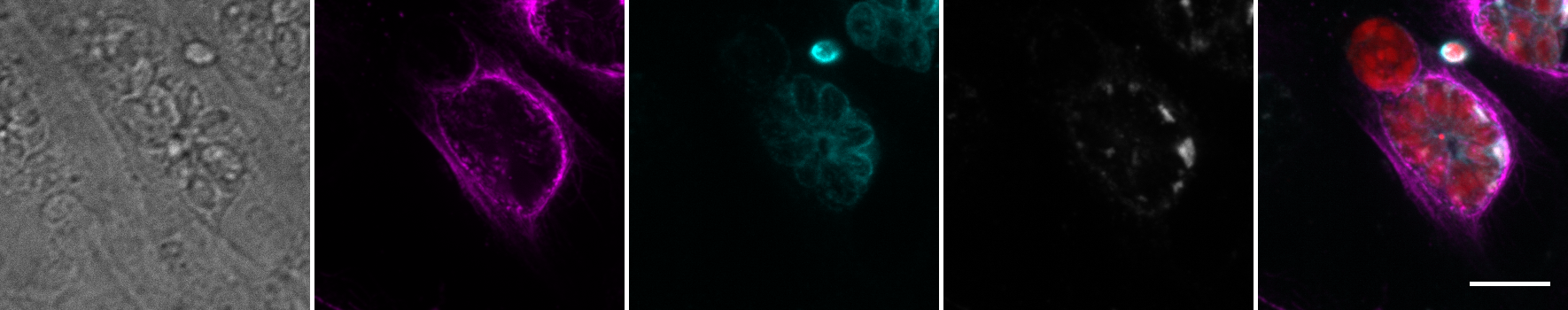

Supplement: Supplementary file 16 — Supplementary Figs. Source data [file 41467_2025_58876_MOESM16_ESM.zip › Source_mian_figures/Figure 5_Source Data/Fig 5a/FT 240226 coinf 15s 100x project/Montage_scale.tif]

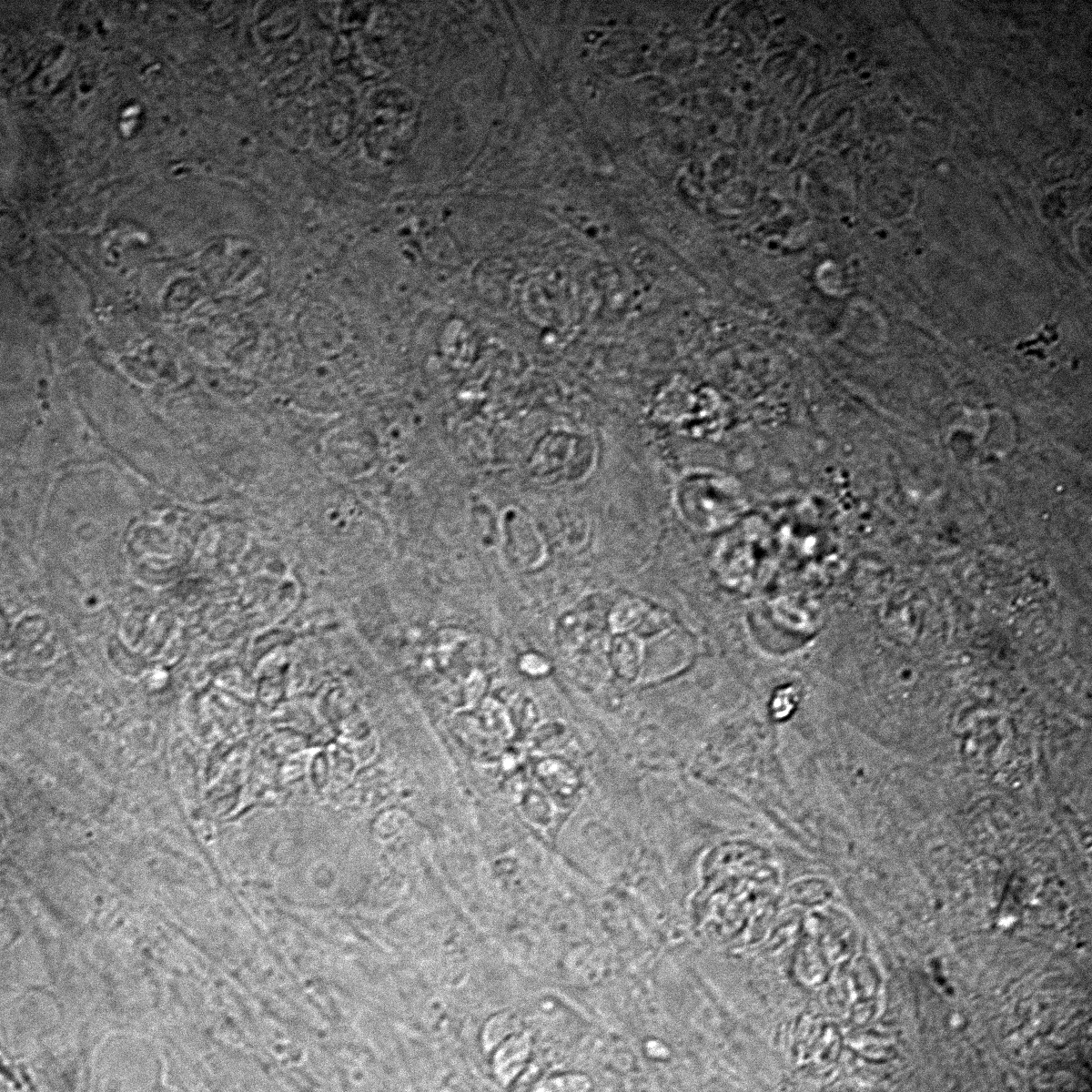

Supplement: Supplementary file 16 — Supplementary Figs. Source data [file 41467_2025_58876_MOESM16_ESM.zip › Source_mian_figures/Figure 5_Source Data/Fig 5a/FT 240226 coinf 15s 100x project/FT_240226_topology-0001_BF.tif]

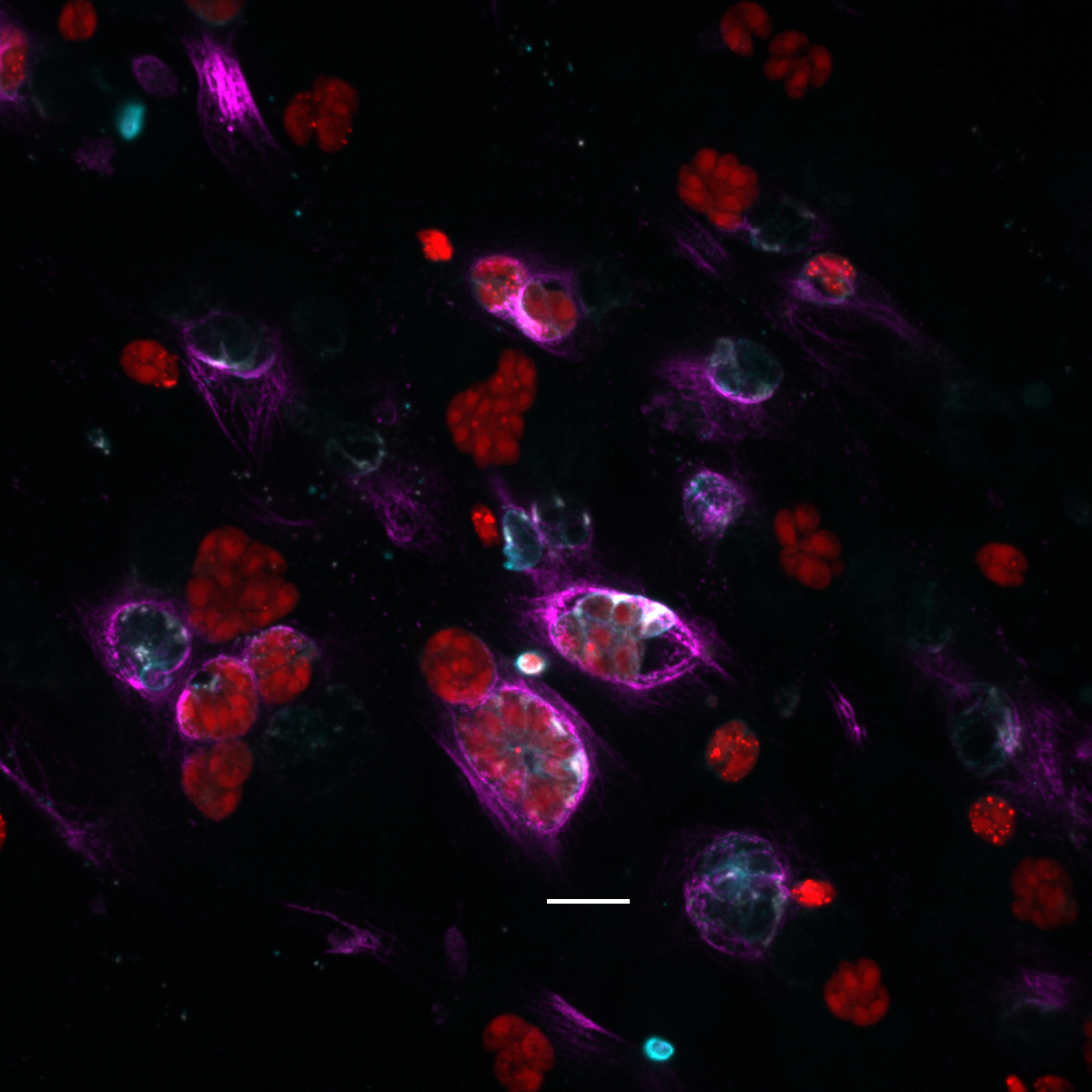

Supplement: Supplementary file 16 — Supplementary Figs. Source data [file 41467_2025_58876_MOESM16_ESM.zip › Source_mian_figures/Figure 5_Source Data/Fig 5a/FT 240226 coinf 15s 100x project/MERGE_scale.tif]

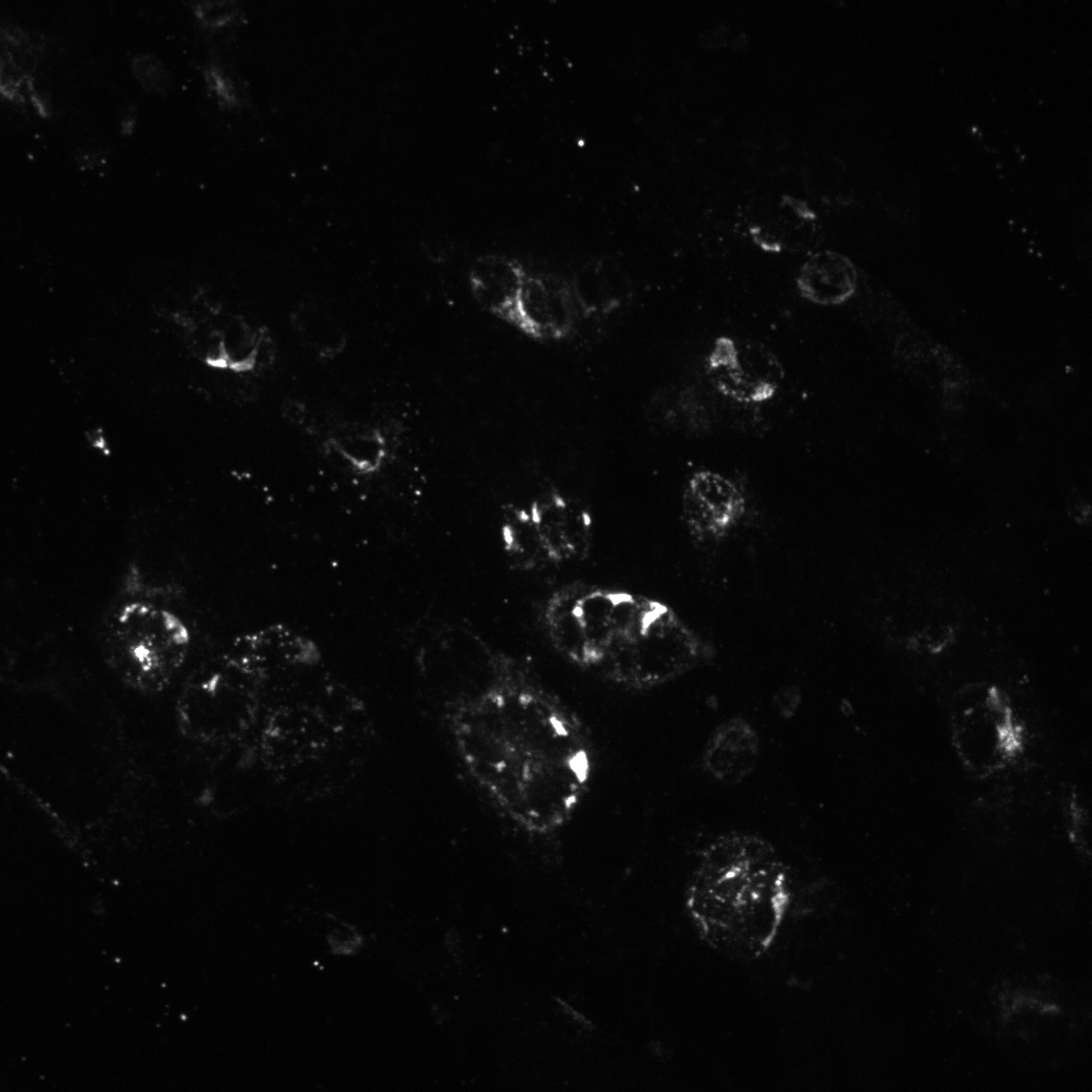

Supplement: Supplementary file 16 — Supplementary Figs. Source data [file 41467_2025_58876_MOESM16_ESM.zip › Source_mian_figures/Figure 5_Source Data/Fig 5a/FT 240226 coinf 15s 100x project/FT_240226_topology-0003_HA.tif]

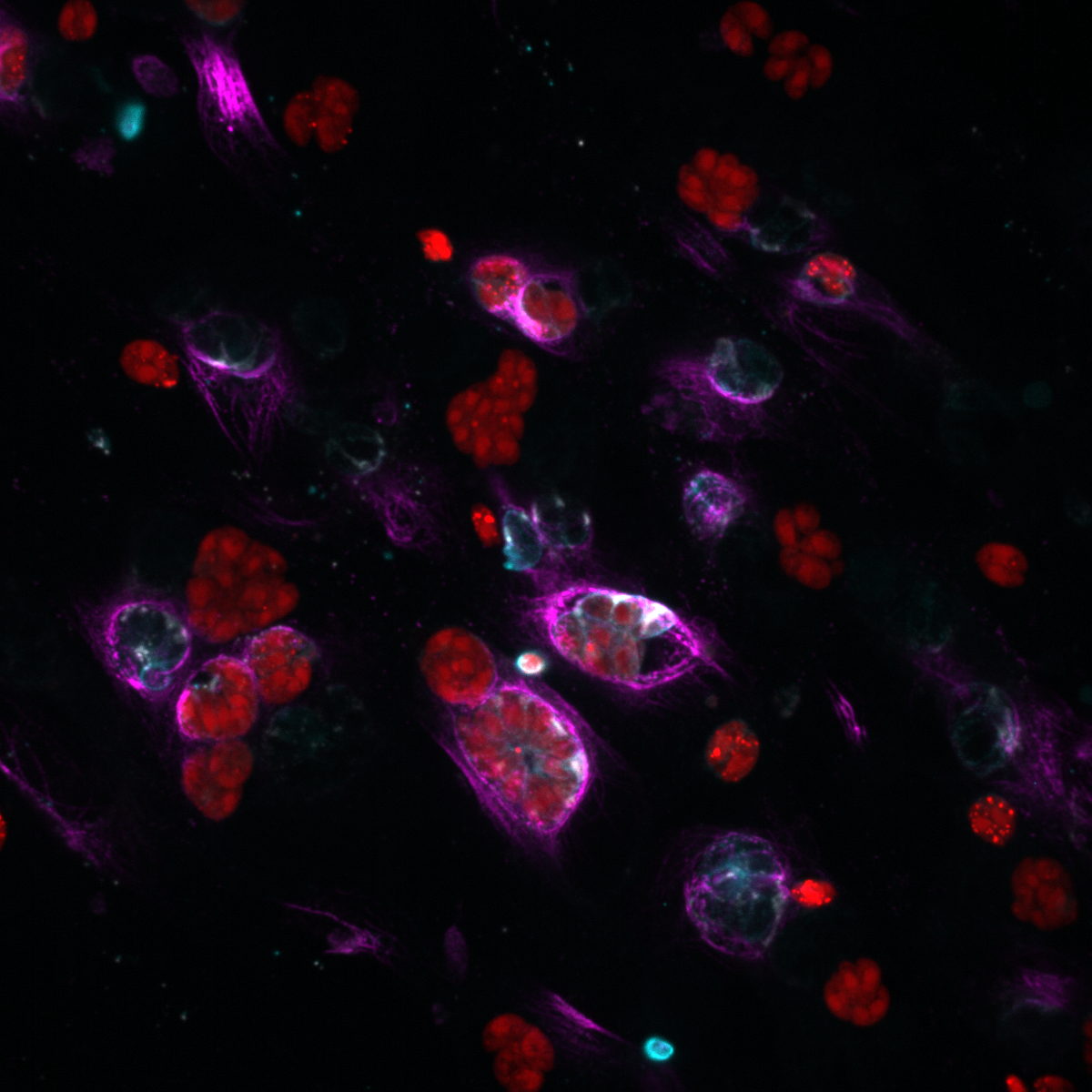

Supplement: Supplementary file 16 — Supplementary Figs. Source data [file 41467_2025_58876_MOESM16_ESM.zip › Source_mian_figures/Figure 5_Source Data/Fig 5a/FT 240226 coinf 15s 100x project/MERGE.tif]

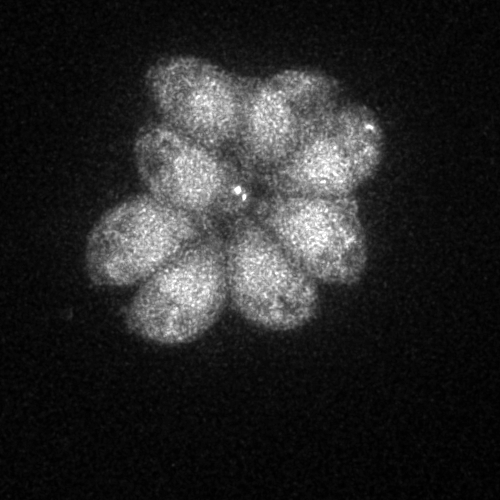

Supplement: Supplementary file 16 — Supplementary Figs. Source data [file 41467_2025_58876_MOESM16_ESM.zip › Source_mian_figures/Figure 2_Source Data/Fig 2a/FT_230911_VAND_COMPL_HA488_GRA2_647_150x_B_1-decon/FT_230911_VAND_COMPL_HA488_GRA2_647_150x_B_1-decon/FT_230911_VAND_COMPL_HA488_GRA2_647_150x_B_1_MMStack_Pos0.ome-0004_toxo.tif]

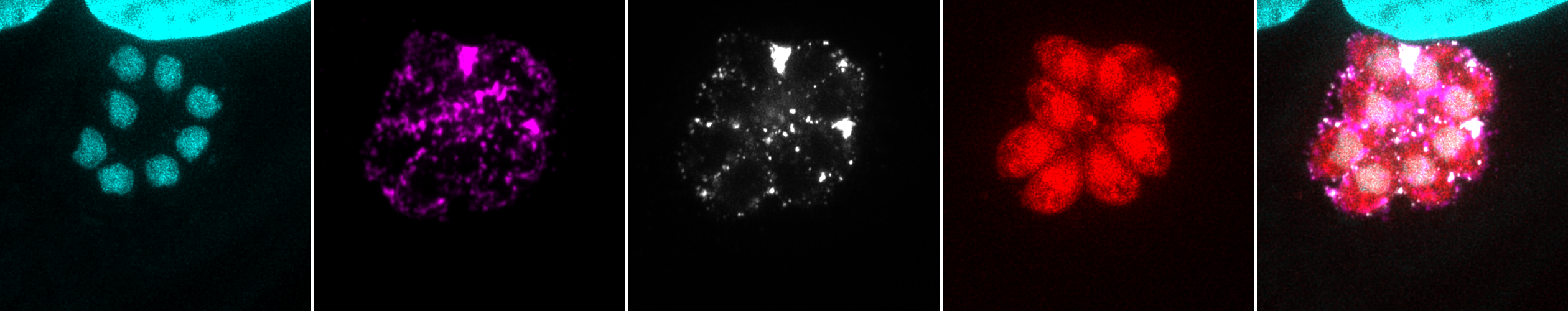

Supplement: Supplementary file 16 — Supplementary Figs. Source data [file 41467_2025_58876_MOESM16_ESM.zip › Source_mian_figures/Figure 2_Source Data/Fig 2a/FT_230911_VAND_COMPL_HA488_GRA2_647_150x_B_1-decon/FT_230911_VAND_COMPL_HA488_GRA2_647_150x_B_1-decon/Montage_NEW.tif]

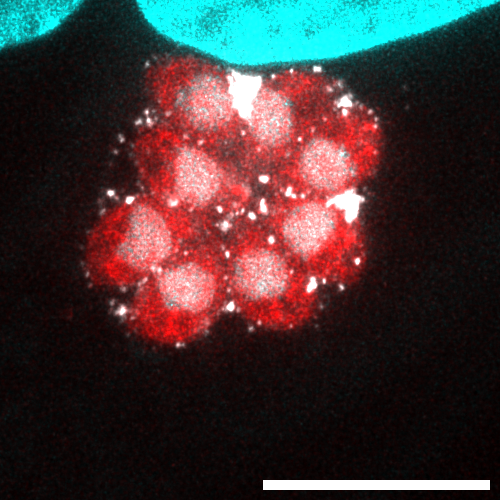

Supplement: Supplementary file 16 — Supplementary Figs. Source data [file 41467_2025_58876_MOESM16_ESM.zip › Source_mian_figures/Figure 2_Source Data/Fig 2a/FT_230911_VAND_COMPL_HA488_GRA2_647_150x_B_1-decon/FT_230911_VAND_COMPL_HA488_GRA2_647_150x_B_1-decon/MERGE_noGRA2_scale.tif]

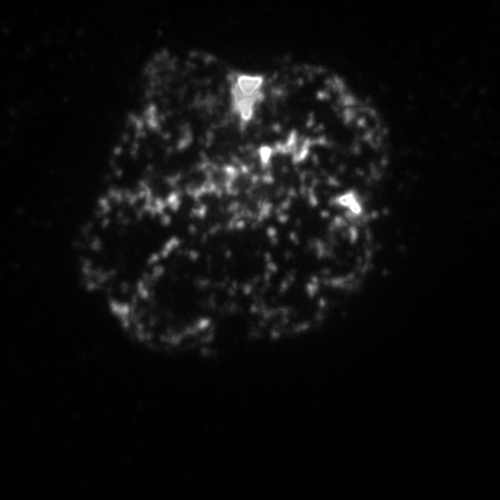

Supplement: Supplementary file 16 — Supplementary Figs. Source data [file 41467_2025_58876_MOESM16_ESM.zip › Source_mian_figures/Figure 2_Source Data/Fig 2a/FT_230911_VAND_COMPL_HA488_GRA2_647_150x_B_1-decon/FT_230911_VAND_COMPL_HA488_GRA2_647_150x_B_1-decon/FT_230911_VAND_COMPL_HA488_GRA2_647_150x_B_1_MMStack_Pos0.ome-0003_GRA2.tif]

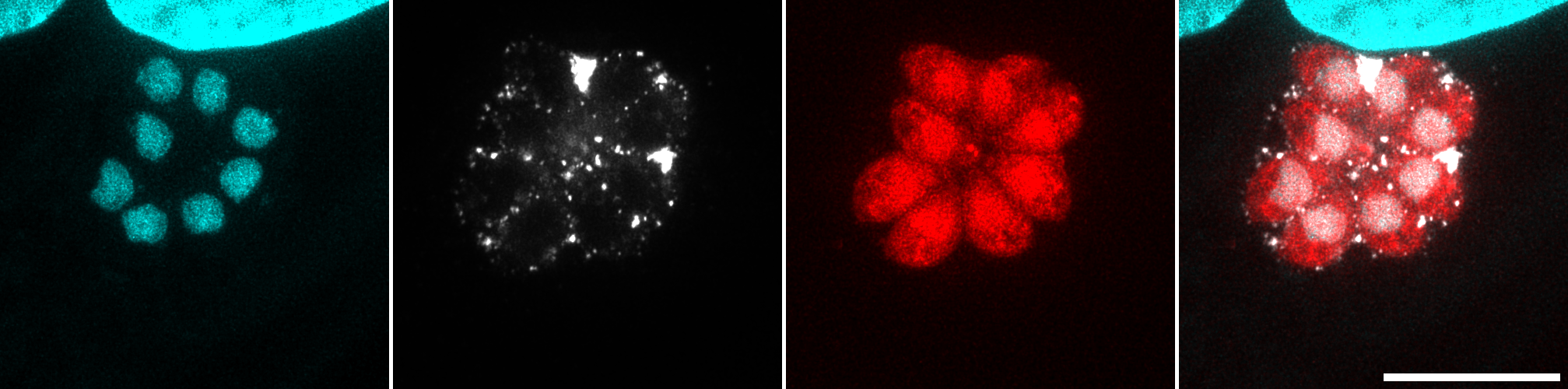

Supplement: Supplementary file 16 — Supplementary Figs. Source data [file 41467_2025_58876_MOESM16_ESM.zip › Source_mian_figures/Figure 2_Source Data/Fig 2a/FT_230911_VAND_COMPL_HA488_GRA2_647_150x_B_1-decon/FT_230911_VAND_COMPL_HA488_GRA2_647_150x_B_1-decon/Montage_NEW_scale.tif]

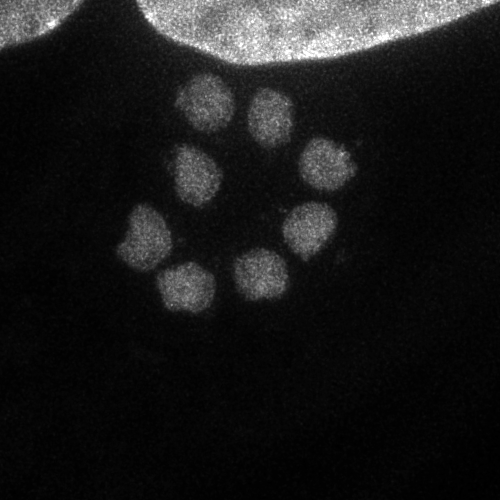

Supplement: Supplementary file 16 — Supplementary Figs. Source data [file 41467_2025_58876_MOESM16_ESM.zip › Source_mian_figures/Figure 2_Source Data/Fig 2a/FT_230911_VAND_COMPL_HA488_GRA2_647_150x_B_1-decon/FT_230911_VAND_COMPL_HA488_GRA2_647_150x_B_1-decon/FT_230911_VAND_COMPL_HA488_GRA2_647_150x_B_1_MMStack_Pos0.ome-0002_DAPI.tif]

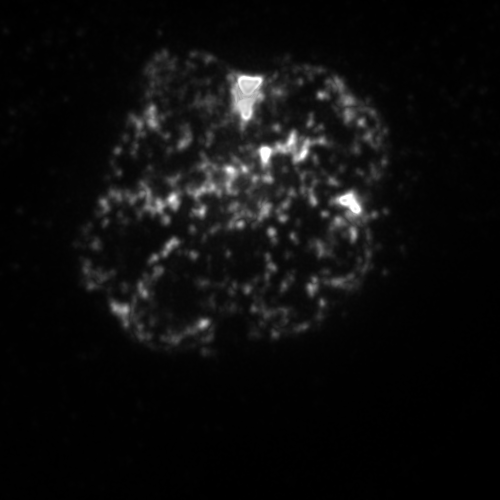

Supplement: Supplementary file 16 — Supplementary Figs. Source data [file 41467_2025_58876_MOESM16_ESM.zip › Source_mian_figures/Figure 2_Source Data/Fig 2a/FT_230911_VAND_COMPL_HA488_GRA2_647_150x_B_1-decon/FT_230911_VAND_COMPL_HA488_GRA2_647_150x_B_1-decon/FT_230911_VAND_COMPL_HA488_GRA2_647_150x_B_1_MMStack_Pos0.ome-0003_GRA2_NEW.tif]

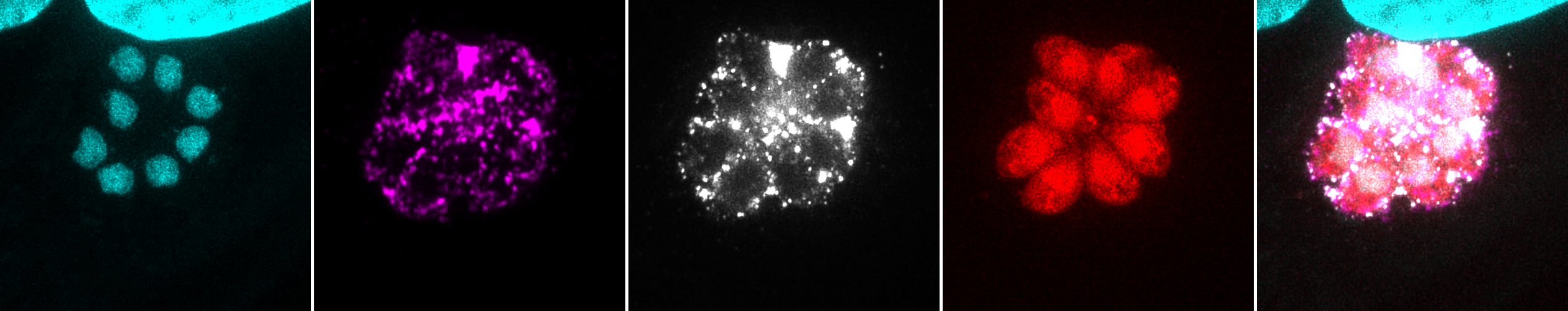

Supplement: Supplementary file 16 — Supplementary Figs. Source data [file 41467_2025_58876_MOESM16_ESM.zip › Source_mian_figures/Figure 2_Source Data/Fig 2a/FT_230911_VAND_COMPL_HA488_GRA2_647_150x_B_1-decon/FT_230911_VAND_COMPL_HA488_GRA2_647_150x_B_1-decon/Montage.tif]

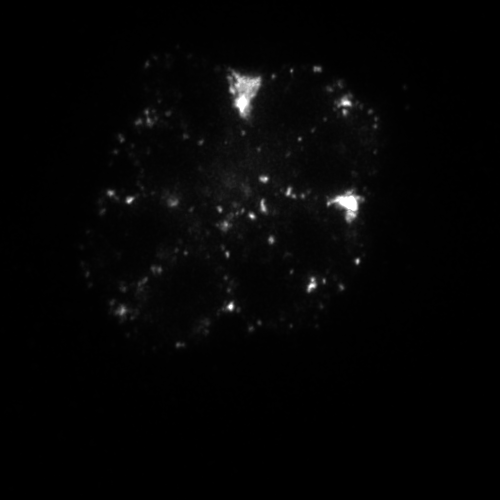

Supplement: Supplementary file 16 — Supplementary Figs. Source data [file 41467_2025_58876_MOESM16_ESM.zip › Source_mian_figures/Figure 2_Source Data/Fig 2a/FT_230911_VAND_COMPL_HA488_GRA2_647_150x_B_1-decon/FT_230911_VAND_COMPL_HA488_GRA2_647_150x_B_1-decon/FT_230911_VAND_COMPL_HA488_GRA2_647_150x_B_1_MMStack_Pos0.ome-0001_HA_NEW.tif]

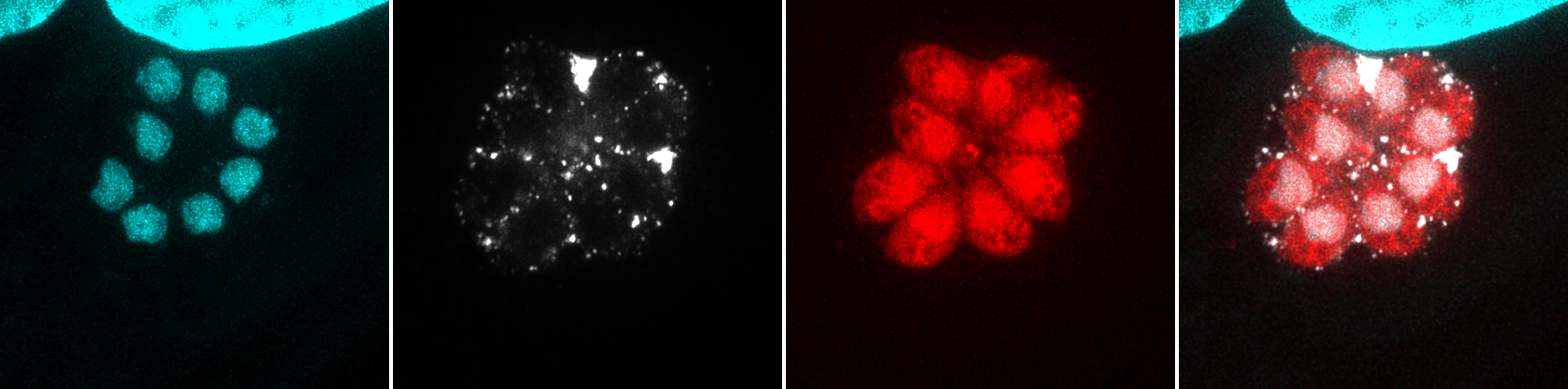

Supplement: Supplementary file 16 — Supplementary Figs. Source data [file 41467_2025_58876_MOESM16_ESM.zip › Source_mian_figures/Figure 2_Source Data/Fig 2a/FT_230911_VAND_COMPL_HA488_GRA2_647_150x_B_1-decon/FT_230911_VAND_COMPL_HA488_GRA2_647_150x_B_1-decon/Montage_noGRA2.tif]

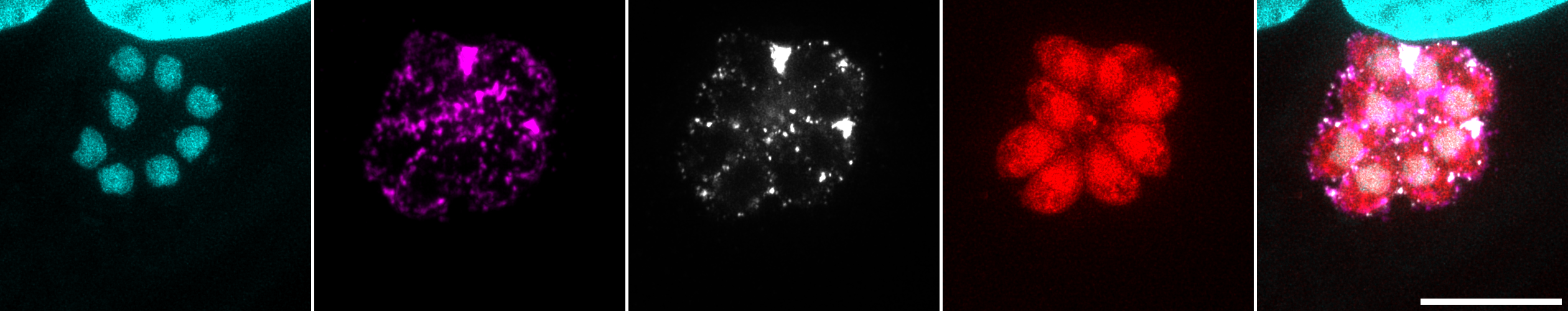

Supplement: Supplementary file 16 — Supplementary Figs. Source data [file 41467_2025_58876_MOESM16_ESM.zip › Source_mian_figures/Figure 2_Source Data/Fig 2a/FT_230911_VAND_COMPL_HA488_GRA2_647_150x_B_1-decon/FT_230911_VAND_COMPL_HA488_GRA2_647_150x_B_1-decon/MERGE_NEW_MONTAGE.tif]

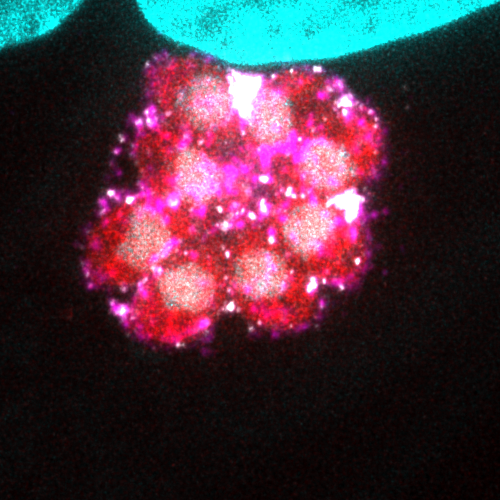

Supplement: Supplementary file 16 — Supplementary Figs. Source data [file 41467_2025_58876_MOESM16_ESM.zip › Source_mian_figures/Figure 2_Source Data/Fig 2a/FT_230911_VAND_COMPL_HA488_GRA2_647_150x_B_1-decon/FT_230911_VAND_COMPL_HA488_GRA2_647_150x_B_1-decon/MERGE_NEW.tif]

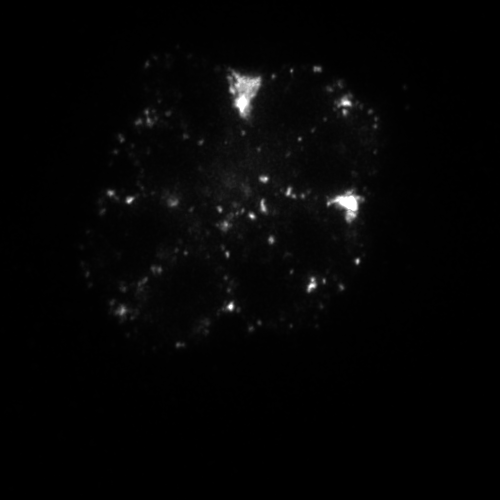

Supplement: Supplementary file 16 — Supplementary Figs. Source data [file 41467_2025_58876_MOESM16_ESM.zip › Source_mian_figures/Figure 2_Source Data/Fig 2a/FT_230911_VAND_COMPL_HA488_GRA2_647_150x_B_1-decon/FT_230911_VAND_COMPL_HA488_GRA2_647_150x_B_1-decon/FT_230911_VAND_COMPL_HA488_GRA2_647_150x_B_1_MMStack_Pos0.ome-0001_HA.tif]

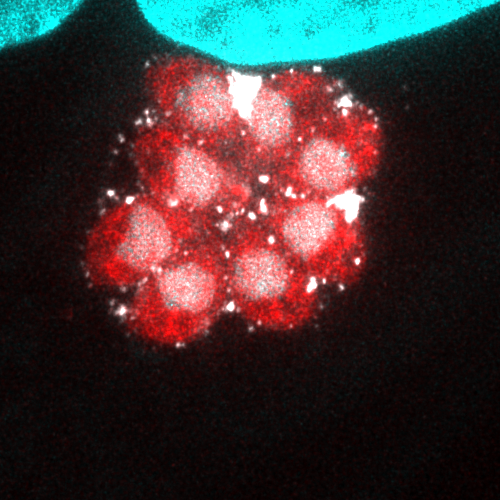

Supplement: Supplementary file 16 — Supplementary Figs. Source data [file 41467_2025_58876_MOESM16_ESM.zip › Source_mian_figures/Figure 2_Source Data/Fig 2a/FT_230911_VAND_COMPL_HA488_GRA2_647_150x_B_1-decon/FT_230911_VAND_COMPL_HA488_GRA2_647_150x_B_1-decon/MERGE_noGRA2.tif]

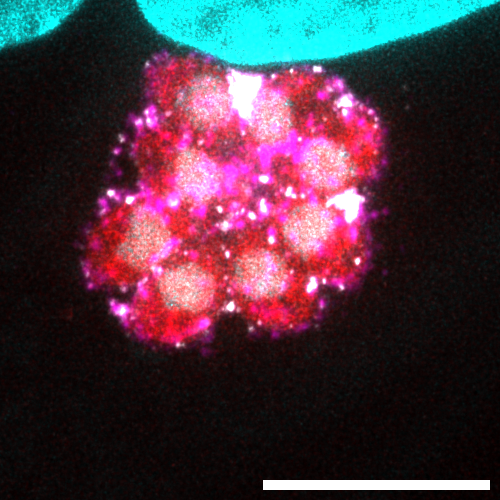

Supplement: Supplementary file 16 — Supplementary Figs. Source data [file 41467_2025_58876_MOESM16_ESM.zip › Source_mian_figures/Figure 2_Source Data/Fig 2a/FT_230911_VAND_COMPL_HA488_GRA2_647_150x_B_1-decon/FT_230911_VAND_COMPL_HA488_GRA2_647_150x_B_1-decon/MERGE_NEW_scale.tif]

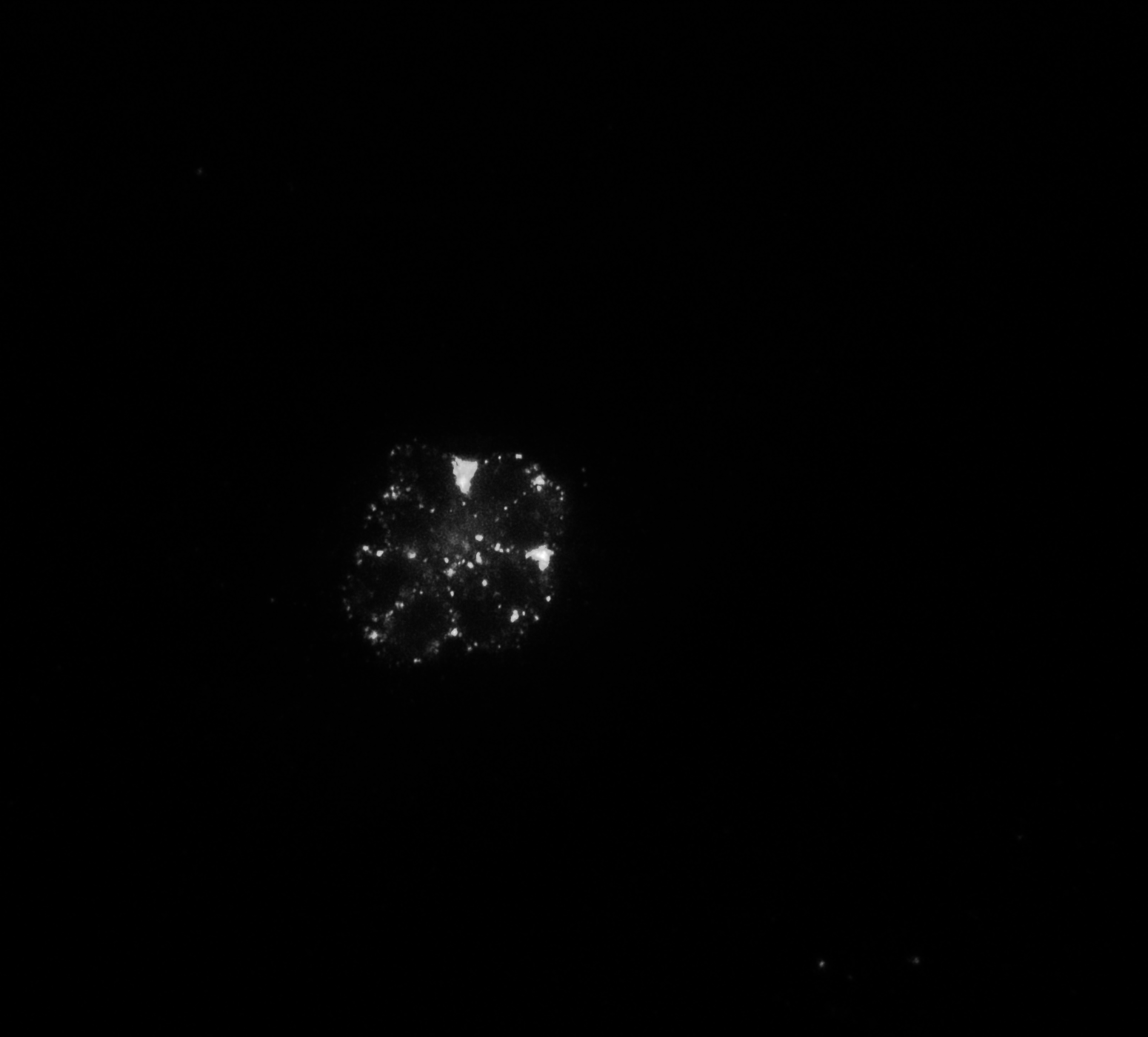

Supplement: Supplementary file 16 — Supplementary Figs. Source data [file 41467_2025_58876_MOESM16_ESM.zip › Source_mian_figures/Figure 2_Source Data/Fig 2a/FT_230911_VAND_COMPL_HA488_GRA2_647_150x_B_1-decon/FT_230911_VAND_COMPL_HA488_GRA2_647_150x_B_1-decon/FT_230911_VAND_COMPL_HA488_GRA2_647_150x_B_1_MMStack_Pos0.ome.tif]

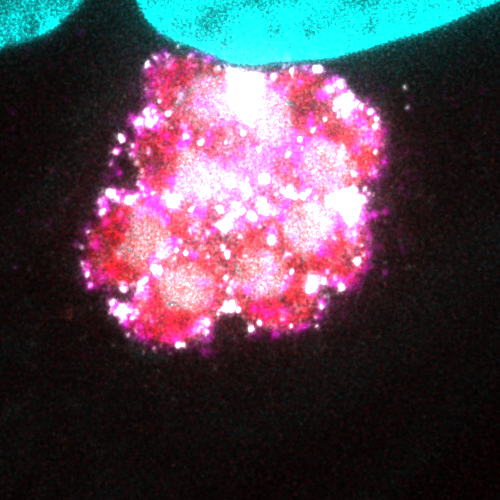

Supplement: Supplementary file 16 — Supplementary Figs. Source data [file 41467_2025_58876_MOESM16_ESM.zip › Source_mian_figures/Figure 2_Source Data/Fig 2a/FT_230911_VAND_COMPL_HA488_GRA2_647_150x_B_1-decon/FT_230911_VAND_COMPL_HA488_GRA2_647_150x_B_1-decon/MERGE.tif]

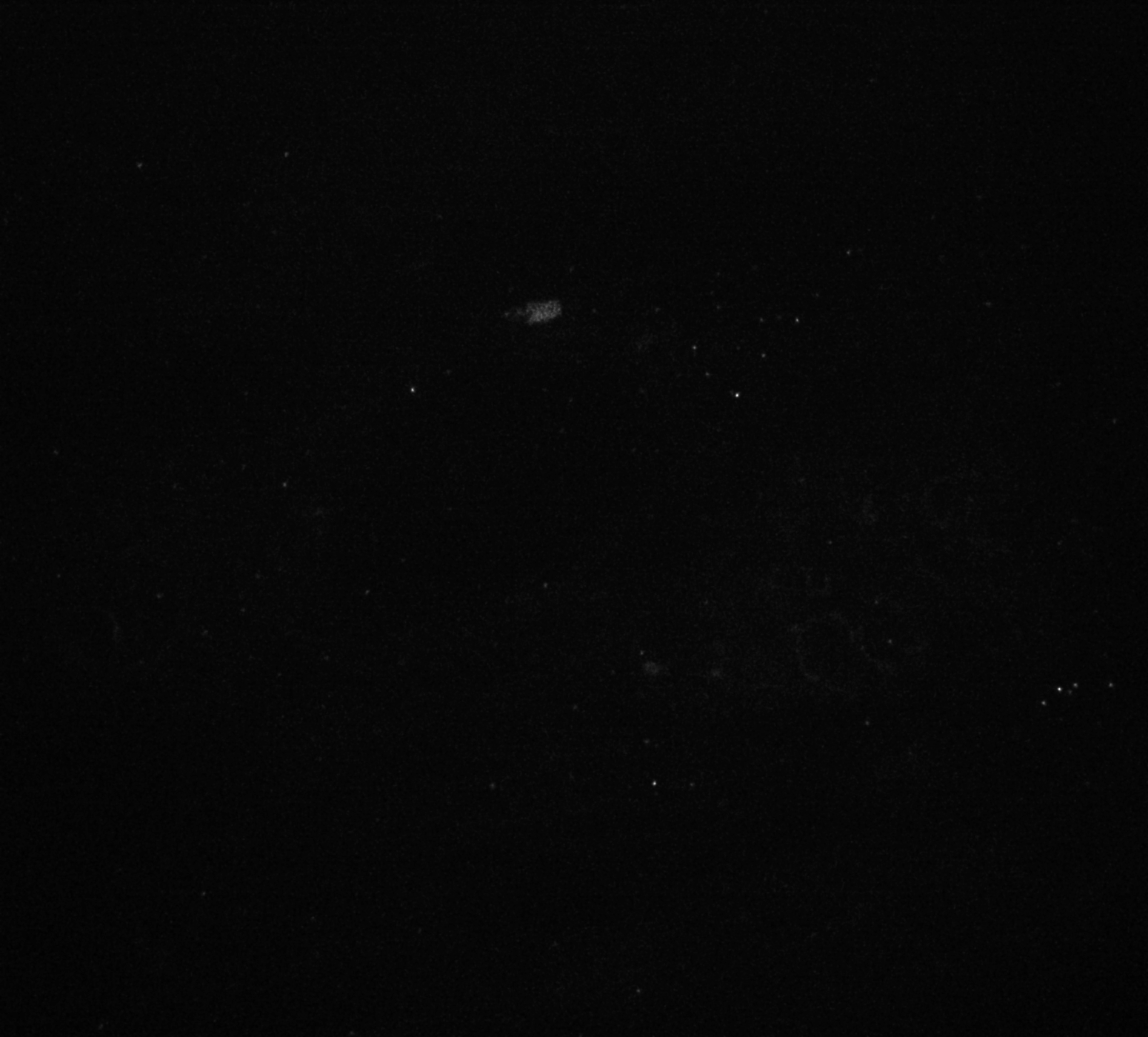

Supplement: Supplementary file 16 — Supplementary Figs. Source data [file 41467_2025_58876_MOESM16_ESM.zip › Source_mian_figures/Figure 2_Source Data/Fig 2a/FT_230911_VAND_dGRA12_HA488_GRA2_647_150x_E_1-decon/FT_230911_VAND_dGRA12_HA488_GRA2_647_150x_E_1-decon/FT_230911_VAND_dGRA12_HA488_GRA2_647_150x_E_1_MMStack_Pos0.ome.tif]

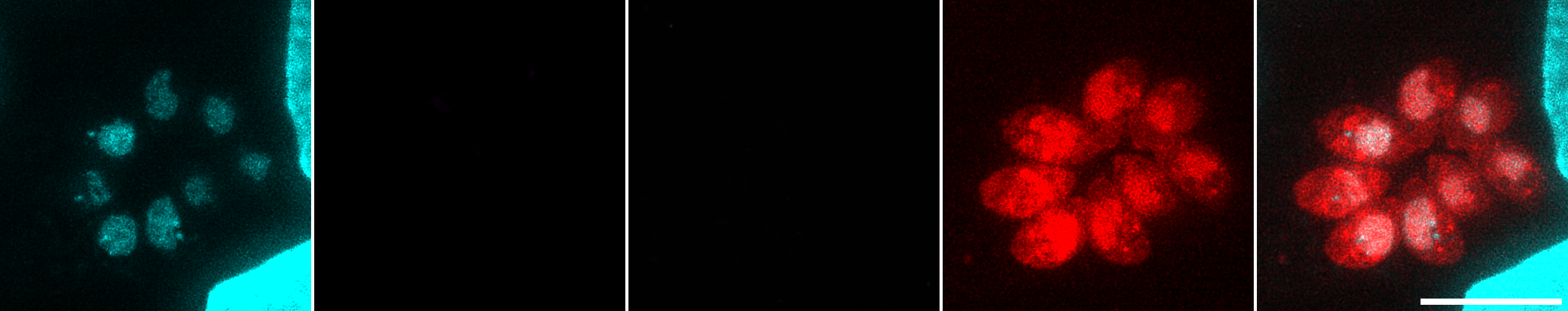

Supplement: Supplementary file 16 — Supplementary Figs. Source data [file 41467_2025_58876_MOESM16_ESM.zip › Source_mian_figures/Figure 2_Source Data/Fig 2a/FT_230911_VAND_dGRA12_HA488_GRA2_647_150x_E_1-decon/FT_230911_VAND_dGRA12_HA488_GRA2_647_150x_E_1-decon/Montage_scale_GRA2.tif]

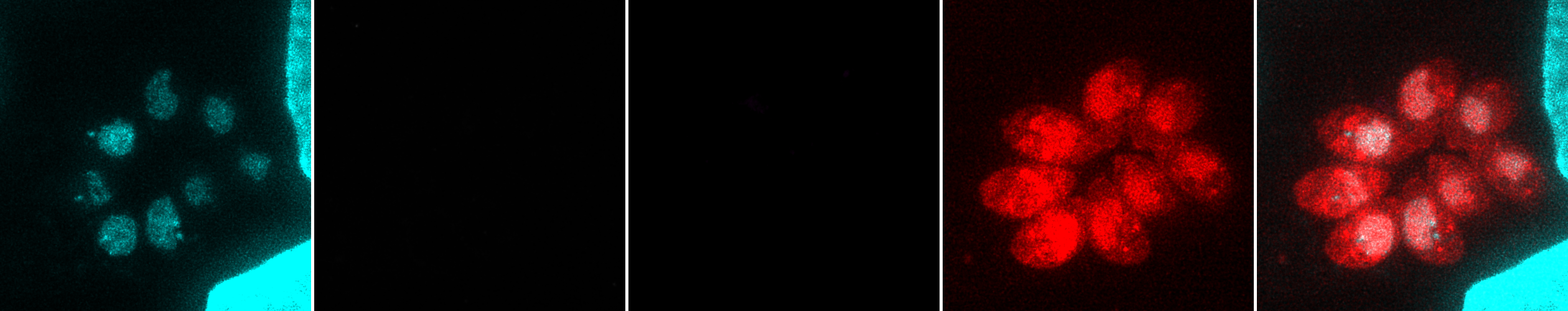

Supplement: Supplementary file 16 — Supplementary Figs. Source data [file 41467_2025_58876_MOESM16_ESM.zip › Source_mian_figures/Figure 2_Source Data/Fig 2a/FT_230911_VAND_dGRA12_HA488_GRA2_647_150x_E_1-decon/FT_230911_VAND_dGRA12_HA488_GRA2_647_150x_E_1-decon/Montage.tif]

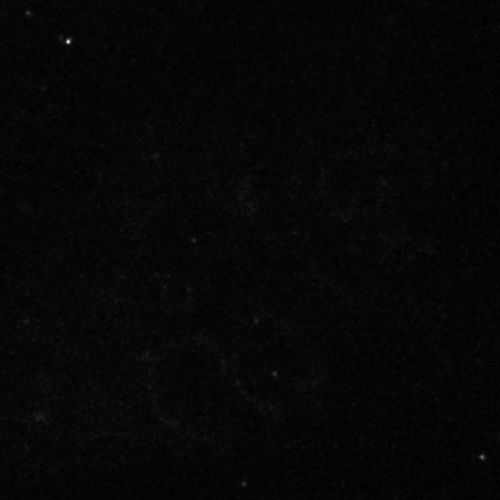

Supplement: Supplementary file 16 — Supplementary Figs. Source data [file 41467_2025_58876_MOESM16_ESM.zip › Source_mian_figures/Figure 2_Source Data/Fig 2a/FT_230911_VAND_dGRA12_HA488_GRA2_647_150x_E_1-decon/FT_230911_VAND_dGRA12_HA488_GRA2_647_150x_E_1-decon/FT_230911_VAND_dGRA12_HA488_GRA2_647_150x_E_1_MMStack_Pos0.ome-0001_HA_adj.tif]

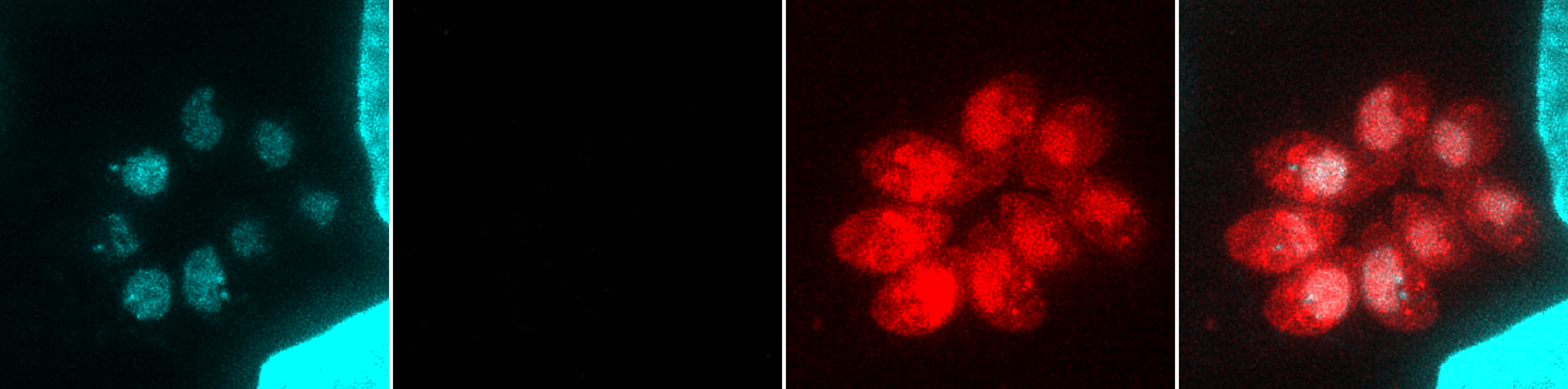

Supplement: Supplementary file 16 — Supplementary Figs. Source data [file 41467_2025_58876_MOESM16_ESM.zip › Source_mian_figures/Figure 2_Source Data/Fig 2a/FT_230911_VAND_dGRA12_HA488_GRA2_647_150x_E_1-decon/FT_230911_VAND_dGRA12_HA488_GRA2_647_150x_E_1-decon/Montage_noGRA2.tif]

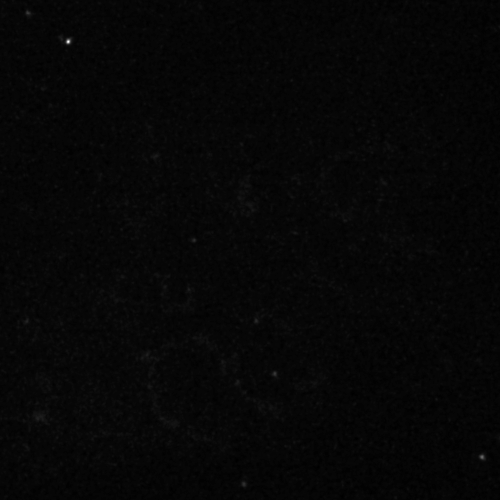

Supplement: Supplementary file 16 — Supplementary Figs. Source data [file 41467_2025_58876_MOESM16_ESM.zip › Source_mian_figures/Figure 2_Source Data/Fig 2a/FT_230911_VAND_dGRA12_HA488_GRA2_647_150x_E_1-decon/FT_230911_VAND_dGRA12_HA488_GRA2_647_150x_E_1-decon/FT_230911_VAND_dGRA12_HA488_GRA2_647_150x_E_1_MMStack_Pos0.ome-0001_HA.tif]

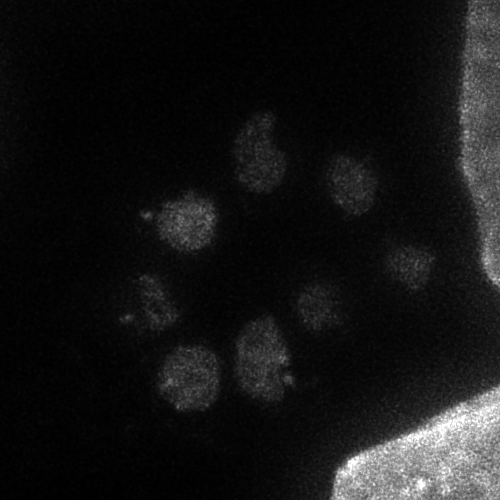

Supplement: Supplementary file 16 — Supplementary Figs. Source data [file 41467_2025_58876_MOESM16_ESM.zip › Source_mian_figures/Figure 2_Source Data/Fig 2a/FT_230911_VAND_dGRA12_HA488_GRA2_647_150x_E_1-decon/FT_230911_VAND_dGRA12_HA488_GRA2_647_150x_E_1-decon/FT_230911_VAND_dGRA12_HA488_GRA2_647_150x_E_1_MMStack_Pos0.ome-0002_DAPI.tif]

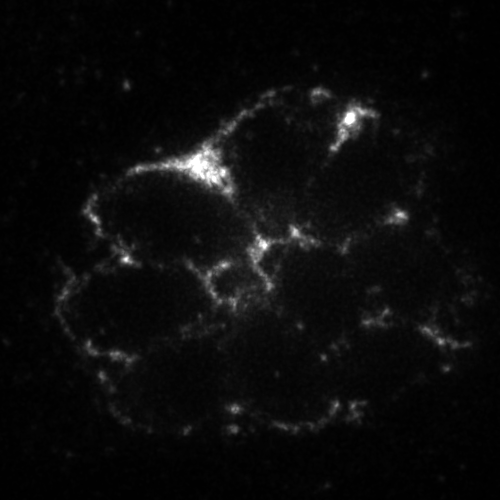

Supplement: Supplementary file 16 — Supplementary Figs. Source data [file 41467_2025_58876_MOESM16_ESM.zip › Source_mian_figures/Figure 2_Source Data/Fig 2a/FT_230911_VAND_dGRA12_HA488_GRA2_647_150x_E_1-decon/FT_230911_VAND_dGRA12_HA488_GRA2_647_150x_E_1-decon/FT_230911_VAND_dGRA12_HA488_GRA2_647_150x_E_1_MMStack_Pos0.ome-0003_GRA2.tif]

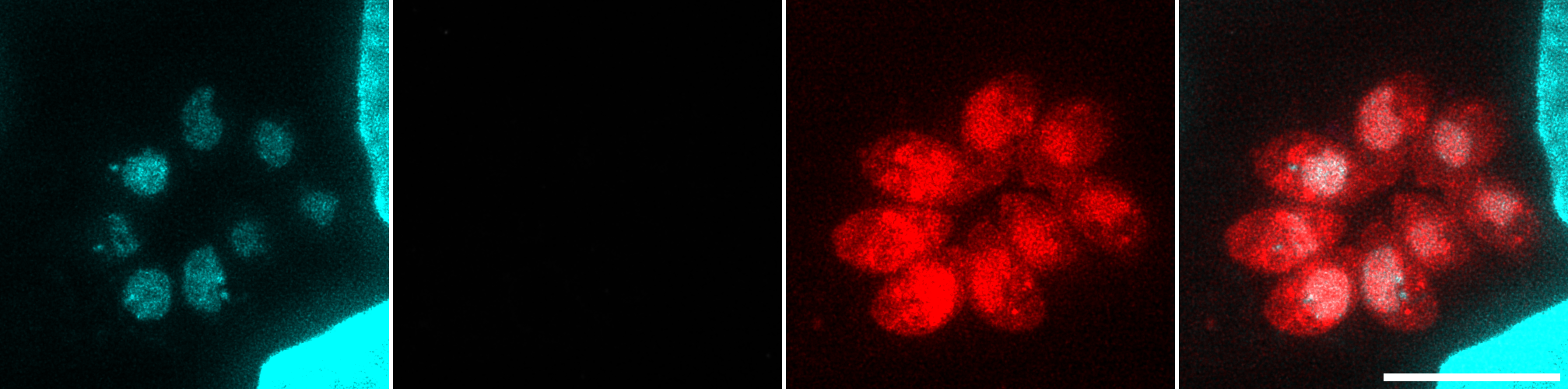

Supplement: Supplementary file 16 — Supplementary Figs. Source data [file 41467_2025_58876_MOESM16_ESM.zip › Source_mian_figures/Figure 2_Source Data/Fig 2a/FT_230911_VAND_dGRA12_HA488_GRA2_647_150x_E_1-decon/FT_230911_VAND_dGRA12_HA488_GRA2_647_150x_E_1-decon/Montage_scale.tif]

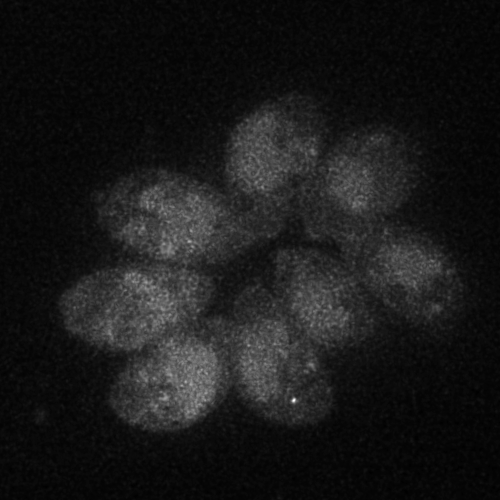

Supplement: Supplementary file 16 — Supplementary Figs. Source data [file 41467_2025_58876_MOESM16_ESM.zip › Source_mian_figures/Figure 2_Source Data/Fig 2a/FT_230911_VAND_dGRA12_HA488_GRA2_647_150x_E_1-decon/FT_230911_VAND_dGRA12_HA488_GRA2_647_150x_E_1-decon/FT_230911_VAND_dGRA12_HA488_GRA2_647_150x_E_1_MMStack_Pos0.ome-0004_toxo.tif]

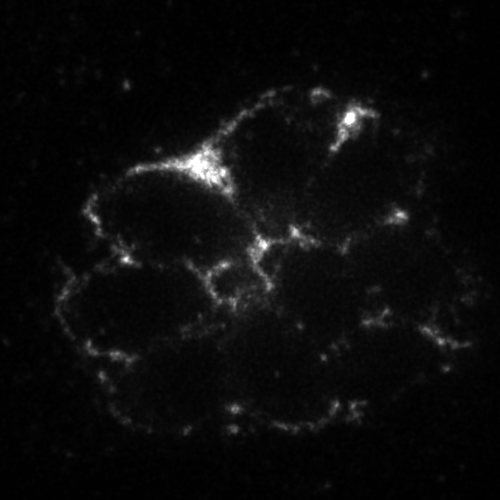

Supplement: Supplementary file 16 — Supplementary Figs. Source data [file 41467_2025_58876_MOESM16_ESM.zip › Source_mian_figures/Figure 2_Source Data/Fig 2a/FT_230911_VAND_dGRA12_HA488_GRA2_647_150x_E_1-decon/FT_230911_VAND_dGRA12_HA488_GRA2_647_150x_E_1-decon/FT_230911_VAND_dGRA12_HA488_GRA2_647_150x_E_1_MMStack_Pos0.ome-0003_GRA2_adj.tif]

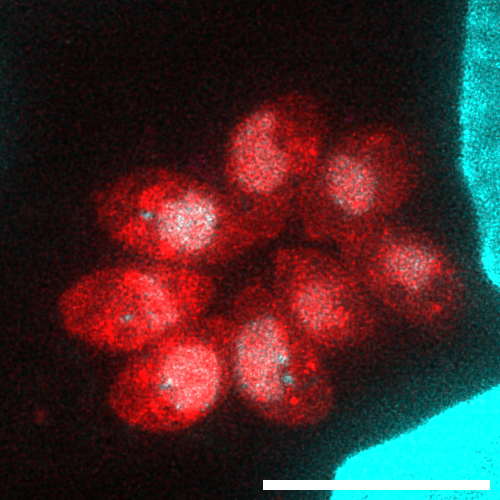

Supplement: Supplementary file 16 — Supplementary Figs. Source data [file 41467_2025_58876_MOESM16_ESM.zip › Source_mian_figures/Figure 2_Source Data/Fig 2a/FT_230911_VAND_dGRA12_HA488_GRA2_647_150x_E_1-decon/FT_230911_VAND_dGRA12_HA488_GRA2_647_150x_E_1-decon/MERGE_SCALE.tif]

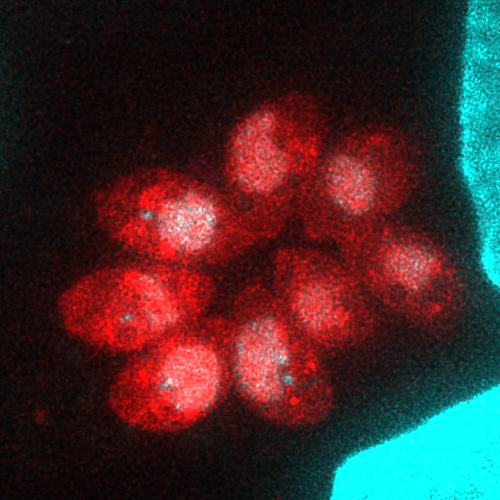

Supplement: Supplementary file 16 — Supplementary Figs. Source data [file 41467_2025_58876_MOESM16_ESM.zip › Source_mian_figures/Figure 2_Source Data/Fig 2a/FT_230911_VAND_dGRA12_HA488_GRA2_647_150x_E_1-decon/FT_230911_VAND_dGRA12_HA488_GRA2_647_150x_E_1-decon/MERGE.tif]

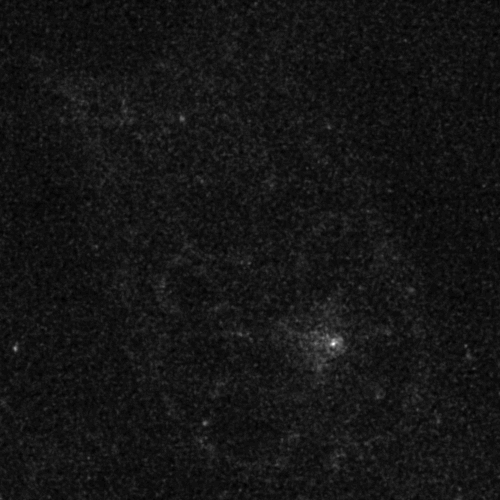

Supplement: Supplementary file 16 — Supplementary Figs. Source data [file 41467_2025_58876_MOESM16_ESM.zip › Source_mian_figures/Figure 2_Source Data/Fig 2a/FT_230911_VAND_dKU_HA488_GRA2_647_150x_D_1-decon/FT_230911_VAND_dKU_HA488_GRA2_647_150x_D_1-decon/FT_230911_VAND_dKU_HA488_GRA2_647_150x_D_1_MMStack_Pos0.ome-0001_HA_adj.tif]

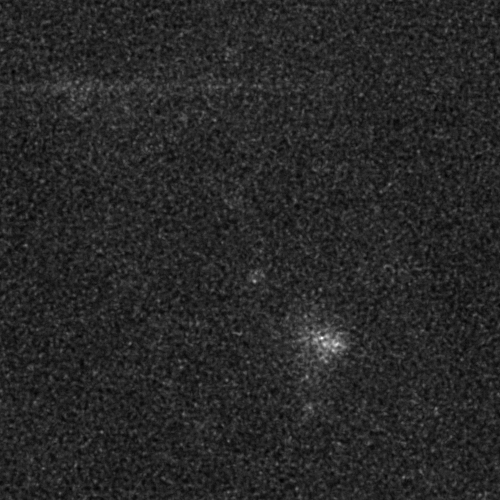

Supplement: Supplementary file 16 — Supplementary Figs. Source data [file 41467_2025_58876_MOESM16_ESM.zip › Source_mian_figures/Figure 2_Source Data/Fig 2a/FT_230911_VAND_dKU_HA488_GRA2_647_150x_D_1-decon/FT_230911_VAND_dKU_HA488_GRA2_647_150x_D_1-decon/FT_230911_VAND_dKU_HA488_GRA2_647_150x_D_1_MMStack_Pos0.ome-0004_toxo_adj.tif]

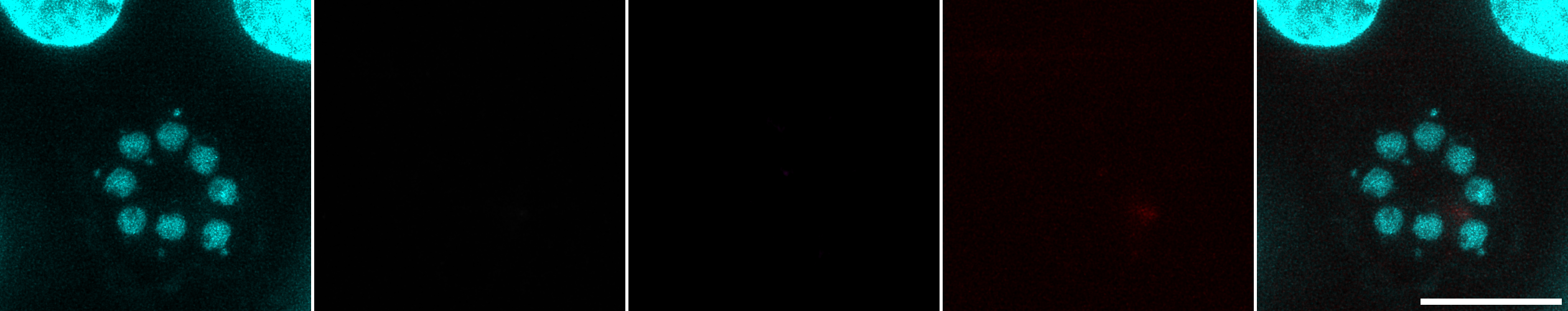

Supplement: Supplementary file 16 — Supplementary Figs. Source data [file 41467_2025_58876_MOESM16_ESM.zip › Source_mian_figures/Figure 2_Source Data/Fig 2a/FT_230911_VAND_dKU_HA488_GRA2_647_150x_D_1-decon/FT_230911_VAND_dKU_HA488_GRA2_647_150x_D_1-decon/Montage_scale_GRA2.tif]

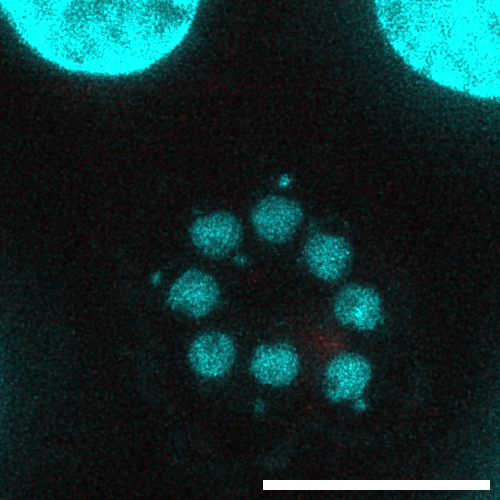

Supplement: Supplementary file 16 — Supplementary Figs. Source data [file 41467_2025_58876_MOESM16_ESM.zip › Source_mian_figures/Figure 2_Source Data/Fig 2a/FT_230911_VAND_dKU_HA488_GRA2_647_150x_D_1-decon/FT_230911_VAND_dKU_HA488_GRA2_647_150x_D_1-decon/MERGE_noGRA2_scale.tif]

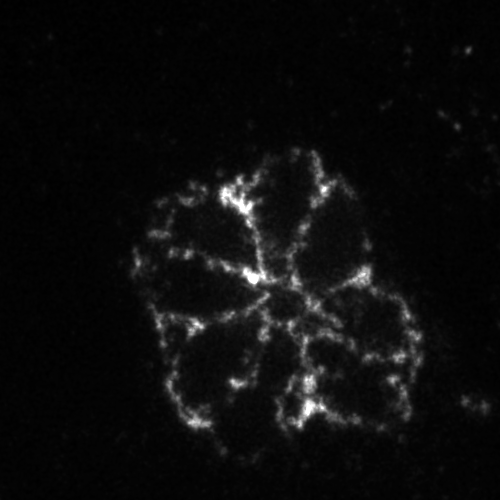

Supplement: Supplementary file 16 — Supplementary Figs. Source data [file 41467_2025_58876_MOESM16_ESM.zip › Source_mian_figures/Figure 2_Source Data/Fig 2a/FT_230911_VAND_dKU_HA488_GRA2_647_150x_D_1-decon/FT_230911_VAND_dKU_HA488_GRA2_647_150x_D_1-decon/FT_230911_VAND_dKU_HA488_GRA2_647_150x_D_1_MMStack_Pos0.ome-0003_GRA2_adj.tif]

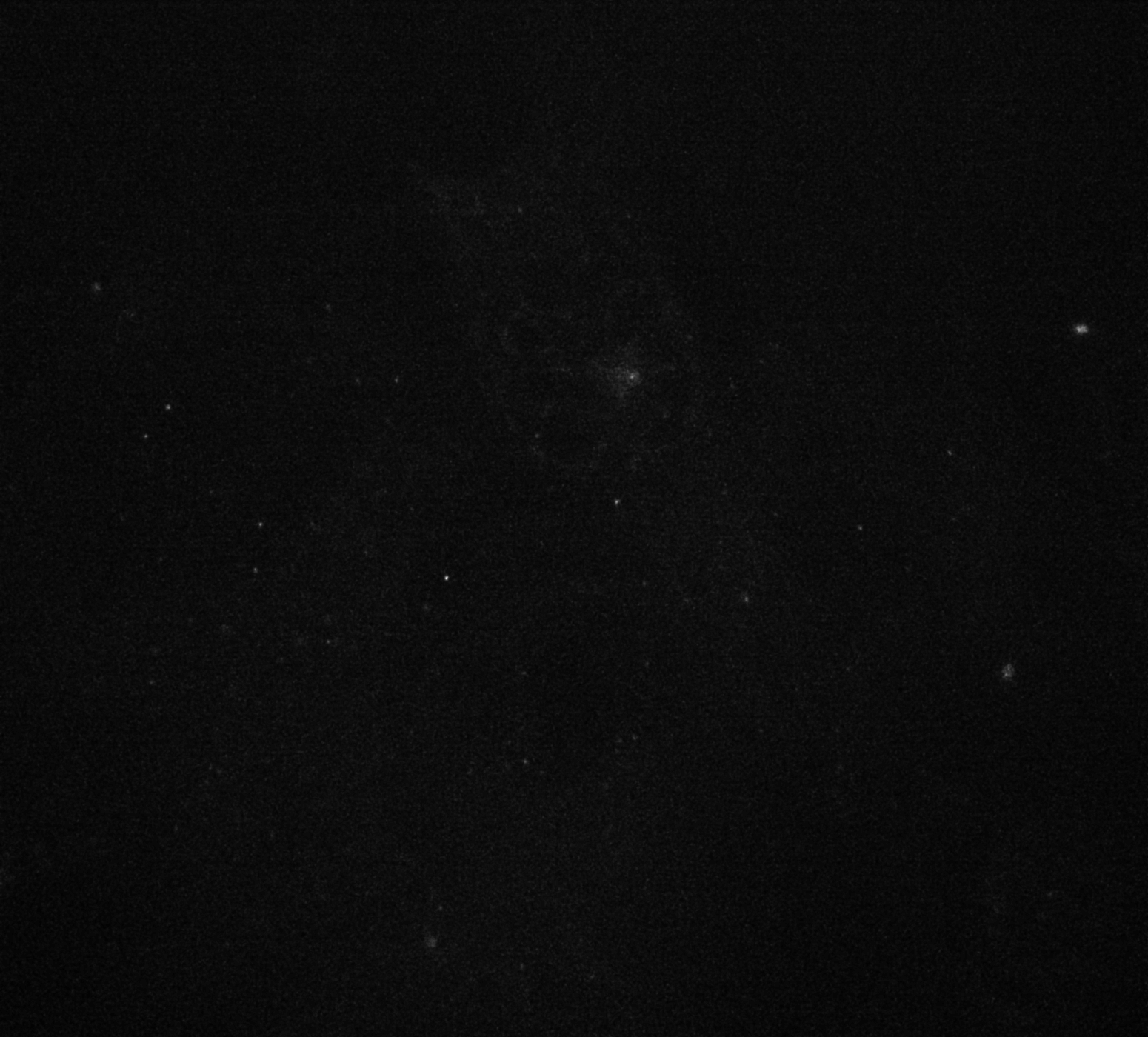

Supplement: Supplementary file 16 — Supplementary Figs. Source data [file 41467_2025_58876_MOESM16_ESM.zip › Source_mian_figures/Figure 2_Source Data/Fig 2a/FT_230911_VAND_dKU_HA488_GRA2_647_150x_D_1-decon/FT_230911_VAND_dKU_HA488_GRA2_647_150x_D_1-decon/FT_230911_VAND_dKU_HA488_GRA2_647_150x_D_1_MMStack_Pos0.ome.tif]

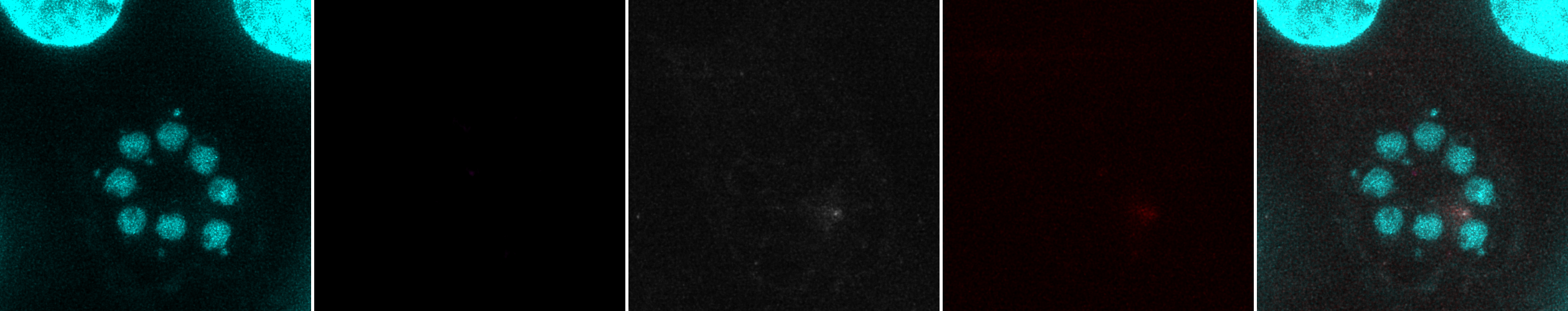

Supplement: Supplementary file 16 — Supplementary Figs. Source data [file 41467_2025_58876_MOESM16_ESM.zip › Source_mian_figures/Figure 2_Source Data/Fig 2a/FT_230911_VAND_dKU_HA488_GRA2_647_150x_D_1-decon/FT_230911_VAND_dKU_HA488_GRA2_647_150x_D_1-decon/Montage.tif]

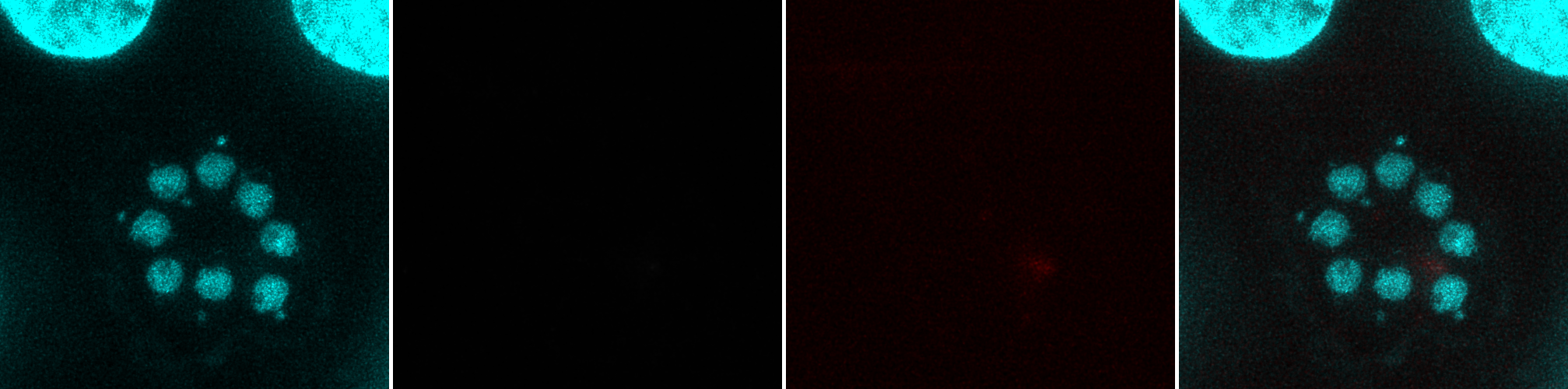

Supplement: Supplementary file 16 — Supplementary Figs. Source data [file 41467_2025_58876_MOESM16_ESM.zip › Source_mian_figures/Figure 2_Source Data/Fig 2a/FT_230911_VAND_dKU_HA488_GRA2_647_150x_D_1-decon/FT_230911_VAND_dKU_HA488_GRA2_647_150x_D_1-decon/Montage_noGRA2.tif]

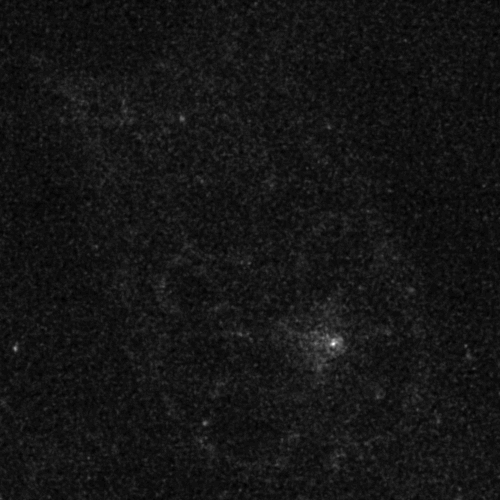

Supplement: Supplementary file 16 — Supplementary Figs. Source data [file 41467_2025_58876_MOESM16_ESM.zip › Source_mian_figures/Figure 2_Source Data/Fig 2a/FT_230911_VAND_dKU_HA488_GRA2_647_150x_D_1-decon/FT_230911_VAND_dKU_HA488_GRA2_647_150x_D_1-decon/FT_230911_VAND_dKU_HA488_GRA2_647_150x_D_1_MMStack_Pos0.ome-0001_HA.tif]

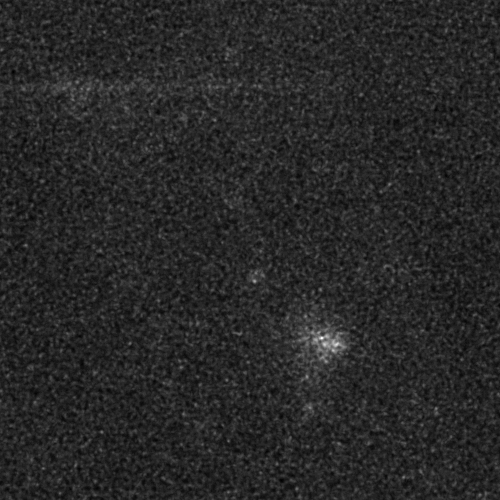

Supplement: Supplementary file 16 — Supplementary Figs. Source data [file 41467_2025_58876_MOESM16_ESM.zip › Source_mian_figures/Figure 2_Source Data/Fig 2a/FT_230911_VAND_dKU_HA488_GRA2_647_150x_D_1-decon/FT_230911_VAND_dKU_HA488_GRA2_647_150x_D_1-decon/FT_230911_VAND_dKU_HA488_GRA2_647_150x_D_1_MMStack_Pos0.ome-0004_toxo.tif]

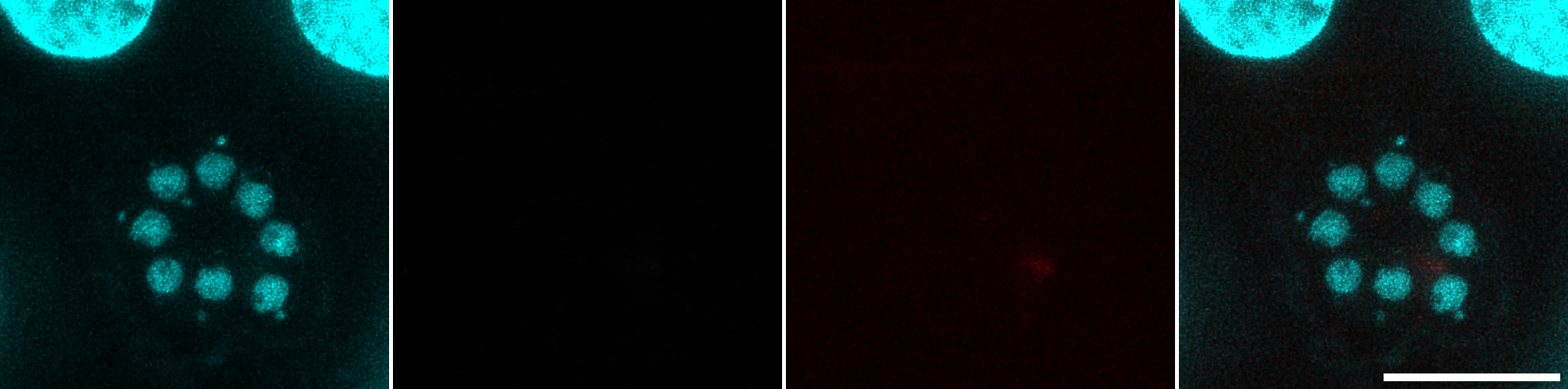

Supplement: Supplementary file 16 — Supplementary Figs. Source data [file 41467_2025_58876_MOESM16_ESM.zip › Source_mian_figures/Figure 2_Source Data/Fig 2a/FT_230911_VAND_dKU_HA488_GRA2_647_150x_D_1-decon/FT_230911_VAND_dKU_HA488_GRA2_647_150x_D_1-decon/Montage_scale.tif]

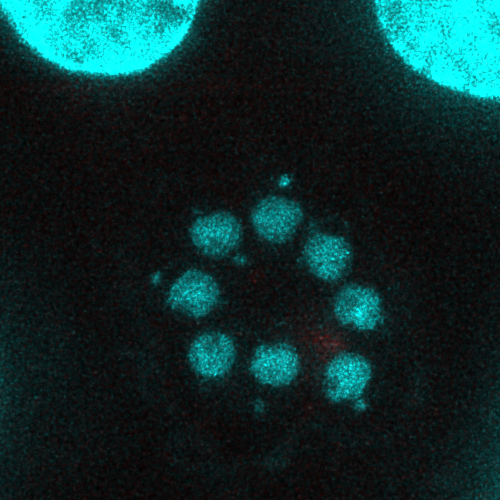

Supplement: Supplementary file 16 — Supplementary Figs. Source data [file 41467_2025_58876_MOESM16_ESM.zip › Source_mian_figures/Figure 2_Source Data/Fig 2a/FT_230911_VAND_dKU_HA488_GRA2_647_150x_D_1-decon/FT_230911_VAND_dKU_HA488_GRA2_647_150x_D_1-decon/MERGE_noGRA2.tif]

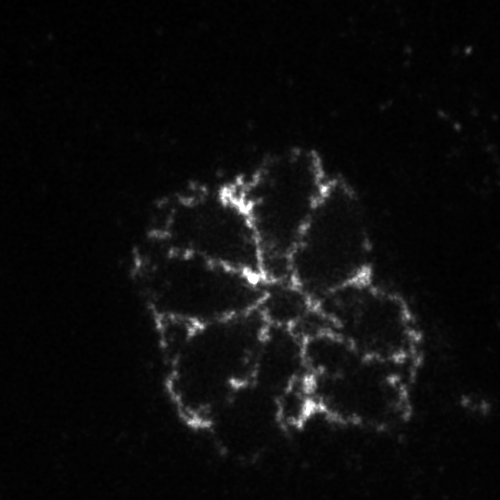

Supplement: Supplementary file 16 — Supplementary Figs. Source data [file 41467_2025_58876_MOESM16_ESM.zip › Source_mian_figures/Figure 2_Source Data/Fig 2a/FT_230911_VAND_dKU_HA488_GRA2_647_150x_D_1-decon/FT_230911_VAND_dKU_HA488_GRA2_647_150x_D_1-decon/FT_230911_VAND_dKU_HA488_GRA2_647_150x_D_1_MMStack_Pos0.ome-0003_GRA2.tif]

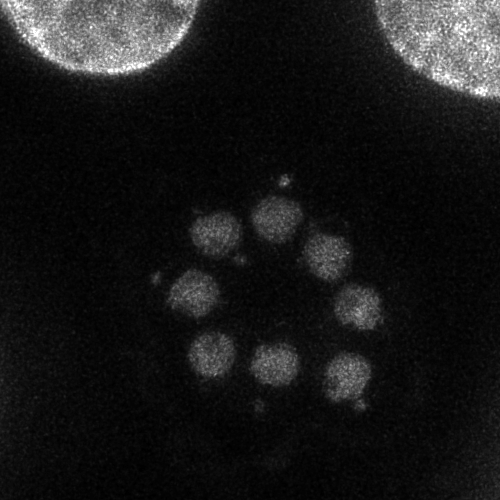

Supplement: Supplementary file 16 — Supplementary Figs. Source data [file 41467_2025_58876_MOESM16_ESM.zip › Source_mian_figures/Figure 2_Source Data/Fig 2a/FT_230911_VAND_dKU_HA488_GRA2_647_150x_D_1-decon/FT_230911_VAND_dKU_HA488_GRA2_647_150x_D_1-decon/FT_230911_VAND_dKU_HA488_GRA2_647_150x_D_1_MMStack_Pos0.ome-0002_DAPI.tif]

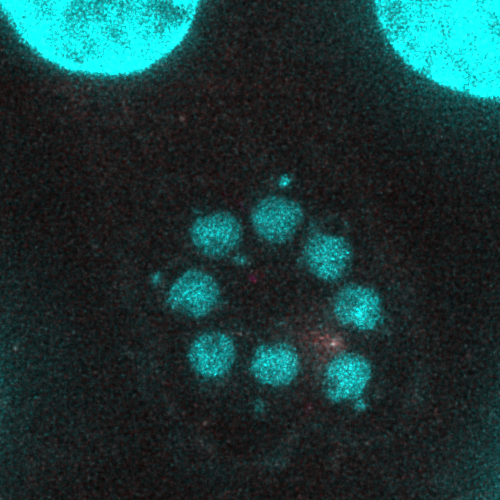

Supplement: Supplementary file 16 — Supplementary Figs. Source data [file 41467_2025_58876_MOESM16_ESM.zip › Source_mian_figures/Figure 2_Source Data/Fig 2a/FT_230911_VAND_dKU_HA488_GRA2_647_150x_D_1-decon/FT_230911_VAND_dKU_HA488_GRA2_647_150x_D_1-decon/MERGE.tif]
